# Facile and diastereoselective arylation of the privileged 1,4-dihydroisoquinolin-3(2*H*)-one scaffold

**DOI:** 10.3762/bjoc.18.109

**Published:** 2022-08-22

**Authors:** Dmitry Dar’in, Grigory Kantin, Alexander Bunev, Mikhail Krasavin

**Affiliations:** 1 Saint Petersburg State University, Saint Petersburg 199034, Russian Federation; 2 Medicinal Chemistry Center, Togliatti State University, 445020 Togliatti, Russian Federation; 3 Immanuel Kant Baltic Federal University, Kaliningrad 236016, Russian Federation

**Keywords:** 1,4-dihydroisoquinol-3-one, heterocyclic diazo compounds, hydroarylation, Regitz diazo transfer, triflic acid

## Abstract

A practically convenient and streamlined protocol for the *trans*-diastereoselective introduction of an aryl substituent at position 4 of the 1,4-dihydroisoquinol-3-one (1,4-DHIQ) scaffold is presented. The protocol involves direct Regitz diazo transfer onto readily available 3(2*H*)-isoquinolones followed by TfOH-promoted hydroarylation by an arene molecule. Screening of the novel 1,2,4-trisubstituted 1,4-DHIQs against cancer cell lines confirmed high cytotoxicity of selected analogs, which validates this new chemotype for further investigations as anticancer cytotoxic agents.

## Introduction

**Figure 1:**
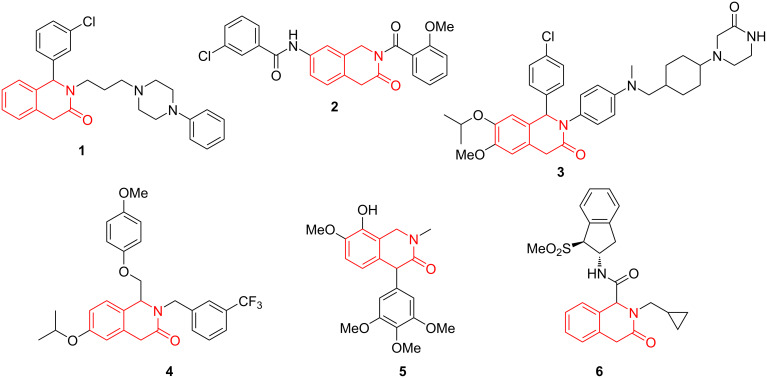
Diverse bioactive compounds based on the privileged 1,4-DHIQ scaffold.

**Figure 2:**
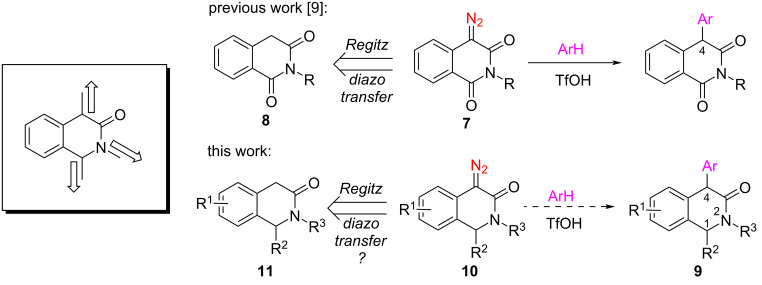
Strategy investigated in this work.

Besides being derivatives of (or a precursor to) the 1,2,3,4-tetrahydroisoquinoline core which itself bears a special significance from the standpoint of associated biological activities and relevance to the naturally occurring alkaloids [1], 1,4-dihydro-3(2*H*)-isoquinolones (1,4-DHIQs) undoubtedly represent a privileged scaffold [2] for drug design considering such diversely bioactive compounds documented in the literature as ligand for serotonin 5-HT_1A_ receptors **1** [3], AChE and BACE-1 inhibitor **2** [4], inhibitor of oncogenic p53-MDM2 protein–protein interaction **3** [5], positive allosteric modulator of ionotropic glutamate receptor NMDA-1 **4** [6], insulin-like growth factor 1 receptor inhibitor **5** [7], and metabotropic glutamate receptor 7 modulator **6** [8] (Figure 1). The privileged 1,4-DHIQs would be a highly suitable platform for a stereodefined presentation of three different diversity vectors of the lactam moiety. However, the methods for the preparation of 1,4-DHIQs with convenient and independent variation of the three lactam substituents are absent in the literature. While pondering possible solutions to fill this void, we drew inspiration in our recent success achieving direct Brønsted acid-catalyzed *C*-arylation of 4-diazo-isoquinoline-1,3-diones **7** [9] which are, in turn, obtainable via the Regitz diazo transfer reaction onto readily available homophthalimides **8** [10–12]. We reasoned that a similar strategy could be adopted for the preparation of 1,2,4-trisubstituted 1,4-DHIQs **9** if access to their diazo precursors **10** was gained. *N*-Sulfonyl analogs of compounds **10** have recently been synthesized via an innovative Dimroth rearrangement of 4-diazoisochroman-3-imines [13] and employed in several acid- and metal-promoted transformations by Lu, Wang et al. [14–18]. However, preparation of precursor **10** by direct diazo transfer onto the methylene group of readily available [19–20] 3(2*H*)-isoquinolones **11** has not been described in the literature. Lured by the prospects of applying a diazo chemistry route to an expedited synthesis of hitherto undescribed 1,2,4-trisubstituted 1,4-DHIQs **9** from **11**, we set off to investigate the obtainability of **10** from **11** by the Regitz [21] diazo transfer and the usage of **10** in acid-promoted direct arylation by aromatic hydrocarbons (Figure 2) [13]. Herein, we describe the results obtained in the course of this investigation.

## Results and Discussion

**Scheme 1:**
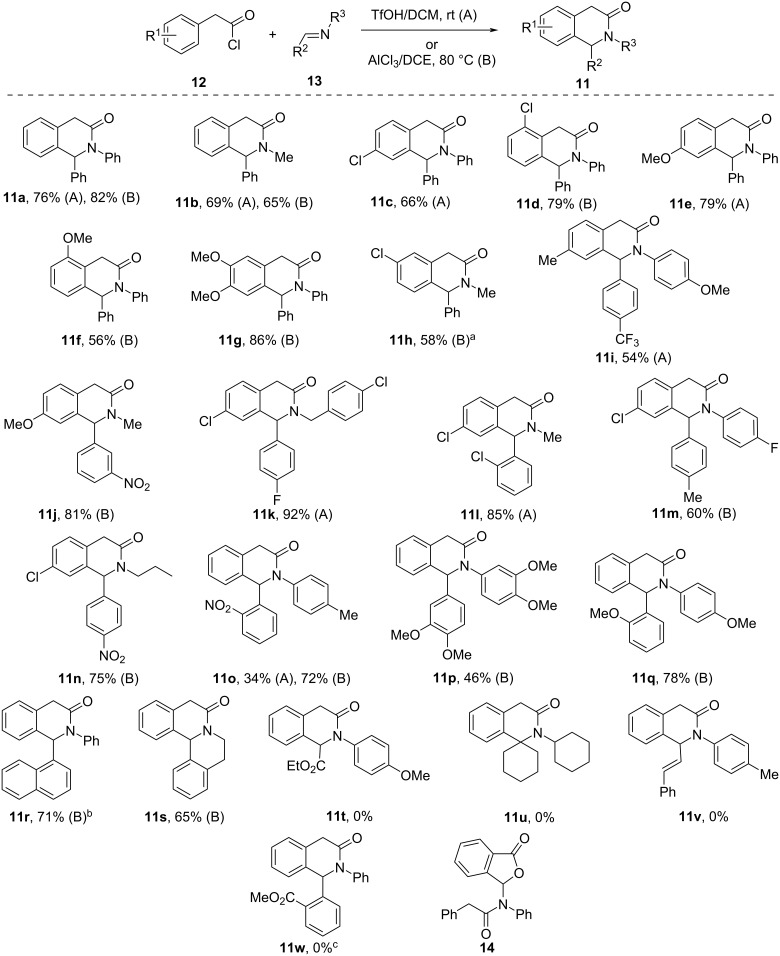
Preparation of 3(2*H*)-isoquinolones **11**. ^a^Obtained as a 10:1 mixture of regioisomers; purified by crystallization. ^b^Employed in the next step without purification (not characterized). ^c^Compound **14** was identified as the reaction product.

We began our investigation by synthesizing a sufficiently broad range of 3(2*H*)-isoquinolones **11** from phenylacetyl chlorides **12** and Schiff bases **13** using conditions described in the literature [19–20]. It was soon established that room-temperature TfOH-promoted cyclocondensation (method A) [19] and AlCl_3_-promoted reaction conducted at elevated temperature (method B) can be employed interchangeably, with yields registered for 3(2*H*)-isoquinolones **11** mostly from good to excellent. While the scope of the 3(2*H*)-isoquinolone synthesis was substantially expanded by these findings compared to the previously reported results [21], certain limitations were also noted. For example, attempts to involve imines derived from ethyl glyoxylate, cyclohexanone, and cinnamaldehyde gave no desired product (**11t–v**). Quite surprisingly, the Schiff base derived from methyl *o*-formylbenzoate also gave no desired product while cyclization led to isobenzofuran-1(3*H*)-one **14** (Scheme 1).

**Scheme 2:**
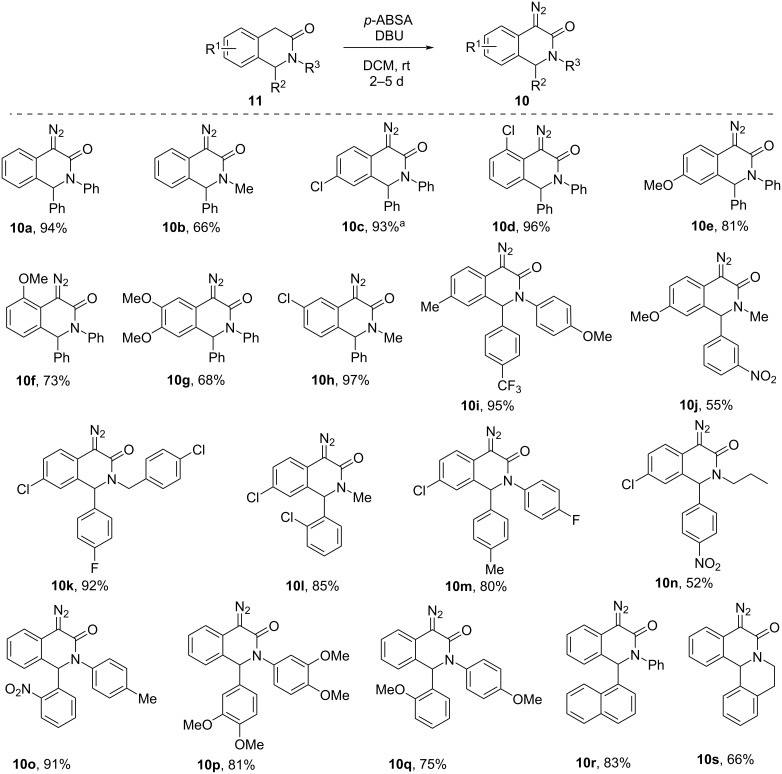
Preparation of 4-diazo-3(2*H*)-isoquinolones **10**. ^a^Confirmed by single-crystal X-ray crystallography (see Supporting Information File 1).

Having secured a supply of diversely substituted 3(2*H*)-isoquinolones **11a**–**s**, we proceeded to investigate their suitability as substrates in the Regitz diazo transfer. We reasoned that if the C–H acidity of the methylene group in **11** would turn out to be insufficient for the Regitz protocol to be directly applied, these substrates could have been additionally activated by trifluoroacetylation (Danheiser method [22]) or ethoxalylation [23]. Fortunately, all of the substrates **11a**–**s** were converted cleanly and smoothly over 2–5 days into their diazo derivatives **10a**–**s** using *p*-(acetamido)benzenesulfonyl azide (*p*-ABSA) as the diazo group donor [24] and DBU as the base. The yields of diazo compounds **10** were generally good to excellent throughout. The notable exception is the evident drop in the isolated yield in the case of substrates bearing a nitrophenyl group at position 1 (cf. compounds **10j** and **10n**). This lowering of the yield, however, likely has to do with the combination of the nitrophenyl substituent and an *N*-alkyl group (considering that *N*-aryl nitrophenyl-substituted compound **10o** was obtained in a respectable 91% yield). The structures of compounds **10a**–**s** were unequivocally confirmed by ^1^H and ^13^C NMR spectroscopy (paying a particular attention to the appearance of the *C*=N_2_ signal in the spectrum), mass spectrometry and, in the case of compound **10c**, by single-crystal X-ray crystallography (Scheme 2).

Using compound **10a** as the model substrate, we proceeded to screen for suitable reaction conditions that would allow involving this kind of diazo compounds in the TfOH-promoted benzene *C*-arylation reaction (Table 1) [13]. The excess of benzene was varied in the range 10 to 60 equiv and the yield of product **9a** was found to improve from 63% to 83% which also allowed lowering the excess of triflic acid to 1.5 equiv. Product **9a** was in all cases obtained after only 15 min reaction with high diastereoselectivity and the principal diastereomer was rightly deemed to be *trans*-configured (vide infra). The order of mixing the reagents was found to be crucial for the successful arylation of compound **10a**. Specifically, the arylation was conducted on adding a DCM solution of substrate **10a** to a vigorously stirred mixture of benzene and triflic acid. On the contrary, when adding TfOH to a solution of **10a** in benzene, the formation of a complex mixture of unidentifiable products was observed, with only a trace amount of **9a** detectable by ^1^H NMR.

**Table 1:** Conditions screening for the TfOH-promoted arylation of diazo substrate **10a**.^a^

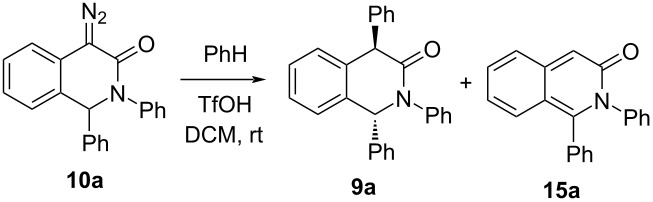				

Entry	PhH (equiv)	TfOH (equiv)	% Yield of **9a** (dr)	% Yield of **15a**

1	10	2.0	63% (98:2)	26%
2	20	2.0	73% (98:2)	20%
3	60	2.0	80% (98:2)	8%
**4**	**60**	**1.5**	**83%** (98:2)	**13%**
5	60	1.0	65% (96:4)	19%
6	0	1.1	–	51%

^a^Concentration: 0.15 M, scale: 0.3 mmol.

The formation of product **9a** was unavoidably accompanied by a varying amount of byproduct **15a** (vide infra for the mechanistic reasoning for its formation) which demonstrated, along with other analogs of **15** which were isolated and characterized, limited chemical stability and deteriorated on prolonged standing as a solution in CDCl_3_ at ambient temperature. Compound **15a** was the exclusive isolable product when benzene was eliminated from the reaction mixture (Table 1, entry 6). Interestingly, the formation of byproduct **15** (which could be, in principle, obtained by DDQ oxidation of **11** [25]) via the elimination of a diazo group has not been reported.

**Scheme 3:**
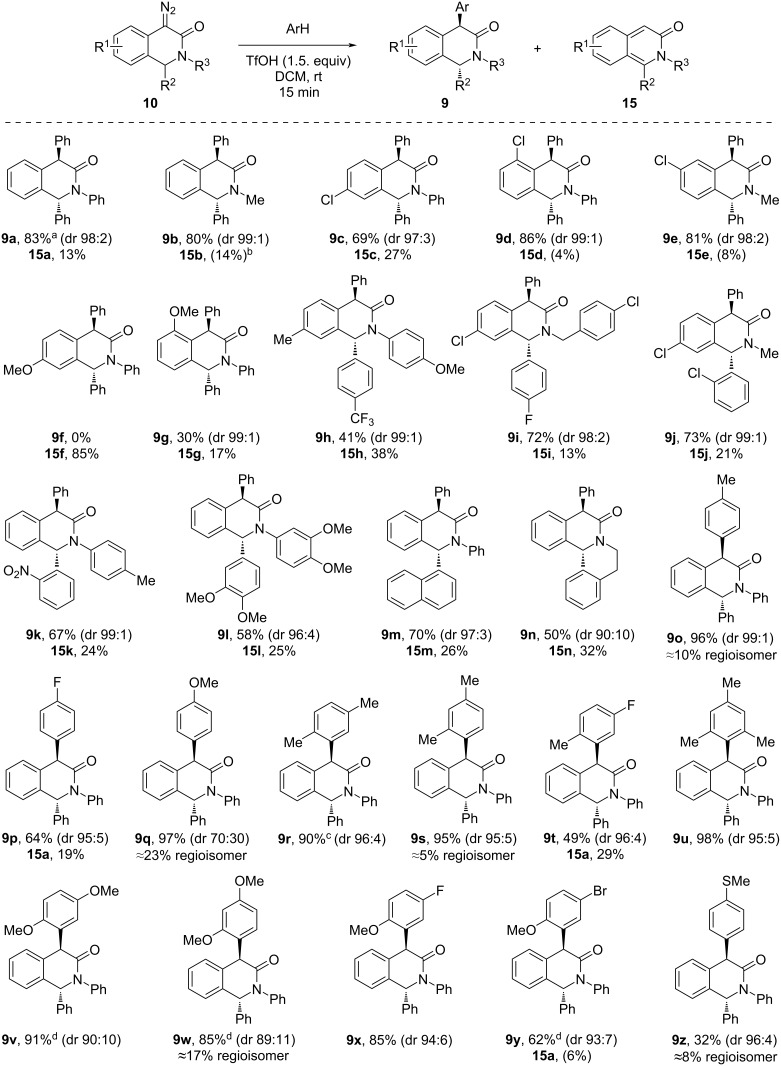
TfOH-promoted arylation of diazo substrates **10**. ^a^Structure confirmed by single-crystal X-ray analysis. ^b^Yields in parentheses estimated by ^1^H NMR. ^c^Run on 5 × scale (1.5 mmol) with 88% yield. ^d^5.0 equiv of arene was used.

Having identified the optimum conditions for the *C*-arylation of diazo substrates **10** (60 equiv ArH, 1.5 equiv TfOH, DCM, rt, 15 min), we proceeded to investigate the scope of this transformation for 4-diazo-3(2*H*)-isoquinolones **10a**–**s** as well as various arenes (Scheme 3). In all cases, this (generally high-yielding) transformation resulted in the highly diastereoselective formation of *C*-arylation products **10** which in some cases was accompanied by a regioisomer formation (with respect to the entering arene moiety) and the formation of 3-isoquinolones **15** (isolated and characterized in several instances). Not unexpectedly, less electron-rich arenes furnished higher amounts of the 3-isoquinolone **15** byproduct compared to their electron-rich counterparts (cf. **9o** vs **9p**).

Likewise, electron-donating substituents in the 4-diazo-3(2*H*)-isoquinolone benzene ring increased the tendency of 3-isoquinolones **15** to form (cf. **9**(**15**)**f**–**h**). Using more reactive (electron-rich) arenes results in lower diastereoselectivity and regiospecificity of the reaction (cf. **9q**, **9v**, and **9w**). The structure and the initially anticipated *trans*-configuration of the products **9** was unequivocally confirmed by ^1^H and ^13^C NMR spectroscopy as well as, in the case of compound **9a**, by single-crystal X-ray analysis.

**Scheme 4:**
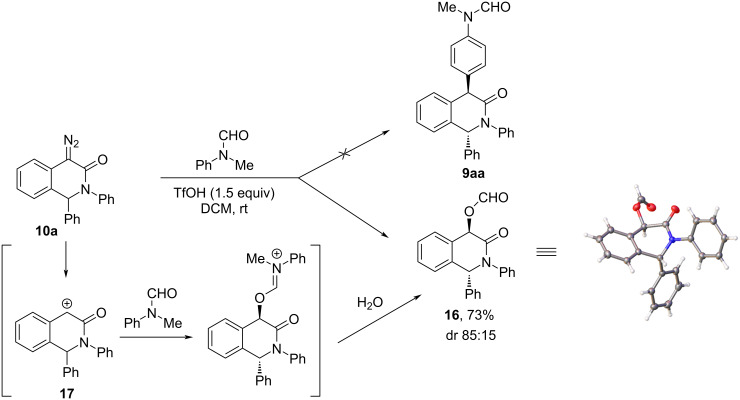
Unexpected outcome of the TfOH-promoted arylation of **10a** with *N*-formyl-*N*-methylaniline giving rise to ester **16**.

A curious and somewhat unexpected result was obtained when trying to employ *N*-formyl-*N*-methylaniline as an arene in the TfOH-promoted arylation of **10a**. Instead of the anticipated product **9aa**, 73% yield of predominantly *trans*-configured formate ester **16** was obtained and its structure was confirmed by single-crystal X-ray analysis. Presumably, the formation of ester **16** can be justified by the trapping of the carbocation intermediate **17** (vide infra) by the formamide carbonyl oxygen atom followed by hydrolysis of the iminium moiety (Scheme 4).

**Scheme 5:**
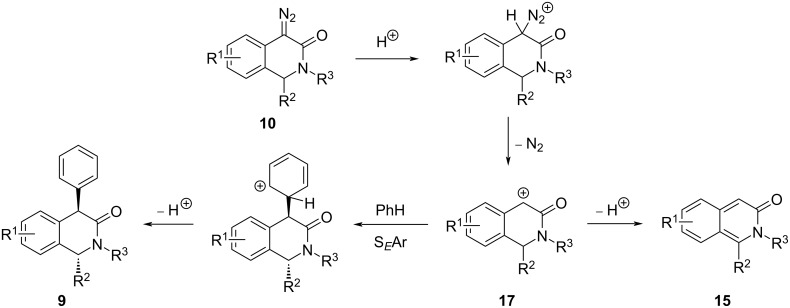
Plausible mechanism for the conversion of diazo substrates **10** to 4-aryl products **9** (shown for ArH = benzene) and 3-isoquinoline byproducts **15**.

Mechanistically, the arylation of diazo substrates **10** likely proceeds via the protonation of the diazo moiety and elimination of a nitrogen molecule, whereby carbocation **17** is generated. The latter can either be deprotonated to give byproduct **15** or be intercepted by an arene molecule in S*_E_*Ar fashion to give arylation product **9**. The *trans*-diastereoselectivity in the latter process is reasonably justified by the approach of the arene molecule to carbocation **17** from the sterically less hindered side (Scheme 5).

Compounds **9** have a pronounced three-dimensional character which makes this novel chemotype a promising probe for protein–protein interactions, including oncogenic ones [26]. As the first step towards biological characterization of compounds **9**, they were screened for cytotoxicity against the NCI-H460 lung carcinoma cell line. The most potent cytotoxic agent (**9j**) reduced the number of viable cells by >95% at 30 μM concentration. Dose–response testing of this compound against both NCI-H460 and A549 lung carcinoma cell lines resulted in the determination of IC_50_ values for compound **9j** as 31.4 ± 0.48 μM and 13.6 ± 3.34 μM, respectively (see Supporting Information File 1 for details).

## Conclusion

In summary, we have presented a practically convenient and streamlined protocol for the *trans*-diastereoselective introduction of an aryl substituent at position 4 of the 1,4-dihydroisoquinol-3-one (1,4-DHIQ) scaffold. The protocol relies on hitherto undescribed direct Regitz diazo transfer onto readily available 3(2*H*)-isoquinolones followed by TfOH-promoted arylation. The generally high-yielding two-step sequence was shown to be applicable to a wide range of substrates. To a varying degree, the arylation step was accompanied by the elimination of the nitrogen molecule and deprotonation to furnish 3-isoquinolone byproducts (formed exclusively in the absence of the arene). The extent of this side reaction was found to be dependent on the electronic character of the 1,4-DHIQs and the carbocation-intercepting arene molecule. Considering the pronounced three-dimensional character of 1,2,4-trisubstituted 1,4-DHIQ adducts synthesized in this work, they were deemed efficient probes for the perturbation of vital cellular targets. Screening of these compounds against lung carcinoma cancer cell lines confirmed high cytotoxicity of selected analogs, which validates this new chemotype for further investigation as anticancer cytotoxic agents.

## Supporting Information

Deposition Numbers 2158046 (for **9a**), 2170881 (for **10c**) and 2170877 (for **16**) contain the supplementary crystallographic data for this paper. These data are provided free of charge by the joint Cambridge Crystallographic Data Centre (http://www.ccdc.cam.ac.uk/structures) and Fachinformationszentrum Karlsruhe Access Structures service.

File 1 General experimental information, X-ray crystallographic data, synthetic procedures, analytical data and NMR spectra for the reported compounds.
