# Supplementary material for: Facile and diastereoselective arylation of the privileged 1,4-dihydroisoquinolin-3(2H)-one scaffold
Source: Beilstein J Org Chem. 2022 Aug 22;18:1070–8. doi: 10.3762/bjoc.18.109 (PMC9443417; doi:10.3762/bjoc.18.109)

BEILSTEIN JOURNAL OF ORGANIC CHEMISTRY

Supporting Information

for

Facile and diastereoselective arylation of the privileged 1,4-dihydroisoquinolin-3(2*H*)-one scaffold

Dmitry Dar'in, Grigory Kantin, Alexander Bunev and Mikhail Krasavin

Beilstein J. Org. Chem. **2022,** *18,* 1070–1078. doi:10.3762/bjoc.18.109

General experimental information, X-ray crystallographic data, synthetic procedures, analytical data and NMR spectra for the reported compounds

License and Terms: This is a supporting information file under the terms of the Creative Commons Attribution License (https://creativecommons.org/licenses/by/4.0). Please note that the reuse, redistribution and reproduction in particular requires that the author(s) and source are credited and that individual graphics may be subject to special legal provisions.
The license is subject to the *Beilstein Journal of Organic Chemistry* terms and conditions: (https://www.beilstein-journals.org/bjoc/terms)

Table of contents

1. Experimental procedures and characterization data S2
2. Crystallographic data for compounds **9a**, **10c** and **16** S27
3. Biological data S30
4. References..S32
5. Copies of ^{1}H, ^{13}C NMR, ^{19}F and NOESY NMR spectra S33

1. Experimental procedures and characterization data

General considerations. All reagents were used as purchased from commercial suppliers without further purification. Dichloromethane (DCM) and 1,2-dichloroethane (DCE) were freshly distilled over P_2O_5. NMR spectrum were recorded using Bruker Avance III spectrometer in $CDCl_3$ (^1H: 400.13 MHz; ^{13}C: 100.61 MHz; ^{19}F 376.50 MHz); chemical shifts are reported as parts per million (δ, ppm); the residual solvent peak ($CHCl_3$) was used as internal standard: 7.26 for ^1H and 77.16 ppm for ^{13}C; multiplicities are abbreviated as follows: s = singlet, d = doublet, t = triplet, q = quartet, m = multiplet, dd = doublet of doublets, dt = doublet of triplets, ddd = doublet/doublets of doublets; coupling constants, *J*, are reported in Hz. Mass spectra were recorded using Bruker microTOF spectrometer (ionization by electrospray, positive ion detection). Melting points were determined in open capillary tubes on Stuart SMP50 Automatic Melting Point Apparatus. Analytical thin-layer chromatography was carried out on UV-254 silica gel plates using appropriate eluents. Compounds were visualized with short-wavelength UV light. Column chromatography was performed using silica gel Merk grade 60 (0.040–0.063 mm) 230–400 mesh. Starting compounds **11** were synthesized according to slightly modified literature procedures.[1,2]

Preparation of 1,4-dihydroisoquinolin-3(2*H*)-ones 11 and compound 14

Method A:

To a solution of the corresponding imine (5.0 mmol) in DCM (50 mL) was added arylacetyl chloride (5.0 mmol). After stirring at ambient temperature for 2.5 h, triflic acid (20 mL) was added and stirring was continued for 4 h. The resulting mixture was poured onto crushed ice, extracted with DCM (3 × 20 mL), washed with water and brine, and dried over $MgSO_4$. After removal of the solvent under reduced pressure, the crude product was purified flash chromatography on silica gel (DCM/acetone, 0–30% of acetone).

Method B:

To a solution of the corresponding imine (4.0 mmol) in DCE (20 mL) was added arylacetyl chloride (4.0 mmol). After stirring for 20 min, $AlCl_3$ (5.0 mmol) was added and stirring was continued at 80 °C for 1.5 h. Upon cooling to ambient temperature, 10% HCl (30 mL) was added and the mixture was extracted with DCM (3 × 15 mL), washed with brine, and evaporated. The crude material was subjected to flash chromatography on silica gel (DCM/acetone, 0–30% of acetone).

1,2-Diphenyl-1,4-dihydroisoquinolin-3(2*H*)-one[2] **(11a):** prepared from 2-phenylacetyl chloride and *N*-benzylidene aniline according to both Method A and Method B (scale – 5 mmol). Yield: 1.14 g (76%) for Method A and 1.23 g (82%) for Method B. White solid; m.p. 145.0–146.1 °C. ^{1}H NMR (400 MHz, $CDCl_3$) δ 7.60 – 7.10 (m, 14H), 5.94 (s, 1H), 3.94 (d, *J* = 19.3 Hz, 1H), 3.83 (d, *J* = 19.2 Hz, 1H).

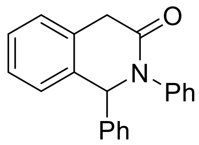

2-Methyl-1-phenyl-1,4-dihydroisoquinolin-3(2*H*)-one[2] **(11b):** prepared from 2-phenylacetyl chloride and *N*-benzylidene methylamine according to both Method A and Method B (scale – 5 mmol). Yield: 819 mg (69%) for Method A and 771 mg (65%) for Method B. White solid; m.p. 96.2–97.7 °C. ^{1}H NMR (400 MHz, $CDCl_3$) δ 7.44 – 7.03 (m, 9H), 5.49 (s, 1H), 3.84 (d, *J* = 20.0 Hz, 1H), 3.73 (d, *J* = 20.0 Hz, 1H), 3.07 (s, 3H).

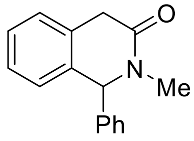

7-Chloro-1,2-diphenyl-1,4-dihydroisoquinolin-3(2*H*)-one (11c): prepared from 2-(4-chlorophenyl)acetyl chloride and *N*-benzylidene aniline according to Method A (scale – 5 mmol). Yield: 1.1 g (66%). White solid; m.p. 146.2–148.0 °C. ^{1}H NMR (400 MHz, $CDCl_3$) δ 7.41 – 7.27 (m, 8H), 7.27 – 7.15 (m, 5H), 5.88 (s, 1H), 3.90 (d, *J* = 19.3 Hz, 1H), 3.80 (d, *J* = 19.3 Hz, 1H). $^{13}C\{^1H\}$ NMR (101 MHz, $CDCl_3$) δ 168.8, 141.7, 139.6, 137.5, 132.8, 130.1, 129.23, 129.19, 129.0, 128.1, 127.2, 126.62, 126.56, 126.2, 68.7, 37.8. HRMS (ESI), *m/z* calcd for $C_{21}H_{17}ClNO$ $[M+H]^+$ 334.0993/336.0971 found 334.0999/336.0972.

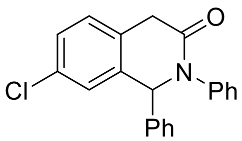

5-Chloro-1,2-diphenyl-1,4-dihydroisoquinolin-3(2*H*)-one (11d): prepared from 2-(2-chlorophenyl)acetyl chloride and *N*-benzylidene aniline according to Method B (scale – 5 mmol). Yield: 1.32 g (79%). White solid; m.p. 182.6–184.0 °C. ^{1}H NMR (400 MHz, $CDCl_3$) δ 7.41 – 7.27 (m, 7H), 7.27 – 7.15 (m, 6H), 5.93 (s, 1H), 4.21 (d, *J* = 20.3 Hz, 1H), 3.81 (d, *J* = 20.3 Hz, 1H). $^{13}C\{^1H\}$ NMR (101 MHz, $CDCl_3$) δ 168.2, 141.5, 140.3, 137.5, 133.2, 129.6, 129.2, 129.0, 128.4, 128.1, 128.0, 127.2, 126.7, 126.6, 124.7, 69.1, 35.4. HRMS (ESI), *m/z* calcd for $C_{21}H_{16}ClNNaO$ $[M+Na]^+$ 356.0813/358.0791 found 356.0818/358.0794.

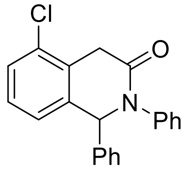

7-Methoxy-1,2-diphenyl-1,4-dihydroisoquinolin-3(2*H*)-one (11e): prepared from 2-(4-methoxyphenyl)acetyl chloride and *N*-benzylidene aniline according to Method A (scale – 5 mmol). Yield: 1.3 g (79%). White solid; m.p. 129.2–130.8 °C. ^{1}H NMR (400 MHz, $CDCl_3$) δ 7.41 – 7.19 (m, 10H), 7.17 (d, *J* = 8.2 Hz, 1H), 6.91 – 6.82 (m, 2H), 5.86 (s, 1H), 3.87 (d, *J* = 19.3 Hz, 1H), 3.82 (s, 3H), 3.77 (d, *J* = 19.3 Hz, 1H). $^{13}C\{^1H\}$ NMR (101 MHz, $CDCl_3$) δ 169.6, 158.6, 142.0, 140.3, 136.9, 129.1, 128.9, 128.8, 127.8, 127.0, 126.7, 126.6,

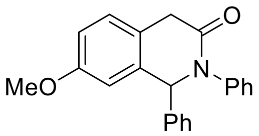

123.6, 113.4, 111.8, 69.2, 55.4, 37.5. HRMS (ESI), *m/z* calcd for $C_{22}H_{20}NO_2$ $[M+H]^+$ 330.1489 found 330.1489.

5-Methoxy-1,2-diphenyl-1,4-dihydroisoquinolin-3(2*H*)-one (11f): prepared from 2-(2-methoxyphenyl)acetyl chloride and *N*-benzylidene aniline according to Method B (scale – 4 mmol). Yield: 742 mg (56%). White solid; m.p. 186.9–188.8 °C. ^{1}H NMR (400 MHz, $CDCl_3$) δ 7.38 – 7.19 (m, 9H), 7.19 – 7.11 (m, 2H), 6.88 (d, *J* = 7.7 Hz, 1H), 6.83 (d, *J* = 8.2 Hz, 1H), 5.89 (s, 1H), 4.13 (d, *J* = 20.6 Hz, 1H), 3.89 (s, 3H), 3.65 (d, *J* = 20.6 Hz, 1H). ^{13}C{^{1}H} NMR (101 MHz, $CDCl_3$) δ 169.2, 156.3, 141.9, 141.0, 136.7, 129.1, 128.8, 127.8, 127.7, 127.0, 126.9, 126.7, 120.0, 118.2, 108.8, 69.2, 55.5, 31.8. HRMS (ESI), *m/z* calcd for $C_{22}H_{20}NO_2$ $[M+H]^+$ 330.1489 found 330.1490.

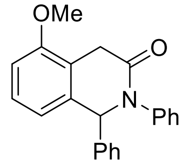

6,7-Dimethoxy-1,2-diphenyl-1,4-dihydroisoquinolin-3(2*H*)-one[2] (11g): prepared from 2-(3,4-dimethoxyphenyl)acetyl chloride and *N*-benzylidene aniline according to Method B (scale – 4 mmol). Yield: 1.24 g (86%). Pale beige amorphous solid. ^{1}H NMR (400 MHz, $CDCl_3$) δ 7.40 – 7.23 (m, 6H), 7.21 – 7.13 (m, 4H), 6.74 (s, 1H), 6.73 (s, 1H), 5.82 (s, 1H), 3.93 (d, *J* = 19.6 Hz, 1H), 3.92 (s, 3H), 3.86 (s, 3H), 3.76 (d, *J* = 19.6 Hz, 1H).

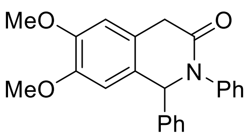

6-Chloro-2-methyl-1-phenyl-1,4-dihydroisoquinolin-3(2*H*)-one (11h): prepared from 2-(3-chlorophenyl)acetyl chloride and *N*-benzylidene methylamine according to Method B (scale – 6 mmol) as mixture with 8-chloro regioisomer (ratio 10:1). Yield: 933 mg (58%); pure compound **11h** was obtained after crystallization from *n*-Hexane–Ether mixture. White solid; m.p. 132.5–133.6 °C. ^{1}H NMR (400 MHz, $CDCl_3$) δ 7.43 – 7.29 (m, 3H), 7.26 – 7.10 (m, 5H), 5.46 (s, 1H), 3.81 (d, *J* = 20.2 Hz, 1H), 3.70 (d, *J* = 20.2 Hz, 1H), 3.05 (s, 3H). ^{13}C{^{1}H} NMR (101 MHz, $CDCl_3$) δ 168.0, 140.3, 133.4, 133.2, 132.8, 129.2, 128.2, 127.8, 127.5, 127.1, 126.4, 67.3, 36.1, 33.5. HRMS (ESI), *m/z* calcd for $C_{16}H_{15}ClNO$ $[M+H]^+$ 272.0837/274.0812 found 272.0843/274.0816.

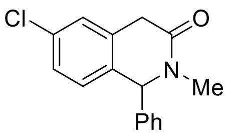

2-(4-Methoxyphenyl)-7-methyl-1-(4-(trifluoromethyl)phenyl)-1,4-dihydroisoquinolin-3(2*H*)-one (11i): prepared from 2-(4-methylphenyl)acetyl chloride and *N*-(4-(trifluoromethyl)benzylidene)4-methoxyaniline according to Method A (scale – 5 mmol). Yield: 1.11 g (54%). Pale beige amorphous solid. ^{1}H NMR (400 MHz, $CDCl_3$) δ 7.58 (d, *J* = 8.1 Hz, 1H), 7.35 (d, *J* = 8.1 Hz, 1H), 7.16 (s, 2H), 7.11 – 7.04 (m, 3H), 6.89 (d, *J* = 9.0 Hz, 2H), 5.87 (s, 1H), 3.91 (d, *J* = 19.6 Hz, 1H), 3.82 (d, *J* = 19.6 Hz, 1H), 3.81 (s, 3H), 2.37 (s, 3H). ^{13}C{^{1}H} NMR (101 MHz, $CDCl_3$) δ 169.3, 158.5, 144.8,

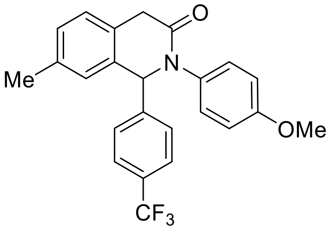

136.9, 134.6, 134.4, 130.1 (q, $^{2}J_{C–F}$ = 32.5 Hz), 129.1, 128.4, 128.0, 127.9, 127.1, 126.6, 125.9 (q, $^{3}J_{C–F}$ = 3.7 Hz), 123.9 (q, $^{1}J_{C–F}$ = 272.1 Hz), 114.6, 69.2, 55.4, 37.5, 21.1. ^{19}F{^{1}H} NMR (377 MHz, $CDCl_3$) δ –62.58. HRMS (ESI), *m/z* calcd for $C_{24}H_{21}F_3NO_2$ $[M+H]^+$ 412.1519 found 412.1521.

7-Methoxy-2-methyl-1-(3-nitrophenyl)-1,4-dihydroisoquinolin-3(2*H*)-one (11j): prepared from 2-(4-methoxyphenyl)acetyl chloride and *N*-(3-nitrobenzylidene) methylamine according to Method B (scale – 4 mmol). Yield: 1.01 g (81%). White solid; m.p. 154.8–156.2 °C. ^{1}H NMR (400 MHz, $CDCl_3$) δ 8.20 – 8.12 (m, 2H), 7.60 – 7.52 (m, 2H), 7.12 (d, *J* = 8.4 Hz, 1H), 6.85 (dd, *J* = 8.5, 2.6 Hz, 1H), 6.72 (d, *J* = 2.6 Hz, 1H), 5.54 (s, 1H), 3.78 (s, 3H), 3.78 (d, *J* = 20.2 Hz, 1H), 3.71 (d, *J* = 20.2 Hz, 1H), 3.07 (s, 3H). ^{13}C{^{1}H} NMR (101 MHz, $CDCl_3$) δ 168.9, 158.6, 148.7, 143.1, 134.3, 132.3, 130.3, 129.2, 123.2, 122.8, 121.7, 114.0, 111.7, 67.2, 55.4, 35.6, 33.6. HRMS (ESI), *m/z* calcd for $C_{17}H_{16}N_2NaO_4$ $[M+Na]^+$ 335.1002 found 335.1008.

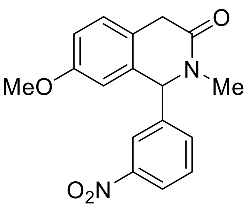

7-Chloro-2-(4-chlorobenzyl)-1-(4-fluorophenyl)-1,4-dihydroisoquinolin-3(2*H*)-one (11k): prepared from 2-(4-chlorophenyl)acetyl chloride and *N*-(4-fluorobenzylidene) 4-chlorobenzylamine according to Method A (scale – 5 mmol). Yield: 1.84 g (92%). White solid; m.p. 115.4–117.3 °C. ^{1}H NMR (400 MHz, $CDCl_3$) δ 7.30 (d, *J* = 8.6 Hz, 2H), 7.23 (dd, *J* = 8.2, 2.1 Hz, 1H), 7.21 – 7.11 (m, 5H), 7.10 – 7.01 (m, 3H), 5.62 (d, *J* = 15.1 Hz, 1H), 5.33 (s, 1H), 3.88 (d, *J* = 20.1 Hz, 1H), 3.80 (d, *J* = 20.1 Hz, 1H), 3.69 (d, *J* = 15.1 Hz, 1H). ^{13}C{^{1}H} NMR (101 MHz, $CDCl_3$) δ 168.4, 162.5 (d, $^{1}J_{C–F}$ = 248.1 Hz), 136.1, 135.6 (d, $^{4}J_{C–F}$ = 3.4 Hz), 134.7, 133.6, 132.8, 129.4, 129.2, 129.0, 128.3 (d, $^{3}J_{C–F}$ = 8.4 Hz), 128.1, 126.3, 116.3 (d, $^{2}J_{C–F}$ = 21.7 Hz), 63.0, 47.0, 36.1. ^{19}F{^{1}H} NMR (377 MHz, $CDCl_3$) δ –113.15. HRMS (ESI), *m/z* calcd for $C_{22}H_{17}Cl_2FNO$ $[M+H]^+$ 400.0666/402.0641 found 400.0667/402.0641.

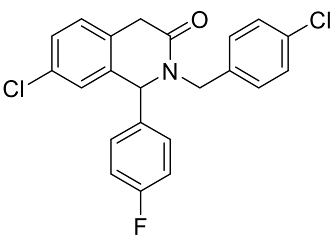

7-Chloro-1-(2-chlorophenyl)-2-methyl-1,4-dihydroisoquinolin-3(2*H*)-one (11l): prepared from 2-(4-chlorophenyl)acetyl chloride and *N*-(2-chlorobenzylidene) methylamine according to Method A (scale – 2.5 mmol). Yield: 655 mg (85%). White solid; m.p. 167.8–169.2 °C. ^{1}H NMR (400 MHz, $CDCl_3$) δ 7.50 – 7.40 (m, 1H), 7.31 – 7.19 (m, 4H), 7.15 – 7.07 (m, 2H), 6.16 (s, 1H), 3.94 (d, *J* = 21.1 Hz, 1H), 3.79 (d, *J* = 21.1 Hz, 1H), 2.94 (s, 3H). ^{13}C{^{1}H} NMR (101 MHz, $CDCl_3$) δ 167.6, 138.7, 135.1, 132.71, 132.69, 130.4, 129.6, 129.2, 128.8, 128.7, 128.12, 128.09, 126.2, 62.9, 35.1, 32.8. HRMS (ESI), *m/z* calcd for $C_{16}H_{14}Cl_2NO$ $[M+H]^+$ 306.0447/308.0420 found 306.0447/308.0418.

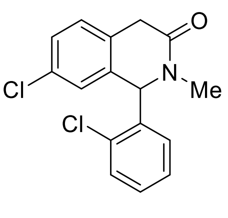

7-Chloro-2-(4-fluorophenyl)-1-(*p*-tolyl)-1,4-dihydroisoquinolin-3(2*H*)-one (11m): prepared from 2-(4-chlorophenyl)acetyl chloride and *N*-(4-methylbenzylidene) 4-fluoroaniline according to Method B (scale – 4 mmol). Yield: 875 mg (60%). Pale tan amorphous solid. ^{1}H NMR (400 MHz, $CDCl_3$) δ 7.31 – 7.26 (m, 2H), 7.19 (d, *J* = 8.0 Hz, 1H), 7.17 – 7.10 (m, 4H), 7.10 – 7.01 (m, 4H), 5.77 (s, 1H), 3.93 (d, *J* = 19.6 Hz, 1H), 3.80 (d, *J* = 19.6 Hz, 1H), 2.34 (s, 3H). ^{13}C{^{1}H} NMR (101 MHz, $CDCl_3$) δ 168.8, 161.3 (d, $^{1}J_{C\text{–}F}$ = 246.9 Hz), 138.2, 137.5 (d, $^{4}J_{C\text{–}F}$ = 3.1 Hz), 137.3, 136.7, 132.8, 129.8, 129.7, 129.2, 128.6 (d, $^{3}J_{C\text{–}F}$ = 8.5 Hz), 128.1, 126.5, 126.2, 116.1 (d, $^{2}J_{C\text{–}F}$ = 22.7 Hz), 68.8, 37.4, 21.0. ^{19}F{^{1}H} NMR (377 MHz, $CDCl_3$) δ –114.34. HRMS (ESI), *m/z* calcd for $C_{22}H_{18}ClFNO$ $[M+H]^+$ 366.1056/368.1034 found 366.1055/368.1031.

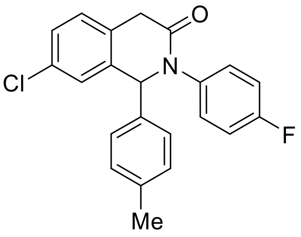

7-Chloro-1-(4-nitrophenyl)-2-propyl-1,4-dihydroisoquinolin-3(2*H*)-one (11n): prepared from 2-(4-chlorophenyl)acetyl chloride and *N*-(4-nitrobenzylidene) *n*-propylamine according to Method B (scale – 4 mmol). Yield: 1.04 g (75%). Light yellow amorphous solid. ^{1}H NMR (400 MHz, $CDCl_3$) δ 8.23 (d, *J* = 8.8 Hz, 2H), 7.44 (d, *J* = 8.7 Hz, 2H), 7.34 – 7.21 (m, 2H), 7.13 (d, *J* = 8.1 Hz, 1H), 5.61 (s, 1H), 4.08 (ddd, *J* = 13.6, 9.2, 6.4 Hz, 1H), 3.72 (d, *J* = 19.8 Hz, 1H), 3.65 (d, *J* = 19.8 Hz, 1H), 2.84 (ddd, *J* = 14.0, 9.1, 5.5 Hz, 1H), 1.72 – 1.50 (m, 2H), 0.90 (t, *J* = 7.4 Hz, 3H). ^{13}C{^{1}H} NMR (101 MHz, $CDCl_3$) δ 168.4, 147.7, 147.1, 135.5, 132.9, 130.0, 129.4, 128.5, 127.2, 126.1, 124.5, 64.5, 48.0, 36.6, 20.8, 11.3. HRMS (ESI), *m/z* calcd for $C_{18}H_{18}ClN_2O_3$ $[M+H]^+$ 345.1001/347.0978 found 345.1005/347.0979.

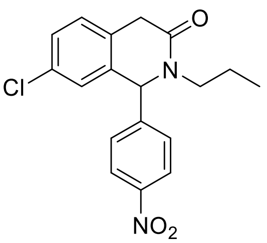

1-(2-Nitrophenyl)-2-(*p*-tolyl)-1,4-dihydroisoquinolin-3(2*H*)-one (11o): prepared from 2-phenylacetyl chloride and *N*-benzylidene aniline according to both Method A (scale – 2.5 mmol) and Method B (scale – 6 mmol). Yield: 308 mg (34%) for Method A and 1.56 g (72%) for Method B. Pale beige amorphous solid. ^{1}H NMR (400 MHz, $CDCl_3$) δ 7.70 (dd, *J* = 8.2, 1.4 Hz, 1H), 7.57 (td, *J* = 7.6, 1.4 Hz, 1H), 7.46 (dd, *J* = 7.9, 1.4 Hz, 1H), 7.42 – 7.23 (m, 6H), 7.11 (d, *J* = 8.1 Hz, 2H), 6.92 (s, 1H), 6.85 (d, *J* = 8.3 Hz, 2H), 4.16 (d, *J* = 20.8 Hz, 1H), 3.97 (d, *J* = 20.8 Hz, 1H), 2.33 (s, 3H). ^{13}C{^{1}H} NMR (101 MHz, $CDCl_3$) δ 168.2, 149.2, 137.8, 137.7, 136.3, 133.32, 133.28, 130.9, 130.1, 129.6, 128.8, 128.3, 128.0, 127.4, 127.0, 126.6, 124.4, 62.4, 36.8, 21.1. HRMS (ESI), *m/z* calcd for $C_{22}H_{19}N_2O_3$ $[M+H]^+$ 359.1390 found 359.1391.

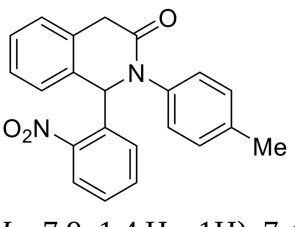

1,2-Bis(3,4-dimethoxyphenyl)-1,4-dihydroisoquinolin-3(2*H*)-one (11p): prepared from 2-phenylacetyl chloride and *N*-(3,4-dimethoxybenzylidene) 3,4-dimethoxyaniline according to Method B (scale – 4 mmol). Yield: 772 mg (46%). Yellowish amorphous solid. ^{1}H NMR (400 MHz, $CDCl_3$) δ 7.34 – 7.24 (m, 4H), 6.84 (d, *J* = 8.4 Hz, 1H), 6.78 (d, *J* = 8.3 Hz, 1H), 6.75 – 6.67 (m, 3H), 6.66 (d, *J* = 2.1 Hz, 1H), 5.79 (s, 1H), 3.99 (d, *J* = 19.6 Hz, 1H), 3.88 (s, 3H), 3.85 (s, 3H), 3.85 (d, *J* = 19.5 Hz, 1H), 3.77 (s, 3H), 3.76 (s, 3H). $^{13}C\{^{1}H\}$ NMR (101 MHz, $CDCl_3$) δ 169.2, 149.2, 149.1, 148.7, 148.1, 135.6, 134.8, 133.3, 131.32, 127.9, 127.8, 127.0, 126.1, 119.2, 119.1, 111.2, 111.1, 111.0, 110.3, 69.4, 56.0, 55.91, 55.90, 37.9. HRMS (ESI), *m/z* calcd for $C_{25}H_{26}NO_5$ $[M+H]^+$ 420.1806 found 420.1811.

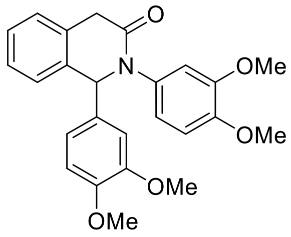

1-(2-Methoxyphenyl)-2-(4-methoxyphenyl)-1,4-dihydroisoquinolin-3(2*H*)-one (11q): prepared from 2-phenylacetyl chloride and *N*-(2-methoxybenzylidene) 4-methoxyaniline according to Method B (scale – 2.5 mmol). Yield: 705 mg (78%). White amorphous solid. ^{1}H NMR (400 MHz, $CDCl_3$) δ 7.40 – 7.34 (m, 1H), 7.31 – 7.17 (m, 5H), 7.02 (d, *J* = 8.9 Hz, 2H), 6.92 (td, *J* = 7.5, 1.1 Hz, 1H), 6.88 – 6.80 (m, 3H), 6.24 (s, 1H), 4.13 (d, *J* = 20.0 Hz, 1H), 3.89 (d, *J* = 20.0 Hz, 1H), 3.78 (s, 3H), 3.74 (s, 3H). $^{13}C\{^{1}H\}$ NMR (101 MHz, $CDCl_3$) δ 169.7, 158.3, 156.6, 134.9, 134.6, 131.4, 129.4, 129.2, 128.3, 128.0, 127.4, 126.5, 126.4, 120.8, 114.3, 111.4, 64.7, 55.37, 55.35, 37.5. HRMS (ESI), *m/z* calcd for $C_{23}H_{22}NO_3$ $[M+H]^+$ 360.1594 found 360.1597.

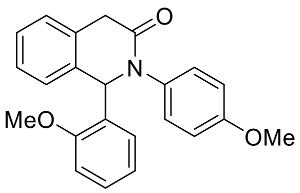

1-(Naphthalen-1-yl)-2-phenyl-1,4-dihydroisoquinolin-3(2*H*)-one (11r): prepared from 2-phenylacetyl chloride and *N*-naphthalen-1-ylidene aniline according to Method B (scale – 4 mmol). Yield: 1.05 g (71%); purity 90%. Pale beige amorphous solid. This substance was used in diazo transfer step without additional purification. ^{1}H NMR (400 MHz, $CDCl_3$) δ 7.92 (d, *J* = 8.5 Hz, 1H), 7.84 (dd, *J* = 8.1, 1.5 Hz, 1H), 7.77 (dd, *J* = 7.9, 1.7 Hz, 1H), 7.48 – 7.37 (m, 4H), 7.35 – 7.27 (m, 3H), 7.21 – 7.11 (m, 4H), 7.04 – 6.96 (m, 2H), 6.78 (s, 1H), 4.33 (d, *J* = 20.5 Hz, 1H), 4.07 (d, *J* = 20.4 Hz, 1H).

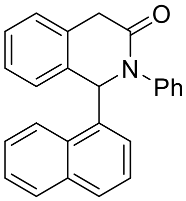

5,8,9,13b-Tetrahydro-6*H*-isoquinolino[1,2-*a*]isoquinolin-6-one (11s): prepared from 2-phenylacetyl chloride and 3,4-dihydroisoquinoline according to Method B (scale – 5 mmol). Yield: 806 mg (65%). Beige amorphous solid. ^{1}H NMR (400 MHz, $CDCl_3$) δ 7.38 – 7.20 (m, 6H), 7.14 – 7.06 (m, 1H), 6.95 (d, *J* = 7.5 Hz, 1H), 5.72 (s, 1H), 4.71 (ddd, *J* = 12.8, 5.7, 3.8 Hz, 1H), 3.70 (d, *J* = 18.3 Hz, 1H), 3.63 (d, *J* = 18.4 Hz, 1H), 3.21 (ddd, *J* = 12.6,

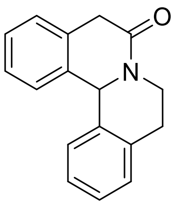

10.1, 4.9 Hz, 1H), 2.96 (ddd, *J* = 15.9, 10.2, 5.7 Hz, 1H), 2.87 (dt, *J* = 15.9, 4.4 Hz, 1H). $^{13}C\{^1H\}$ NMR (101 MHz, $CDCl_3$) δ 169.9, 136.3, 135.0, 134.1, 133.6, 129.0, 127.9, 127.7, 127.4, 127.0, 126.4, 126.2, 125.6, 59.2, 40.7, 38.6, 28.5. HRMS (ESI), *m/z* calcd for $C_{17}H_{16}NO$ $[M+H]^+$ 250.1226 found 250.1231.

***N*-(3-Oxo-1,3-dihydroisobenzofuran-1-yl)-*N*,2-diphenylacetamide (14):** prepared from 2-phenylacetyl chloride and methyl 2-((phenylimino)methyl)benzoate according to Method B (scale – 4 mmol). Yield: 1.13 g (82%). White solid; m.p. 162.0–162.7 °C. ^1H NMR (400 MHz, $CDCl_3$) δ 8.04 (s, 1H), 7.67 (dd, *J* = 7.7, 1.0 Hz, 1H), 7.63 (td, *J* = 7.5, 1.1 Hz, 1H), 7.53 (d, *J* = 7.7 Hz, 1H), 7.44 (t, *J* = 7.5 Hz, 1H), 7.32 – 7.10 (m, 7H), 7.10 – 7.04 (m, 2H), 6.72 (br.s, 1H), 3.53 (s, 2H). $^{13}C\{^1H\}$ NMR (101 MHz, $CDCl_3$) δ 172.6, 168.6, 144.8, 135.7, 134.1, 134.0, 130.4, 130.1, 129.6, 129.1, 129.0, 128.5, 127.5, 127.0, 125.4, 123.4, 83.4, 41.7. HRMS (ESI), *m/z* calcd for $C_{22}H_{17}NNaO_3$ $[M+Na]^+$ 366.1101 found 366.1107.

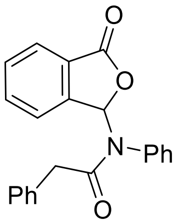

Preparation of Diazo isoquinolin-3-ones 10. General procedure (GP1):

To a stirring mixture of compound **11** (2.0 mmol) and 4-acetamidobenzenesulfonyl azide (2.2 mmol) in DCM (8 mL) was added DBU (2.1 mmol) and the resulting solution was stirred at room temperature for 2–5 days (controlled by TLC). The reaction mixture was subjected to flash chromatography on silica gel (10 g) eluting with DCM to afford pure diazo compound **10**.

4-Diazo-1,2-diphenyl-1,4-dihydroisoquinolin-3(2*H*)-one (10a): prepared according to the general procedure GP1 from compound **11a** (scale – 2 mmol). Yield: 612 mg (94%). Light orange solid; m.p. 142.6–144.3 °C. ^1H NMR (400 MHz, $CDCl_3$) δ 7.39 – 7.20 (m, 9H), 7.18 – 7.07 (m, 4H), 7.02 (d, *J* = 7.8 Hz, 1H), 5.91 (s, 1H). $^{13}C\{^1H\}$ NMR (101 MHz, $CDCl_3$) δ 162.5, 142.0, 141.2, 130.2, 129.1, 128.9, 128.5, 128.1, 127.5, 127.22, 127.18, 126.7, 125.3, 122.5, 119.4, 69.0, 65.2 ($C=N_2$). HRMS (ESI), *m/z* calcd for $C_{21}H_{16}N_3O$ $[M+H]^+$ 326.1288 found 326.1289.

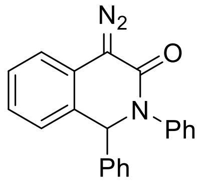

4-Diazo-2-methyl-1-phenyl-1,4-dihydroisoquinolin-3(2*H*)-one (10b): prepared according to the general procedure GP1 from compound **11b** (scale – 2.5 mmol). Yield: 434 mg (66%). Light orange solid; m.p. 96.6–97.9 °C. ^1H NMR (400 MHz, $CDCl_3$) δ 7.39 – 7.22 (m, 6H), 7.10 – 6.99 (m, 2H), 6.92 (d, *J* = 7.6 Hz, 1H), 5.53 (s, 1H), 2.99 (s, 3H). $^{13}C\{^1H\}$ NMR (101 MHz, $CDCl_3$) δ 162.1, 141.6, 129.5, 129.2, 128.3, 127.4, 126.7, 125.1, 122.1, 119.3, 67.7, 64.2 ($C=N_2$), 33.4. HRMS (ESI), *m/z* calcd for $C_{16}H_{13}N_3NaO$ $[M+Na]^+$ 286.0951 found 286.0950.

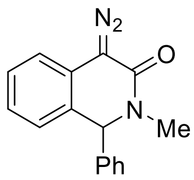

7-Chloro-4-diazo-1,2-diphenyl-1,4-dihydroisoquinolin-3(2*H*)-one (10c): prepared according to the general procedure GP1 from compound **11c** (scale – 1.5 mmol). Yield: 504 mg (93%). Orange solid; m.p. 160.4–162.1 °C. ^{1}H NMR (400 MHz, $CDCl_3$) δ 7.38 – 7.24 (m, 7H), 7.27 – 7.19 (m, 2H), 7.13 (d, *J* = 2.1 Hz, 1H), 7.09 (d, *J* = 7.6 Hz, 2H), 6.94 (d, *J* = 8.4 Hz, 1H), 5.85 (s, 1H). ^{13}C{^{1}H} NMR (101 MHz, $CDCl_3$) δ 162.0, 141.3, 140.9, 131.6, 130.7, 129.2, 129.1, 128.7, 128.4, 127.5, 127.4, 127.3, 126.7, 121.3, 120.6, 68.6, 65.1 (C=N_2). HRMS (ESI), *m/z* calcd for $C_{21}H_{14}ClN_3NaO$ [M+Na]$^+$ 382.0718/384.0696 found 382.0719/384.0694.

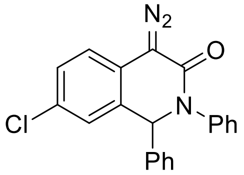

5-Chloro-4-diazo-1,2-diphenyl-1,4-dihydroisoquinolin-3(2*H*)-one (10d): prepared according to the general procedure GP1 from compound **11d** (scale – 2.0 mmol). Yield: 690 mg (96%). Orange solid; m.p. 158.1–159.7 °C. ^{1}H NMR (400 MHz, $CDCl_3$) δ 7.40 – 7.22 (m, 9H), 7.20 – 7.11 (m, 3H), 7.07 (t, *J* = 7.8 Hz, 1H), 5.85 (s, 1H). ^{13}C{^{1}H} NMR (101 MHz, $CDCl_3$) δ 163.7, 141.4, 141.0, 132.9, 130.0, 129.1, 129.0, 128.2, 126.8, 126.4, 126.3, 126.1, 125.6, 121.0, 68.1, 64.7 (C=N_2). HRMS (ESI), *m/z* calcd for $C_{21}H_{14}ClN_3NaO$ [M+Na]$^+$ 382.0718/384.0696 found 382.0724/384.0701.

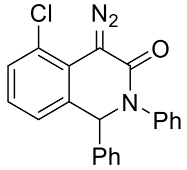

4-Diazo-7-methoxy-1,2-diphenyl-1,4-dihydroisoquinolin-3(2*H*)-one (10e): prepared according to the general procedure GP1 from compound **11e** (scale – 1.5 mmol). Yield: 432 mg (81%). Orange solid; m.p. 148.3–149.7 °C. ^{1}H NMR (400 MHz, $CDCl_3$) δ 7.37 – 7.19 (m, 8H), 7.15 – 7.06 (m, 2H), 6.98 – 6.89 (m, 2H), 6.71 (s, 1H), 5.85 (s, 1H), 3.77 (s, 3H). ^{13}C{^{1}H} NMR (101 MHz, $CDCl_3$) δ 163.0, 157.6, 141.8, 141.2, 131.5, 129.0, 128.9, 128.1, 127.6, 127.1, 126.7, 120.7, 114.5, 114.0, 113.2, 69.1, 64.5 (C=N_2), 55.5. HRMS (ESI), *m/z* calcd for $C_{22}H_{18}N_3O_2$ [M+H]$^+$ 356.1394 found 356.1399.

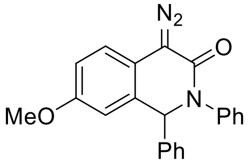

4-Diazo-5-methoxy-1,2-diphenyl-1,4-dihydroisoquinolin-3(2*H*)-one (10f): prepared according to the general procedure GP1 from compound **11f** (scale – 1.2 mmol). Yield: 312 mg (73%). Orange solid; m.p. 170.3–171.9 °C. ^{1}H NMR (400 MHz, $CDCl_3$) δ 7.36 – 7.19 (m, 8H), 7.17 – 7.10 (m, 2H), 7.07 (t, *J* = 8.0 Hz, 1H), 6.82 (d, *J* = 7.5 Hz, 1H), 6.79 (d, *J* = 8.3 Hz, 1H), 5.85 (s, 1H), 3.88 (s, 3H). ^{13}C{^{1}H} NMR (101 MHz, $CDCl_3$) δ 164.2, 152.8, 141.9, 141.7, 131.1, 129.0, 128.9, 128.0, 127.0, 126.8, 126.5, 125.9, 119.6, 111.6, 109.6, 68.5, 62.7 (C=N_2), 55.7. HRMS (ESI), *m/z* calcd for $C_{22}H_{20}N_3O_2$ [M+H]$^+$ 356.1394 found 356.1396.

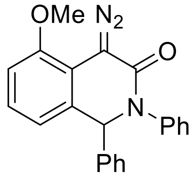

4-Diazo-6,7-dimethoxy-1,2-diphenyl-1,4-dihydroisoquinolin-3(2*H*)-one (10g): prepared according to

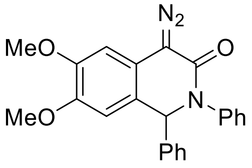

the general procedure GP1 from compound **11g** (scale – 1.5 mmol). Yield: 393 mg (68%). Red-orange solid; m.p. 159.3–160.6 °C. ^{1}H NMR (400 MHz, $CDCl_3$) δ 7.35 – 7.20 (m, 6H), 7.23 – 7.15 (m, 2H), 7.11 – 7.02 (m, 2H), 6.60 (s, 1H), 6.45 (s, 1H), 5.82 (s, 1H), 3.92 (s, 3H), 3.79 (s, 3H). $^{13}C\{^1H\}$ NMR (101 MHz, $CDCl_3$) δ 162.6, 149.7, 147.4, 142.0, 141.1, 129.0, 128.9, 128.1, 127.8, 127.2, 126.8, 122.4, 113.9, 110.8, 102.4, 69.0, 64.8 (C=N_2), 56.2, 56.1. HRMS (ESI), *m/z* calcd for $C_{23}H_{19}N_3NaO_3$ $[M+Na]^+$ 408.1319 found 408.1327.

6-Chloro-4-diazo-2-methyl-1-phenyl-1,4-dihydroisoquinolin-3(2*H*)-one (10h): prepared according

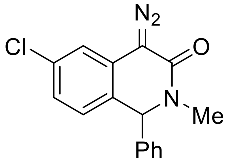

to the general procedure GP1 from compound **11h** (scale – 1.8 mmol). Yield: 520 mg (97%). Orange solid; m.p. 130.8–132.3 °C. ^{1}H NMR (400 MHz, $CDCl_3$) δ 7.41 – 7.23 (m, 5H), 7.03 – 6.92 (m, 2H), 6.89 (d, *J* = 1.9 Hz, 1H), 5.49 (s, 1H), 2.97 (s, 3H). $^{13}C\{^1H\}$ NMR (101 MHz, $CDCl_3$) δ 161.3, 141.2, 134.2, 129.3, 128.7, 128.5, 127.8, 126.6, 125.2, 124.4, 118.9, 67.2, 64.2 (C=N_2), 33.3 HRMS (ESI), *m/z* calcd for $C_{16}H_{13}ClN_3O$ $[M+H]^+$ 298.0742/300.0717 found 298.0741/300.0719.

4-Diazo-2-(4-methoxyphenyl)-7-methyl-1-(4-(trifluoromethyl)phenyl)-1,4-dihydroisoquinolin-

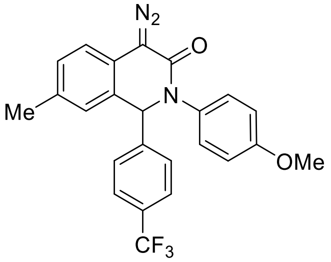

3(2*H*)-one (10i): prepared according to the general procedure GP1 from compound **11i** (scale – 1.5 mmol). Yield: 623 mg (95%). Orange solid; m.p. 165.4–166.5 °C. ^{1}H NMR (400 MHz, $CDCl_3$) δ 7.55 (d, *J* = 8.1 Hz, 2H), 7.34 (d, *J* = 8.1 Hz, 2H), 7.18 (dd, *J* = 8.0, 1.9 Hz, 1H), 6.97 (d, *J* = 8.9 Hz, 2H), 6.92 (d, *J* = 8.0 Hz, 1H), 6.90 (d, *J* = 1.7 Hz, 1H), 6.84 (d, *J* = 8.9 Hz, 2H), 5.87 (s, 1H), 3.80 (s, 3H), 2.30 (s, 3H). $^{13}C\{^1H\}$ NMR (101 MHz, $CDCl_3$) δ 162.5, 158.6, 146.0, 135.3, 133.6, 130.3 (q, $^2J_{C-F}$ = 32.6 Hz), 129.7, 129.1, 128.9, 127.8, 127.3, 126.0 (q, $^3J_{C-F}$ = 3.8 Hz), 123.9 (q, $^1J_{C-F}$ = 272.3 Hz), 119.5, 119.4, 114.5, 69.0, 64.7 (C=N_2), 55.4, 21.0. $^{19}F\{^1H\}$ NMR (377 MHz, $CDCl_3$) δ –62.57. HRMS (ESI), *m/z* calcd for $C_{24}H_{19}F_3N_3O_2$ $[M+H]^+$ 438.1424 found 438.1427.

4-Diazo-7-methoxy-2-methyl-1-(3-nitrophenyl)-1,4-dihydroisoquinolin-3(2*H*)-one (10j): prepared

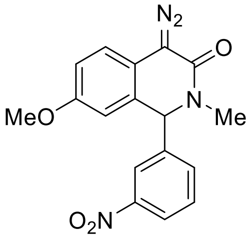

according to the general procedure GP1 from compound **11j** (scale – 2.6 mmol). Yield: 484 mg (55%). Orange solid; m.p. 153.8–155.5 °C. ^{1}H NMR (400 MHz, $CDCl_3$) δ 8.30 – 8.08 (m, 2H), 7.64 (dt, *J* = 7.8, 1.5 Hz, 1H), 7.56 (dd, *J* = 8.7, 7.7 Hz, 1H), 6.96 – 6.78 (m, 2H), 6.61 (d, *J* = 2.1 Hz, 1H), 5.60 (s, 1H), 3.74 (s, 3H), 3.00 (s, 3H). $^{13}C\{^1H\}$ NMR (101 MHz, $CDCl_3$) δ 162.4, 157.6, 148.6, 143.7, 132.6,

130.5, 129.2, 123.4, 121.7, 120.9, 114.9, 113.9, 113.3, 67.0, 63.6 (C=N_2), 55.5, 33.5. HRMS (ESI), *m/z* calcd for $C_{17}H_{15}N_4O_4$ [M+H]$^+$ 339.1088 found 339.1084.

7-Chloro-2-(4-chlorobenzyl)-4-diazo-1-(4-fluorophenyl)-1,4-dihydroisoquinolin-3(2*H*)-one (10k):

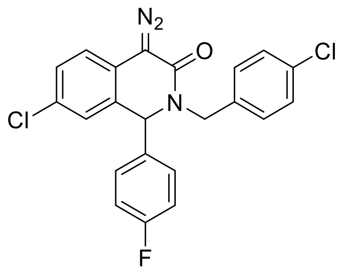

prepared according to the general procedure GP1 from compound **11k** (scale – 1.5 mmol). Yield: 588 mg (92%). Orange solid; m.p. 64.8–66.0 °C. ^{1}H NMR (400 MHz, $CDCl_3$) δ 7.32 (d, *J* = 8.4 Hz, 2H), 7.28 – 7.21 (m, 3H), 7.18 (d, *J* = 8.3 Hz, 2H), 7.07 (t, *J* = 8.5 Hz, 2H), 6.91 (d, *J* = 2.1 Hz, 1H), 6.87 (d, *J* = 8.4 Hz, 1H), 5.59 (d, *J* = 15.2 Hz, 1H), 5.35 (s, 1H), 3.57 (d, *J* = 15.2 Hz, 1H). ^{13}C{^{1}H} NMR (101 MHz, $CDCl_3$) δ 162.7 (d, $^1J_{C–F}$ = 248.4 Hz), 161.9, 136.7 (d, $^4J_{C–F}$ = 3.3 Hz), 134.6, 133.7, 130.7, 130.4, 129.6, 129.1, 128.8, 128.5 (d, $^3J_{C–F}$ = 8.2 Hz), 127.6, 120.6, 120.5, 116.4 (d, $^2J_{C–F}$ = 21.9 Hz), 64.3 (C=N_2), 62.8, 46.6. ^{19}F{^{1}H} NMR (377 MHz, $CDCl_3$) δ –112.56. HRMS (ESI), *m/z* calcd for $C_{22}H_{15}Cl_2FN_3O$ [M+H]$^+$ 426.0571/428.0546 found 426.0574/428.0546.

7-Chloro-1-(2-chlorophenyl)-4-diazo-2-methyl-1,4-dihydroisoquinolin-3(2*H*)-one (10l): prepared

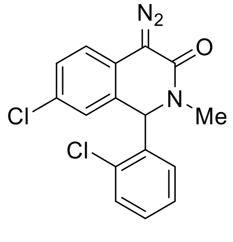

according to the general procedure GP1 from compound **11l** (scale – 1.8 mmol). Yield: 508 mg (85%). Orange solid; m.p. 157.3–153.8 °C. ^{1}H NMR (400 MHz, $CDCl_3$) δ 7.48 – 7.42 (m, 1H), 7.34 – 7.29 (m, 1H), 7.28 – 7.23 (m, 3H), 7.15 (d, *J* = 2.1 Hz, 1H), 6.85 (d, *J* = 8.4 Hz, 1H), 6.24 (s, 1H), 2.93 (s, 3H). ^{13}C{^{1}H} NMR (101 MHz, $CDCl_3$) δ 161.4, 138.8, 132.0, 130.7, 130.3, 130.1, 129.8, 128.81, 128.80, 128.3, 126.9, 120.9, 120.5, 64.1 (C=N_2), 62.3, 33.0. HRMS (ESI), *m/z* calcd for $C_{16}H_{12}Cl_2N_3O$ [M+H]$^+$ 332.0352/334.0325 found 332.0354/334.0330.

7-Chloro-4-diazo-2-(4-fluorophenyl)-1-(*p*-tolyl)-1,4-dihydroisoquinolin-3(2*H*)-one (10m): prepared

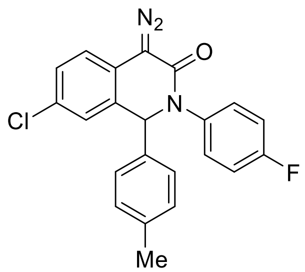

according to the general procedure GP1 from compound **11m** (scale – 1.5 mmol). Yield: 470 mg (80%). Light orange solid; m.p. 150.1–151.6 °C. ^{1}H NMR (400 MHz, $CDCl_3$) δ 7.30 (dd, *J* = 8.5, 2.2 Hz, 1H), 7.14 – 7.04 (m, 5H), 7.08 – 6.97 (m, 5H), 6.93 (d, *J* = 8.4 Hz, 1H), 5.75 (s, 1H), 2.33 (s, 3H). ^{13}C{^{1}H} NMR (101 MHz, $CDCl_3$) δ 162.0, 161.5 (d, $^1J_{C–F}$ = 247.3 Hz), 138.5, 138.2, 136.7 (d, $^4J_{C–F}$ = 3.3 Hz), 131.6, 130.8, 129.8, 129.6 (d, $^3J_{C–F}$ = 8.6 Hz), 128.7, 127.4, 126.7, 121.0, 120.6, 116.1 (d, $^2J_{C–F}$ = 22.7 Hz), 68.7, 65.0 (C=N_2), 21.1. ^{19}F{^{1}H} NMR (377 MHz, $CDCl_3$) δ –114.00. HRMS (ESI), *m/z* calcd for $C_{22}H_{15}ClFN_3NaO$ [M+Na]$^+$ 414.0780/416.0759 found 414.0785/416.0760.

7-Chloro-4-diazo-1-(4-nitrophenyl)-2-propyl-1,4-dihydroisoquinolin-3(2*H*)-one (10n): prepared according to the general procedure GP1 from compound **11n** (scale – 2.5 mmol). Yield: 482 mg (52%). Orange amorphous solid. ^{1}H NMR (400 MHz, $CDCl_3$) δ 8.30 – 8.11 (m, 2H), 7.61 – 7.45 (m, 2H), 7.28 (dd, *J* = 8.5, 2.0 Hz, 1H), 7.10 (d, *J* = 2.1 Hz, 1H), 6.88 (d, *J* = 8.4 Hz, 1H), 5.66 (s, 1H), 4.02 (ddd, *J* = 13.9, 9.0, 6.7 Hz, 1H), 2.74 (ddd, *J* = 14.0, 8.9, 5.3 Hz, 1H), 1.77 – 1.43 (m, 2H), 0.91 (t, *J* = 7.4 Hz, 3H). ^{13}C{^{1}H} NMR (101 MHz, $CDCl_3$) δ 161.6, 148.4, 147.9, 130.8, 129.4, 129.1, 127.3, 127.2, 124.6, 121.3, 120.8, 64.2 (C=N_2), 47.5, 20.7, 11.24. HRMS (ESI), *m/z* calcd for $C_{18}H_{16}ClN_4O_3$ $[M+H]^+$ 371.0905/373.0883 found 371.0907/373.0885.

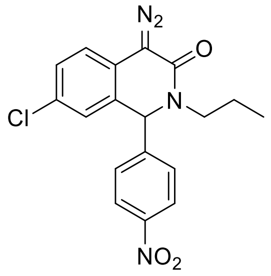

4-Diazo-1-(2-nitrophenyl)-2-(*p*-tolyl)-1,4-dihydroisoquinolin-3(2*H*)-one (10o): prepared according to the general procedure GP1 from compound **11o** (scale – 1.5 mmol). Yield: 525 mg (91%). Light orange solid; m.p. 159.4–161.3 °C. ^{1}H NMR (400 MHz, $CDCl_3$) δ 7.73 – 7.63 (m, 2H), 7.59 (td, *J* = 7.6, 1.4 Hz, 1H), 7.40 – 7.37 (m, 2H), 7.31 (d, *J* = 7.8 Hz, 1H), 7.12 (td, *J* = 7.6, 1.2 Hz, 1H), 7.08 (d, *J* = 8.0 Hz, 2H), 7.03 (d, *J* = 7.9 Hz, 1H), 6.98 (s, 1H), 6.87 – 6.78 (m, 2H), 2.32 (s, 3H). ^{13}C{^{1}H} NMR (101 MHz, $CDCl_3$) δ 161.7, 148.4, 137.7, 137.2, 136.9, 133.7, 130.1, 130.0, 129.0, 128.9, 128.6, 127.5, 127.3, 125.6, 124.0, 122.7, 119.4, 64.9 (C=N_2), 61.7, 21.1. HRMS (ESI), *m/z* calcd for $C_{22}H_{16}N_4NaO_3$ $[M+Na]^+$ 407.1115 found 407.1116.

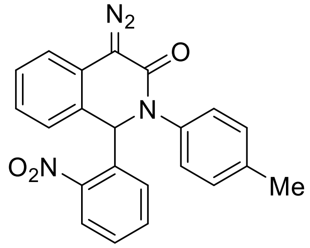

4-Diazo-1,2-bis(3,4-dimethoxyphenyl)-1,4-dihydroisoquinolin-3(2*H*)-one (10p): prepared according to the general procedure GP1 from compound **11p** (scale – 1.5 mmol). Yield: 541 mg (81%). Orange amorphous solid. ^{1}H NMR (400 MHz, $CDCl_3$) δ 7.34 – 7.24 (m, 4H), 6.84 (d, *J* = 8.4 Hz, 1H), 6.78 (d, *J* = 8.3 Hz, 1H), 6.75 – 6.67 (m, 3H), 6.66 (d, *J* = 2.1 Hz, 1H), 5.79 (s, 1H), 3.99 (d, *J* = 19.6 Hz, 1H), 3.88 (s, 3H), 3.85 (s, 3H), 3.85 (d, *J* = 19.5 Hz, 1H), 3.77 (s, 3H), 3.76 (s, 3H). ^{13}C{^{1}H} NMR (101 MHz, $CDCl_3$) δ 162.3, 149.2, 149.0, 148.9, 148.2, 134.7, 133.9, 130.2, 128.4, 127.3, 125.3, 122.3, 120.1, 119.7, 119.3, 111.7, 111.1, 111.0, 110.2, 69.3, 64.9 (C=N_2), 55.98, 55.97, 55.9, 55.8. HRMS (ESI), *m/z* calcd for $C_{25}H_{24}N_3O_5$ $[M+H]^+$ 446.1711 found 446.1719.

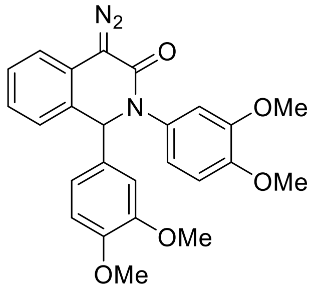

4-Diazo-1-(2-methoxyphenyl)-2-(4-methoxyphenyl)-1,4-dihydroisoquinolin-3(2*H*)-one (10q):

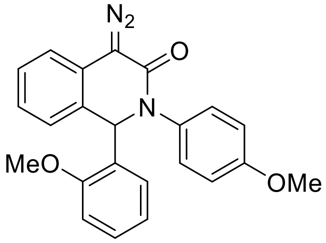

prepared according to the general procedure GP1 from compound **11q** (scale – 1.7 mmol). Yield: 490 mg (75%). Light orange solid; m.p. 128.8–129.4 °C. ^{1}H NMR (400 MHz, $CDCl_3$) δ 7.34 (dd, *J* = 7.6, 1.7 Hz, 1H), 7.30 (td, *J* = 7.6, 1.3 Hz, 1H), 7.25 – 7.17 (m, 2H), 7.05 (td, *J* = 7.5, 1.0 Hz, 1H), 7.01 (d, *J* = 8.9 Hz, 2H), 6.97 (dd, *J* = 7.8, 1.2 Hz, 1H), 6.91 (td, *J* = 7.5, 1.1 Hz, 1H), 6.85 – 6.76 (m, 3H), 6.43 (s, 1H), 3.77 (s, 3H), 3.69 (s, 3H). ^{13}C{^{1}H} NMR (101 MHz, $CDCl_3$) δ 162.9, 158.3, 156.0, 134.0, 130.7, 130.5, 129.2, 128.8, 128.1, 128.0, 126.9, 125.0, 122.6, 121.2, 119.1, 114.1, 111.3, 65.0 (C=N_2), 62.7, 55.6, 55.4. HRMS (ESI), *m/z* calcd for $C_{23}H_{20}N_3O_3$ $[M+H]^+$ 386.1499 found 386.1500.

4-Diazo-1-(naphthalen-1-yl)-2-phenyl-1,4-dihydroisoquinolin-3(2*H*)-one (10r): prepared according

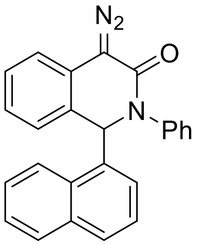

to the general procedure GP1 from compound **11r** (scale – 2.0 mmol). Yield: 623 mg (83%). Orange amorphous solid. ^{1}H NMR (400 MHz, $CDCl_3$) δ 8.07 – 8.00 (m, 1H), 7.87 – 7.80 (m, 1H), 7.76 (dd, *J* = 8.2, 1.2 Hz, 1H), 7.51 – 7.47 (m, 1H), 7.46 – 7.41 (m, 2H), 7.37 (dd, *J* = 8.2, 7.2 Hz, 1H), 7.31 (ddd, *J* = 8.4, 7.3, 1.7 Hz, 1H), 7.18 – 7.09 (m, 3H), 7.05 (dd, *J* = 7.7, 1.1 Hz, 1H), 7.01 – 6.91 (m, 4H), 6.78 (s, 1H). ^{13}C{^{1}H} NMR (101 MHz, $CDCl_3$) δ 162.2, 140.7, 137.3, 134.1, 130.4, 130.1, 129.11, 129.07, 127.0, 128.5, 127.9, 127.4, 127.3, 126.9, 126.6, 125.7, 125.4, 125.2, 123.1, 122.1, 119.3, 66.1, 65.2 (C=N_2). HRMS (ESI), *m/z* calcd for $C_{25}H_{18}N_3O$ $[M+H]^+$ 376.1444 found 376.1448.

5-Diazo-5,8,9,13b-tetrahydro-6*H*-isoquinolino[1,2-*a*]isoquinolin-6-one (10s): prepared according to

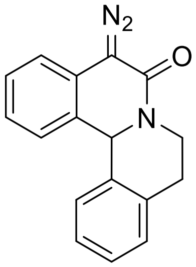

the general procedure GP1 from compound **11s** (scale – 3.0 mmol). Yield: 545 mg (66%). Beige solid; m.p. 131.4–133.6 °C. ^{1}H NMR (400 MHz, $CDCl_3$) δ 7.43 (td, *J* = 7.5, 1.4 Hz, 1H), 7.34 – 7.29 (m, 1H), 7.27 – 7.18 (m, 3H), 7.11 (ddd, *J* = 8.6, 6.9, 2.1 Hz, 1H), 6.98 (dd, *J* = 7.9, 1.1 Hz, 1H), 6.83 (dd, *J* = 7.8, 1.4 Hz, 1H), 5.77 (s, 1H), 4.69 (ddd, *J* = 12.4, 6.8, 4.4 Hz, 1H), 3.42 (ddd, *J* = 12.5, 9.0, 5.9 Hz, 1H), 3.38 – 3.27 (m, 1H), 2.99 – 2.88 (m, 1H). ^{13}C{^{1}H} NMR (101 MHz, $CDCl_3$) δ 163.3, 137.5, 135.3, 129.2, 128.8, 128.7, 127.7, 126.11, 126.08, 124.8, 124.4, 124.1, 119.8, 64.7 (C=N_2), 60.2, 42.5, 27.3. HRMS (ESI), *m/z* calcd for $C_{17}H_{13}N_3NaO$ $[M+Na]^+$ 298.0951 found 298.0949.

General procedure for the hydroarylation of diazo compounds 10 (GP2)

To a stirred mixture of arene (18 mmol, 60 equiv or 1.5 mmol, 5 equiv) and TfOH (0.45 mmol, 1.5 equiv) the solution of diazo compound **10** (0.3 mmol) in DCM (0.5 mL) was added dropwise during 1 min (vigorous gas evolution). Upon stirring for 15 min at room temperature, the mixture was diluted with DCM (5 mL), washed with sat. aq. $NaHCO_3$, dried over $CaCl_2$, and evaporated. The crude material was subjected to column chromatography on silica gel. Elution with *n*-hexane/acetone (5 to 35% of acetone) gave compound **9**, subsequent elution with acetone gave isoquinolone **15**.

(1*R/S*,4*R/S*)-1,2,4-Triphenyl-1,4-dihydroisoquinolin-3(2*H*)-one (9a) and 1,2-diphenylisoquinolin-3(2*H*)-one (15a): prepared according to the general procedure GP2 from diazo compound **10a** and benzene (60 equiv). Eluent: *n*-hexane/acetone (5% to 35%, then 100% of acetone).

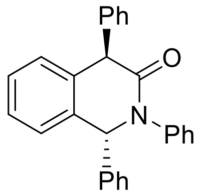

Compound **9a**: Yield: 94 mg (83%). White solid; m.p. 176.1–177.9 °C. ^{1}H NMR (400 MHz, $CDCl_3$) δ 7.46 – 7.37 (m, 2H), 7.38 – 7.18 (m, 16H), 6.85 (d, *J* = 7.6 Hz, 1H), 6.12 (s, 1H), 4.96 (s, 1H). ^{13}C{^{1}H} NMR (101 MHz, $CDCl_3$) δ 170.4, 142.0, 139.9, 138.2, 135.85, 135.84, 130.4, 128.9, 128.8, 128.5, 128.1, 127.9, 127.8, 127.3, 127.2, 127.0, 126.8, 126.7, 126.3, 68.0, 53.0. HRMS (ESI), *m/z* calcd for $C_{27}H_{22}NO$ $[M+H]^+$ 376.1696 found 376.1703.

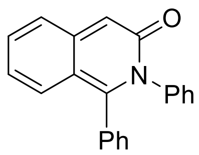

Compound **15a**: Yield: 12 mg (13%). Yellow solid; m.p. 232.8–234.7 °C (decomp.). ^{1}H NMR (400 MHz, $CDCl_3$) δ 7.35 (d, *J* = 8.8 Hz, 1H), 7.31 – 7.18 (m, 7H), 7.18 – 7.11 (m, 2H), 7.12 – 7.05 (m, 2H), 7.00 (d, *J* = 8.9 Hz, 1H), 6.91 (s, 1H), 6.78 (ddd, *J* = 9.0, 6.4, 1.2 Hz, 1H). ^{13}C{^{1}H} NMR (101 MHz, $CDCl_3$) δ 161.2, 151.5, 143.5, 139.3, 132.8, 131.5, 130.2, 128.9, 128.8, 128.7, 128.3, 128.2, 128.0, 125.2, 122.1, 116.7, 110.4. HRMS (ESI), *m/z* calcd for $C_{21}H_{16}NO$ $[M+H]^+$ 298.1226 found 298.1228.

(1*R/S*,4*R/S*)-2-Methyl-1,4-diphenyl-1,4-dihydroisoquinolin-3(2*H*)-one (9b): prepared according to the general procedure GP2 from diazo compound **10b** and benzene (60 equiv). Eluent: *n*-hexane/acetone (5% to 35% of acetone). Yield: 75 mg (80%). White amorphous solid. ^{1}H NMR (400 MHz, $CDCl_3$) δ 7.43 – 7.29 (m, 8H), 7.26 – 7.22 (m, 2H), 7.21 – 7.18 (m, 1H), 7.18 – 7.12 (m, 2H), 6.87 – 6.81 (m, 1H), 5.67 (d, *J* = 1.4 Hz, 1H), 4.90 (s, 1H), 3.02 (s, 3H). ^{13}C{^{1}H} NMR (101 MHz, $CDCl_3$) δ 170.1, 140.8, 140.3, 135.0, 134.6, 129.7, 129.1, 128.62, 128.60, 128.1, 127.6, 127.1, 126.9, 126.5, 67.2, 51.5, 34.1. HRMS (ESI), *m/z* calcd for $C_{22}H_{20}NNaO$ $[M+Na]^+$ 336.1359 found 336.1361.

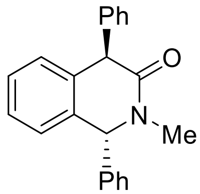

(1*R/S*,4*R/S*)-7-Chloro-1,2,4-triphenyl-1,4-dihydroisoquinolin-3(2*H*)-one (9c) and 7-chloro-1,2-diphenylisoquinolin-3(2*H*)-one (15c): prepared according to the general procedure GP2 from diazo compound **10c** and benzene (60 equiv). Eluent: *n*-hexane/acetone (5% to 35%, then 100% of acetone).

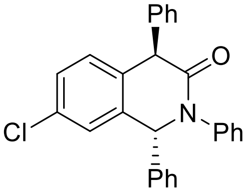

Compound **9c**: Yield: 85 mg (69%). White solid; m.p. 131.5–133.3 °C. ^{1}H NMR (400 MHz, $CDCl_3$) δ 7.43 – 7.19 (m, 17H), 6.79 (d, *J* = 8.3 Hz, 1H), 6.05 (s, 1H), 4.92 (s, 1H). ^{13}C{^{1}H} NMR (101 MHz, $CDCl_3$) δ 169.9, 141.7, 139.3, 137.9, 137.4, 134.4, 132.9, 130.2, 129.7, 129.0, 128.9, 128.7, 128.2, 128.1, 127.5, 127.2, 126.9, 126.8, 126.2, 67.6, 52.5. HRMS (ESI), *m/z* calcd for $C_{27}H_{21}ClNO$ $[M+H]^+$ 410.1306/412.1288 found 410.1307/412.1287.

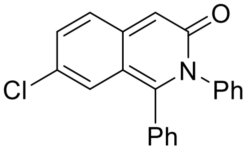

Compound **15c**: Yield: 28 mg (27%). Yellow amorphous solid. ^{1}H NMR (400 MHz, $CDCl_3$) δ 7.35 – 7.24 (m, 6H), 7.24 – 7.19 (m, 1H), 7.18 – 7.10 (m, 3H), 7.10 – 7.05 (m, 2H), 6.96 (dt, *J* = 1.9, 0.9 Hz, 1H), 6.90 (d, *J* = 1.0 Hz, 1H). ^{13}C{^{1}H} NMR (101 MHz, $CDCl_3$) δ 161.0, 150.8, 141.5, 139.0, 132.8, 132.2, 130.1, 129.2, 128.9, 128.5, 128.3, 128.2, 127.4, 127.2, 125.9, 116.7, 111.3. HRMS (ESI), *m/z* calcd for $C_{21}H_{15}ClNO$ $[M+H]^+$ 332.0837/334.807 found 332.0844/334.809.

(1*R/S*,4*R/S*)-5-Chloro-1,2,4-triphenyl-1,4-dihydroisoquinolin-3(2*H*)-one (9d): prepared according to the general procedure GP2 from diazo compound **10d** and benzene (60 equiv). Eluent: *n*-hexane/acetone (5% to 35% of acetone). Yield: 106 mg (86%). White solid; m.p. 210.6–212.0 °C. ^{1}H NMR (400 MHz, $CDCl_3$) δ 7.41 (dt, *J* = 8.0, 1.0 Hz, 1H), 7.38 – 7.29 (m, 5H), 7.23 – 7.11 (m, 6H), 7.10 – 7.03 (m, 3H), 6.91 – 6.85 (m, 2H), 6.68 (dd, *J* = 7.9, 1.1 Hz, 1H), 6.08 (d, *J* = 1.0 Hz, 1H), 5.56 (s, 1H). ^{13}C{^{1}H} NMR (101 MHz, $CDCl_3$) δ 169.4, 140.2, 140.0, 138.03, 138.00, 133.7, 132.9, 129.7, 129.2, 128.7, 128.50, 128.49, 128.4, 128.2, 128.1, 128.0, 127.4, 126.9, 126.6, 67.1, 50.5. HRMS (ESI), *m/z* calcd for $C_{27}H_{21}ClNO$ $[M+H]^+$ 410.1306/412.1288 found 410.1311/412.1289.

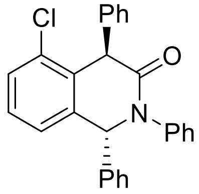

(1*R/S*,4*R/S*)-6-Chloro-2-methyl-1,4-diphenyl-1,4-dihydroisoquinolin-3(2*H*)-one (9e): prepared according to the general procedure GP2 from diazo compound **10h** and benzene (60 equiv). Eluent: *n*-hexane/acetone (5% to 35% of acetone). Yield: 84 mg (81%). White solid; m.p. 153.6–155.0 °C. ^{1}H NMR (400 MHz, $CDCl_3$) δ 7.44 – 7.31 (m, 6H), 7.30 – 7.26 (m, 2H), 7.24 – 7.20 (m, 2H), 7.17 (ddd, *J* = 8.4, 2.2, 0.8 Hz, 1H), 7.06 (d, *J* = 8.3 Hz, 1H), 6.85 (dd, *J* = 2.2, 1.0 Hz, 1H), 5.70 – 5.59 (m, 1H), 4.86 (s, 1H), 3.00 (s, 3H). ^{13}C{^{1}H} NMR (101 MHz, $CDCl_3$) δ 169.4, 140.4, 139.6, 136.9, 133.6, 133.1, 129.5, 129.2,

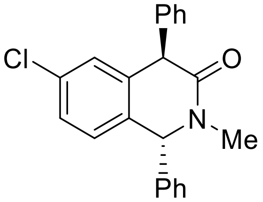

128.8, 128.4, 128.3, 128.1, 127.5, 127.3, 127.1, 66.7, 51.3, 34.1. HRMS (ESI), *m/z* calcd for $C_{22}H_{18}ClNO$ $[M+H]^+$ 348.1150/350.1128 found 348.1151/350.1126.

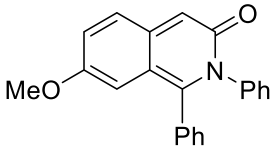

7-Methoxy-1,2-diphenylisoquinolin-3(2*H*)-one (15f): prepared according to the general procedure GP2 from diazo compound **10e** and benzene (60 equiv). Eluent: *n*-hexane/acetone (5% to 35%, then 100% of acetone). Yield: 83 mg (85%). Orange solid; m.p. 97.2–99.8 °C. ^{1}H NMR (400 MHz, $CDCl_3$) δ 7.31 (d, *J* = 9.4 Hz, 1H), 7.29 – 7.24 (m, 5H), 7.22 – 7.17 (m, 1H), 7.16 – 7.12 (m, 2H), 7.11 – 7.06 (m, 2H), 7.00 (dd, *J* = 9.4, 2.4 Hz, 1H), 6.92 (s, 1H), 6.13 (d, *J* = 2.4 Hz, 1H), 3.56 (s, 3H). $^{13}C\{^1H\}$ NMR (101 MHz, $CDCl_3$) δ 161.0, 154.3, 147.8, 140.7, 139.5, 133.3, 130.3, 128.79, 128.76, 128.7, 128.1, 128.0, 127.5, 127.1, 116.8, 111.7, 101.9, 54.9. HRMS (ESI), *m/z* calcd for $C_{22}H_{18}NO_2$ $[M+H]^+$ 328.1332 found 328.1327.

(1*R/S*,4*R/S*)-5-Methoxy-1,2,4-triphenyl-1,4-dihydroisoquinolin-3(2*H*)-one (9g) and 5-methoxy-1,2-diphenylisoquinolin-3(2*H*)-one (15g): prepared according to the general procedure GP2 from diazo compound **10f** and benzene (60 equiv). Eluent: *n*-hexane/acetone (5% to 35%, then 100% of acetone).

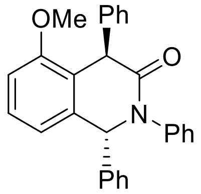

Compound **9g**: Yield: 37 mg (30%). White solid; m.p. 233.3–235.0 °C. ^{1}H NMR (400 MHz, $CDCl_3$) δ 7.38 – 7.30 (m, 4H), 7.28 – 7.24 (m, 1H), 7.23 – 7.11 (m, 6H), 7.11 – 7.03 (m, 3H), 6.96 – 6.88 (m, 2H), 6.83 (d, *J* = 8.2 Hz, 1H), 6.36 (d, *J* = 7.9 Hz, 1H), 6.10 (s, 1H), 5.47 (s, 1H), 3.75 (s, 3H). $^{13}C\{^1H\}$ NMR (101 MHz, $CDCl_3$) δ 170.2, 156.3, 140.8, 140.7, 140.1, 136.7, 129.6, 128.51, 128.46, 128.4, 128.3, 127.9, 127.8, 126.9, 126.7, 123.9, 119.8, 109.50, 67.3, 55.7, 47.2. HRMS (ESI), *m/z* calcd for $C_{28}H_{24}NO_2$ $[M+H]^+$ 406.1802 found 406.1808.

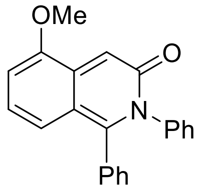

Compound **15g**: Yield: 17 mg (17%). Yellow amorphous solid. ^{1}H NMR (400 MHz, $CDCl_3$) δ 7.35 (d, *J* = 1.0 Hz, 1H), 7.30 – 7.24 (m, 5H), 7.23 – 7.17 (m, 1H), 7.16 – 7.11 (m, 2H), 7.10 – 7.07 (m, 2H), 6.57 (dt, *J* = 8.9, 1.0 Hz, 1H), 6.49 (d, *J* = 7.2 Hz, 1H), 3.97 (s, 3H). $^{13}C\{^1H\}$ NMR (101 MHz, $CDCl_3$) δ 161.3, 152.5, 150.3, 139.3, 137.7, 133.2, 130.3, 128.8, 128.73, 128.71, 128.1, 127.9, 121.7, 119.8, 117.3, 106.7, 105.6, 55.6. HRMS (ESI), *m/z* calcd for $C_{22}H_{18}NO_2$ $[M+H]^+$ 328.1332 found 328.1337.

(1*R/S*,4*R/S*)-2-(4-Methoxyphenyl)-7-methyl-4-phenyl-1-(4-(trifluoromethyl)phenyl)-1,4-dihydro-isoquinolin-3(2*H*)-one (9h) and 2-(4-methoxyphenyl)-7-methyl-1-(4-(trifluoromethyl)phenyl)-isoquinolin-3(2*H*)-one (15h): prepared according to the general procedure GP2 from diazo compound **10i** and benzene (60 equiv). Eluent: *n*-hexane/acetone (5% to 35%, then 100% of acetone).

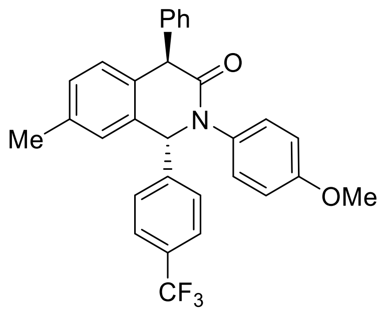

Compound **9h**: Yield: 60 mg (41%). White solid; m.p. 96.9–98.0 °C. ^{1}H NMR (400 MHz, $CDCl_3$) δ 7.61 (d, *J* = 8.0 Hz, 2H), 7.46 – 7.31 (m, 4H), 7.36 – 7.32 (m, 1H), 7.31 – 7.23 (m, 2H), 7.17 – 7.01 (m, 4H), 6.88 – 6.79 (m, 2H), 6.77 (d, *J* = 7.9 Hz, 1H), 6.07 (s, 1H), 4.94 (s, 1H), 3.79 (s, 3H), 2.36 (s, 3H). ^{13}C{^{1}H} NMR (101 MHz, $CDCl_3$) δ 170.4, 158.2, 144.6, 138.7, 137.0, 134.54, 134.47, 132.8, 130.2, 130.1 (q, $^{2}J_{C–F}$ = 32.7 Hz), 129.1, 128.6, 128.4, 128.2, 127.7, 127.3, 126.8, 125.8 (q, $^{3}J_{C–F}$ = 3.8 Hz), 123.9 (q, $^{1}J_{C–F}$ = 272.1 Hz), 114.3, 68.1, 55.4, 52.4, 21.1. ^{19}F{^{1}H} NMR (377 MHz, $CDCl_3$) δ –62.52. HRMS (ESI), *m/z* calcd for $C_{30}H_{25}F_3NO_2$ $[M+H]^+$ 488.1832 found 488.1836.

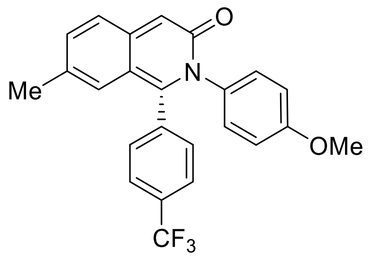

Compound **15h**: Yield: 47 mg (38%). Yellow solid; m.p. 142.6–144.7 °C. ^{1}H NMR (400 MHz, $CDCl_3$) δ 7.58 (d, *J* = 7.9 Hz, 2H), 7.33 – 7.23 (m, 3H), 7.10 (dd, *J* = 8.9, 1.6 Hz, 1H), 6.97 (d, *J* = 8.9 Hz, 2H), 6.91 (d, *J* = 0.9 Hz, 1H), 6.78 (d, *J* = 8.9 Hz, 2H), 6.59 (t, *J* = 1.3 Hz, 1H), 3.75 (s, 3H), 2.19 (d, *J* = 1.2 Hz, 3H). ^{13}C{^{1}H} NMR (101 MHz, $CDCl_3$) δ 161.3, 159.1, 148.1, 142.2, 136.9, 134.6, 132.0, 131.6, 130.8, 130.7 (q, $^{2}J_{C–F}$ = 32.8 Hz), 129.6, 125.3, 125.1 (q, $^{3}J_{C–F}$ = 3.8 Hz), 124.5, 123.6 (q, $^{1}J_{C–F}$ = 272.5 Hz), 116.7, 114.3, 111.4, 55.4, 21.5. ^{19}F{^{1}H} NMR (377 MHz, $CDCl_3$) δ –62.83. HRMS (ESI), *m/z* calcd for $C_{24}H_{19}F_3NO_2$ $[M+H]^+$ 410.1362 found 410,1370.

(1*R/S*,4*R/S*)-7-Chloro-2-(4-chlorobenzyl)-1-(4-fluorophenyl)-4-phenyl-1,4-dihydroisoquinolin-3(2*H*)-one (9i) and 7-chloro-2-(4-chlorobenzyl)-1-(4-fluorophenyl)isoquinolin-3(2*H*)-one (15i): prepared according to the general procedure GP2 from diazo compound **10k** and benzene (60 equiv). Eluent: *n*-hexane/acetone (5% to 35%, then 100% of acetone).

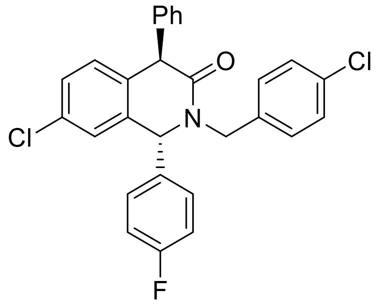

Compound **9i**: Yield: 103 mg (72%). White solid; m.p. 135.8–137.5 °C. ^{1}H NMR (400 MHz, $CDCl_3$) δ 7.46 – 7.32 (m, 3H), 7.27 – 7.17 (m, 6H), 7.16 – 7.06 (m, 3H), 6.99 (d, *J* = 8.3 Hz, 2H), 6.95 (d, *J* = 2.1 Hz, 1H), 6.81 (d, *J* = 8.4 Hz, 1H), 5.59 (d, *J* = 15.1 Hz, 1H), 5.46 (s, 1H), 4.95 (s, 1H), 3.63 (d, *J* = 15.0 Hz, 1H). ^{13}C{^{1}H} NMR (101 MHz, $CDCl_3$) δ 169.8, 162.6 (d, $^{1}J_{C–F}$ = 248.3 Hz), 139.7, 135.9, 135.6 (d, $^{4}J_{C–F}$ = 3.4 Hz), 134.9, 133.5, 133.04, 133.01, 130.3, 129.5, 129.4, 129.1 (d, $^{3}J_{C–F}$ = 8.3 Hz), 128.90, 128.88, 128.2, 127.6, 126.5, 116.4 (d, $^{2}J_{C–F}$ = 21.7 Hz), 62.6, 51.2, 47.2. ^{19}F{^{1}H} NMR (377 MHz, $CDCl_3$) δ –112.73. HRMS (ESI), *m/z* calcd for $C_{28}H_{21}Cl_2FNO$ $[M+H]^+$ 476.0979/478.0956 found 476.0987/478.0966.

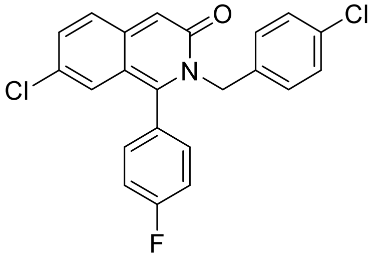

Compound **15i**: Yield: 16 mg (13%). Yellow amorphous solid. ^{1}H NMR (400 MHz, $CDCl_3$) δ 7.31 (d, *J* = 9.3 Hz, 1H), 7.24 – 7.11 (m, 5H), 7.06 (dd, *J* = 8.0, 5.1 Hz, 2H), 6.92 (s, 1H), 6.80 (d, *J* = 8.4 Hz, 2H), 6.77 – 6.70 (m, 1H), 5.33 (br.s, 2H). $^{13}C\{^{1}H\}$ NMR (101 MHz, $CDCl_3$) δ 163.5 (d, $^{1}J_{C-F}$ = 252.0 Hz), 160.6, 149.6, 140.9, 134.9, 133.46, 132.9, 131.4 (d, $^{3}J_{C-F}$ = 8.5 Hz), 128.7, 128.4, 127.9, 127.7 (d, $^{4}J_{C-F}$ = 3.8 Hz), 127.0, 125.2, 117.7, 116.3 (d, $^{2}J_{C-F}$ = 22.0 Hz), 111.1, 49.7. $^{19}F\{^{1}H\}$ NMR (377 MHz, $CDCl_3$) δ –108.90. HRMS (ESI), *m/z* calcd for $C_{22}H_{15}Cl_2FNO$ $[M+H]^+$ 398.0509/400.0484 found 398.0513/400.0486.

(1*R/S*,4*R/S*)-7-Chloro-1-(2-chlorophenyl)-2-methyl-4-phenyl-1,4-dihydroisoquinolin-3(2*H*)-one (9j) and 7-chloro-1-(2-chlorophenyl)-2-methylisoquinolin-3(2*H*)-one (15j): prepared according to the general procedure GP2 from diazo compound **10i** and benzene (60 equiv). Eluent: *n*-hexane/acetone (5% to 35%, then 100% of acetone).

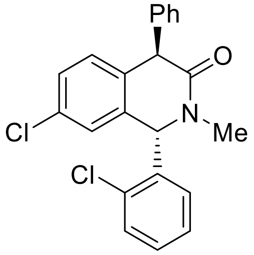

Compound **9j**: Yield: 84 mg (73%). White solid; m.p. 153.8–154.9 °C. ^{1}H NMR (400 MHz, $CDCl_3$) δ 7.53 – 7.46 (m, 1H), 7.40 – 7.34 (m, 2H), 7.34 – 7.29 (m, 3H), 7.26 – 7.20 (m, 2H), 7.20 – 7.12 (m, 2H), 7.09 (d, *J* = 2.2 Hz, 1H), 6.89 (dd, *J* = 8.4, 0.9 Hz, 1H), 6.34 (d, *J* = 1.9 Hz, 1H), 4.97 (s, 1H), 2.88 (s, 3H). $^{13}C\{^{1}H\}$ NMR (101 MHz, $CDCl_3$) δ 169.1, 141.1, 138.5, 134.9, 133.13, 133.10, 132.9, 130.6, 130.4, 129.8, 129.4, 129.1, 128.8, 128.3, 128.1, 127.4, 126.1, 62.7, 50.5, 33.4. HRMS (ESI), *m/z* calcd for $C_{22}H_{18}Cl_2NO$ $[M+H]^+$ 382.0760/384.0735 found 382.0767/384.0740.

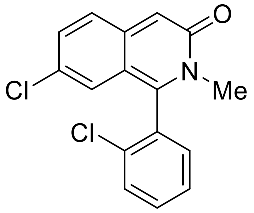

Compound **15j**: Yield: 22 mg (21%). Yellow amorphous solid. ^{1}H NMR (400 MHz, $CDCl_3$) δ 7.67 (dd, *J* = 8.1, 1.2 Hz, 1H), 7.60 (td, *J* = 7.7, 1.7 Hz, 1H), 7.54 (td, *J* = 7.5, 1.4 Hz, 1H), 7.35 – 7.29 (m, 2H), 7.14 (dd, *J* = 9.2, 2.0 Hz, 1H), 6.85 (s, 1H), 6.74 (dt, *J* = 1.9, 0.8 Hz, 1H), 3.53 (s, 3H). $^{13}C\{^{1}H\}$ NMR (101 MHz, $CDCl_3$) δ 160.8, 147.5, 140.5, 133.7, 132.5, 131.8, 131.6, 130.7, 130.6, 127.8, 127.7, 127.1, 124.7, 116.8, 110.6, 34.8. HRMS (ESI), *m/z* calcd for $C_{16}H_{12}Cl_2NO$ $[M+H]^+$ 304.0291/306.0263 found 304.0296/306.0267.

(1*R/S*,4*R/S*)-1-(2-Nitrophenyl)-4-phenyl-2-(*p*-tolyl)-1,4-dihydroisoquinolin-3(2*H*)-one (9k) and 1-(2-nitrophenyl)-2-(*p*-tolyl)isoquinolin-3(2*H*)-one (15k): prepared according to the general procedure GP2 from diazo compound **10o** and benzene (60 equiv). Eluent: *n*-hexane/acetone (5% to 35%, then 100% of acetone).

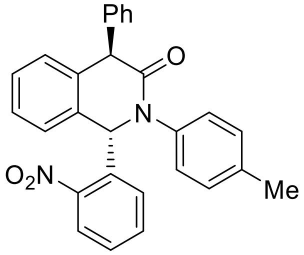

Compound **9k**: Yield: 87 mg (67%). White solid; m.p. 105.6–107.3 °C. ^{1}H NMR (400 MHz, $CDCl_3$) δ 7.70 (dd, *J* = 8.1, 1.3 Hz, 1H), 7.55 (td, *J* = 7.6, 1.4 Hz, 1H), 7.44 (dd, *J* = 8.0, 1.4 Hz, 1H), 7.41 – 7.34 (m, 3H), 7.33 – 7.22 (m, 5H), 7.14 (dd, *J* = 7.6, 1.6 Hz, 1H), 7.11 – 7.07 (m, 1H), 7.06 (d, *J* = 1.5 Hz, 1H), 7.01 (d, *J* = 8.1 Hz, 2H), 6.83 (d, *J* = 8.3 Hz, 2H), 5.19 (s, 1H), 2.26 (s, 3H). ^{13}C{^{1}H} NMR (101 MHz, $CDCl_3$) δ 169.5, 149.8, 140.2, 137.6, 137.2, 136.2, 134.9, 133.4, 133.2, 130.8, 129.7, 129.1, 129.0, 128.8, 128.7, 128.4, 127.4, 127.3, 127.2, 126.9, 124.1, 61.5, 52.1, 21.0. HRMS (ESI), *m/z* calcd for $C_{28}H_{23}N_2O_3$ $[M+H]^+$ 435.1703 found 435.1709.

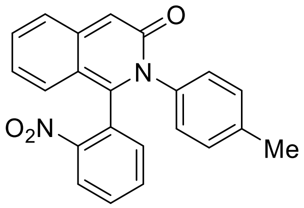

Compound **15k**: Yield: 26 mg (24%). Yellow solid; m.p. 247.5–249.3 °C (decomp.). ^{1}H NMR (400 MHz, $CDCl_3$) δ 8.11 (dd, *J* = 8.2, 1.3 Hz, 1H), 7.65 (td, *J* = 7.6, 1.3 Hz, 1H), 7.55 (td, *J* = 7.9, 1.5 Hz, 1H), 7.42 – 7.33 (m, 2H), 7.22 (ddd, *J* = 8.8, 6.3, 1.3 Hz, 1H), 7.13 – 6.99 (m, 3H), 6.94 (s, 1H), 6.88 (dd, *J* = 8.0, 2.1 Hz, 1H), 6.75 (ddd, *J* = 9.1, 6.2, 1.0 Hz, 1H), 6.69 (dd, *J* = 8.9, 1.2 Hz, 1H), 2.25 (s, 3H). ^{13}C{^{1}H} NMR (101 MHz, $CDCl_3$) δ 161.4, 147.5, 147.2, 143.3, 138.8, 136.2, 133.5, 133.0, 131.4, 130.7, 130.2, 129.6, 128.4, 128.1, 126.9, 126.3, 125.6, 125.0, 122.7, 115.8, 111.2, 21.1. HRMS (ESI), *m/z* calcd for $C_{22}H_{17}N_2O_3$ $[M+H]^+$ 357.1234 found 357.1238.

(1*R/S*,4*R/S*)-1,2-Bis(3,4-dimethoxyphenyl)-4-phenyl-1,4-dihydroisoquinolin-3(2*H*)-one (9l) and 1,2-bis(3,4-dimethoxyphenyl)isoquinolin-3(2*H*)-one (15l): prepared according to the general procedure GP2 from diazo compound **10p** and benzene (60 equiv). Eluent: *n*-hexane/acetone (5% to 35%, then 100% of acetone).

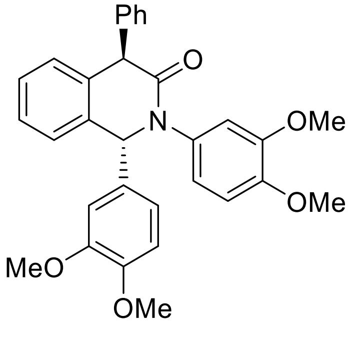

Compound **9l**: Yield: 86 mg (58%). Pale beige amorphous solid. ^{1}H NMR (400 MHz, $CDCl_3$) δ 7.47 – 7.35 (m, 2H), 7.36 – 7.23 (m, 4H), 7.20 (dd, *J* = 7.2, 1.9 Hz, 1H), 6.91 (d, *J* = 7.3 Hz, 1H), 6.85 – 6.62 (m, 6H), 5.99 (s, 1H), 5.01 (s, 1H), 3.87 (s, 3H), 3.85 (s, 3H), 3.77 (s, 3H), 3.75 (s, 3H). ^{13}C{^{1}H} NMR (101 MHz, $CDCl_3$) δ 170.4, 148.8, 148.7, 147.8, 138.5, 135.7, 134.9, 132.6, 130.1, 128.6, 128.3, 127.9, 127.3, 127.0, 126.4, 120.1, 119.2, 111.4, 111.0, 110.9, 110.8, 68.1, 56.0, 55.93, 55.89, 52.9. HRMS (ESI), *m/z* calcd for $C_{31}H_{30}NO_5$ $[M+H]^+$ 496.2119 found 496.2127.

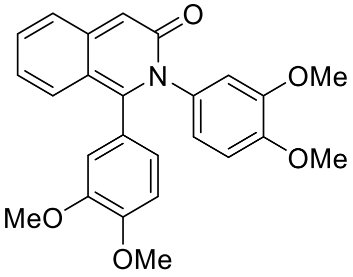

Compound **15l**: Yield: 30 mg (25%). Yellow amorphous solid. ^{1}H NMR (400 MHz, $CDCl_3$, 2 rotamers (55:45)) δ 7.35 – 7.28 (m, 1H), 7.27 – 7.18 (m, 1H), 7.14 – 7.02 (m, 1H), 6.86 (d, J = 2.3 Hz, 1H), 6.82 – 6.71 (m, 4.55H), 6.68 (d, J = 2.3 Hz, 0.45H), 6.64 (d, J = 1.9 Hz, 0.45H), 6.62 – 6.57 (m, 1H), 6.52 (d, J = 2.1 Hz, 0.55H), 3.88 (s, 1.35H), 3.87 (s, 1.65H), 3.84 (s, 1.65H), 3.83 (s, 1.35H), 3.76 (s, 1.35H), 3.73 (s, 1.65H), 3.72 (s, 1.35H), 3.71 (s, 1.65H). ^{13}C{^{1}H} NMR (101 MHz, $CDCl_3$, 2 rotamers (55:45)) δ 161.40, 149.3, 149.0, 148.9, 148.6, 148.4, 148.3, 143.4, 132.2, 131.5, 128.5, 128.4, 125.22, 125.20, 125.1, 123.6, 123.2, 122.03, 122.02, 121.0, 120.9, 117.0, 113.6, 113.2, 112.1, 111.9, 110.9, 110.6, 110.4, 110.22, 110.20, 110.1, 55.95, 55.92, 55.89, 55.87, 55.82, 55.78. HRMS (ESI), *m/z* calcd for $C_{25}H_{24}NO_5$ [M+H]$^+$ 418.1649 found 418.1657.

(1*R/S*,4*R/S*)-1-(Naphthalen-1-yl)-2,4-diphenyl-1,4-dihydroisoquinolin-3(2*H*)-one (9m) and 1-(naphthalen-1-yl)-2-phenylisoquinolin-3(2*H*)-one (15m): prepared according to the general procedure GP2 from diazo compound **10r** and benzene (60 equiv). Eluent: *n*-hexane/acetone (5% to 35%, then 100% of acetone).

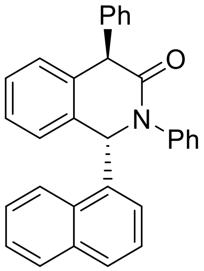

Compound **9m**: Yield: 89 mg (70%). White solid; m.p. 206.9–208.6 °C. ^{1}H NMR (400 MHz, $CDCl_3$) δ 7.85 (d, J = 8.9 Hz, 1H), 7.80 (dd, J = 8.2, 1.4 Hz, 1H), 7.73 (dd, J = 8.3, 1.5 Hz, 1H), 7.49 – 7.22 (m, 11H), 7.10 (td, J = 7.5, 1.6 Hz, 1H), 7.04 – 6.93 (m, 3H), 6.91 – 6.85 (m, 2H), 6.82 (d, J = 7.9 Hz, 1H), 6.69 (s, 1H), 5.36 (s, 1H). ^{13}C{^{1}H} NMR (101 MHz, $CDCl_3$) δ 170.1, 140.7, 139.7, 135.3, 135.2, 134.3, 134.2, 130.9, 129.32, 129.29, 128.9, 128.84, 126.83, 128.8, 128.6, 128.2, 128.1, 127.8, 127.3, 127.2, 126.8, 126.1, 125.6, 124.9, 124.5, 66.2, 53.0. HRMS (ESI), *m/z* calcd for $C_{31}H_{23}NNaO$ [M+Na]$^+$ 448.1672 found 448.1673.

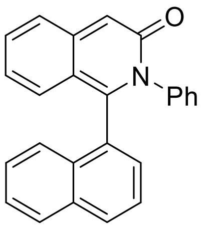

Compound **15m**: Yield: 25 mg (26%). Yellow solid; m.p. 116.4–118.5 °C. ^{1}H NMR (400 MHz, $CDCl_3$) δ 7.85 – 7.75 (m, 2H), 7.52 – 7.43 (m, 2H), 7.42 – 7.33 (m, 3H), 7.31 – 7.17 (m, 4H), 7.06 – 7.02 (m, 1H), 7.01 (s, 1H), 6.92 – 6.83 (m, 2H), 6.75 (dd, J = 8.9, 1.2 Hz, 1H), 6.67 (ddd, J = 9.0, 6.3, 1.1 Hz, 1H). ^{13}C{^{1}H} NMR (101 MHz, $CDCl_3$) δ 161.5, 150.1, 143.3, 139.1, 132.9, 131.8, 131.6, 130.2, 129.7, 129.1, 128.6, 128.47, 128.45, 128.4, 128.2, 128.1, 127.1, 126.8, 126.4, 125.4, 125.2, 124.5, 122.3, 117.4, 110.9. HRMS (ESI), *m/z* calcd for $C_{25}H_{18}NO$ [M+H]$^+$ 348.1383 found 348.1388.

(5*R*/*S*,13b*R*/*S*)-5-Phenyl-5,8,9,13b-tetrahydro-6*H*-isoquinolino[1,2-*a*]isoquinolin-6-one (9n) and 8,9-dihydro-6*H*-isoquinolino[1,2-*a*]isoquinolin-6-one (15n): prepared according to the general procedure GP2 from diazo compound **10s** and benzene (60 equiv). Eluent: *n*-hexane/acetone (5% to 35%, then 100% of acetone).

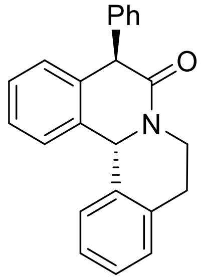

Compound **9n**: Yield: 49 mg (50%) as mixture of diastereomers 90:10. Pure compound **9n** was obtained after additional chromatographic purification, eluent: *n*-hexane/acetone (5% to 25% of acetone). White amorphous solid. ^{1}H NMR (400 MHz, $CDCl_3$) δ 7.45 – 7.30 (m, 2H), 7.30 – 7.19 (m, 4H), 7.18 – 7.07 (m, 5H), 7.05 – 6.96 (m, 2H), 5.85 (s, 1H), 4.87 (s, 1H), 4.66 (ddd, *J* = 12.9, 6.5, 4.7 Hz, 1H), 3.38 (ddd, *J* = 12.9, 9.1, 5.8 Hz, 1H), 3.04 (ddd, *J* = 15.8, 9.0, 6.4 Hz, 1H), 2.93 (dt, *J* = 16.1, 5.3 Hz, 1H). $^{13}C\{^{1}H\}$ NMR (101 MHz, $CDCl_3$) δ 169.6, 139.1, 136.6, 136.5, 135.6, 132.5, 129.2, 129.0, 128.6, 128.3, 128.2, 127.7, 127.1, 126.9, 126.5, 126.2, 125.7, 59.5, 52.6, 41.8, 28.0. HRMS (ESI), *m/z* calcd for $C_{23}H_{19}NNaO$ $[M+Na]^+$ 348.1359 found 348.1361.

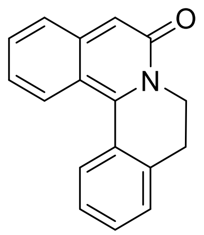

Compound **15n**: Yield: 25 mg (32%). Dark yellow amorphous solid. ^{1}H NMR (400 MHz, $CDCl_3$) δ 7.95 (d, *J* = 9.0 Hz, 1H), 7.85 (d, *J* = 7.5 Hz, 1H), 7.50 (td, *J* = 7.0, 6.5, 1.3 Hz, 1H), 7.46 – 7.39 (m, 2H), 7.35 (d, *J* = 8.7 Hz, 1H), 7.31 – 7.21 (m, 1H), 6.95 (ddd, *J* = 9.1, 6.4, 1.2 Hz, 1H), 6.79 (s, 1H), 4.44 (br.s, 1H), 2.98 (t, *J* = 6.1 Hz, 1H). $^{13}C\{^{1}H\}$ NMR (101 MHz, $CDCl_3$) δ 159.7, 145.9, 142.9, 138.8, 131.2, 130.9, 130.8, 128.9, 127.7, 127.6, 126.7, 125.8, 122.5, 115.3, 109.1, 41.4, 28.4. HRMS (ESI), *m/z* calcd for $C_{17}H_{14}NO$ $[M+H]^+$ 248.1070 found 248.1074.

(1*R/S*,4*R/S*)-1,2-Diphenyl-4-(*p*-tolyl)-1,4-dihydroisoquinolin-3(2*H*)-one (9o): prepared according to the general procedure GP2 from diazo compound **10a** and toluene (60 equiv). Eluent: *n*-hexane/acetone (5% to 35% of acetone). Yield: 112 mg (96%) as mixture of regioisomers 10:1. White solid; m.p. 112.3–115.0 °C. ^{1}H NMR (400 MHz, $CDCl_3$) δ 7.40 – 7.15 (m, 17H), 6.90 (dd, *J* = 7.5, 1.1 Hz, 1H), 6.13 (s, 1H), 4.94 (s, 1H), 2.41 (s, 3H). $^{13}C\{^{1}H\}$ NMR (101 MHz, $CDCl_3$) δ 170.6, 142.1, 140.0, 136.9, 136.0, 135.9, 135.1, 130.2, 129.3, 128.82, 128.79, 128.2, 127.8, 127.2, 126.9, 126.8, 126.6, 126.2, 67.9, 52.7, 21.2. HRMS (ESI), *m/z* calcd for $C_{28}H_{24}NO$ $[M+H]^+$ 390.1852 found 390.1853.

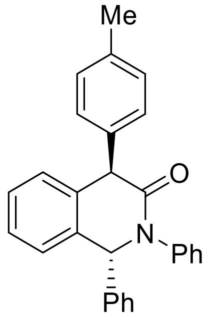

(1*R/S*,4*R/S*)-4-(4-Fluorophenyl)-1,2-diphenyl-1,4-dihydroisoquinolin-3(2*H*)-one (9p): prepared according to the general procedure GP2 from diazo compound **10a** and fluorobenzene (60 equiv). Eluent: *n*-hexane/acetone (5% to 35% of acetone). Yield: 76 mg (64%) as mixture diastereomers 95:5. White solid; m.p. 161.9–162.3 °C. ^{1}H NMR (400 MHz, $CDCl_3$) δ 7.38 – 7.20 (m, 15H), 7.11 (t, *J* = 8.7 Hz, 2H), 6.82 (d, *J* = 7.6 Hz, 1H), 6.12 (s, 1H), 4.94 (s, 1H). ^{13}C{^{1}H} NMR (101 MHz, $CDCl_3$) δ 170.2, 162.2 (d, $^{1}J_{C\text{-}F}$ = 245.7 Hz), 142.0, 139.7, 135.9, 135.7, 133.7 (d, $^{4}J_{C\text{-}F}$ = 3.3 Hz), 132.1 (d, $^{3}J_{C\text{-}F}$ = 8.0 Hz), 128.9, 128.8, 128.0, 127.9, 127.1, 127.0, 126.8, 126.6, 126.3, 115.4 (d, $^{2}J_{C\text{-}F}$ = 21.5 Hz), 68.0, 52.3. ^{19}F{^{1}H} NMR (377 MHz, $CDCl_3$) δ –115.43. HRMS (ESI), *m/z* calcd for $C_{27}H_{21}FNO$ $[M+H]^+$ 394.1602 found 394.1607.

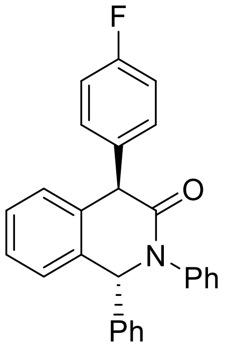

Additionally, elution with acetone afforded isoquinolinone **15a**. Yield: 17 mg (19%).

(1*R/S*,4*R/S*)-4-(4-Methoxyphenyl)-1,2-diphenyl-1,4-dihydroisoquinolin-3(2*H*)-one (9q): prepared according to the general procedure GP2 from diazo compound **10a** and anisole (60 equiv). Eluent: *n*-hexane/acetone (5% to 35% of acetone). Yield: 118 mg (97%) as mixture two diastereomers and regioisomer 68:9:23. Crystallization from *n*-hexane/acetone afforded mixture of regioisomers 94:6. White solid; m.p. 162.8–163.6 °C. ^{1}H NMR (400 MHz, $CDCl_3$) δ 7.47 – 7.16 (m, 15H), 6.97 (d, *J* = 8.4 Hz, 2H), 6.89 (d, *J* = 7.6 Hz, 2H), 6.13 (s, 2H), 4.92 (s, 2H), 3.86 (s, 3H). ^{13}C{^{1}H} NMR (101 MHz, $CDCl_3$) δ 170.7, 158.8, 142.2, 139.9, 136.2, 135.9, 131.4, 130.1, 128.9, 128.8, 128.1, 127.9, 127.8, 127.2, 126.9, 126.7, 126.6, 126.2, 114.0, 67.9, 55.3, 52.3. HRMS (ESI), *m/z* calcd for $C_{28}H_{24}NO_2$ $[M+H]^+$ 406.1802 found 406.1809.

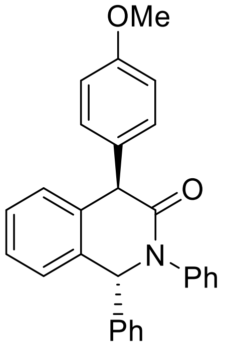

(1*R/S*,4*R/S*)-4-(2,5-Dimethylphenyl)-1,2-diphenyl-1,4-dihydroisoquinolin-3(2*H*)-one (9r): prepared according to the general procedure GP2 from diazo compound **10a** and *p*-xylene (60 equiv). Eluent: *n*-hexane/acetone (5% to 35% of acetone). Yield: 109 mg (90%) as mixture diastereomers 96:4. Pure compound **9r** was obtained by recrystallization from *n*-hexane/acetone. White solid; m.p. 186.5–187.9 °C. ^{1}H NMR (400 MHz, $CDCl_3$) δ 7.41 – 7.18 (m, 13H), 7.14 (d, *J* = 7.7 Hz, 1H), 7.09 (dd, *J* = 7.6, 1.9 Hz, 1H), 7.06 (d, *J* = 1.8 Hz, 1H), 6.74 (d, *J* = 7.8 Hz, 1H), 6.12 (s, 1H), 5.04 (s, 1H), 2.35 (s, 3H), 2.07 (s, 3H). ^{13}C{^{1}H} NMR (101 MHz, $CDCl_3$) δ 170.2, 142.3, 140.4, 136.4, 135.4, 135.3, 135.2, 134.1, 132.8, 131.0, 129.0, 128.9, 128.3, 127.9, 127.8, 127.4, 126.8, 126.7, 126.6, 126.5, 125.9, 68.3, 51.4, 21.0, 19.6. HRMS (ESI), *m/z* calcd for $C_{29}H_{26}NO$ $[M+H]^+$ 404.2009 found 404.2016.

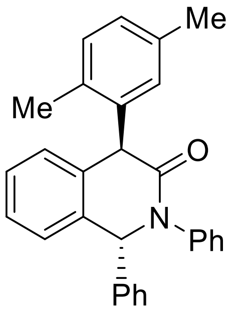

(1*R/S*,4*R/S*)-4-(2,4-Dimethylphenyl)-1,2-diphenyl-1,4-dihydroisoquinolin-3(2*H*)-one (9s): prepared according to the general procedure GP2 from diazo compound **10a** and *m*-xylene (60 equiv). Eluent: *n*-hexane/acetone (5% to 35% of acetone). Yield: 115 mg (95%) as mixture two diastereomers and regioisomer 90:5:5. Pure compound **9s** was obtained by recrystallization from *n*-hexane/acetone. White solid; m.p. 173.9–175.1 °C. ^{1}H NMR (400 MHz, $CDCl_3$) δ 7.41 – 7.25 (m, 11H), 7.25 – 7.16 (m, 2H), 7.12 (d, *J* = 7.5 Hz, 1H), 7.10 – 7.03 (m, 2H), 6.76 (d, *J* = 7.8 Hz, 1H), 6.11 (s, 1H), 5.05 (s, 1H), 2.37 (s, 3H), 2.10 (s, 3H). ^{13}C{^{1}H} NMR (101 MHz, $CDCl_3$) δ 170.3, 140.4, 137.02, 137.01, 135.5, 135.2, 133.7, 131.9, 131.8, 128.90, 128.88, 127.9, 127.8, 127.4, 126.8, 126.76, 126.74, 126.6, 126.4, 125.9, 68.2, 50.9, 21.1, 20.0. HRMS (ESI), *m/z* calcd for $C_{29}H_{26}NO$ $[M+H]^+$ 404.2009 found 404.2014.

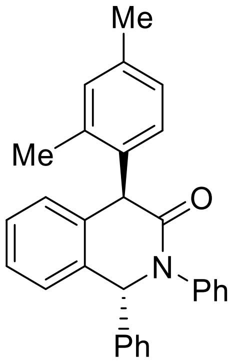

(1*R/S*,4*R/S*)-4-(5-Fluoro-2-methylphenyl)-1,2-diphenyl-1,4-dihydroisoquinolin-3(2*H*)-one (9t): prepared according to the general procedure GP2 from diazo compound **10a** and 4-fluorotoluene (60 equiv). Eluent: *n*-hexane/acetone (5% to 35% of acetone). Yield: 60 mg (49%) as mixture diastereomers 96:4. White solid; m.p. 119.7–122.6 °C. ^{1}H NMR (400 MHz, $CDCl_3$) δ 7.45 – 7.19 (m, 13H), 7.20 – 7.10 (m, 2H), 7.03 (dd, *J* = 10.0, 8.3 Hz, 1H), 6.83 (d, *J* = 7.7 Hz, 1H), 6.13 (s, 1H), 5.09 (s, 1H), 2.37 (s, 3H). ^{13}C{^{1}H} NMR (101 MHz, $CDCl_3$) δ 169.4, 159.5 (d, $^1J_{C\text{-}F}$ = 244.6 Hz), 142.2, 140.1, 135.4, 134.7, 133.5 (d, $^4J_{C\text{-}F}$ = 3.6 Hz), 133.1, 133.0, 129.8 (d, $^3J_{C\text{-}F}$ = 8.1 Hz), 129.0, 128.9, 127.90, 127.88, 127.1 (d, $^3J_{C\text{-}F}$ = 10.3 Hz), 126.8, 126.7, 126.5, 126.1, 125.0 (d, $^2J_{C\text{-}F}$ = 14.4 Hz), 115.4 (d, $^2J_{C\text{-}F}$ = 21.7 Hz), 68.2, 47.8 (d, $^4J_{C\text{-}F}$ = 1.7 Hz), 20.7. ^{19}F{^{1}H} NMR (377 MHz, $CDCl_3$) δ –120.20. HRMS (ESI), *m/z* calcd for $C_{28}H_{23}FNO$ $[M+H]^+$ 408.1758 found 408.1759.

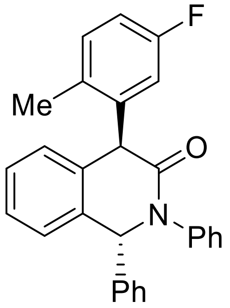

Additionally, elution with acetone afforded isoquinolinone **15a**. Yield: 26 mg (29%).

(1*R/S*,4*R/S*)-4-(2,4,6-Trimethylphenyl)-1,2-diphenyl-1,4-dihydroisoquinolin-3(2*H*)-one (9u): prepared according to the general procedure GP2 from diazo compound **10a** and mesitylene (60 equiv). Eluent: *n*-hexane/acetone (5% to 35% of acetone). Yield: 122 mg (98%) as mixture diastereomers 95:5. Pure compound **9u** was obtained by recrystallization from *n*-hexane–acetone. White solid; m.p. 228.3–229.4 °C. ^{1}H NMR (400 MHz, $CDCl_3$) δ 7.43 – 7.22 (m, 12H), 7.18 (td, *J* = 7.6, 1.3 Hz, 1H), 6.97 (s, 1H), 6.91 (s, 1H), 6.71 (d, *J* = 7.8 Hz, 1H), 6.09 (s, 1H), 5.35 (s, 1H), 2.34 (s, 3H), 2.33 (s, 3H), 1.92 (s, 3H). ^{13}C{^{1}H} NMR (101 MHz, $CDCl_3$) δ 170.2, 142.4,

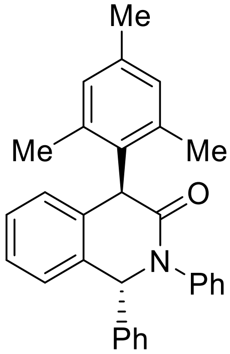

140.7, 137.5, 137.3, 136.6, 135.0, 134.9, 132.5, 130.5, 128.93, 128.91, 128.7, 128.0, 127.8, 126.7, 126.6, 126.5, 126.3, 125.9, 68.3, 47.0, 21.0, 20.8. HRMS (ESI), *m/z* calcd for $C_{30}H_{28}NO$ $[M+H]^+$ 418.2165 found 418.2168.

(1*R/S*,4*R/S*)-4-(2,5-Dimethoxyphenyl)-1,2-diphenyl-1,4-dihydroisoquinolin-3(2*H*)-one (9v):

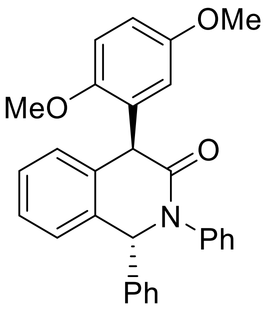

prepared according to the general procedure GP2 from diazo compound **10a** and 1,4-dimethoxybenzene (5 equiv). Eluent: *n*-hexane/acetone (5% to 35% of acetone). Yield: 120 mg (91%) as mixture diastereomers 90:10. Pure compound **9v** was obtained by recrystallization from *n*-hexane–acetone. White solid; m.p. 154.8–156.2 °C. ^{1}H NMR (400 MHz, $CDCl_3$) δ 7.38 – 7.15 (m, 13H), 6.96 – 6.84 (m, 3H), 6.80 (d, *J* = 7.8 Hz, 1H), 6.08 (s, 1H), 5.14 (s, 1H), 3.80 (s, 3H), 3.67 (s, 3H). $^{13}C\{^1H\}$ NMR (101 MHz, $CDCl_3$) δ 170.1, 153.7, 152.0, 142.4, 140.8, 135.5, 135.0, 128.9, 128.81, 128.78, 127.7, 127.6, 127.2, 126.9, 126.7, 126.6, 126.5, 125.9, 118.1, 113.4, 112.9, 68.3, 56.5, 55.8, 48.3. HRMS (ESI), *m/z* calcd for $C_{29}H_{26}NO_3$ $[M+H]^+$ 436.1907 found 436.1915.

(1*R/S*,4*R/S*)-4-(2,4-Dimethoxyphenyl)-1,2-diphenyl-1,4-dihydroisoquinolin-3(2*H*)-one (9w):

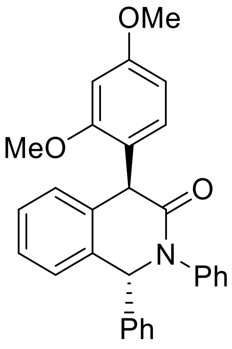

prepared according to the general procedure GP2 from diazo compound **10a** and 1,3-dimethoxybenzene (5 equiv). Eluent: *n*-hexane/acetone (5% to 35% of acetone). Yield: 112 mg (85%) as mixture two diastereomers and regioisomer 74:9:17. Crystallization from *n*-hexane–acetone afforded mixture of regioisomers 94:6. White solid; m.p. 161.5–163.8 °C. ^{1}H NMR (400 MHz, $CDCl_3$) δ 7.37 – 7.15 (m, 14H), 6.80 (d, *J* = 7.8 Hz, 1H), 6.61 – 6.52 (m, 2H), 6.08 (s, 1H), 5.12 (s, 1H), 3.86 (s, 3H), 3.70 (s, 3H). $^{13}C\{^1H\}$ NMR (101 MHz, $CDCl_3$) δ 170.6, 160.4, 158.6, 142.6, 140.7, 136.0, 135.2, 132.5, 128.83, 128.80, 127.7, 127.5, 127.2, 126.8, 126.50, 126.45, 126.4, 125.8, 119.9, 104.6, 99.4, 68.2, 55.7, 55.4, 47.6. HRMS (ESI), *m/z* calcd for $C_{29}H_{26}NO_3$ $[M+H]^+$ 436.1907 found 436.1914.

(1*R/S*,4*R/S*)-4-(5-Fluoro-2-methoxyphenyl)-1,2-diphenyl-1,4-dihydroisoquinolin-3(2*H*)-one (9x):

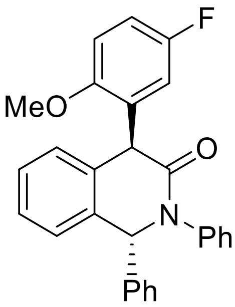

prepared according to the general procedure GP2 from diazo compound **10a** and 4-fluoroanisole (60 equiv). Eluent: *n*-hexane/acetone (5% to 35% of acetone). Yield: 108 mg (85%) as mixture diastereomers 94:6. Pure compound **9x** was obtained by recrystallization from *n*-hexane–acetone. White solid; m.p. 203.3–205.1 °C. ^{1}H NMR (400 MHz, $CDCl_3$) δ 7.47 – 7.13 (m, 13H), 7.09 – 6.99 (m, 2H), 6.95 – 6.85 (m, 1H), 6.78 (d, *J* = 7.7 Hz, 1H), 6.09 (s, 1H), 5.20 (s, 1H), 3.71 (s, 3H). $^{13}C\{^1H\}$

NMR (101 MHz, $CDCl_3$) δ 169.8, 157.0 (d, $^1J_{C\text{-}F}$ = 238.6 Hz), 153.9 (d, $^4J_{C\text{-}F}$ = 2.2 Hz), 142.3, 140.6, 135.1, 135.0, 129.1 (d, $^3J_{C\text{-}F}$ = 7.4 Hz), 128.93, 128.87, 127.8, 127.7, 127.0, 126.8, 126.8, 126.7, 126.6, 126.0, 118.9 (d, $^2J_{C\text{-}F}$ = 23.6 Hz), 114.7 (d, $^2J_{C\text{-}F}$ = 22.7 Hz), 112.5 (d, $^3J_{C\text{-}F}$ = 8.3 Hz), 68.3, 56.4, 47.8. $^{19}F\{^1H\}$ NMR (377 MHz, $CDCl_3$) δ –123.71. HRMS (ESI), *m/z* calcd for $C_{28}H_{23}FNO_2$ $[M+H]^+$ 424.1707 found 424.1713.

(1*R/S*,4*R/S*)-4-(5-Bromo-2-methoxyphenyl)-1,2-diphenyl-1,4-dihydroisoquinolin-3(2*H*)-one (9y):

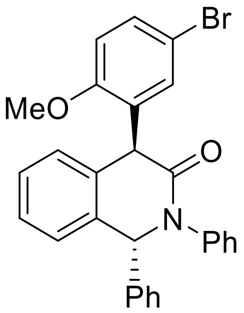

prepared according to the general procedure GP2 from diazo compound **10a** and 4-bromoanisole (5 equiv). Eluent: *n*-hexane/acetone (5% to 35% of acetone). Yield: 90 mg (62%) as mixture diastereomers 93:7. Pure compound **9y** was obtained by recrystallization from *n*-hexane–acetone. White solid; m.p. 204.9–206.3 °C. ^1H NMR (400 MHz, $CDCl_3$) δ 7.45 (dd, *J* = 8.7, 2.5 Hz, 1H), 7.40 (d, *J* = 2.5 Hz, 1H), 7.37 – 7.14 (m, 13H), 6.85 (d, *J* = 8.7 Hz, 1H), 6.76 (dt, *J* = 8.0, 1.4 Hz, 1H), 6.08 (s, 1H), 5.14 (s, 1H), 3.71 (s, 3H). $^{13}C\{^1H\}$ NMR (101 MHz, $CDCl_3$) δ 169.7, 156.9, 142.3, 140.5, 135.0, 134.9, 134.8, 131.5, 129.6, 128.9, 128.8, 127.8, 127.7, 127.0, 126.80, 126.78, 126.7, 126.6, 126.0, 113.2, 112.9, 68.3, 56.0, 47.9. HRMS (ESI), *m/z* calcd for $C_{28}H_{23}BrNO_2$ $[M+H]^+$ 484.0907/486.0890 found 484.0914/486.0897.

Additionally, elution with acetone afforded isoquinolinone **15a**. Yield: 5 mg (6%).

(1*R/S*,4*R/S*)-4-(4-(Methylthio)phenyl)-1,2-diphenyl-1,4-dihydroisoquinolin-3(2*H*)-one (9z):

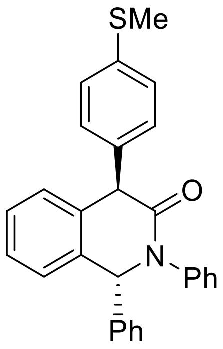

prepared according to the general procedure GP2 from diazo compound **10a** and thioanisole (60 equiv). Eluent: *n*-hexane/acetone (5% to 35% of acetone). Yield: 41 mg (32%) as mixture two diastereomers and regioisomer 88:4:8. Crystallization from *n*-hexane–acetone afforded pure compound **9z**. White solid; m.p. 180.4–181.8 °C. ^1H NMR (400 MHz, $CDCl_3$) δ 7.54 – 7.10 (m, 17H), 6.86 (dd, *J* = 7.6, 1.1 Hz, 1H), 6.11 (s, 1H), 4.91 (s, 1H), 2.52 (s, 3H). $^{13}C\{^1H\}$ NMR (101 MHz, $CDCl_3$) δ 170.3, 142.0, 139.8, 137.4, 135.9, 135.7, 134.9, 130.8, 128.9, 128.8, 128.0, 127.91, 127.87, 127.1, 127.0, 126.9, 126.7, 126.3, 67.9, 52.5, 16.0. HRMS (ESI), *m/z* calcd for $C_{28}H_{24}NOS$ $[M+H]^+$ 422.1573 found 422.1579.

(1*R/S*,4*R/S*)-3-Oxo-1,2-diphenyl-1,2,3,4-tetrahydroisoquinolin-4-yl formate (16): prepared according to the general procedure GP2 from diazo compound **10a** and *N*-methyl formanilide (5 equiv). Eluent: *n*-hexane/acetone (5% to 25% of acetone). Yield: 75 mg (73%) as mixture two diastereomers and regioisomer 85:15. Crystallization from *n*-hexane/acetone afforded pure compound **16**. White solid; m.p. 187.7–188.5 °C. ^{1}H NMR (400 MHz, $CDCl_3$) δ 8.50 (d, *J* = 1.0 Hz, 1H), 7.51 – 7.48 (m, 1H), 7.47 – 7.42 (m, 3H), 7.42 – 7.34 (m, 4H), 7.34 – 7.26 (m, 6H), 6.57 (s, 1H), 6.05 (s, 1H). ^{13}C{^{1}H} NMR (101 MHz, $CDCl_3$) δ 165.7, 159.9, 141.5, 138.6, 134.8, 130.9, 129.2, 129.1, 128.33, 128.32, 128.1, 127.2, 126.3, 126.02, 125.99, 124.3, 68.4, 68.1. HRMS (ESI), *m/z* calcd for $C_{22}H_{17}NNaO_3$ $[M+Na]^+$ 366.1095 found 366.1095.

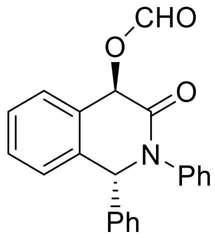

2. Crystallographic data for compounds 9a, 10c and 16

X-ray single crystal analysis was performed on a Rigaku XtaLAB Synergy-S diffractometer with monochromated CuKα radiation. Crystal growth was performed by slow evaporation of a solution in *n*-hexane/acetone mixture 2:1 at 5 °C. The crystal was kept at 100 K during data collection. Using Olex2[3], the structure was solved with the SHELXT[4] structure solution program using Intrinsic Phasing and refined with the SHELXL[5] refinement package using Least Squares minimization. CCDC 2158046 (**9a**), CCDC 2170881 (**10c**), and CCDC 2170877 (**16**) contain the supplementary crystallographic data for this file. These data can be obtained free of charge from The Cambridge Crystallographic Data Centre via https://www.ccdc.cam.ac.uk/.

Table S1. Crystal data and ORTEP representation for **9a (2158046)**

**Empirical Formula**	$C_{27}H_{21}NO$
**Formula weight**	375.45
**Temperature, K**	100.15
**Crystal system**	monoclinic
**Space group**	$P2_1/n$
**a/Å**	5.67820(10)
**b/Å**	18.7912(3)
**c/Å**	18.4603(3)
**α/Å**	90
**β/Å**	94.686(2)
**γ/Å**	90
**Volume/Å³**	1963.13(6)
**Z**	4
**ρ_{calc}g/cm³**	1.270
**μ/mm⁻¹**	0.595
**F(000)**	792.0
**Crystal size/mm³**	0.12 × 0.12 × 0.1
**Radiation**	CuKα (λ = 1.54184)
**2Θ range for data collection/°**	9.412 to 152.518
**Index ranges**	$-6 \leq h \leq 7, -22 \leq k \leq 23, -22 \leq l \leq 22$
**Reflections collected**	9001
**Independent reflections**	4005 [R_{int} = 0.0289, R_{sigma} = 0.0314]
**Data/restraints/parameters**	4005/0/281
**Goodness-of-fit on F^2**	1.041
**Final R indexes [I>=2σ (I)]**	R_1 = 0.0485, wR_2 = 0.1176
**Final R indexes [all data]**	R_1 = 0.0558, wR_2 = 0.1231
**Largest diff. peak/hole / e Å^{-3}**	0.38/-0.41

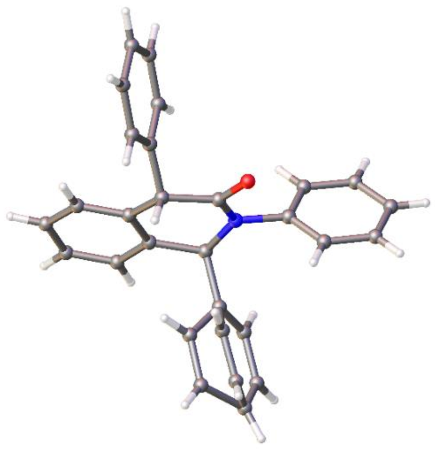

Figure S1. ORTEP representation of compound **9a** (thermal ellipsoids are shown at 50% probability)

**Table S2.** Crystal data and ORTEP representation for **10c (2170881)**	
**Empirical Formula**	$C_{21}H_{14}ClN_3O$
**Formula weight**	359.80
**Temperature, K**	100.15
**Crystal system**	monoclinic
**Space group**	$P2_1/c$
**a/Å**	13.2187(2)
**b/Å**	9.93980(10)
**c/Å**	12.9617(2)
**α/Å**	90
**β/Å**	91.9380(10)
**γ/Å**	90
**Volume/Å^3**	1702.08(4)
**Z**	4
**ρ_{calc}g/cm^3**	1.404
**μ/mm^{-1}**	2.106
**F(000)**	744.0
**Crystal size/mm^3**	0.1 × 0.05 × 0.04
**Radiation**	CuKα (λ = 1.54184)
**2Θ range for data collection/°**	6.69 to 160.914
**Index ranges**	$-16 \leq h \leq 16$, $-12 \leq k \leq 7$, $-16 \leq l \leq 13$
**Reflections collected**	14200
**Independent reflections**	3578 [R_{int} = 0.0345, R_{sigma} = 0.0285]
**Data/restraints/parameters**	3578/0/235
**Goodness-of-fit on F^2**	1.081
**Final R indexes [I>=2σ (I)]**	R_1 = 0.0372, wR_2 = 0.0908
**Final R indexes [all data]**	R_1 = 0.0397, wR_2 = 0.0925
**Largest diff. peak/hole / e Å^{-3}**	0.27/-0.32

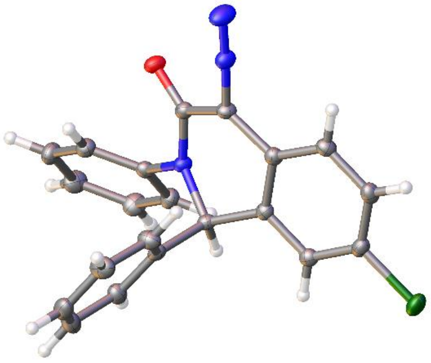

Figure S2. ORTEP representation of compound **10c** (thermal ellipsoids are shown at 50% probability)

**Table S3.** Crystal data and ORTEP representation for **16 (2170877)**	
**Empirical Formula**	$C_{22}H_{17}NO_3$
**Formula weight**	343.36
**Temperature, K**	100.15
**Crystal system**	orthorhombic
**Space group**	$P2_12_12_1$
**a/Å**	11.4269(6)
**b/Å**	11.6108(7)
**c/Å**	12.5127(8)
**α/Å**	90
**β/Å**	90
**γ/Å**	90
**Volume/Å³**	1660.13(17)
**Z**	4
**ρ_{calc}g/cm³**	1.374
**μ/mm⁻¹**	0.741
**F(000)**	720.0
**Crystal size/mm³**	0.06 × 0.01 × 0.01
**Radiation**	CuKα (λ = 1.54184)
**2Θ range for data collection/°**	10.394 to 160.51
**Index ranges**	$-14 \leq h \leq 13$, $-13 \leq k \leq 14$, $-15 \leq l \leq 15$
**Reflections collected**	6733
**Independent reflections**	3218 [R_{int} = 0.0820, R_{sigma} = 0.0808]
**Data/restraints/parameters**	3218/0/235
**Goodness-of-fit on F^2**	1.051
**Final R indexes [I>=2σ (I)]**	R_1 = 0.0623, wR_2 = 0.1658
**Final R indexes [all data]**	R_1 = 0.0665, wR_2 = 0.1704
**Largest diff. peak/hole / e Å⁻³**	0.43/-0.36

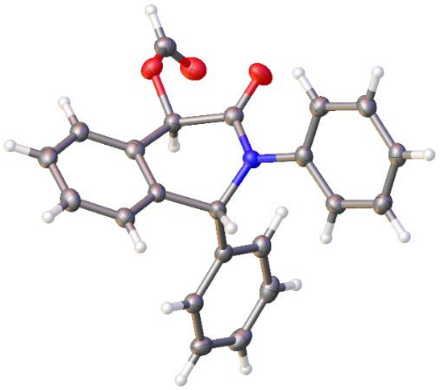

Figure S3. ORTEP representation of compound **16** (thermal ellipsoids are shown at 50% probability)

3. Biological data

Cell culture

NCI-H460 and A549 lung carcinoma cells were purchased from the ATCC. NCI-H460 and A549 cells were maintained in Advanced RPMI-1640 (Gibco, UK) supplemented with 5% fetal bovine serum (FBS, Gibco, UK), penicillin (100 UI mL^{-1}), streptomycin (100 µg mL^{-1}), and GlutaMax (2 mM, Gibco, UK). Cell line cultivation was performed under a humidified atmosphere of 95% air/5% CO_2 at 37 °C. Subconfluent monolayers, in the log growth phase, were harvested by a brief treatment with TrypLE Express solution (Gibco, UK) in phosphate buffered saline (PBS, Capricorn Scientific, Germany), and washed three times in serum-free PBS. The number of viable cells was determined by trypan blue exclusion.

Antiproliferative assay

The effects of the synthesized compounds on cell viability were determined using the MTT colorimetric test. All examined cells were diluted with the growth medium to 3.5 × 104 cells per mL and the aliquots (7 × 103 cells per 200 µL) were placed in individual wells in 96-multiplates (Eppendorf, Germany) and incubated for 24 h. The next day the cells were then treated with synthesized compounds separately at the final concentration 30 µM (or 300.0 µM concentration and diluted at various concentrations for determination of IC50) and incubated for 24 h at 37 °C in 5% CO_2 atmosphere. After incubation, the cells were then treated with 40 µL MTT solution (3-(4,5-dimethylthiazol-2-yl)-2,5-diphenyltetrazolium bromide, 5 mg mL^{-1} in PBS) and incubated for 4 h. After an additional 4 h of incubation, the medium with MTT was removed and DMSO (150 µL) was added to dissolve the formazan crystals. The plates were shaken for 10 min. The optical density of each well was determined at 560 nm using a microplate reader GloMax Multi+ (Promega, USA). Each of the tested compounds was evaluated for cytotoxicity in three separate experiments.

NCI-H460 cell viability in the presence of 30 μM concentration of compounds **9a–z**

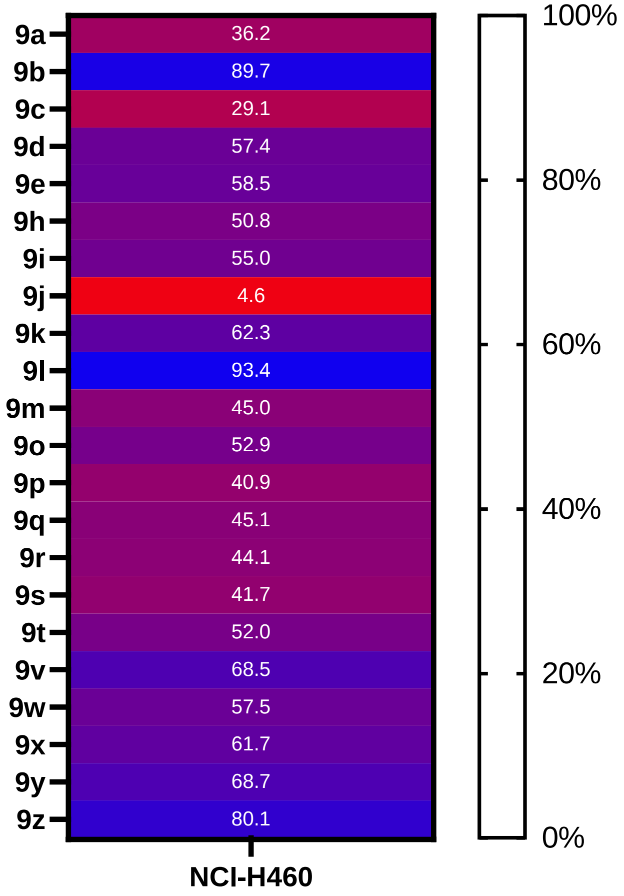

IC_{50} value of the most cytotoxic compound **9j** determined against A549 and NCI-H460 cell lines

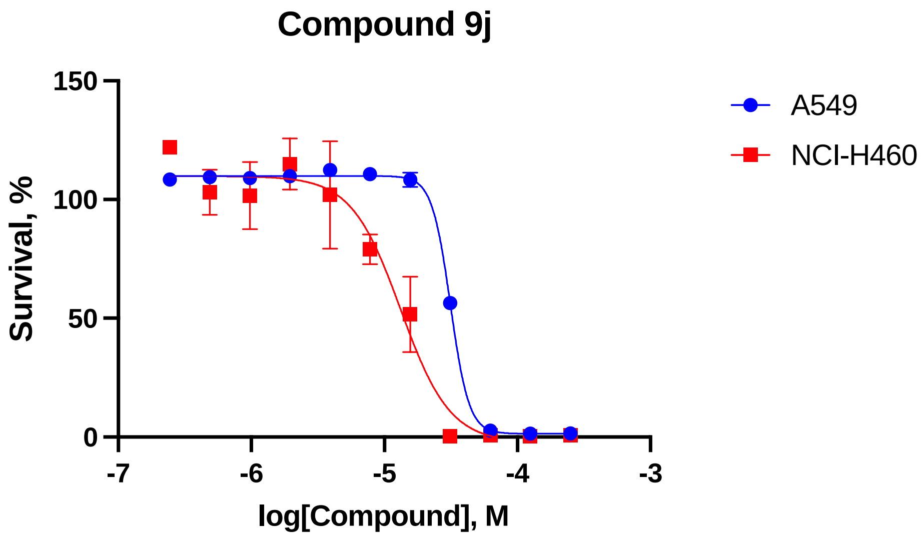

IC_{50} (A549) = 31.4 ± 0.48 μM

IC_{50} (NCI-H460) = 13.6 ± 3.34 μM

4. References

1. Zhang, Y.; Kindelin, P. J.; DeSchepper, D. J.; Zheng, C.; Klumpp, D. A. *Synthesis* **2006**, 1775-1780.
2. Venkov, A. P.; Mollov, N. M. *Synthesis* **1982**, 216-217.
3. Dolomanov, O. V; Bourhis, L. J.; Gildea, R. J.; Howard, J. A. K.; Puschmann, H. *J. Appl. Crystallogr.* **2009**, *42*, 339–341.
4. Sheldrick, G. M. A *Acta Crystallogr. Sec. A* **2015**, *71*, 3–8.
5. Sheldrick, G. M. *Acta Crystallogr. Sec. C* **2015**, *71*, 3–8.

5. Copies of ^{1}H, ^{13}C NMR, ^{19}F and NOESY NMR spectra

Copies of ^{1}H (400.13 MHz, $CDCl_3$) and $^{13}C\{^{1}H\}$ (100.61 MHz, $CDCl_3$) spectra of **11c**

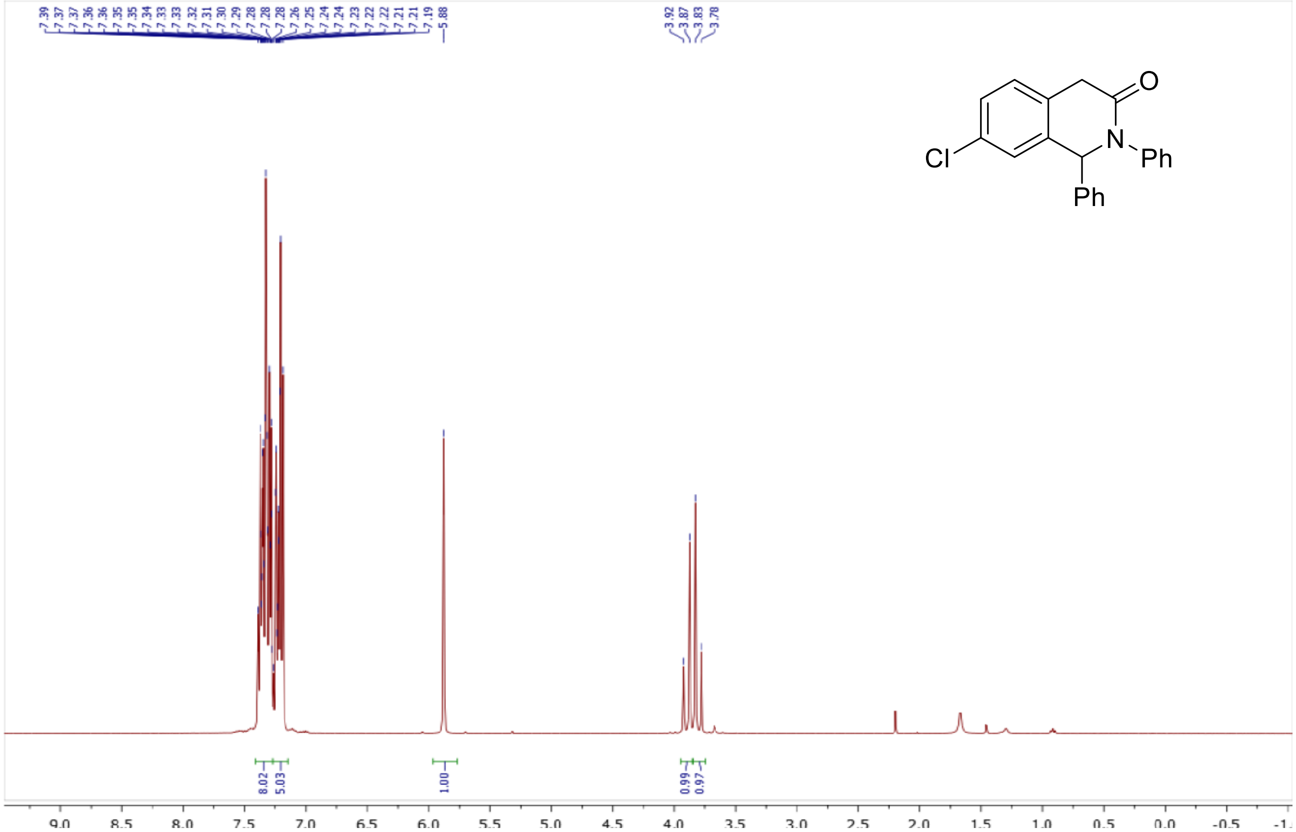

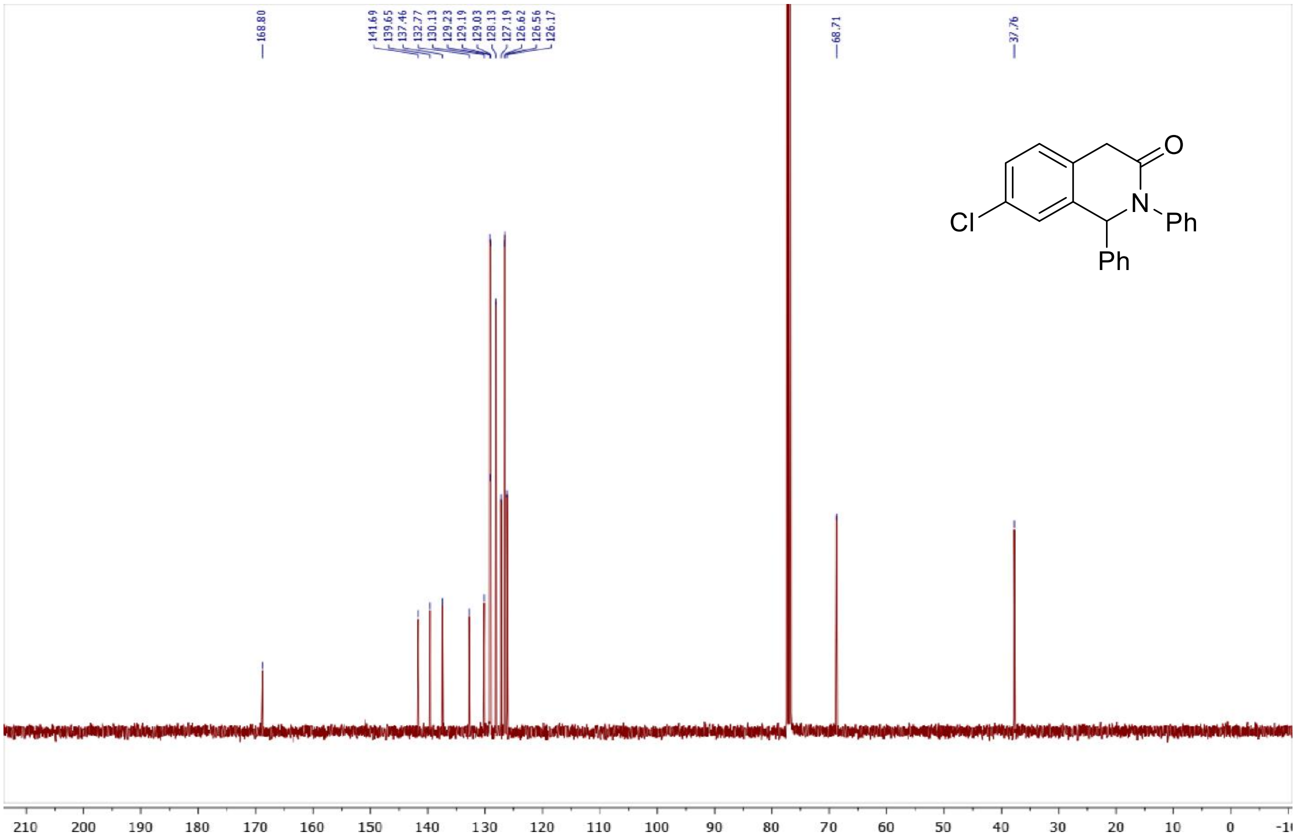

Copies of ^{1}H (400.13 MHz, $CDCl_3$) and $^{13}C\{^1H\}$ (100.61 MHz, $CDCl_3$) spectra of **11d**

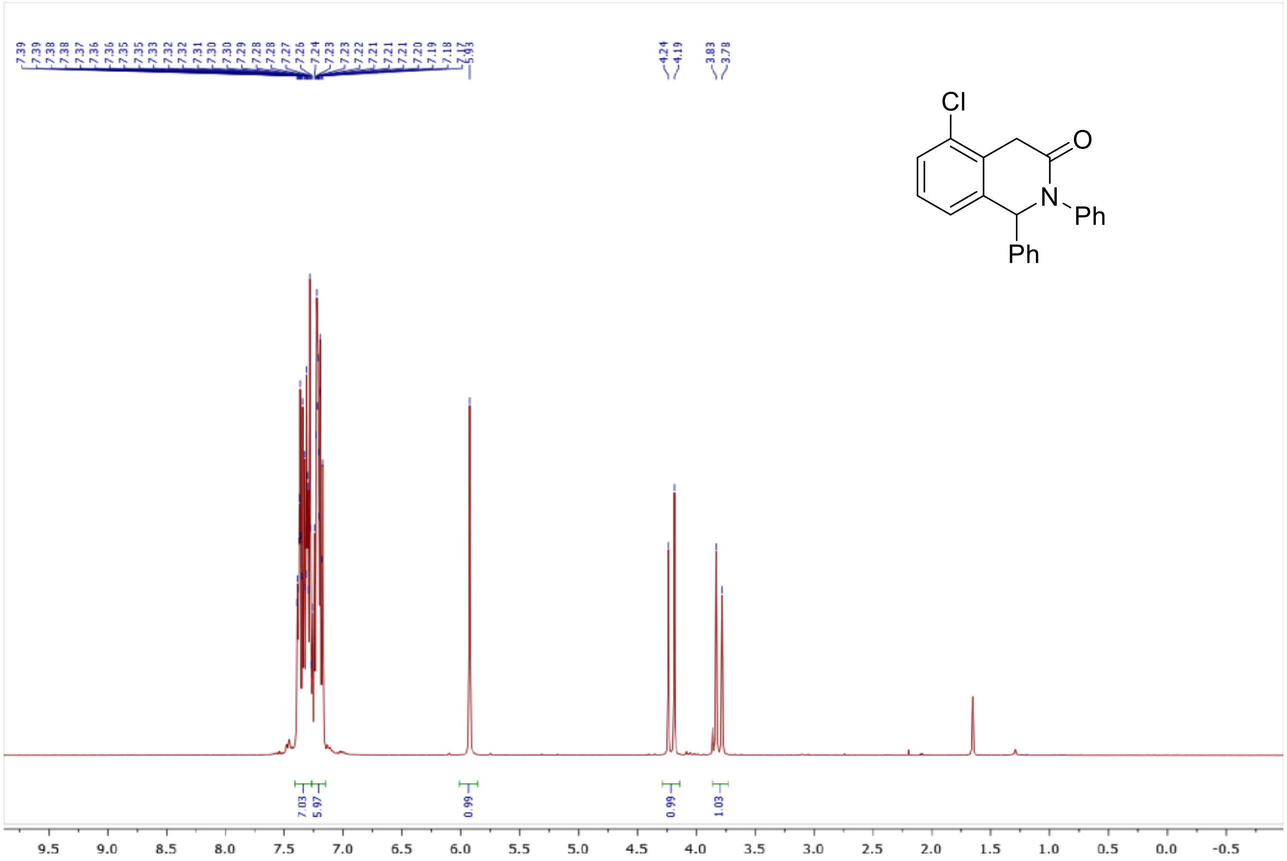

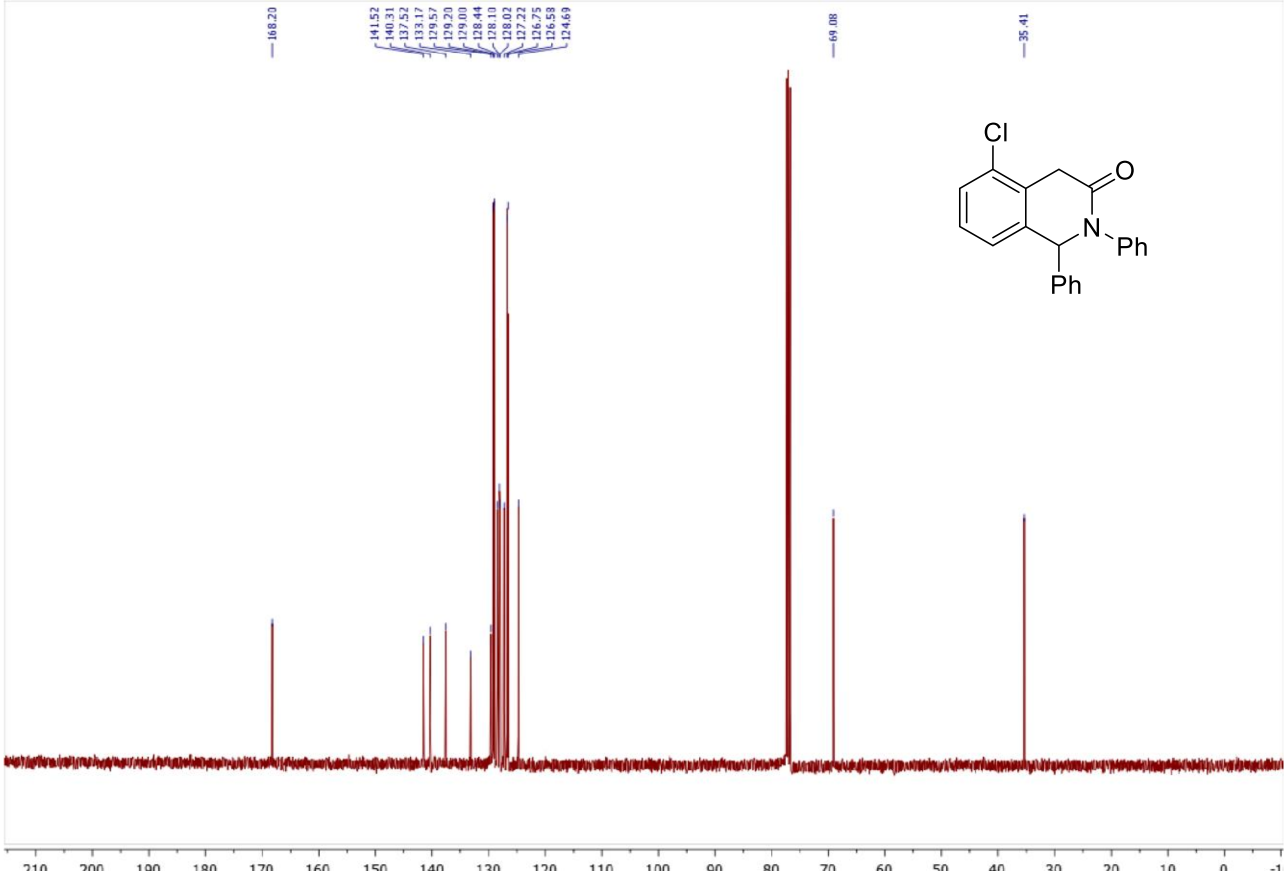

Copies of ^{1}H (400.13 MHz, $CDCl_3$) and $^{13}C\{^{1}H\}$ (100.61 MHz, $CDCl_3$) spectra of **11e**

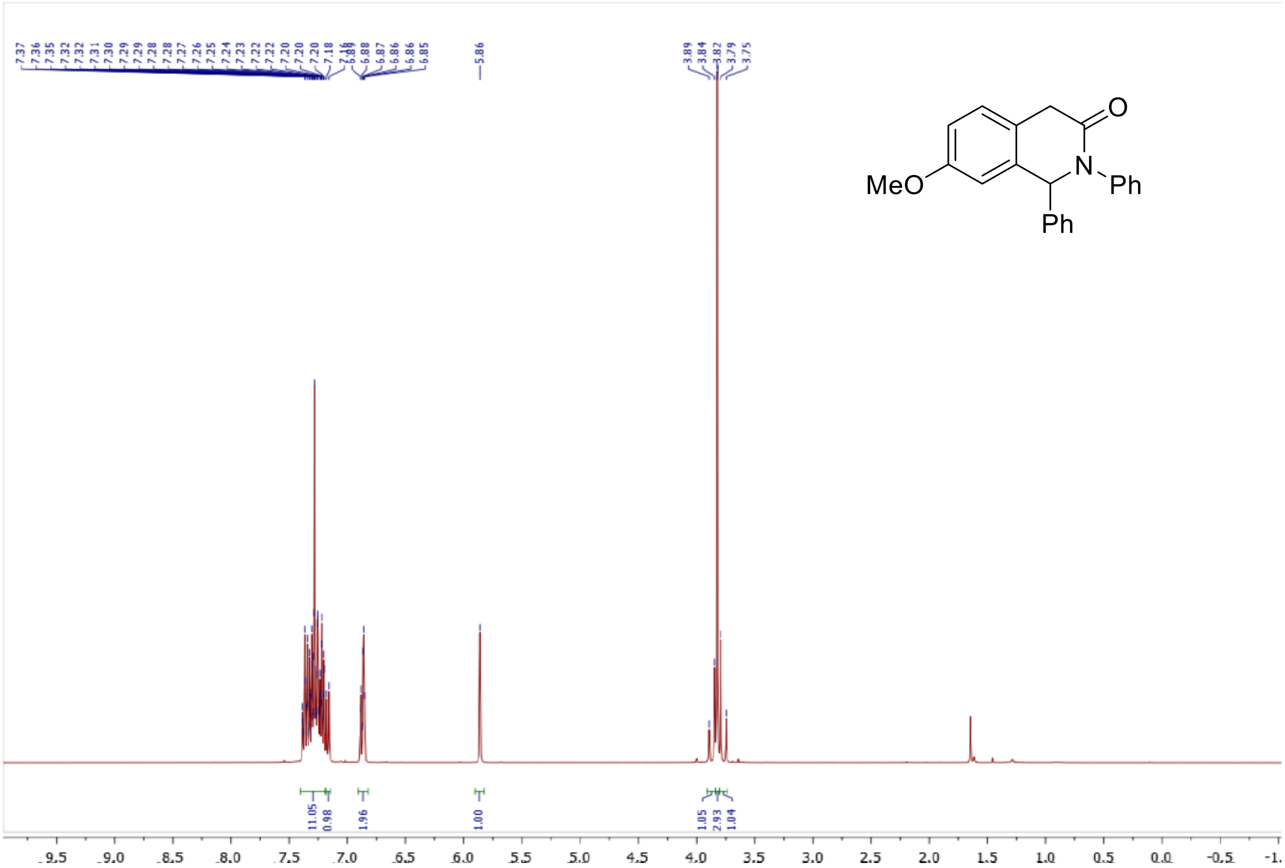

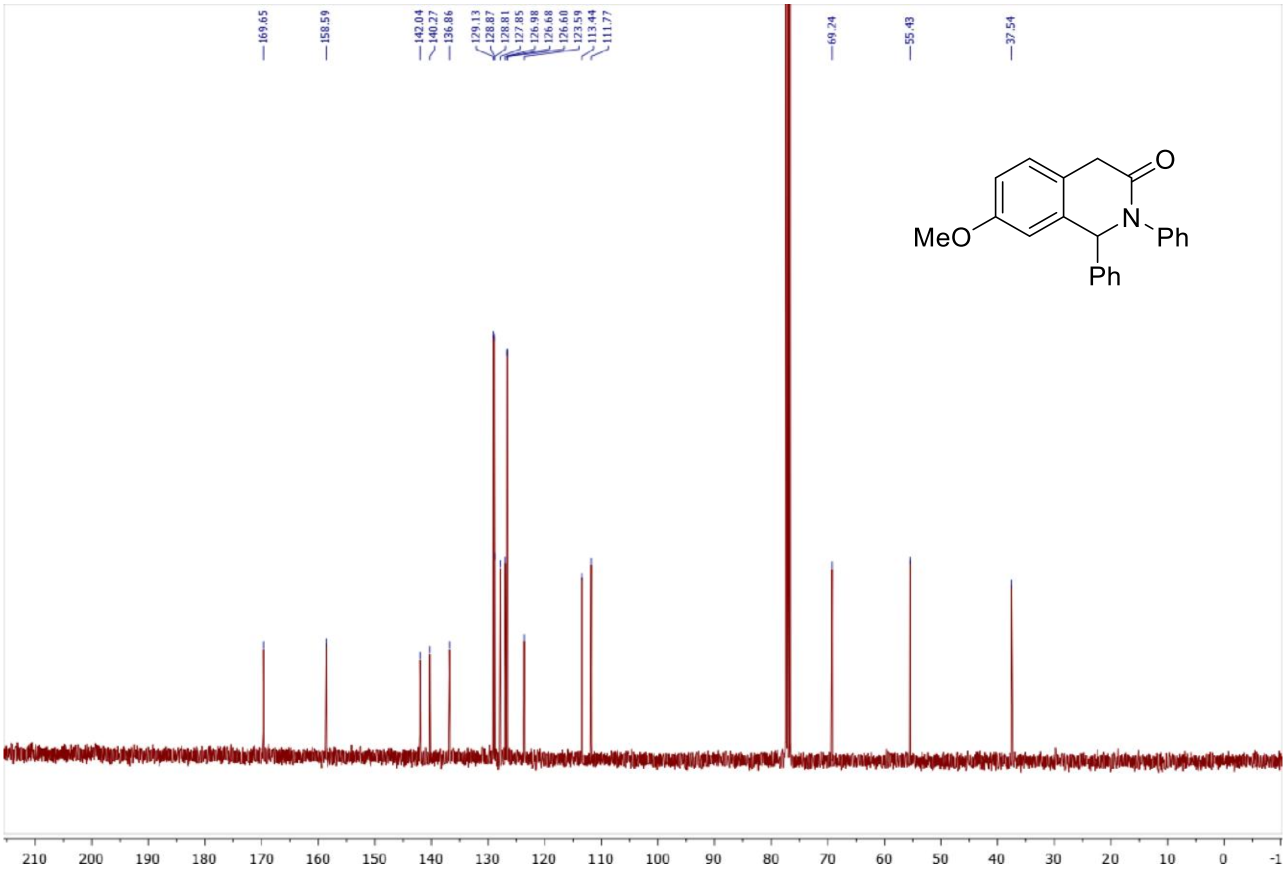

Copies of ^{1}H (400.13 MHz, $CDCl_3$) and ^{13}C{^{1}H} (100.61 MHz, $CDCl_3$) spectra of **11f**

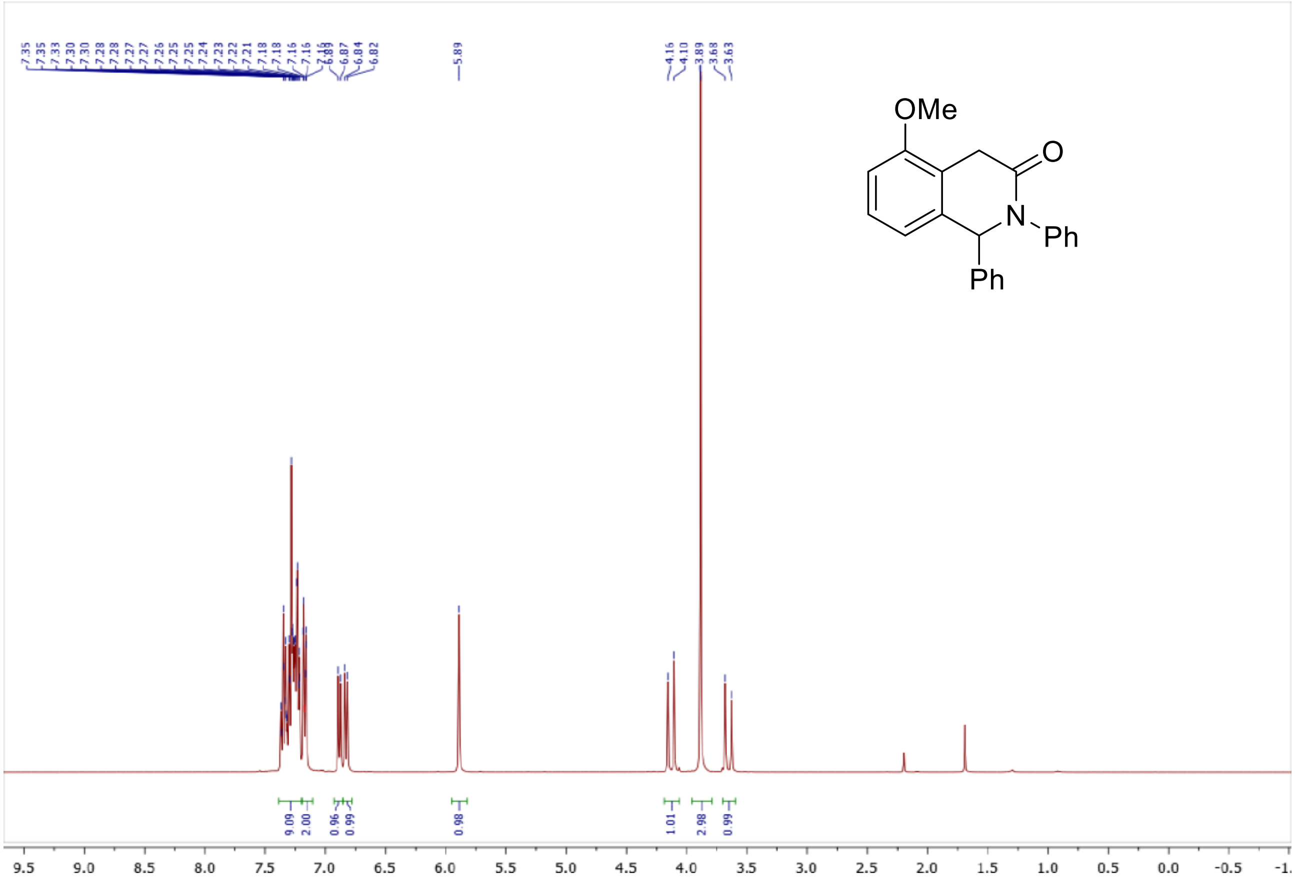

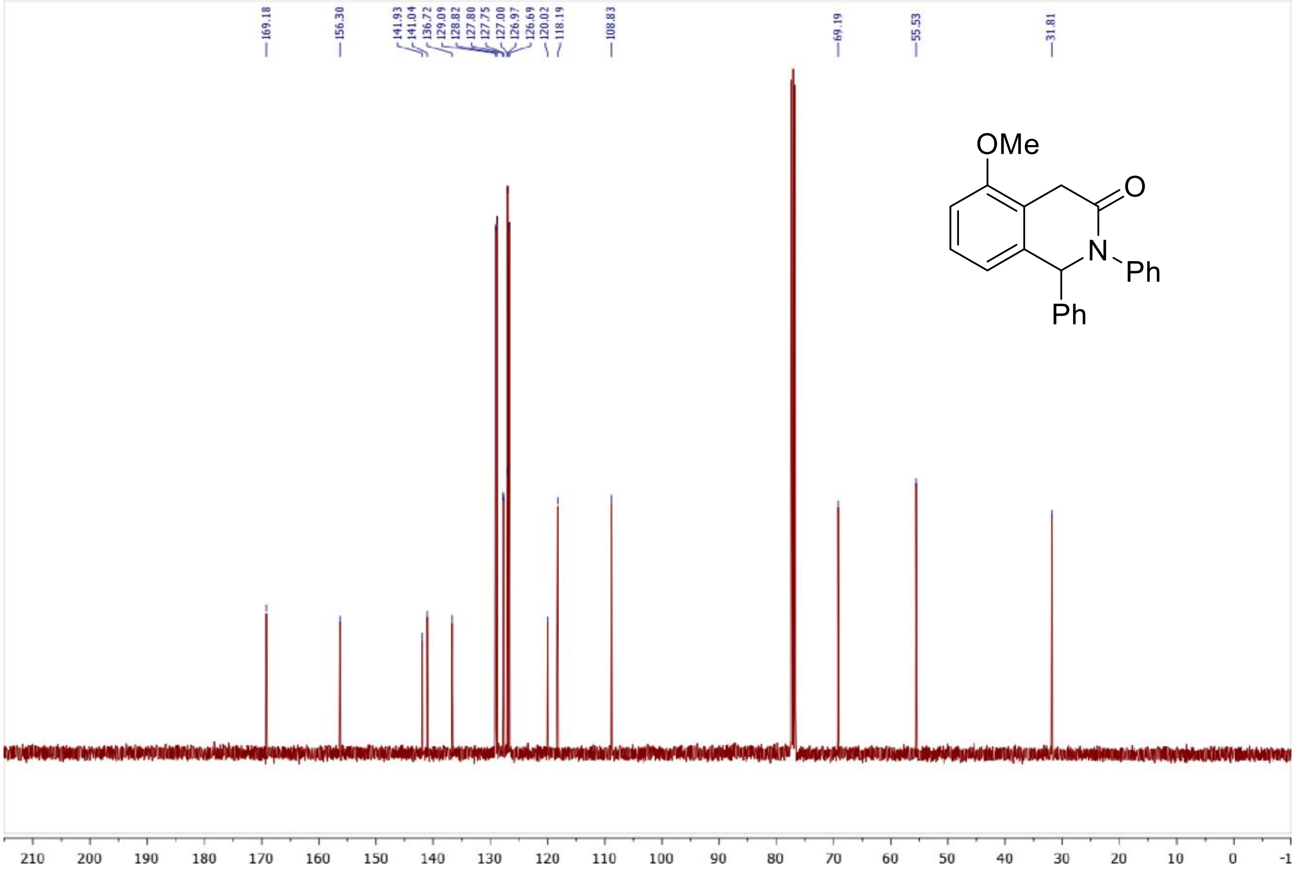

Copies of ^{1}H (400.13 MHz, $CDCl_3$) and $^{13}C\{^{1}H\}$ (100.61 MHz, $CDCl_3$) spectra of **11h**

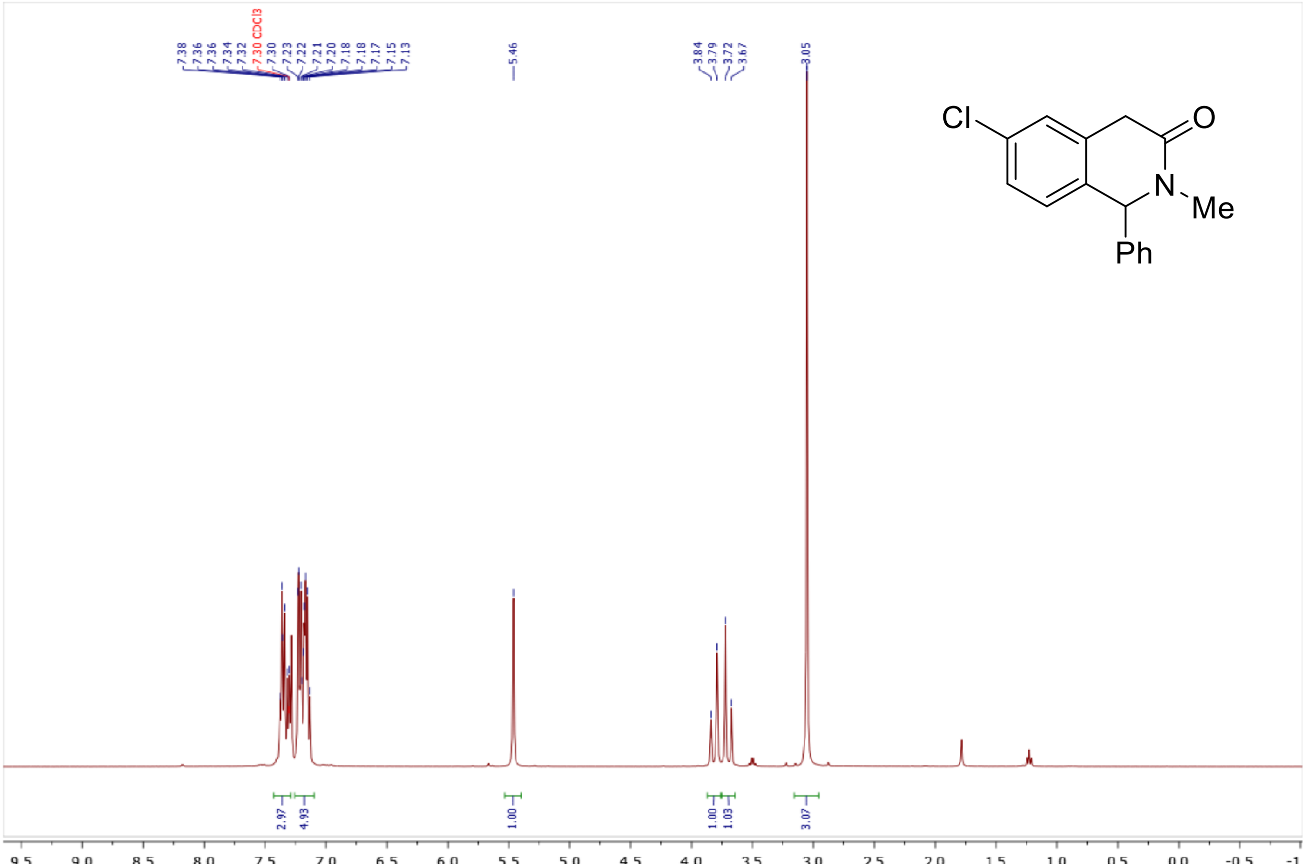

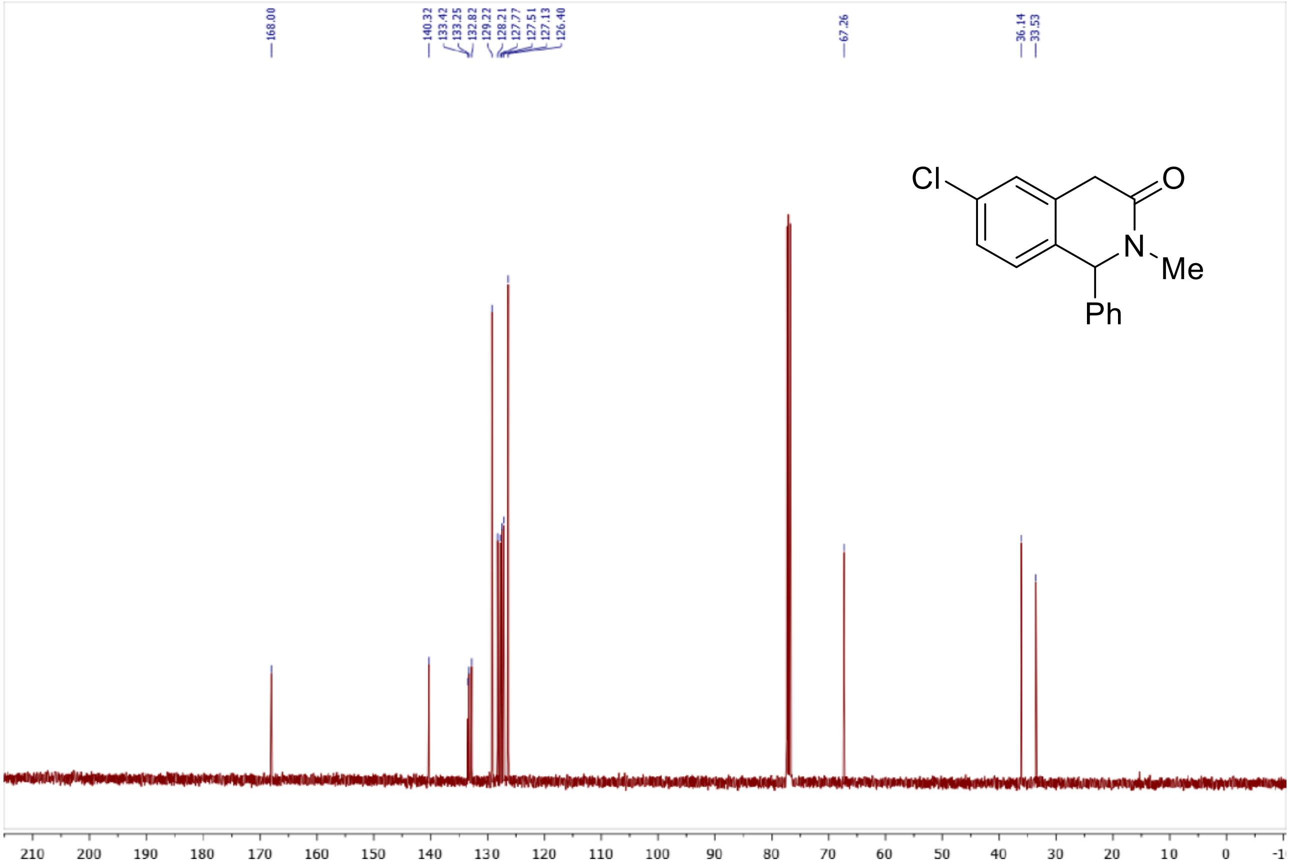

Copies of ^{1}H (400.13 MHz, $CDCl_3$) and $^{13}C\{^{1}H\}$ (100.61 MHz, $CDCl_3$) spectra of **11i**

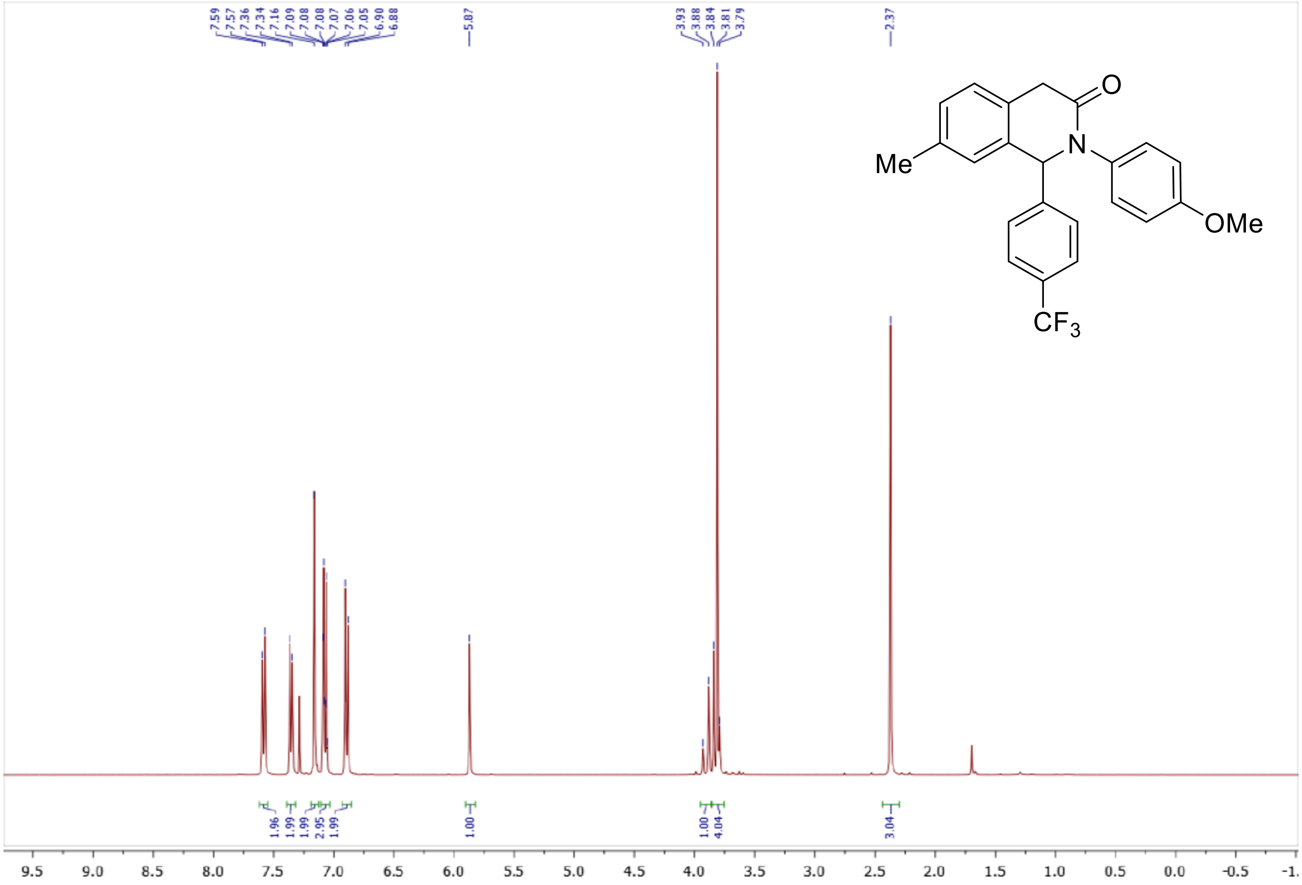

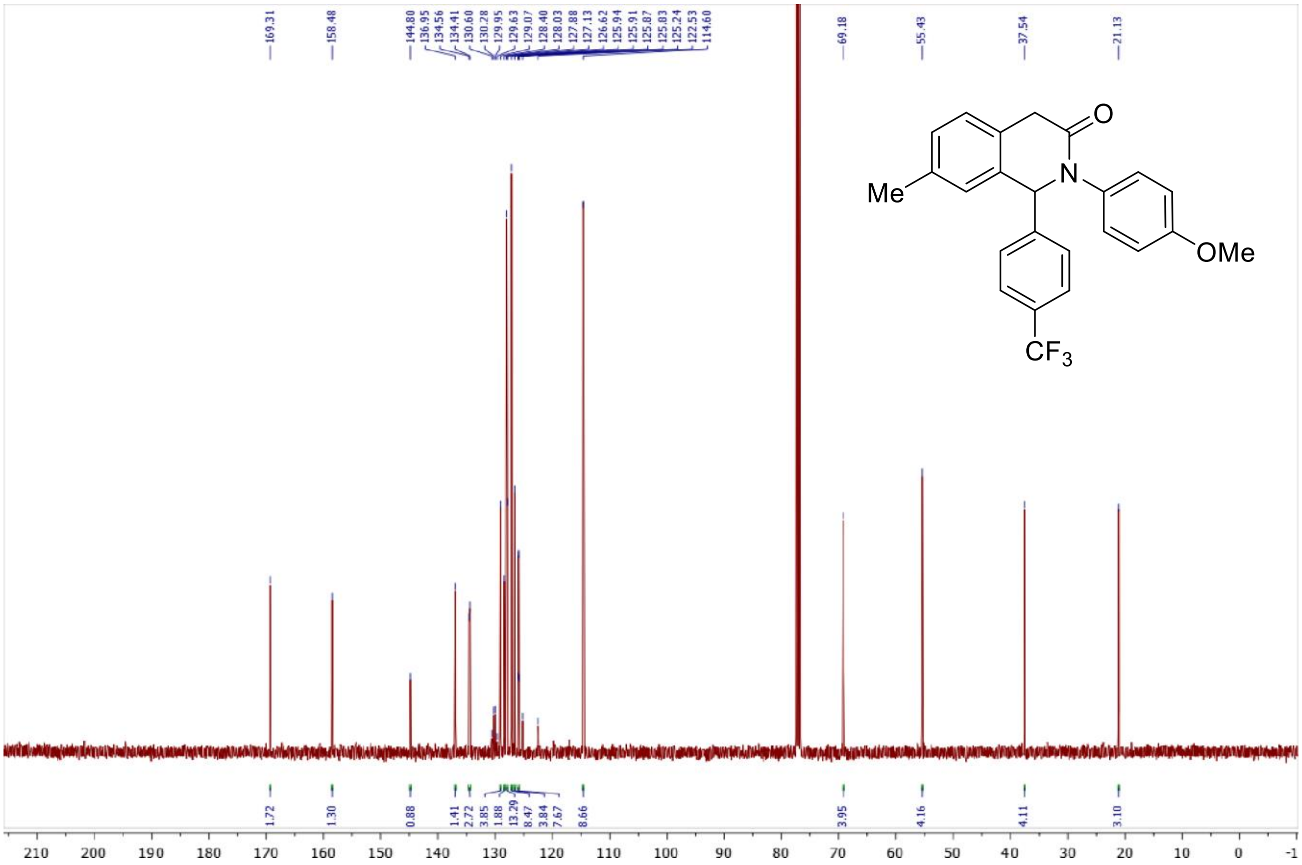

Copy of $^{19}F\{^{1}H\}$ (376.50 MHz, $CDCl_3$) spectrum of **11i**

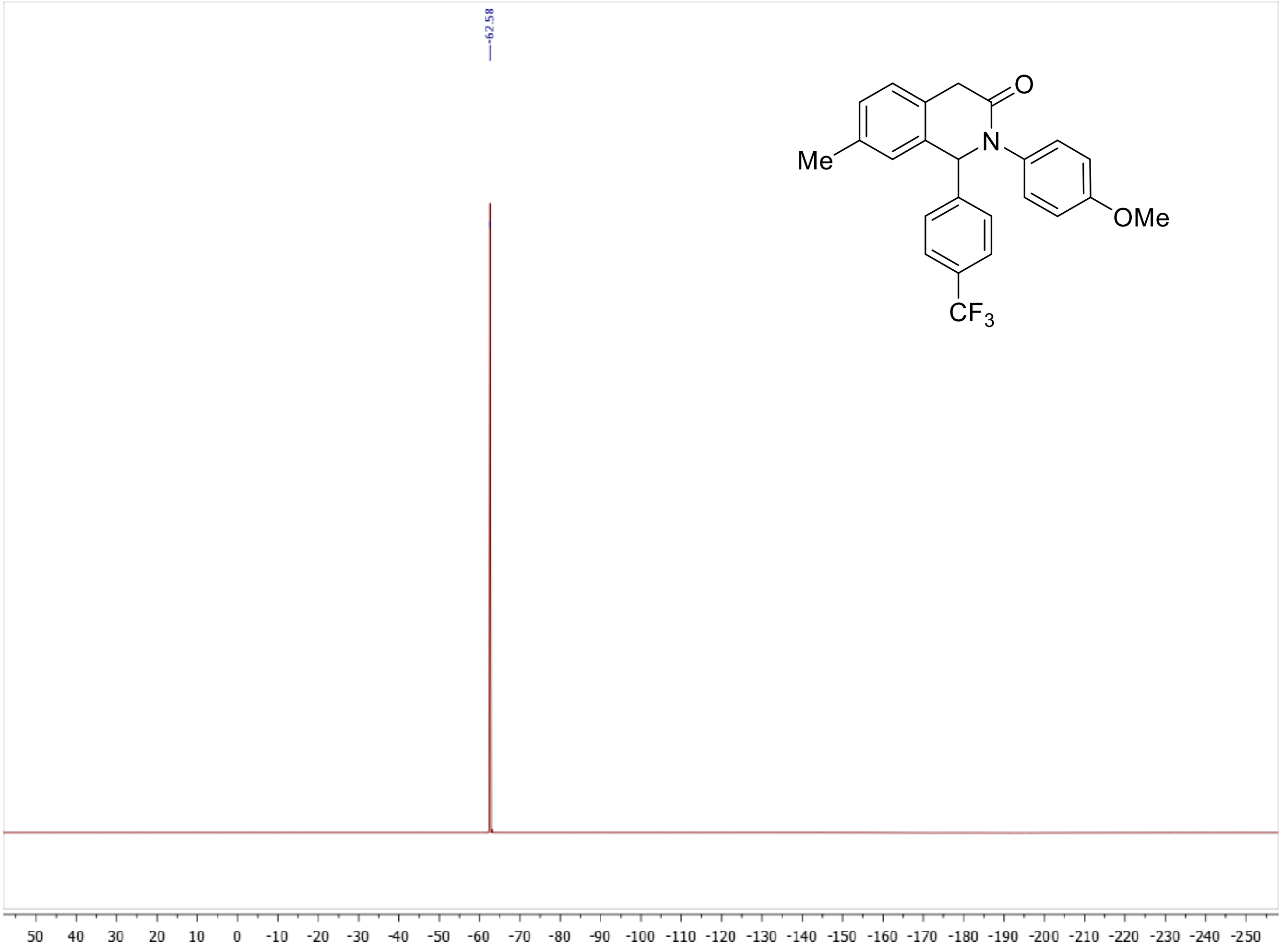

Copies of ^{1}H (400.13 MHz, $CDCl_3$) and ^{13}C{^{1}H} (100.61 MHz, $CDCl_3$) spectra of **11j**

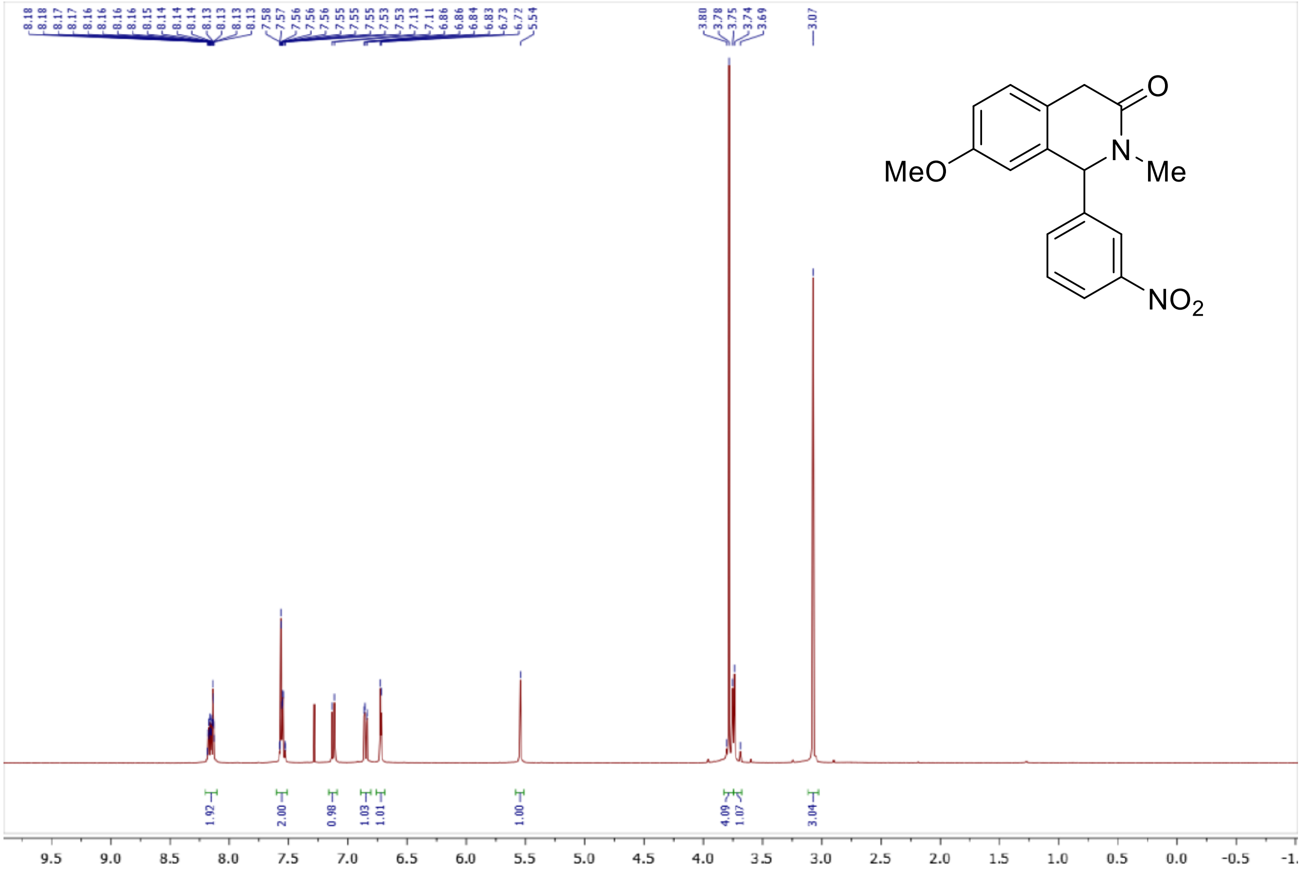

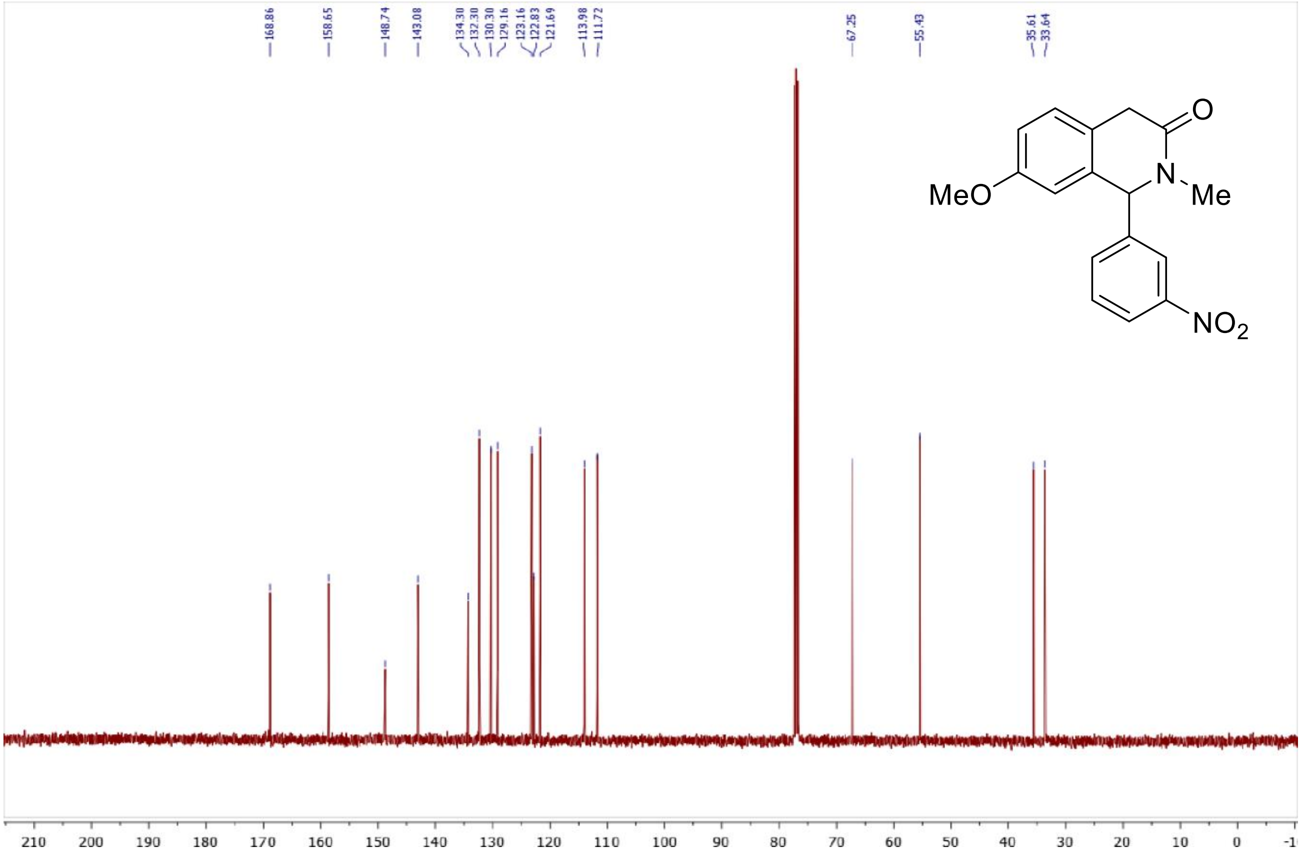

Copies of ^{1}H (400.13 MHz, $CDCl_3$) and ^{13}C{^{1}H} (100.61 MHz, $CDCl_3$) spectra of **11k**

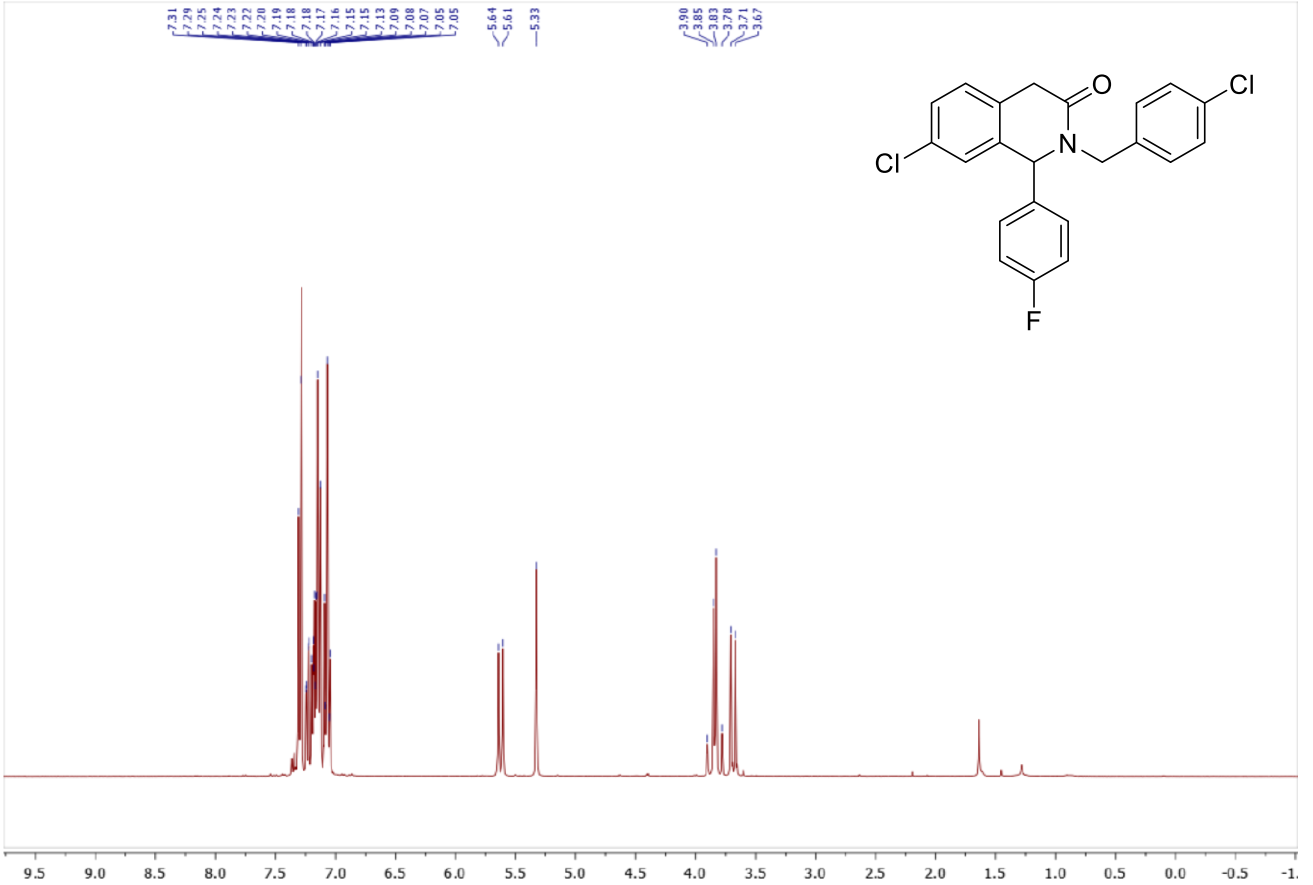

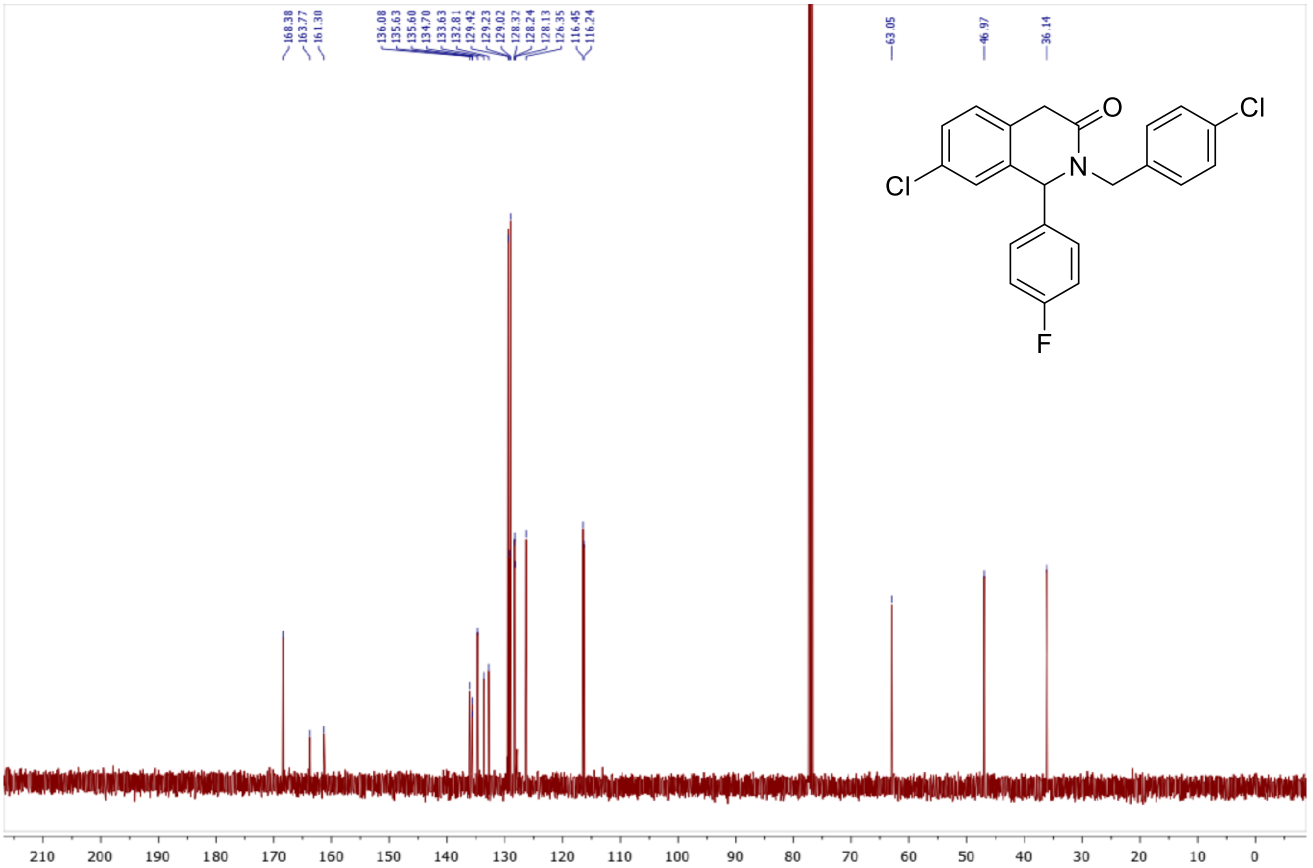

Copy of $^{19}F\{^{1}H\}$ (376.50 MHz, $CDCl_3$) spectrum of **11k**

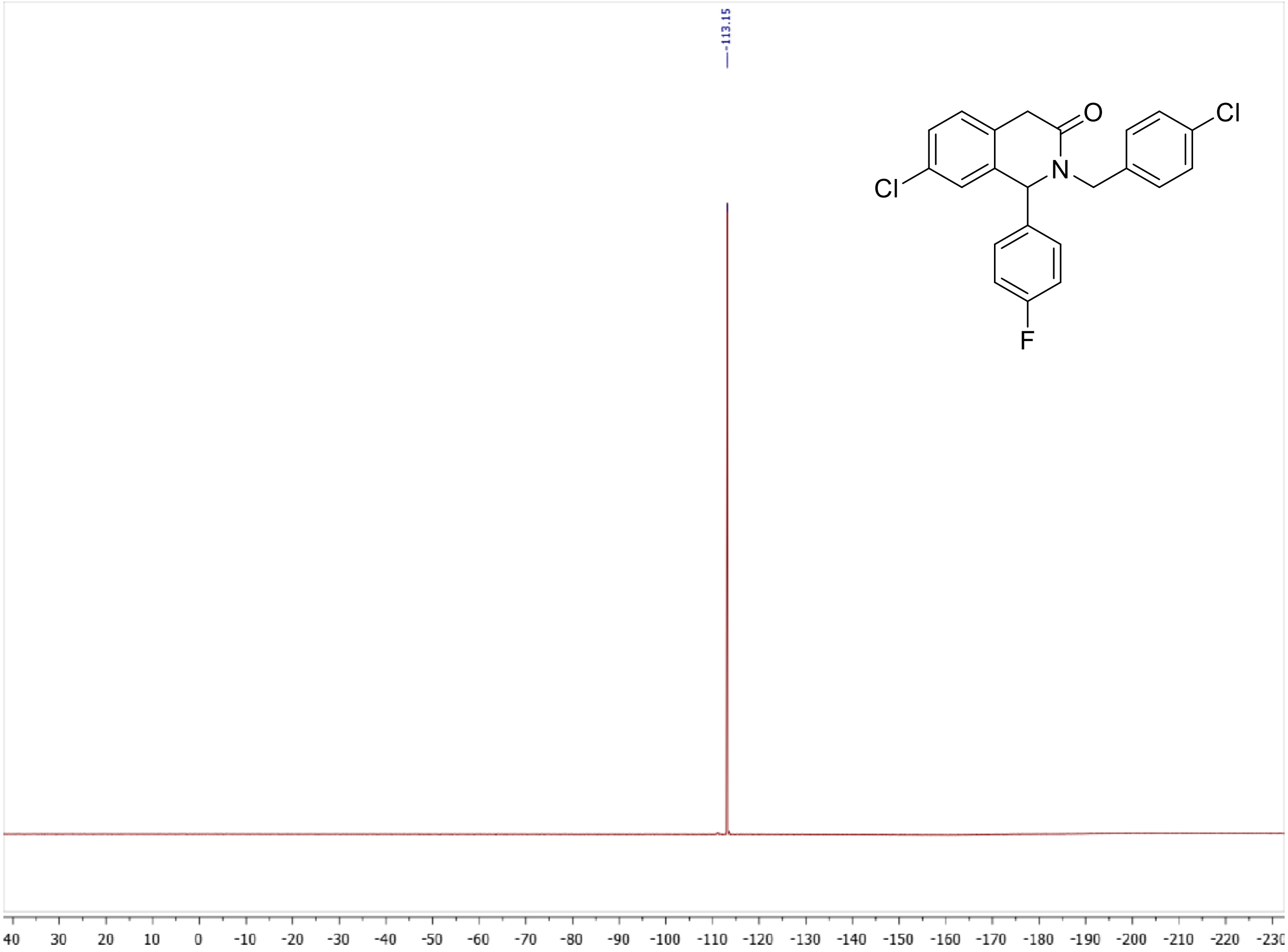

Copies of ^{1}H (400.13 MHz, $CDCl_3$) and $^{13}C\{^{1}H\}$ (100.61 MHz, $CDCl_3$) spectra of **11l**

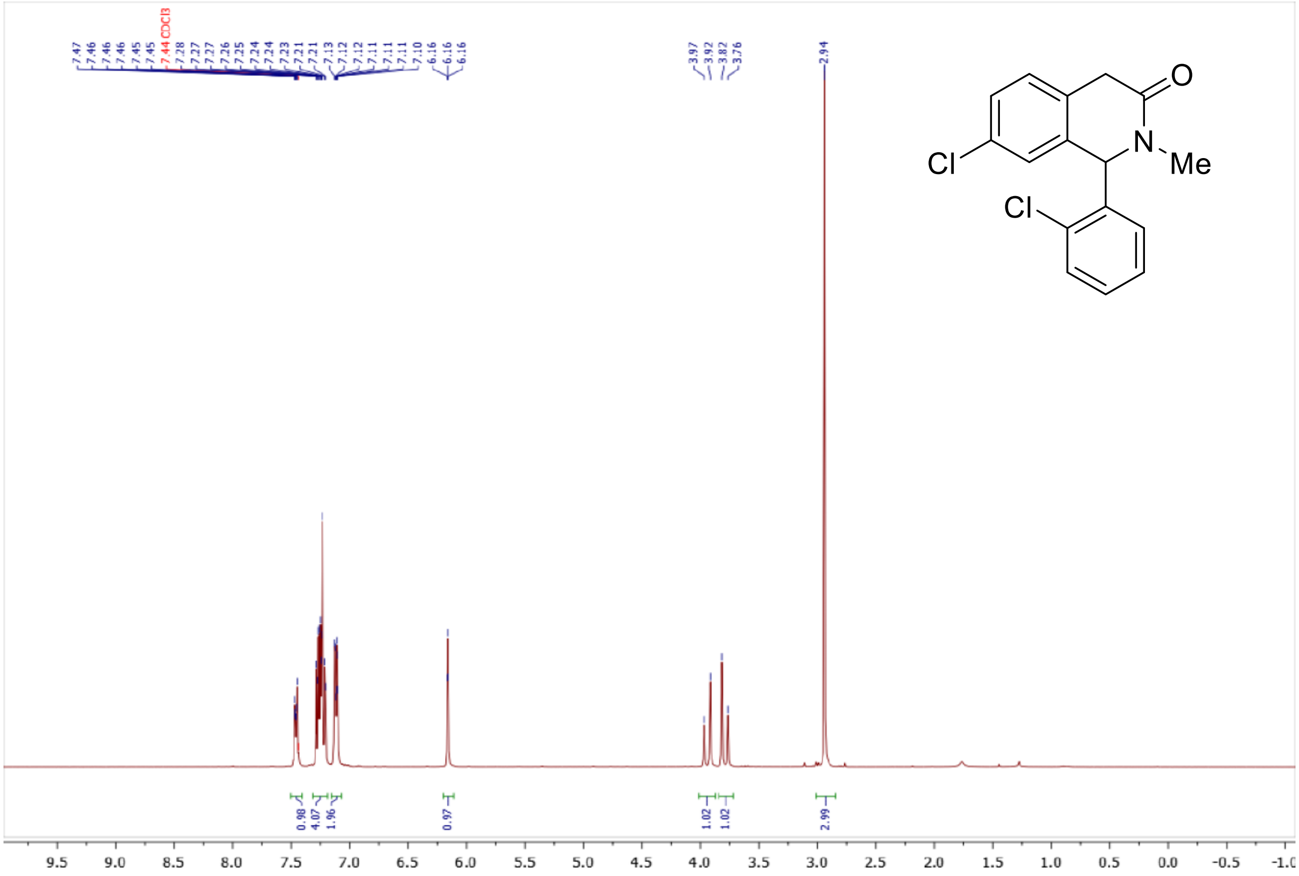

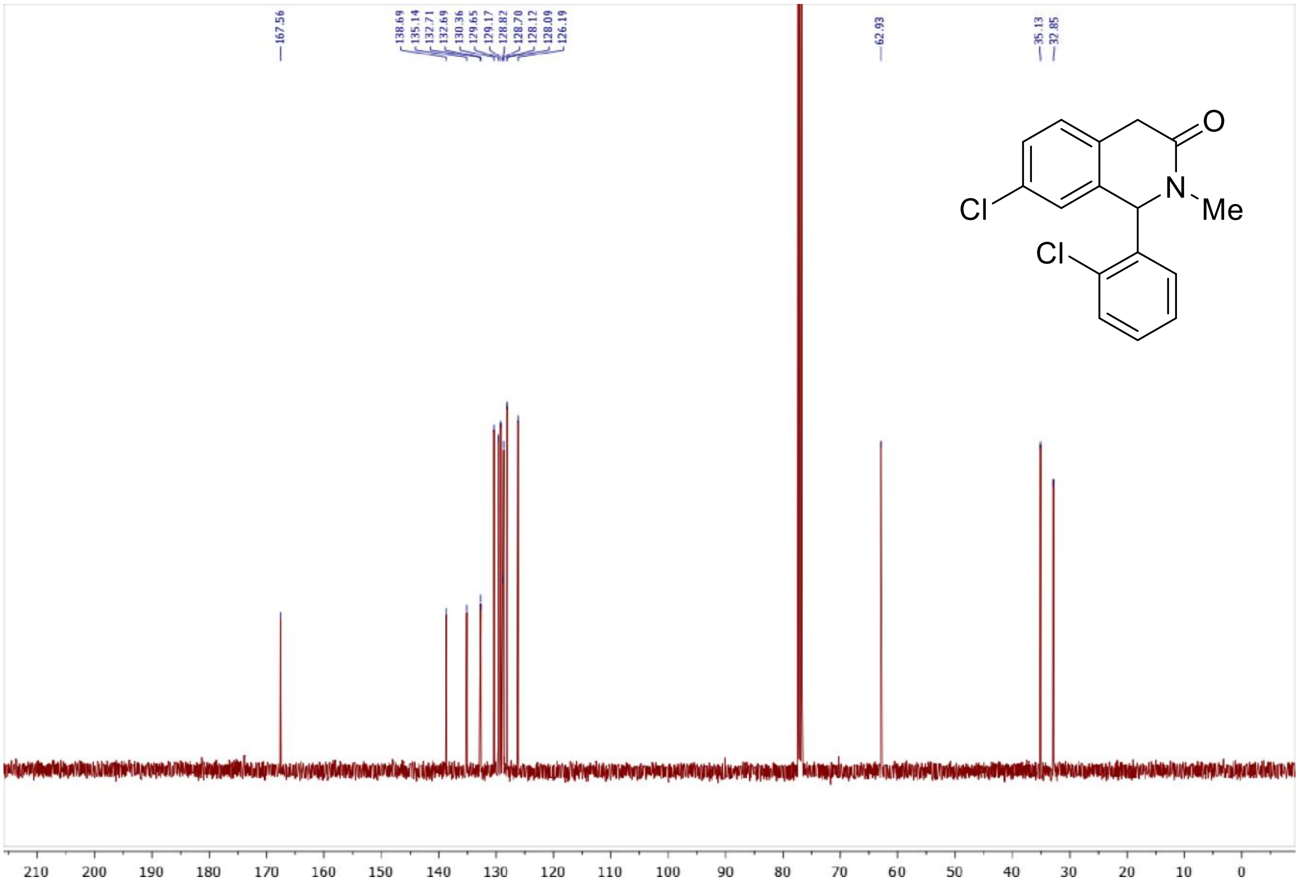

Copies of ^{1}H (400.13 MHz, $CDCl_3$) and $^{13}C\{^{1}H\}$ (100.61 MHz, $CDCl_3$) spectra of **11m**

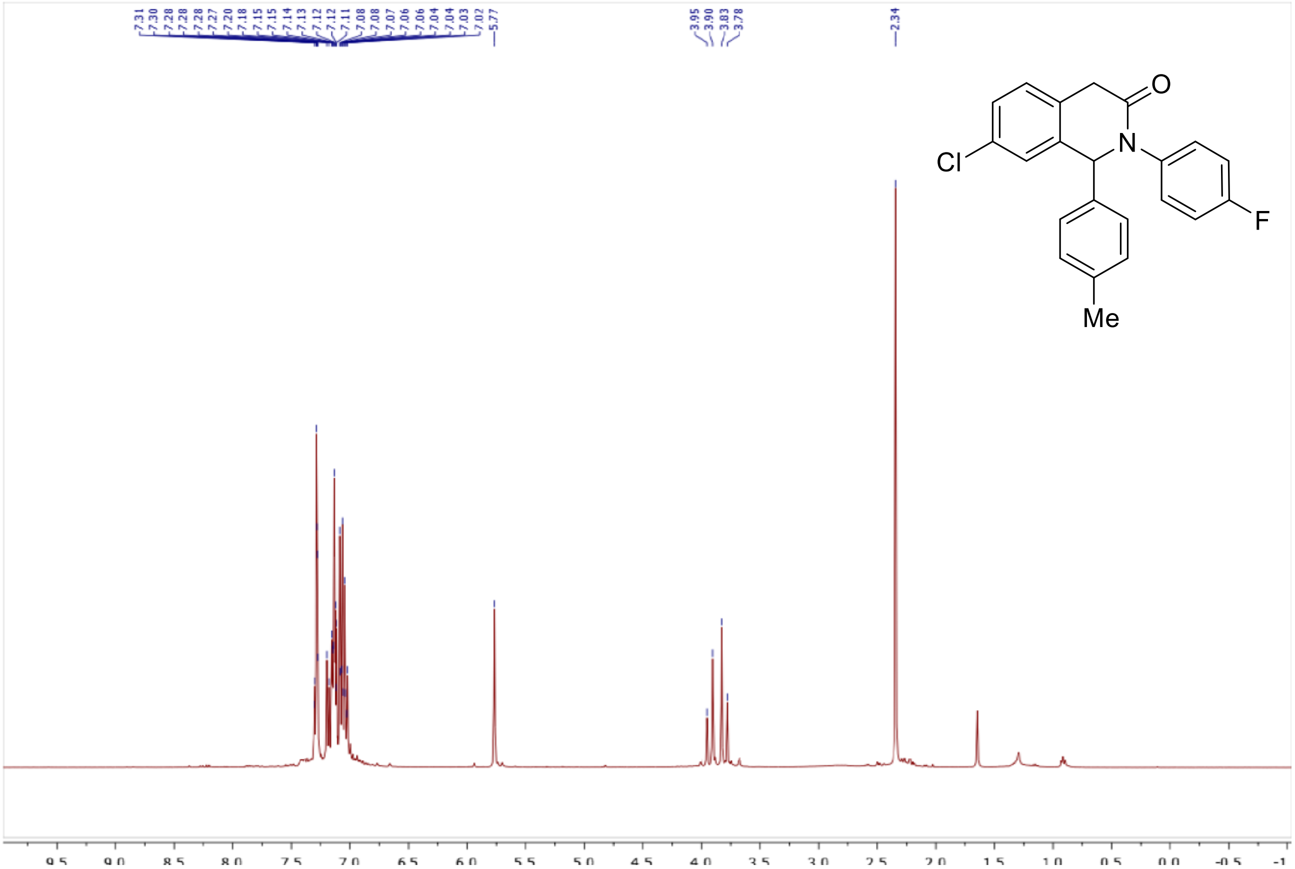

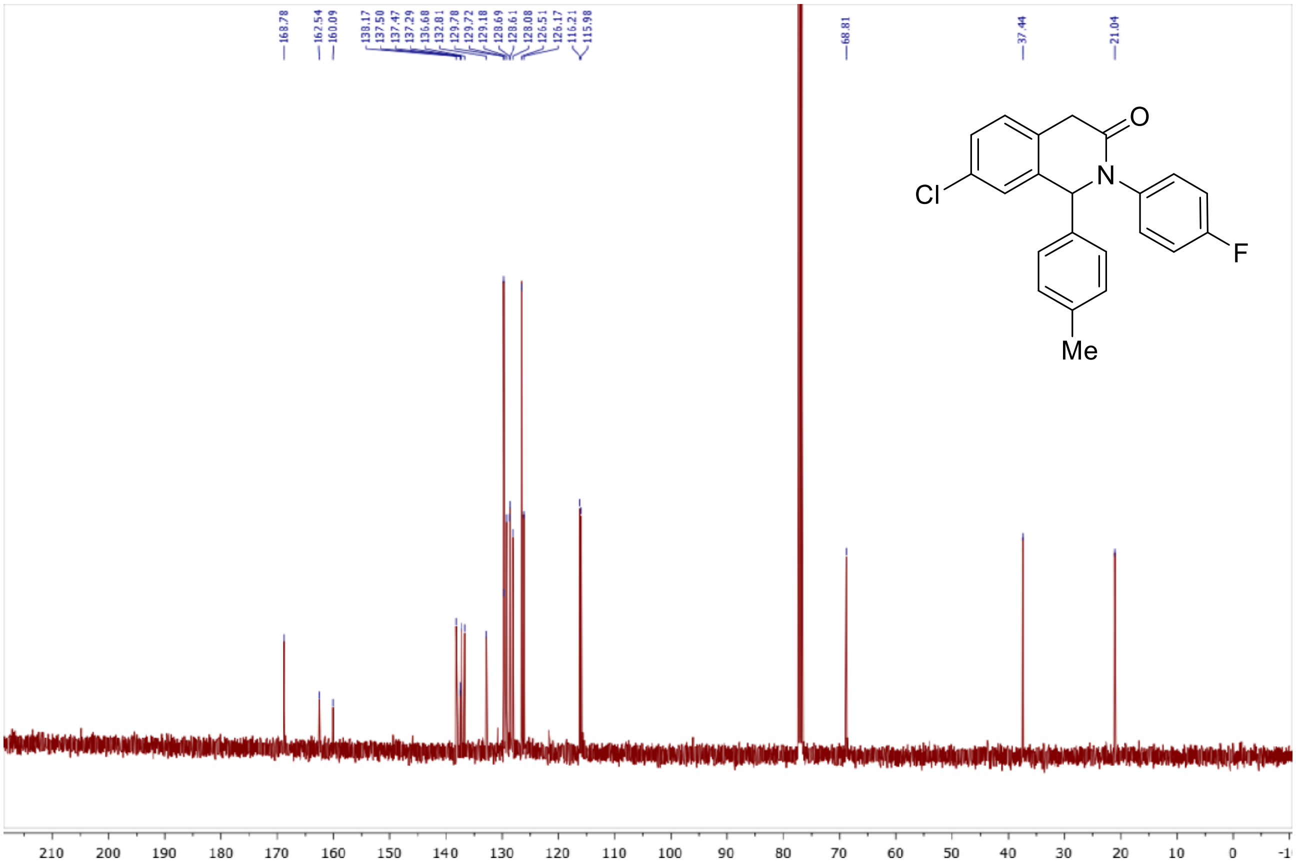

Copy of $^{19}F\{^{1}H\}$ (376.50 MHz, $CDCl_3$) spectrum of **11m**

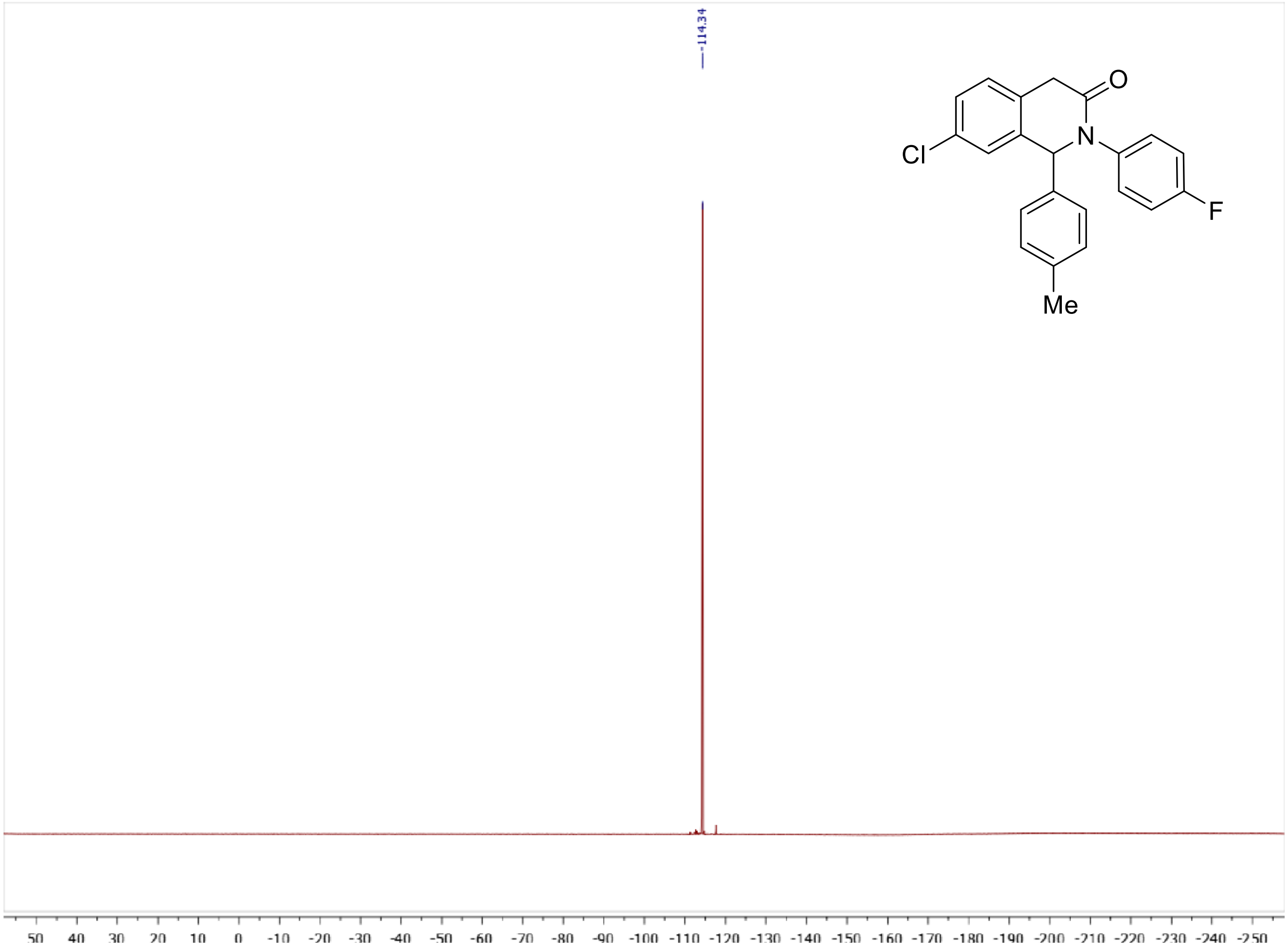

Copies of ^{1}H (400.13 MHz, CDCl$_3$) and ^{13}C{^{1}H} (100.61 MHz, CDCl$_3$) spectra of **11n**

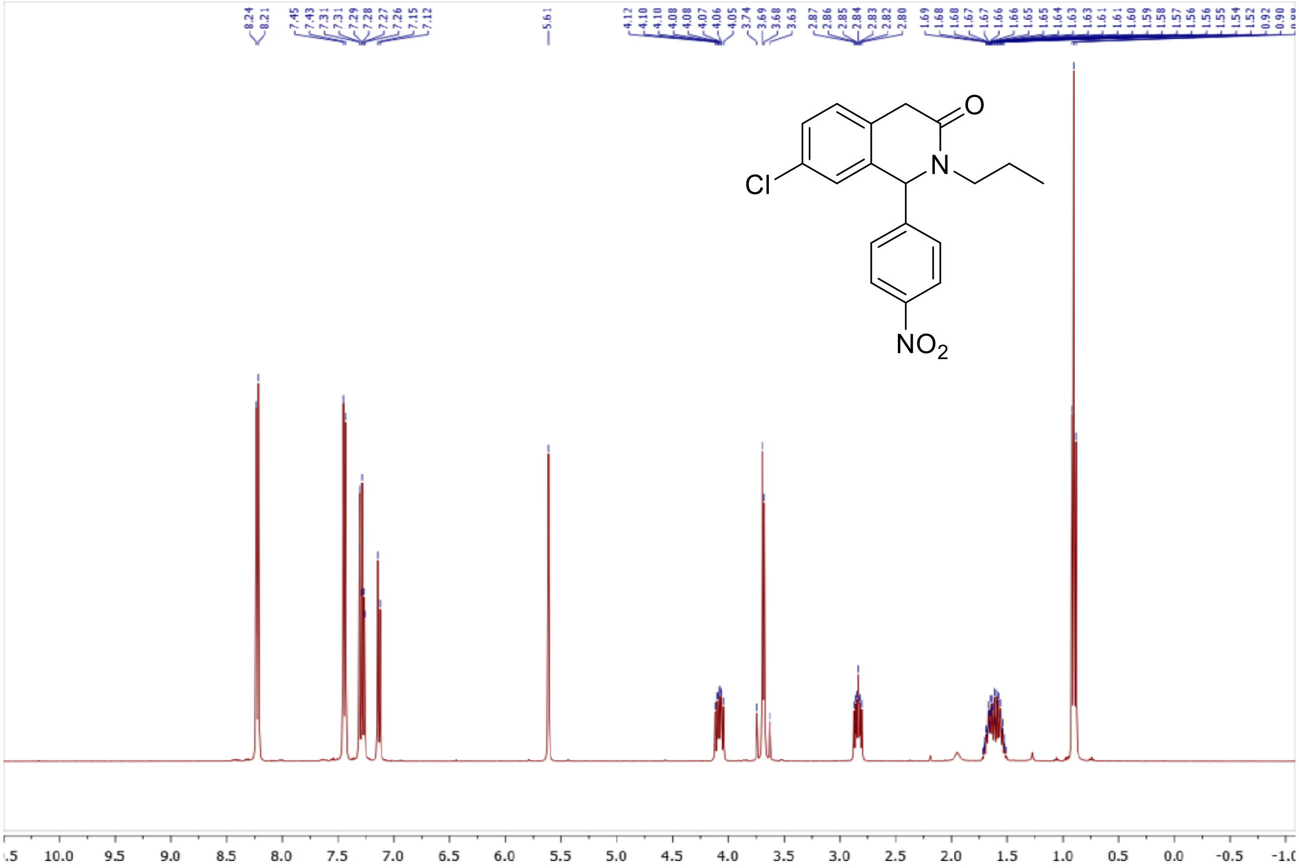

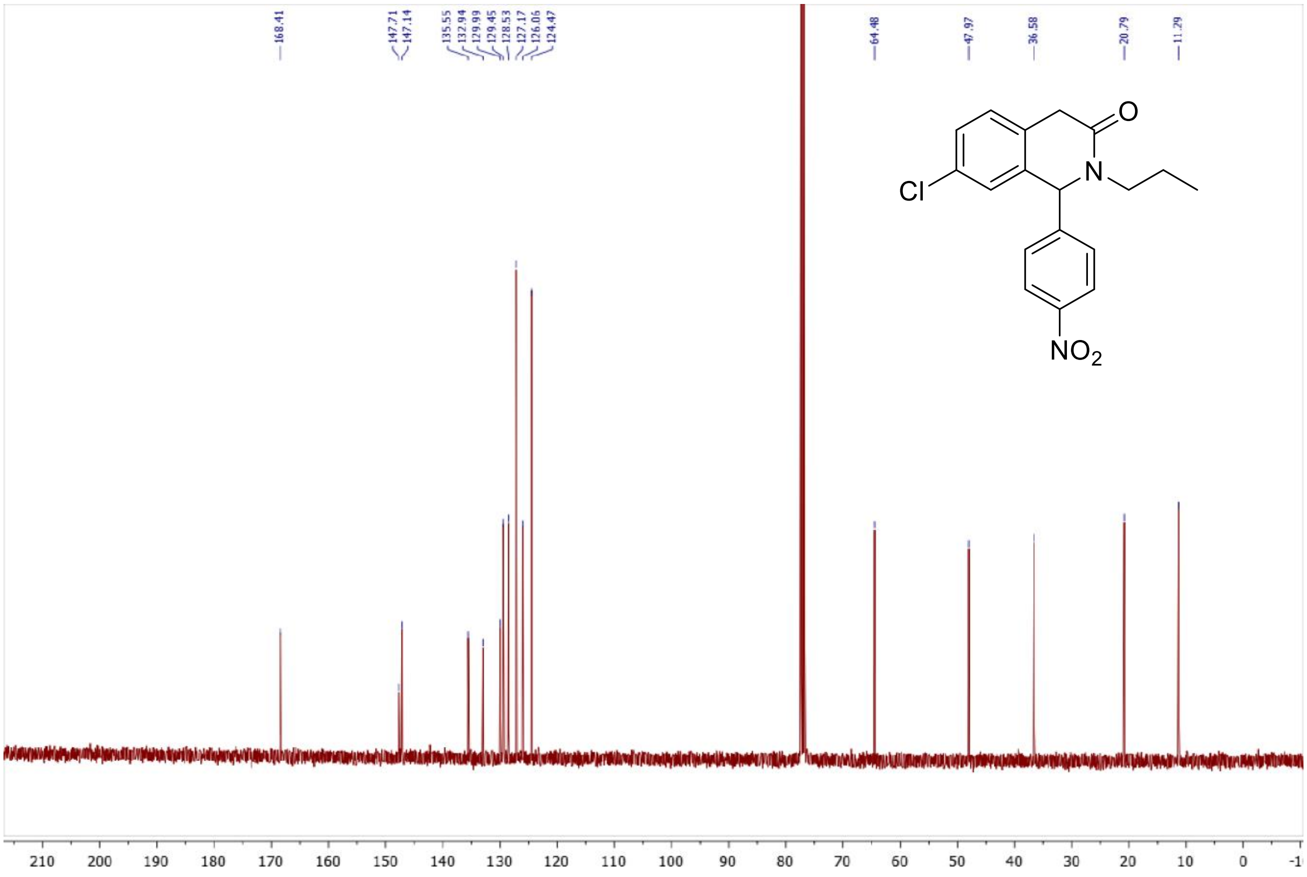

Copies of ^{1}H (400.13 MHz, $CDCl_3$) and $^{13}C\{^{1}H\}$ (100.61 MHz, $CDCl_3$) spectra of **11o**

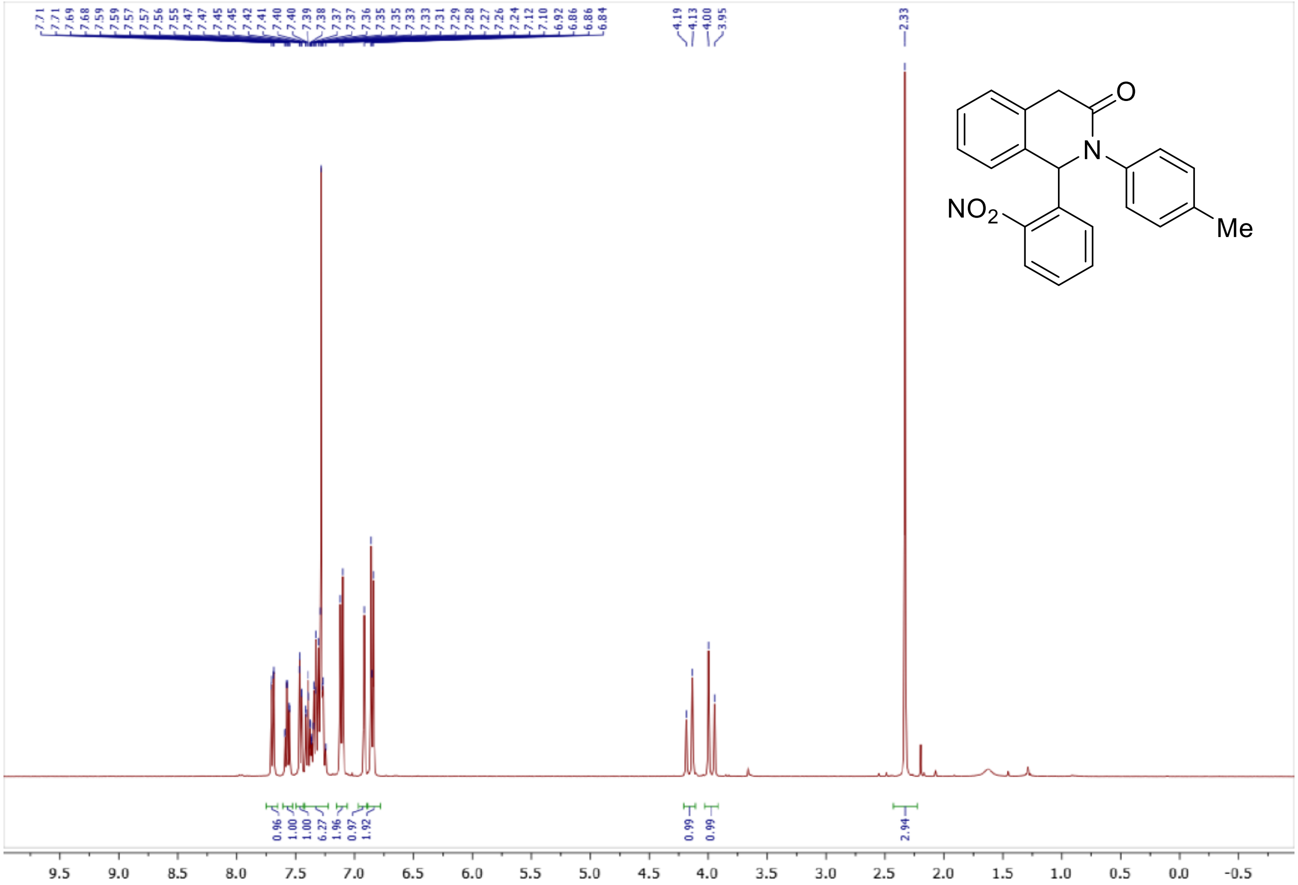

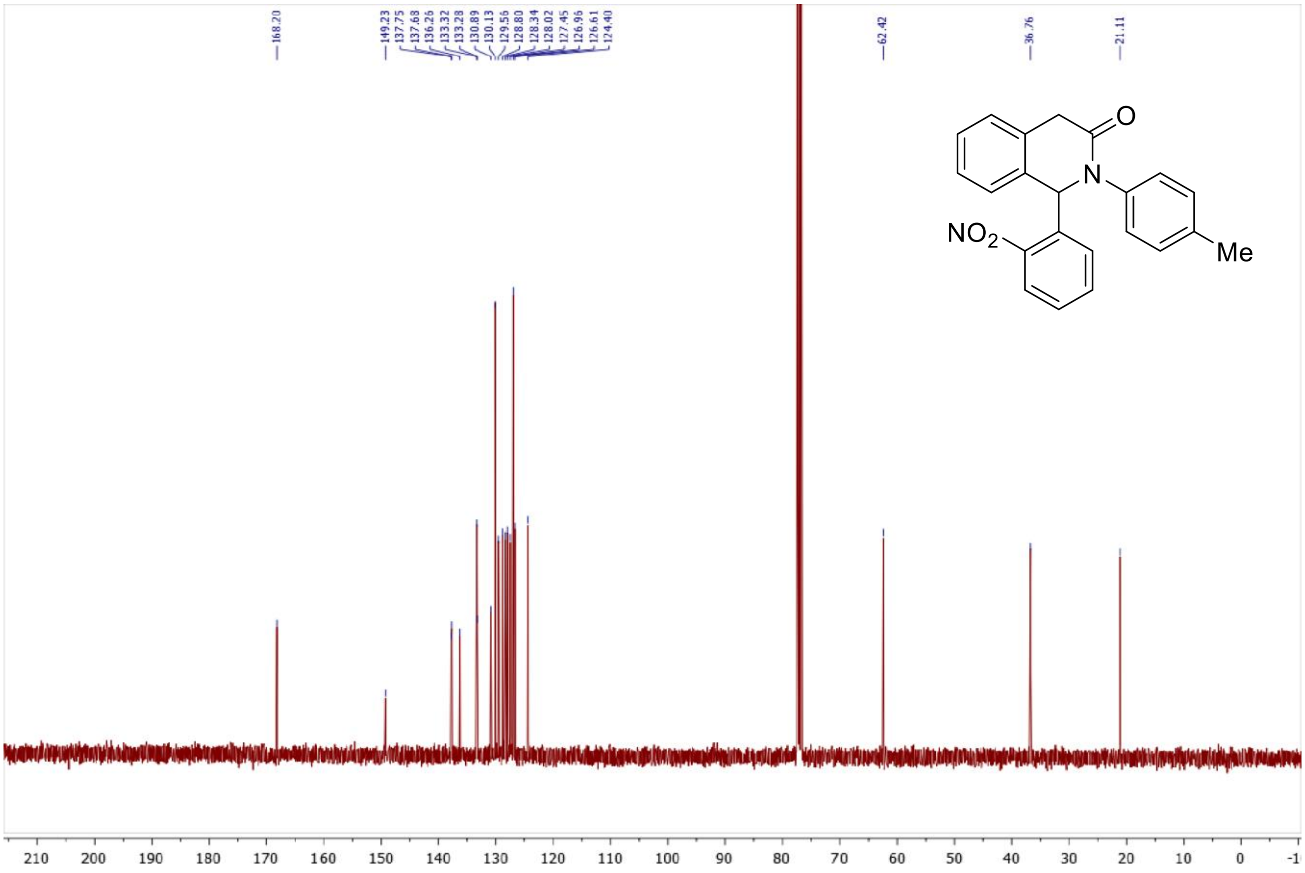

Copies of ^{1}H (400.13 MHz, $CDCl_3$) and $^{13}C\{^{1}H\}$ (100.61 MHz, $CDCl_3$) spectra of **11p**

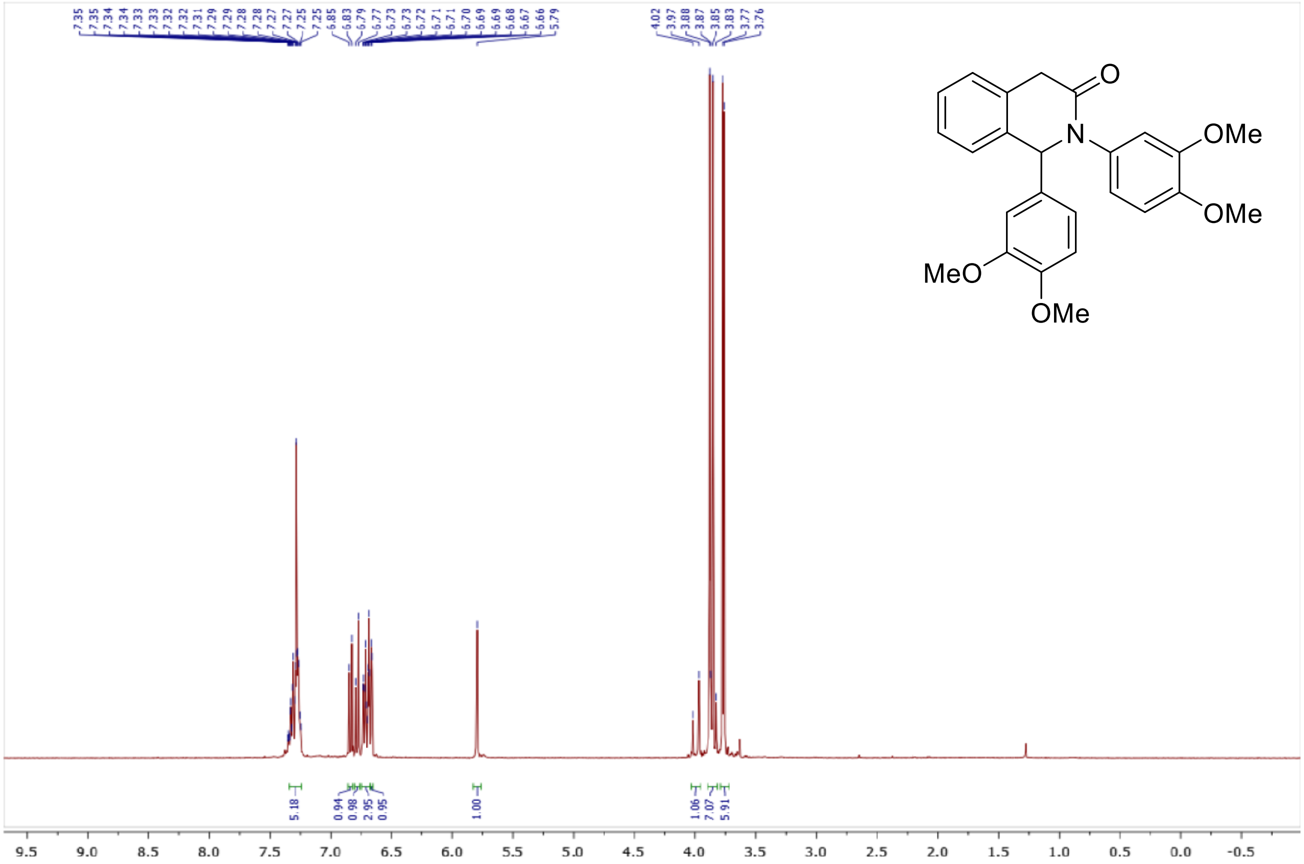

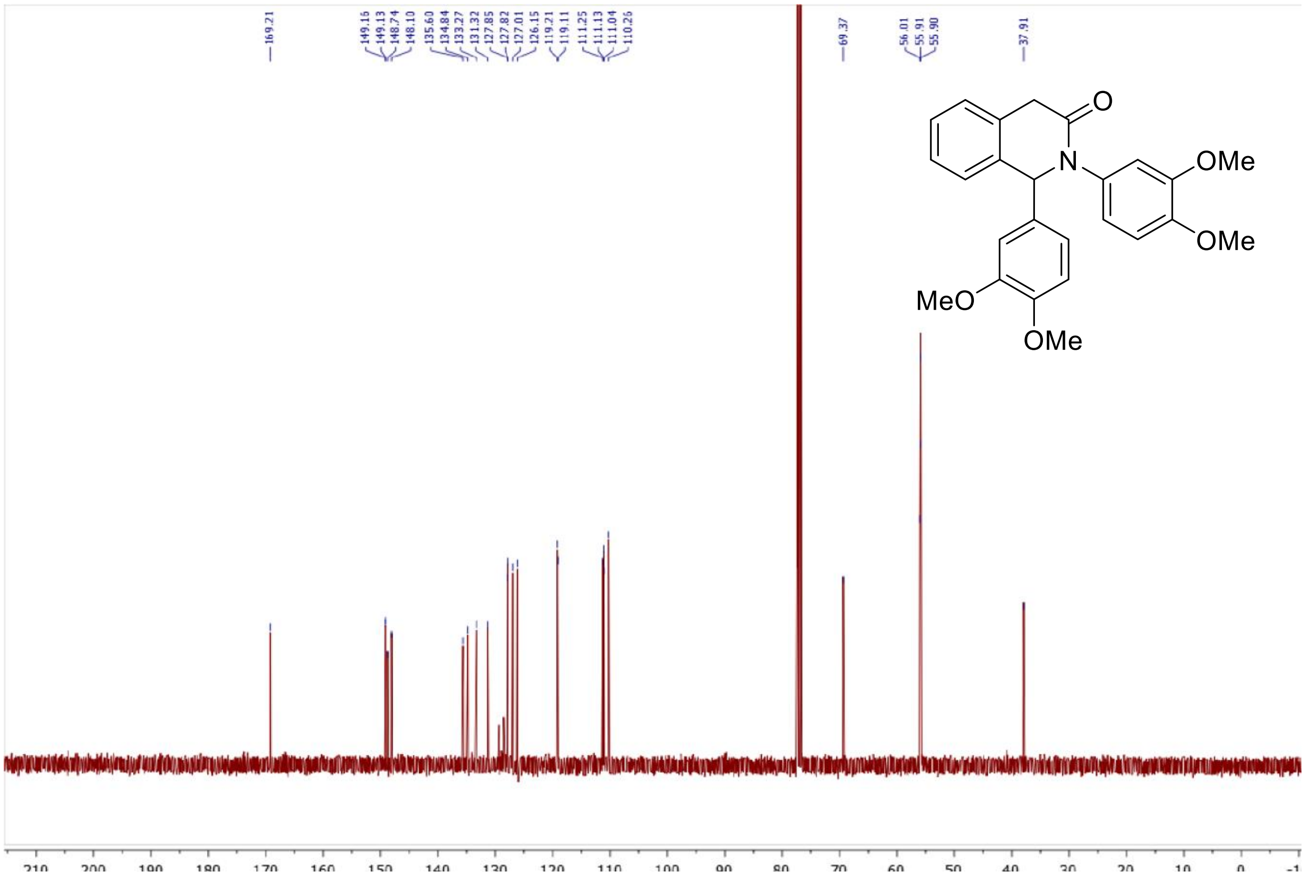

Copies of ^{1}H (400.13 MHz, $CDCl_3$) and $^{13}C\{^{1}H\}$ (100.61 MHz, $CDCl_3$) spectra of **11q**

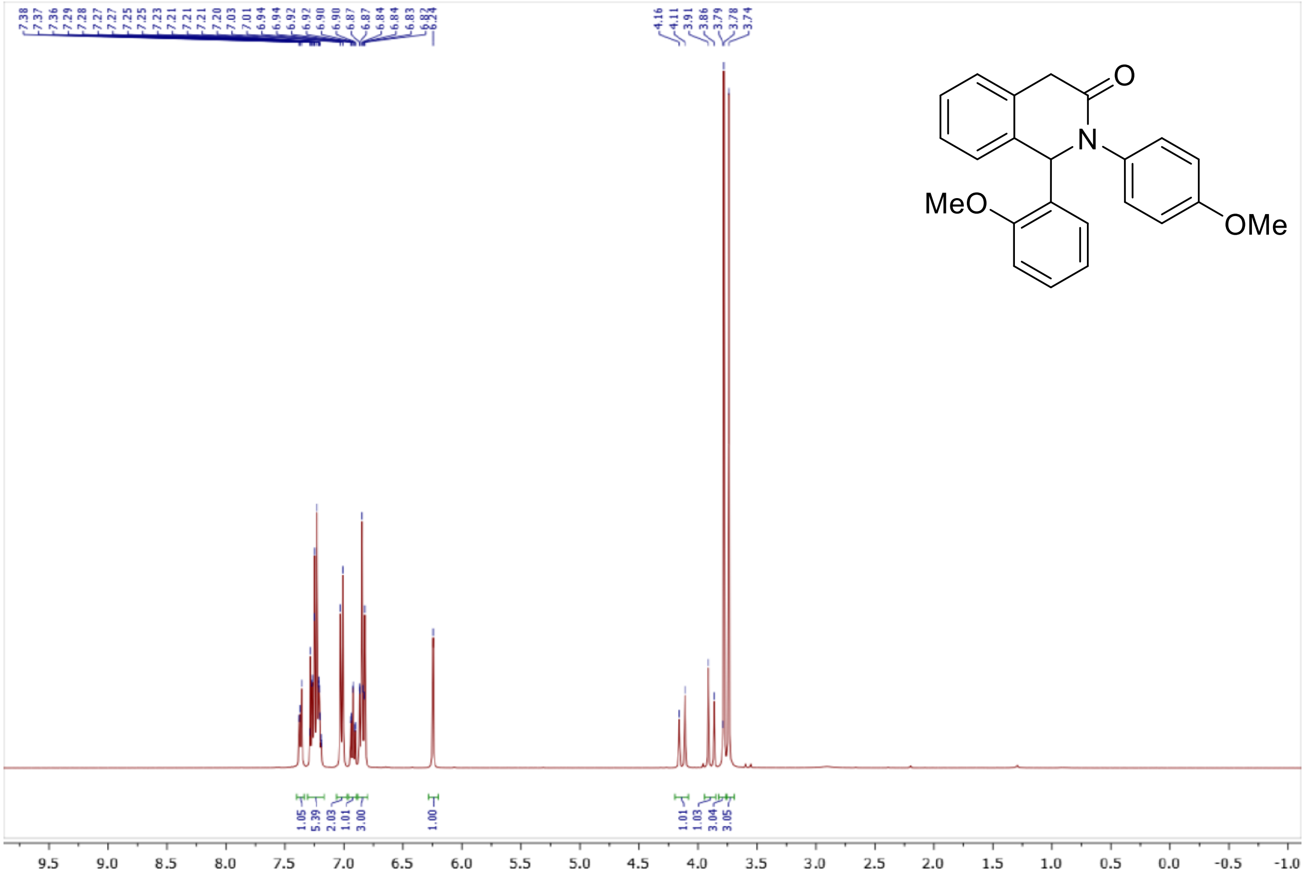

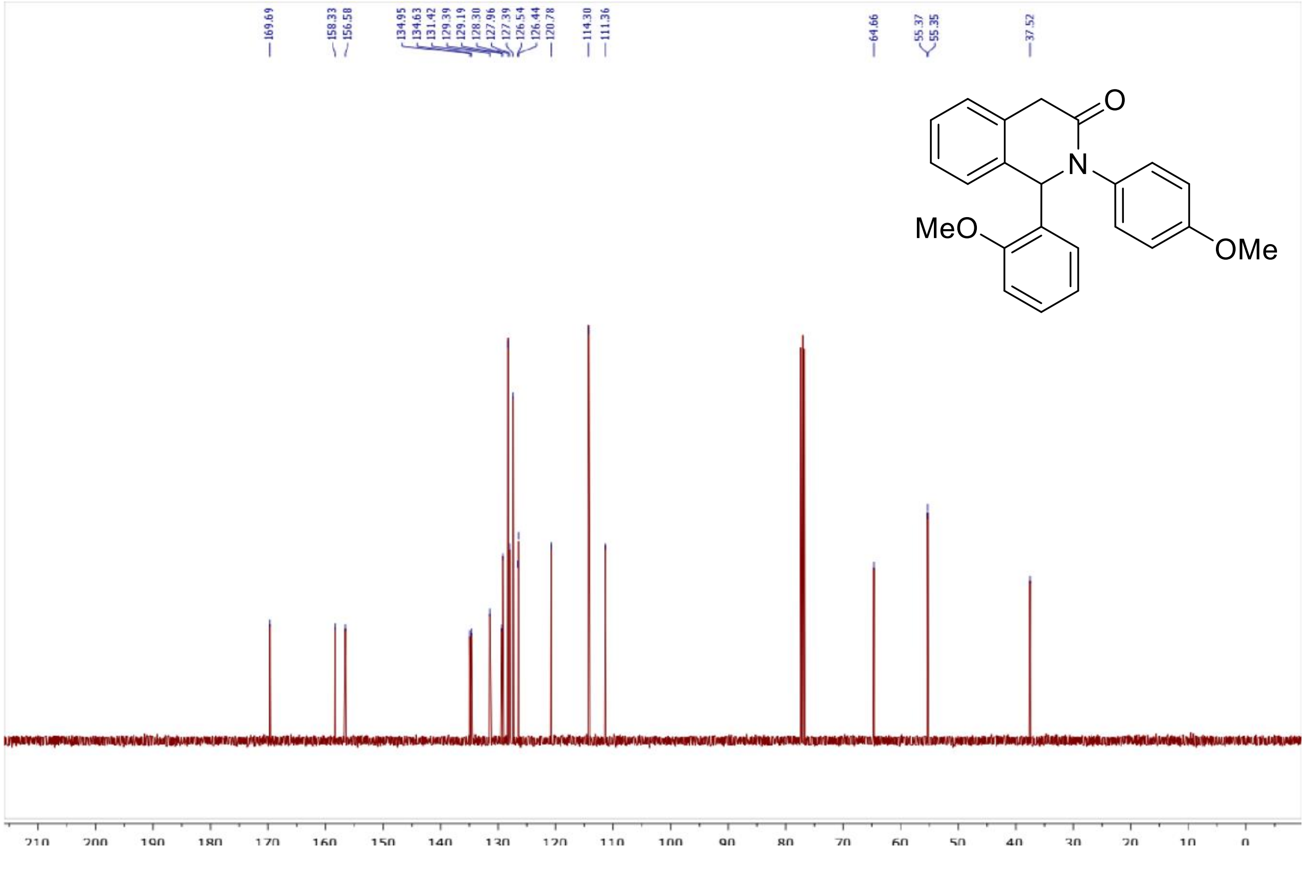

Copies of ^{1}H (400.13 MHz, $CDCl_3$) and ^{13}C{^{1}H} (100.61 MHz, $CDCl_3$) spectra of **11s**

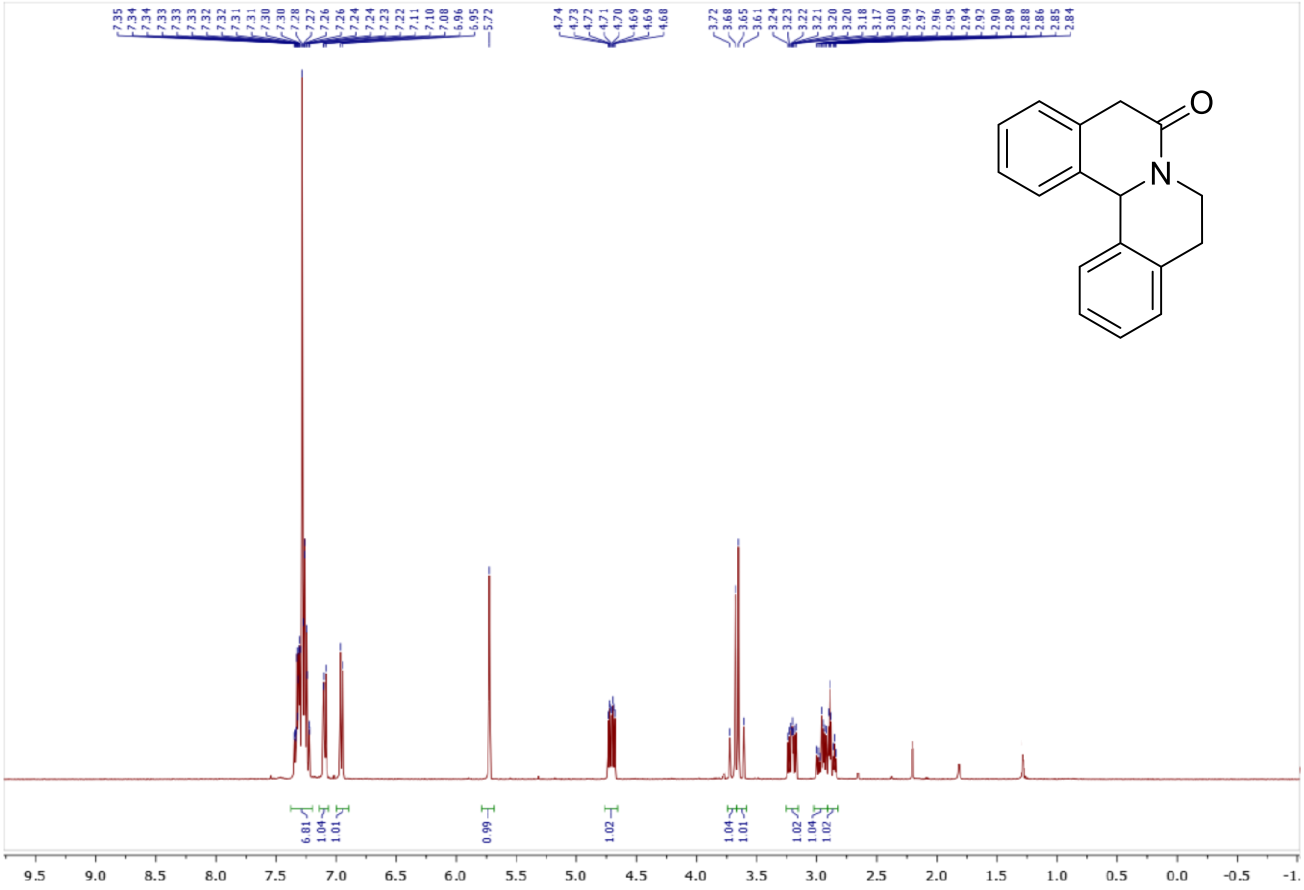

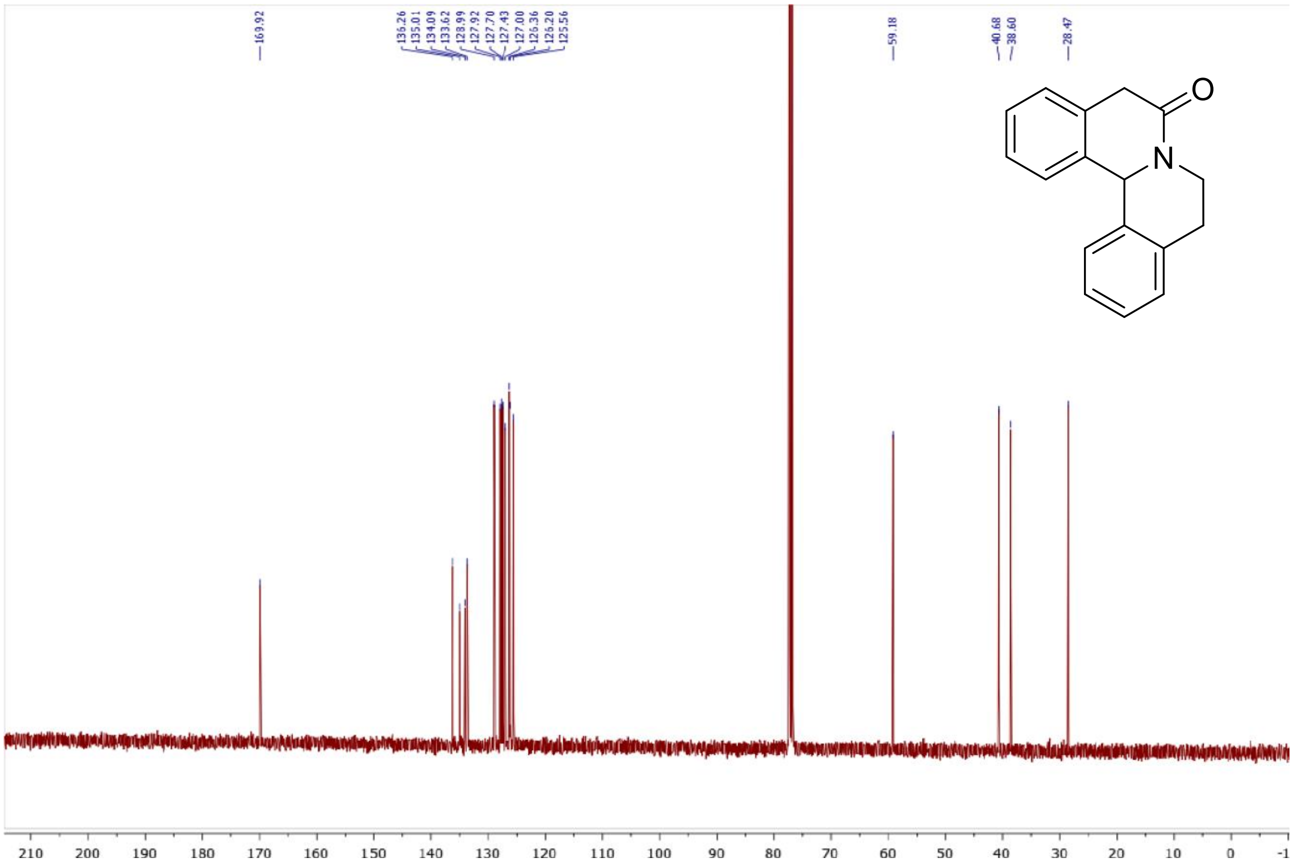

Copies of ^{1}H (400.13 MHz, $CDCl_3$) and $^{13}C\{^{1}H\}$ (100.61 MHz, $CDCl_3$) spectra of **14**

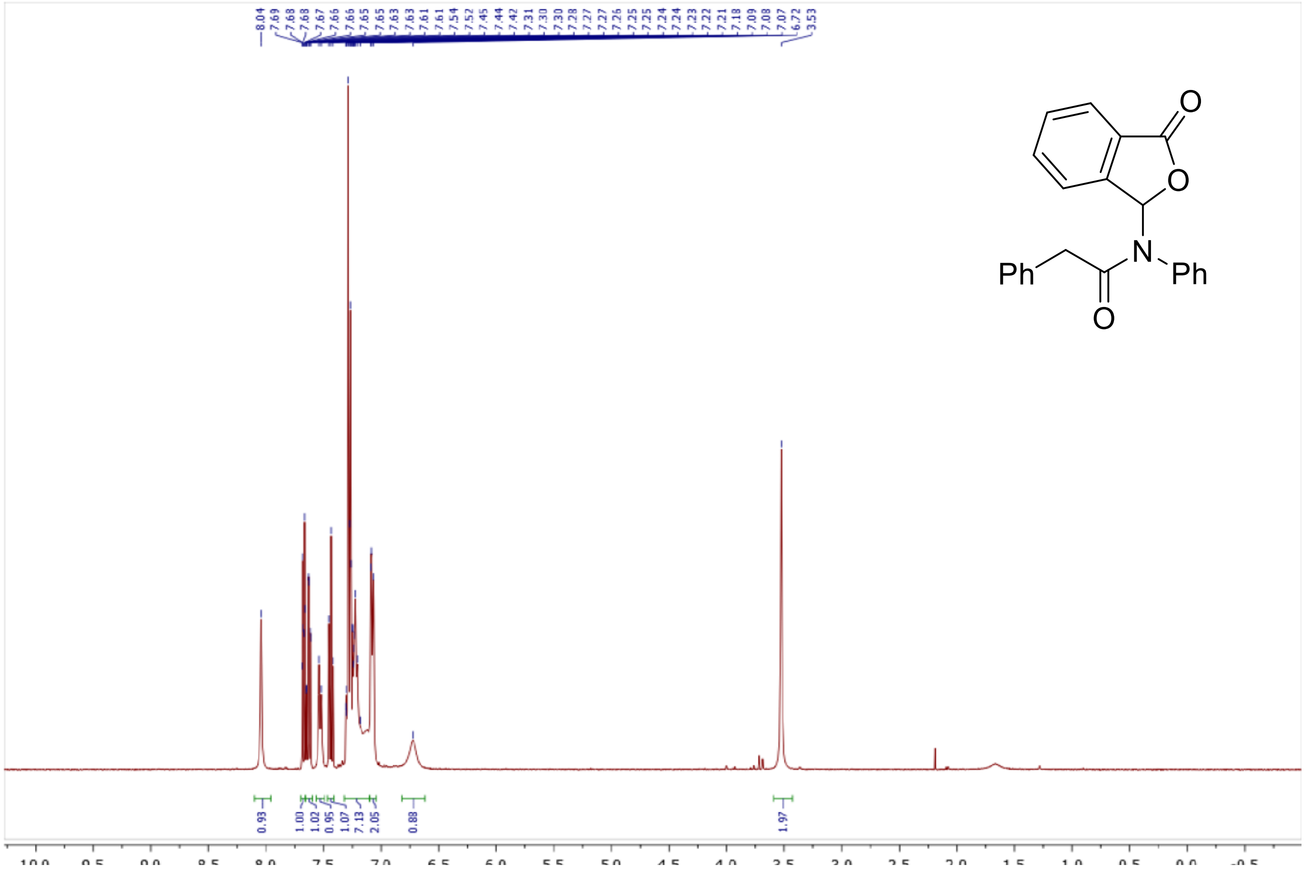

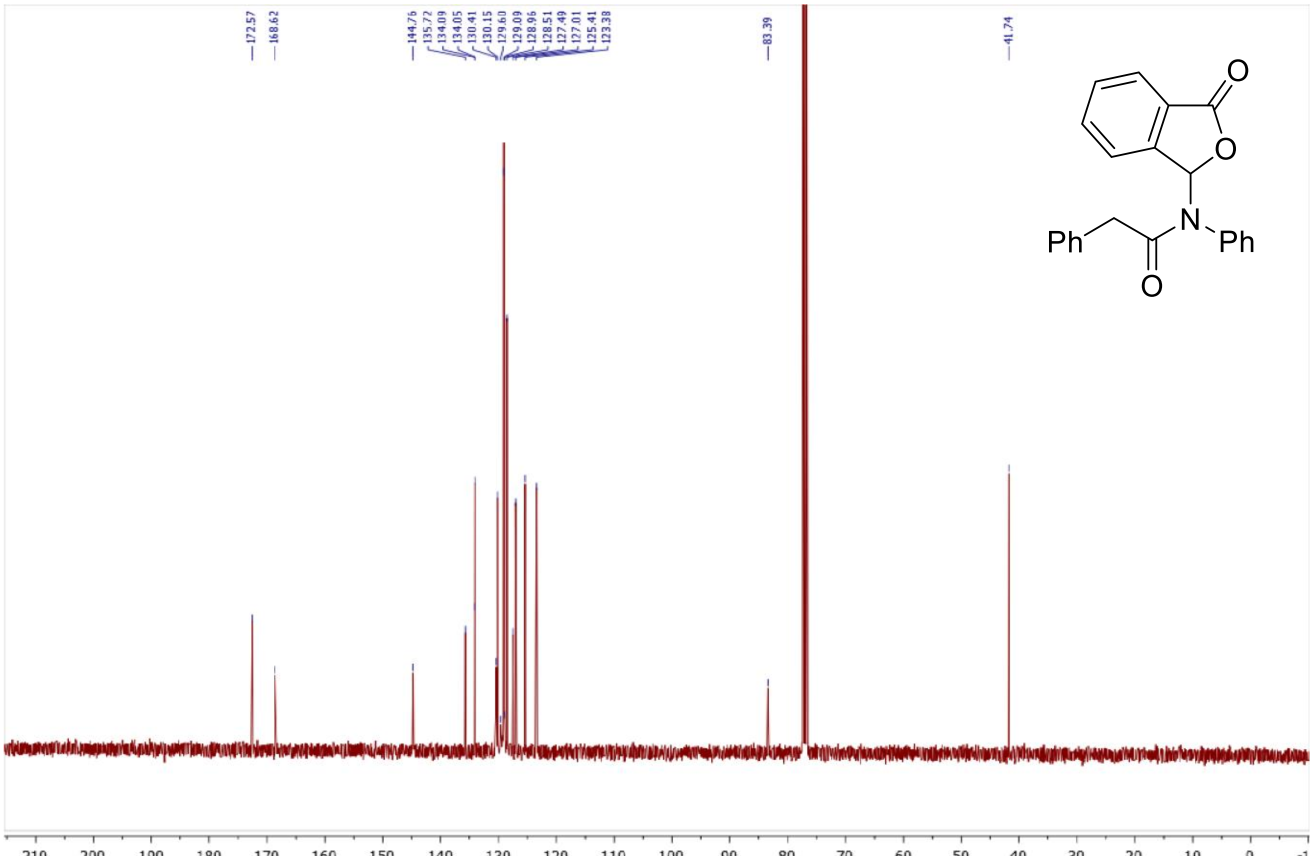

Copies of ^{1}H (400.13 MHz, $CDCl_3$) and ^{13}C{^{1}H} (100.61 MHz, $CDCl_3$) spectra of **10a**

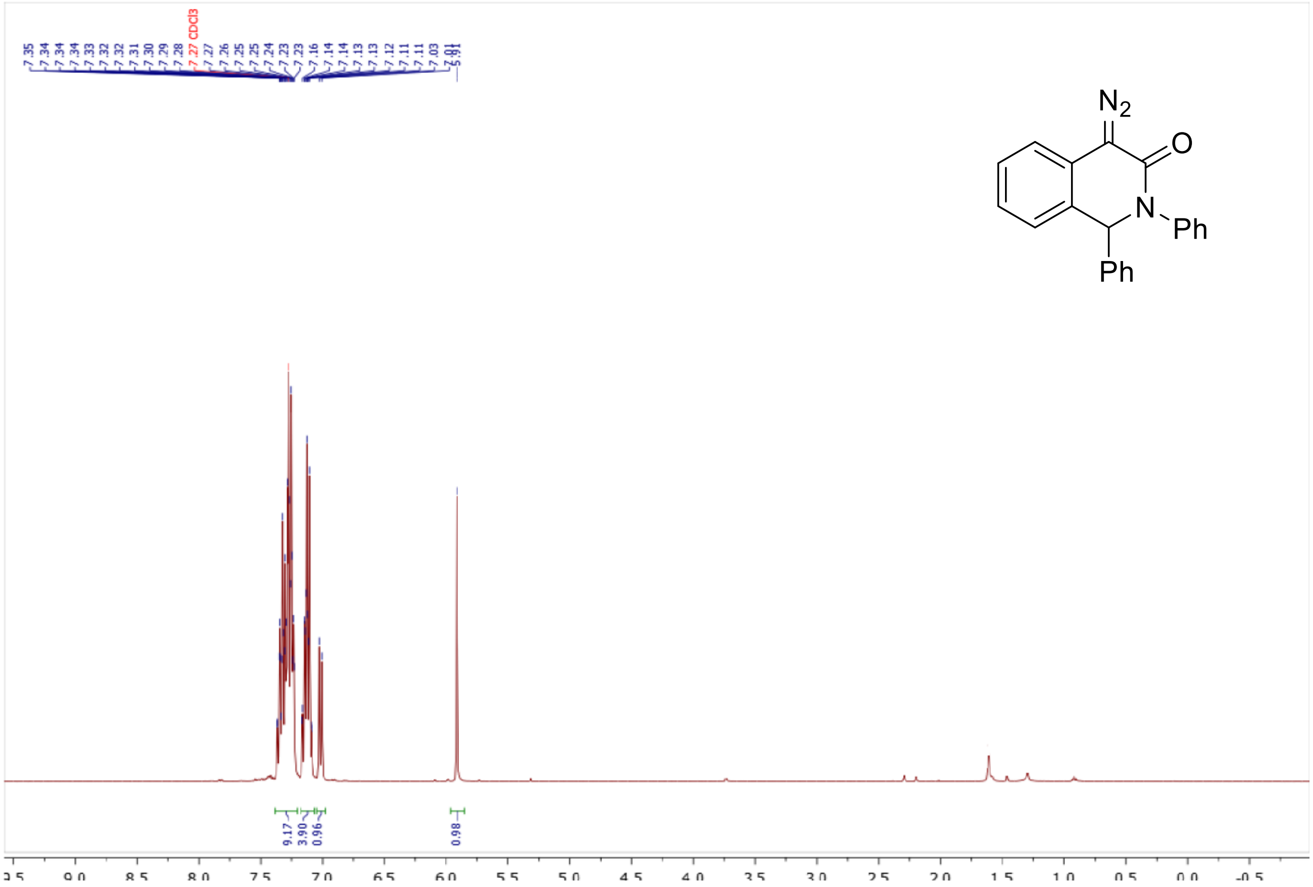

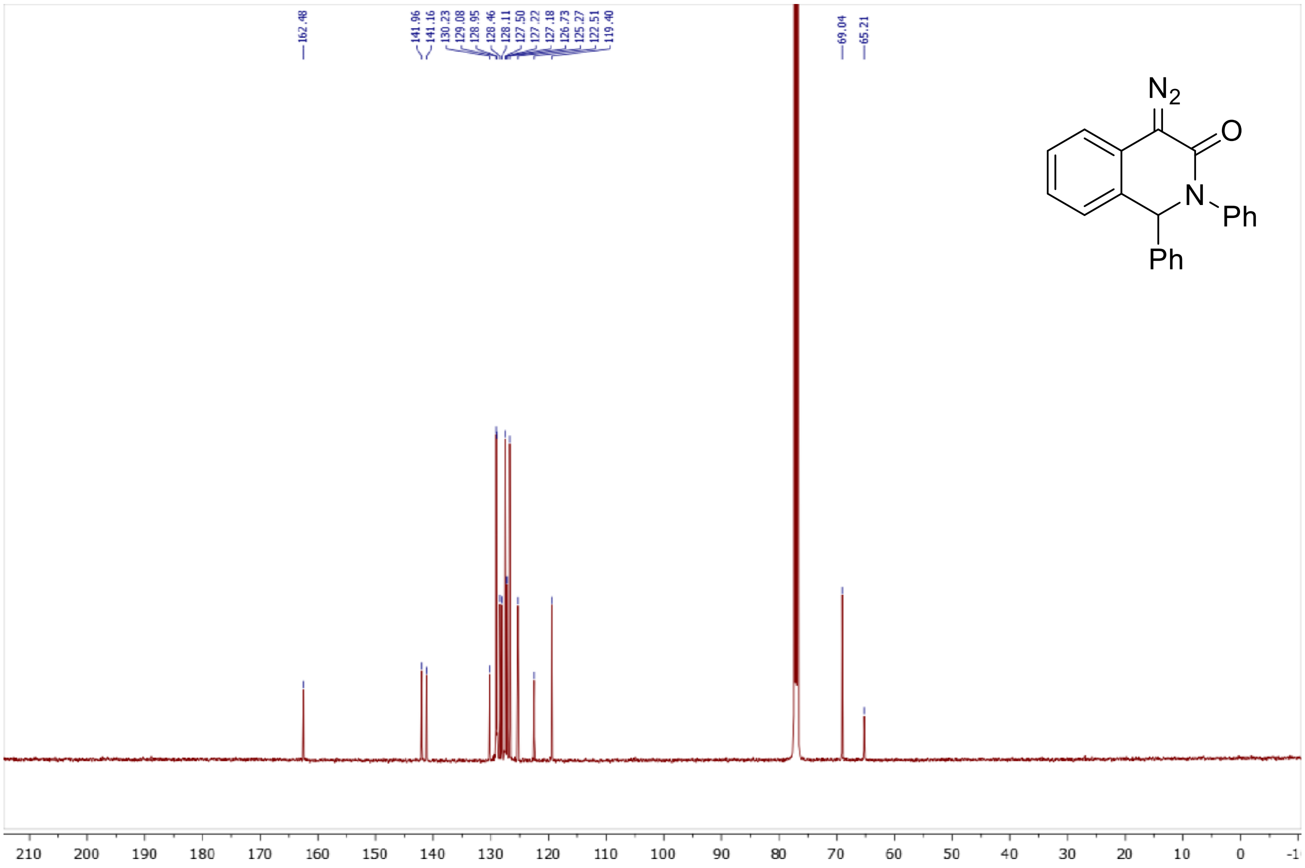

Copies of ^{1}H (400.13 MHz, $CDCl_3$) and $^{13}C\{^{1}H\}$ (100.61 MHz, $CDCl_3$) spectra of **10b**

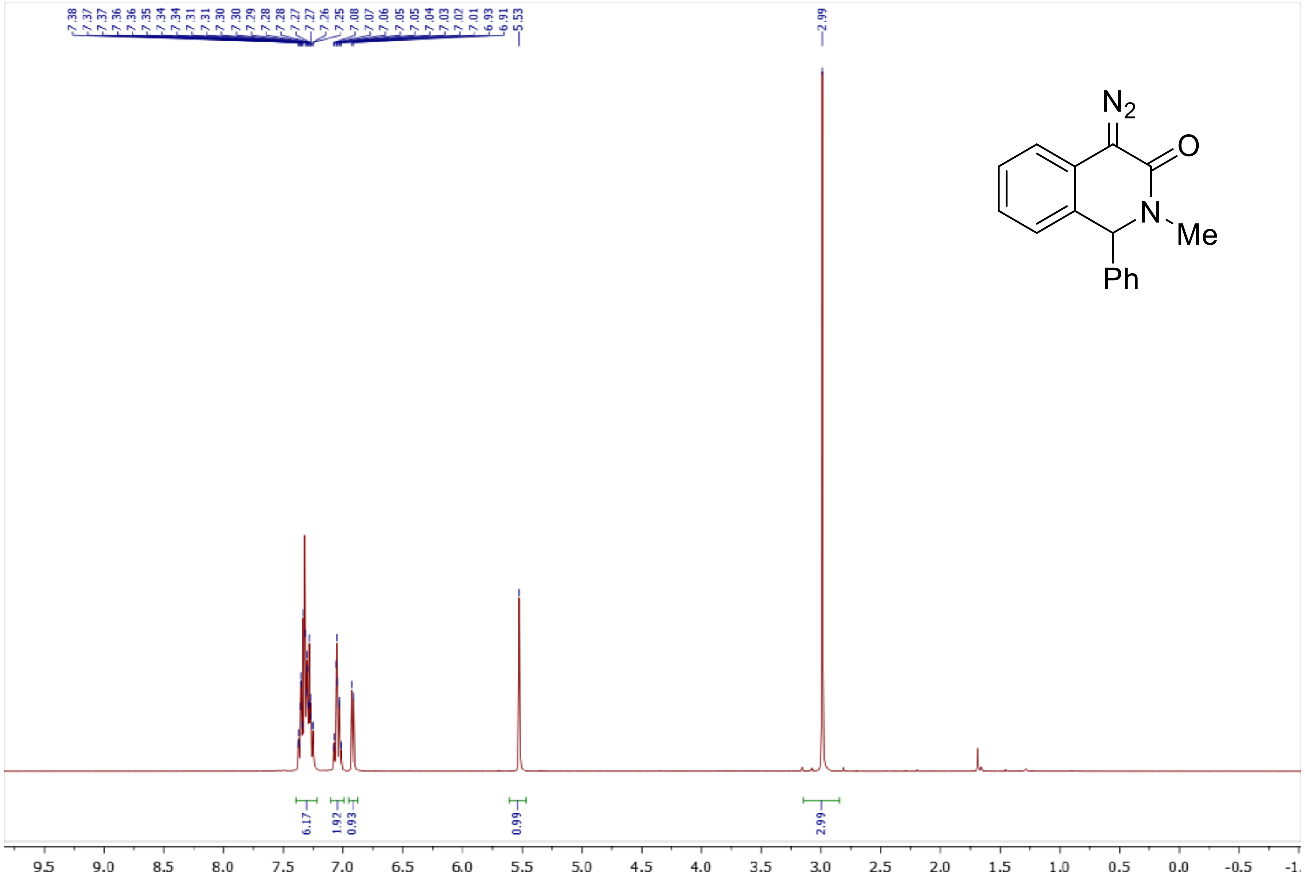

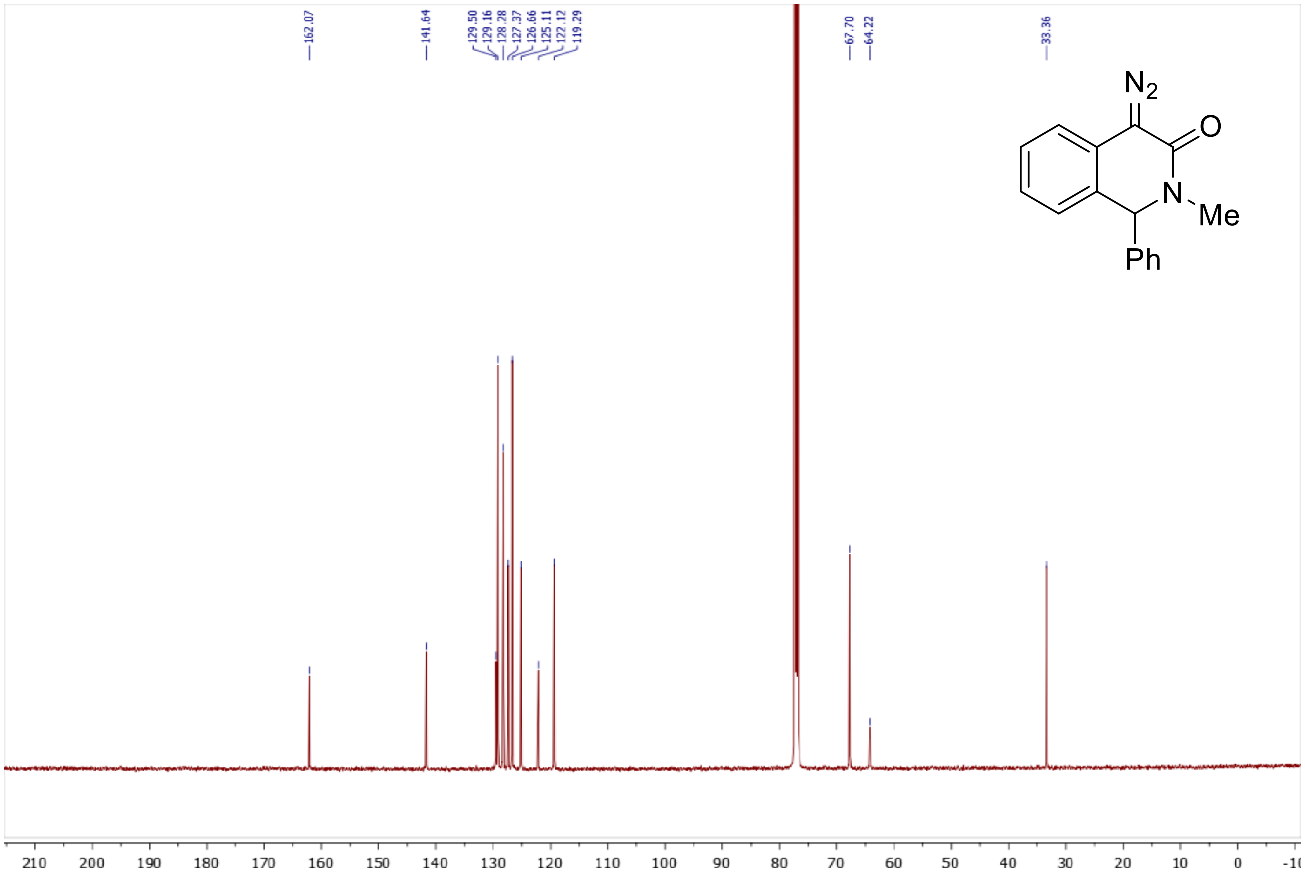

Copies of ^{1}H (400.13 MHz, $CDCl_3$) and $^{13}C\{^{1}H\}$ (100.61 MHz, $CDCl_3$) spectra of **10c**

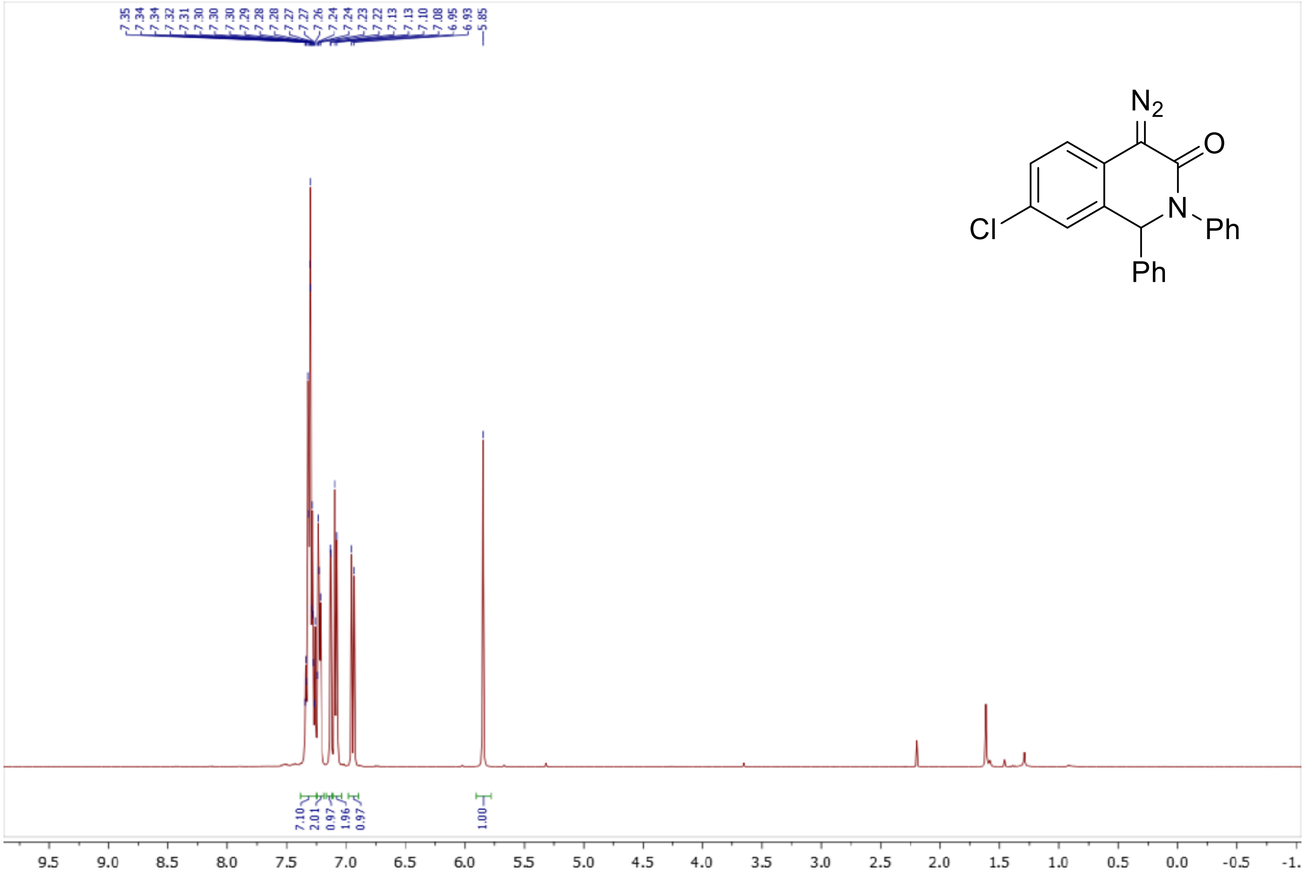

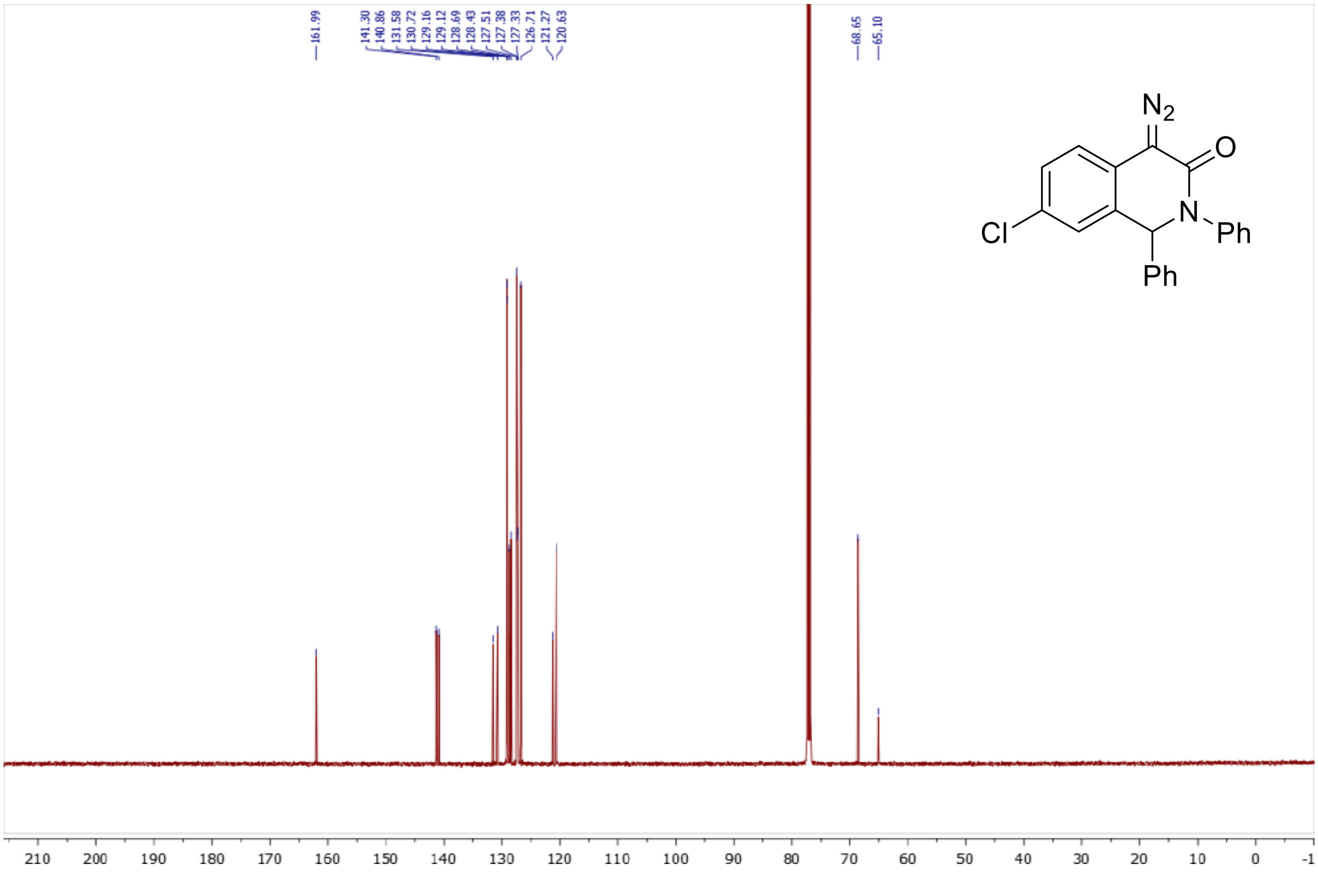

Copies of ^{1}H (400.13 MHz, $CDCl_3$) and $^{13}C\{^{1}H\}$ (100.61 MHz, $CDCl_3$) spectra of **10d**

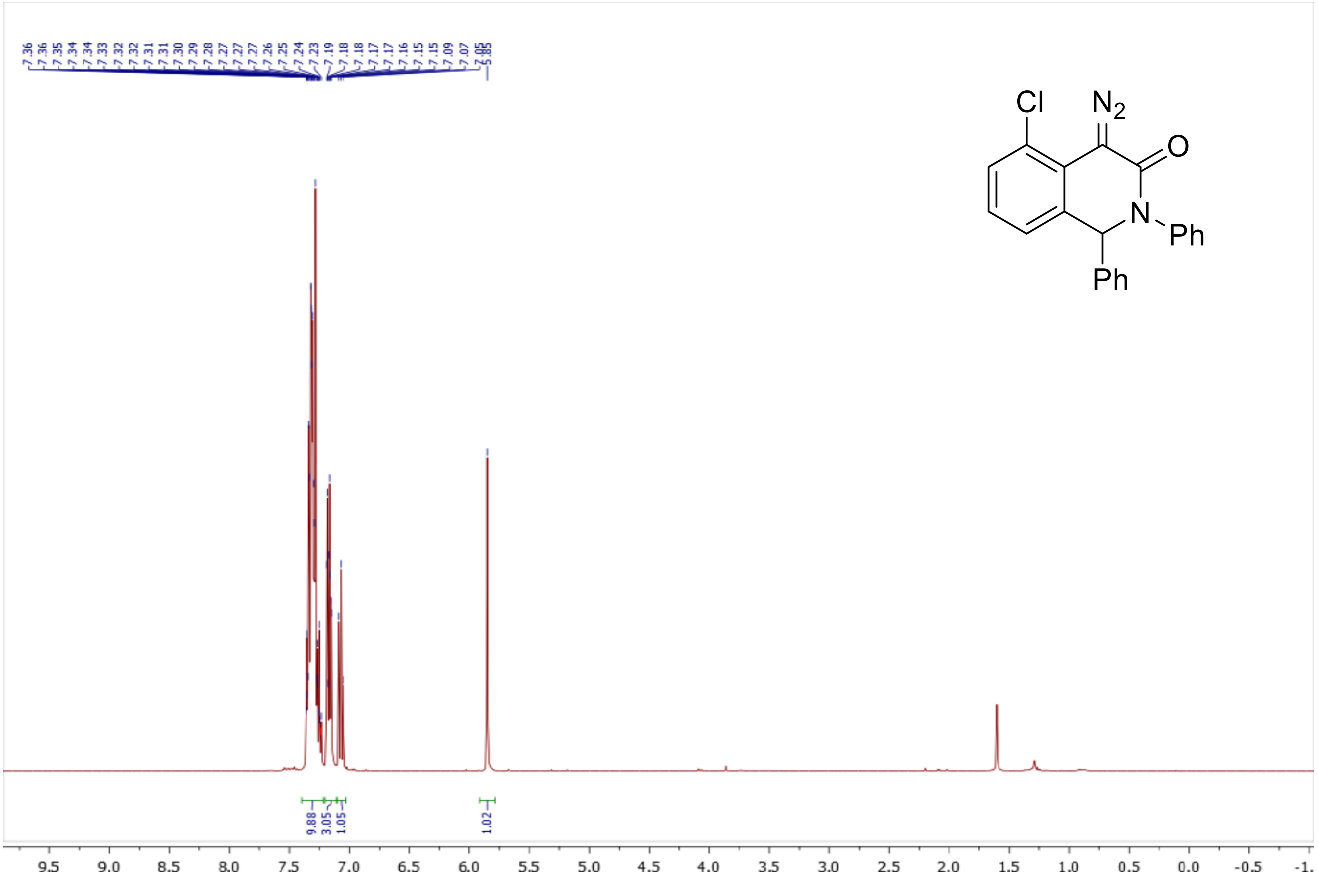

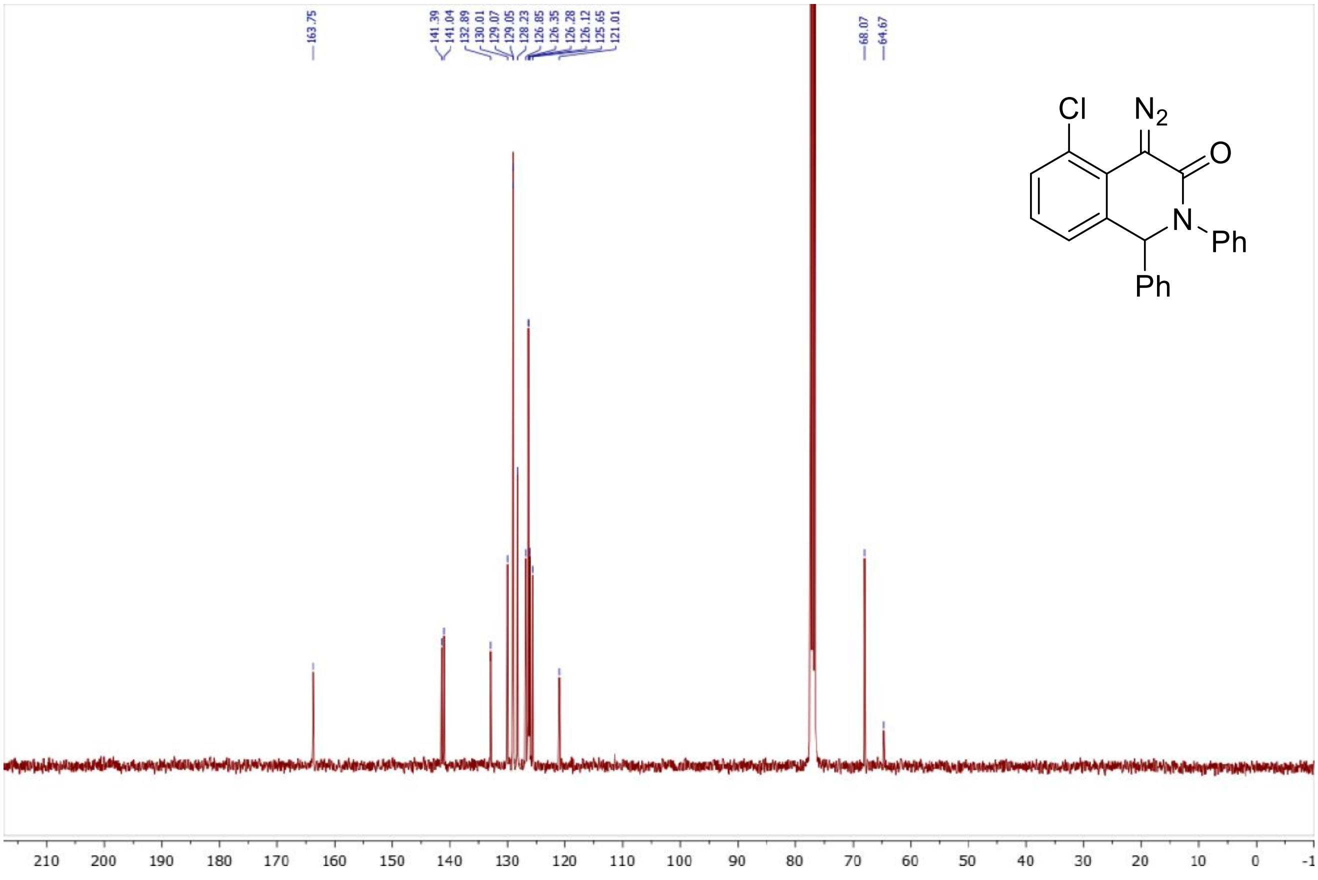

Copies of ^{1}H (400.13 MHz, $CDCl_3$) and $^{13}C\{^{1}H\}$ (100.61 MHz, $CDCl_3$) spectra of **10e**

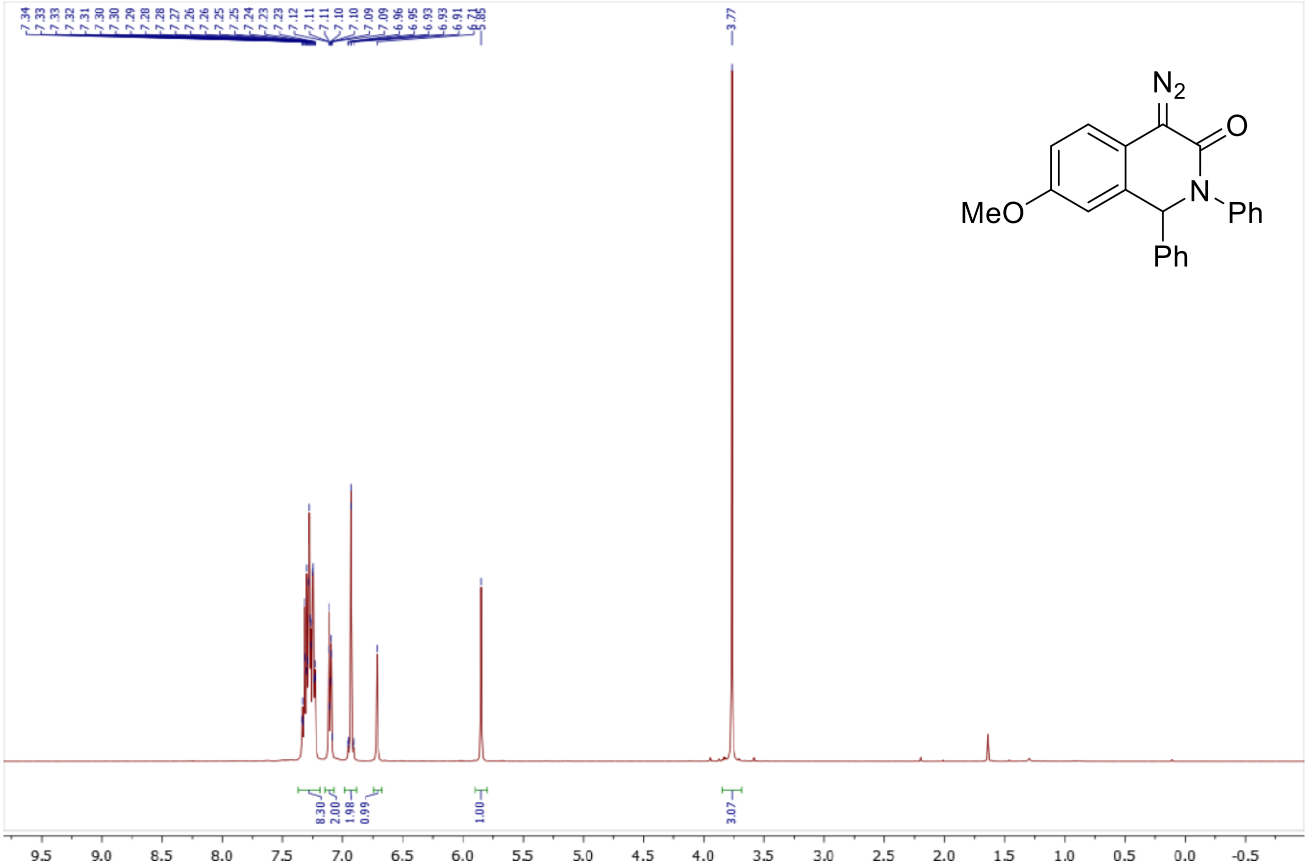

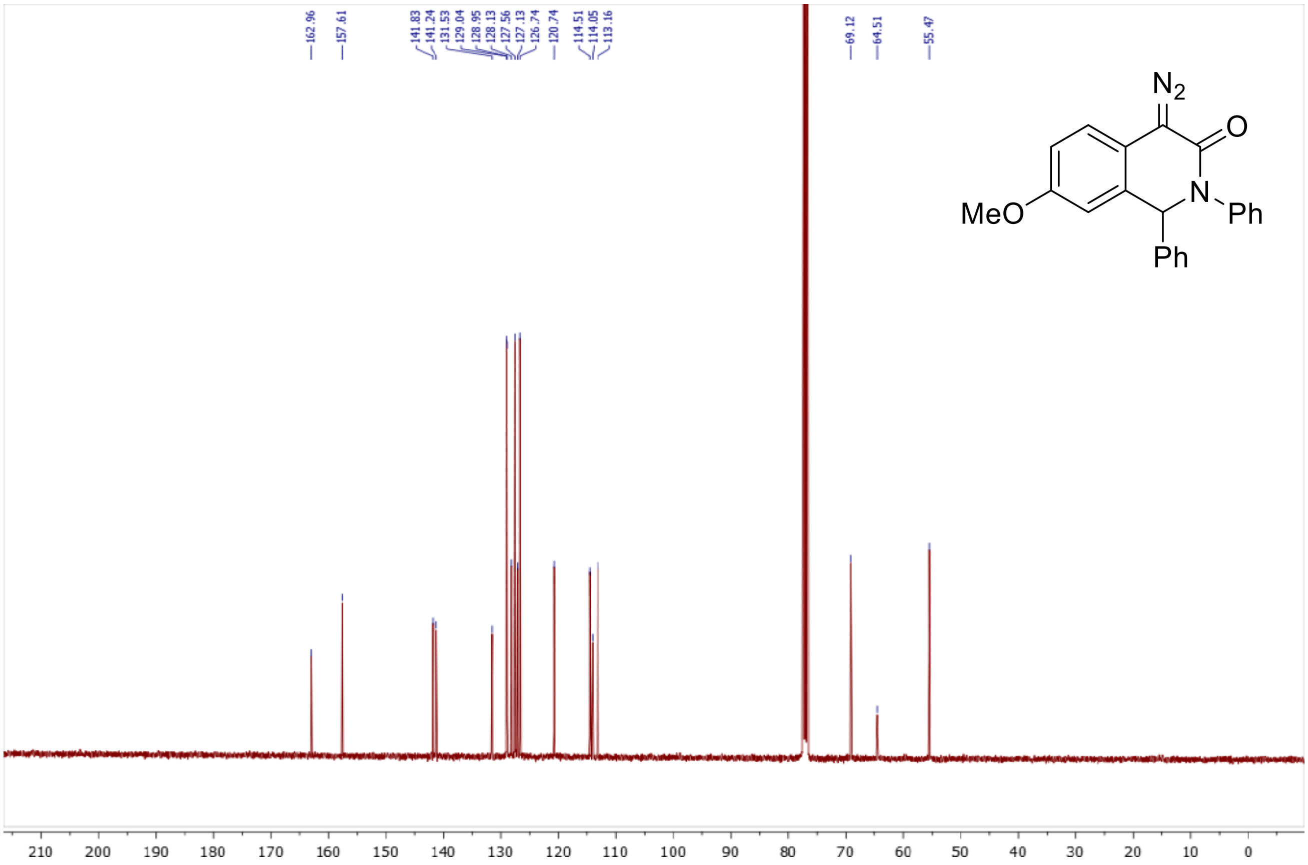

Copies of ^{1}H (400.13 MHz, $CDCl_3$) and $^{13}C\{^{1}H\}$ (100.61 MHz, $CDCl_3$) spectra of **10f**

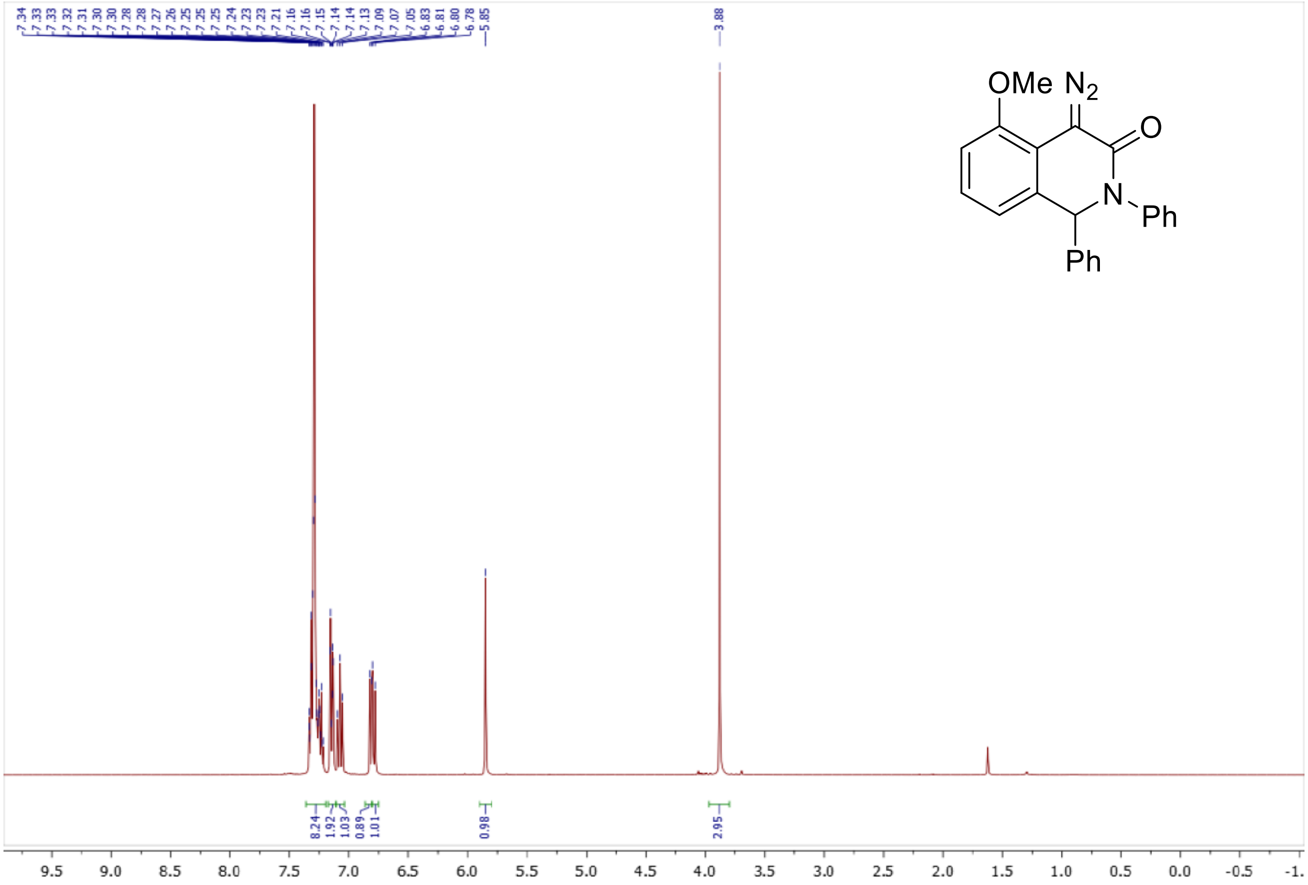

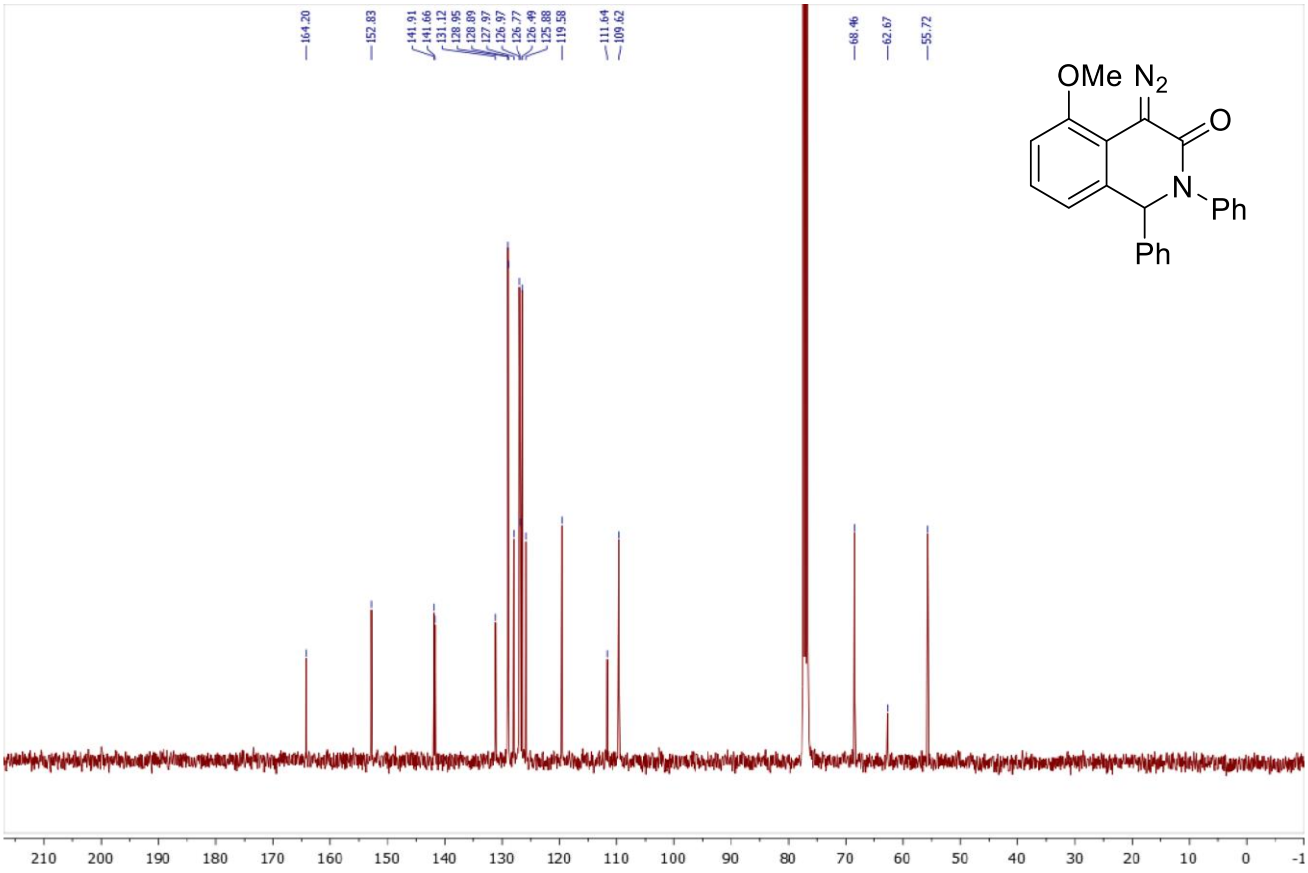

Copies of ^{1}H (400.13 MHz, $CDCl_3$) and ^{13}C{^{1}H} (100.61 MHz, $CDCl_3$) spectra of **10g**

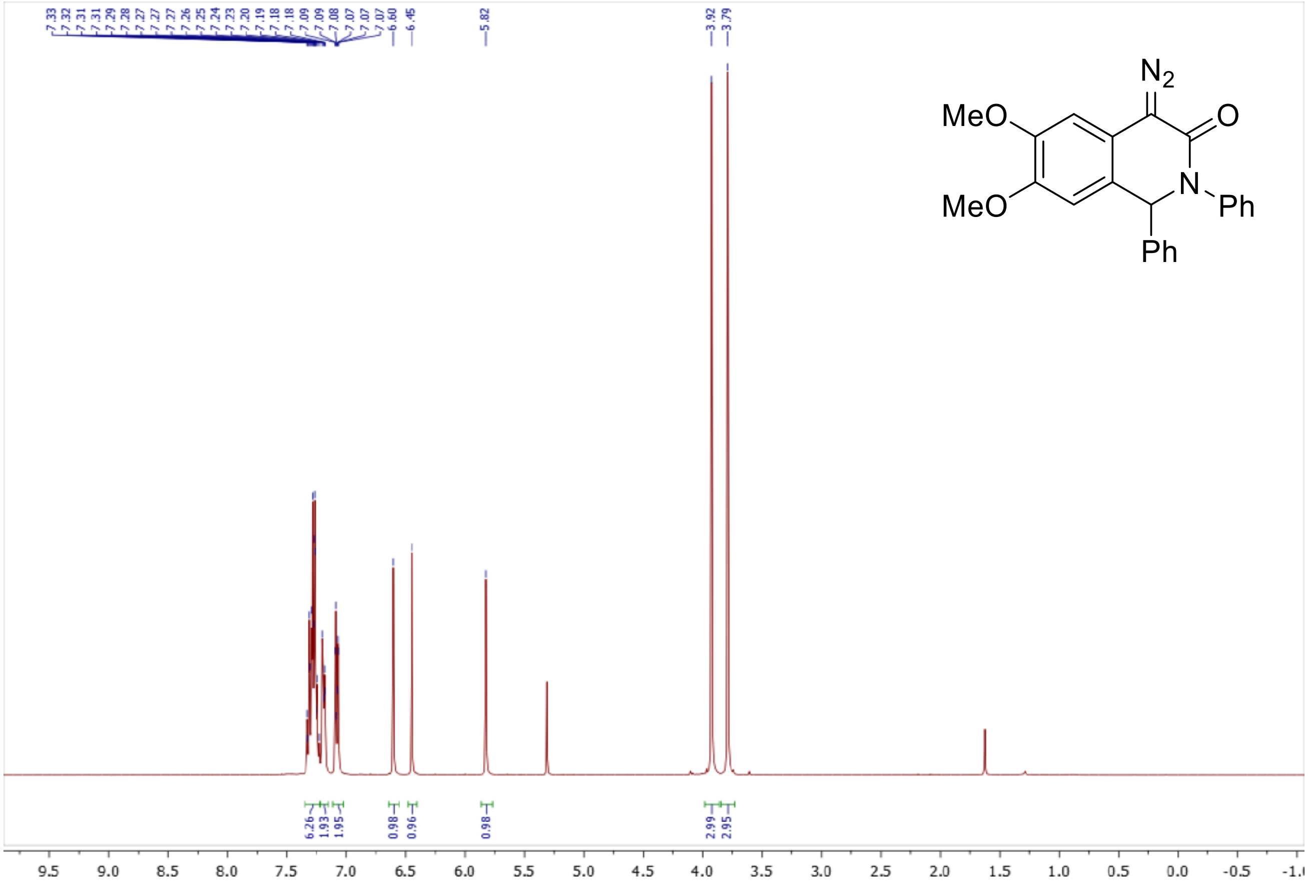

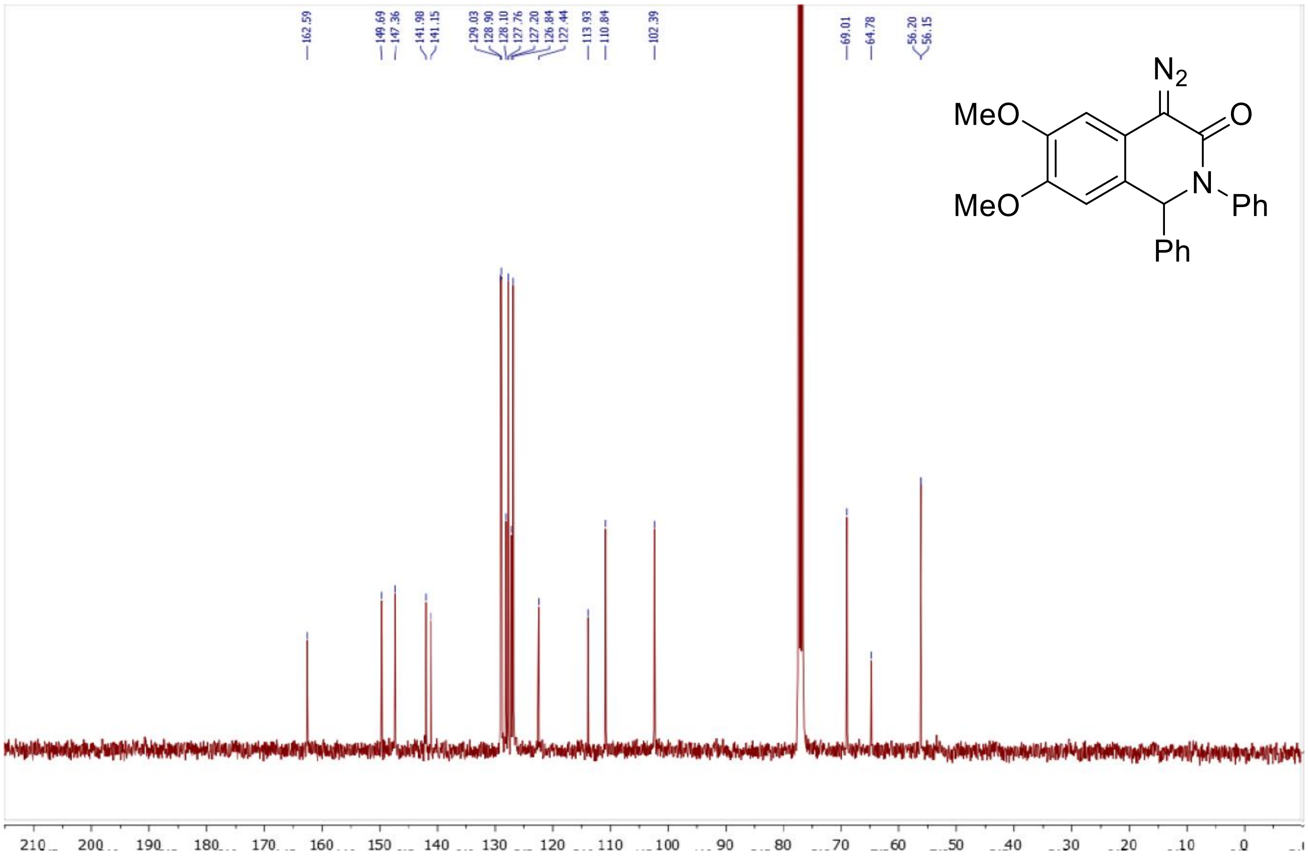

Copies of ^{1}H (400.13 MHz, $CDCl_3$) and $^{13}C\{^{1}H\}$ (100.61 MHz, $CDCl_3$) spectra of **10h**

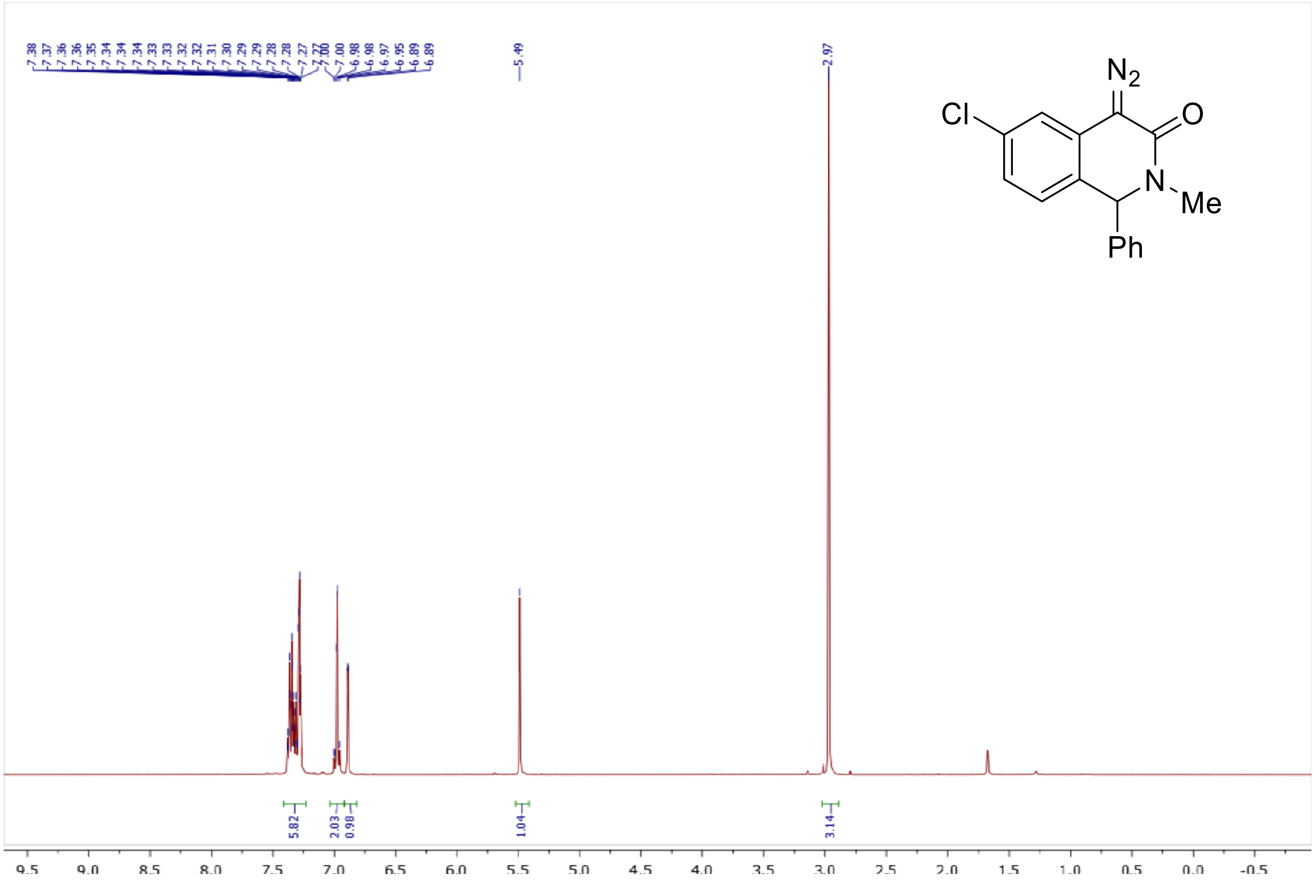

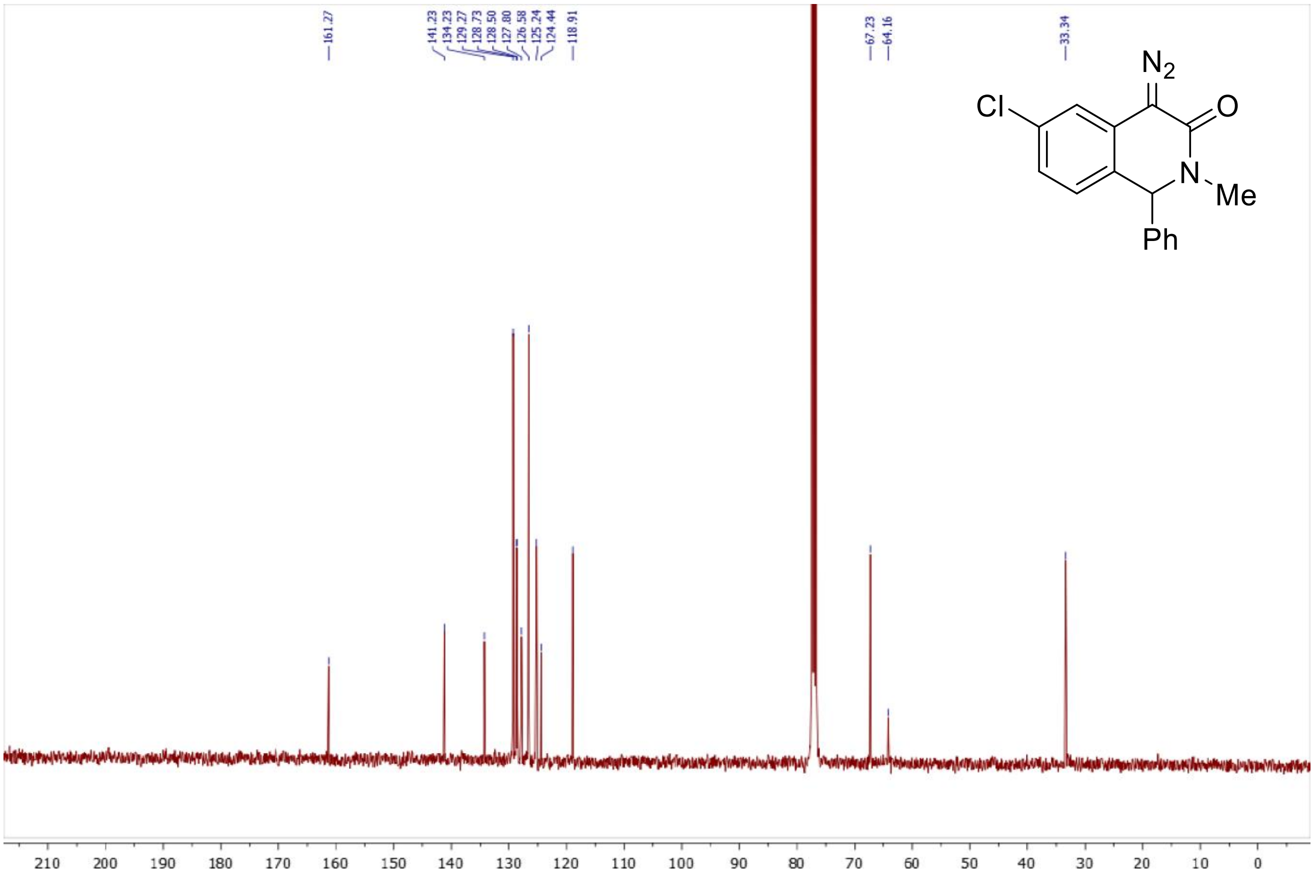

Copies of ^{1}H (400.13 MHz, $CDCl_3$) and $^{13}C\{^{1}H\}$ (100.61 MHz, $CDCl_3$) spectra of **10i**

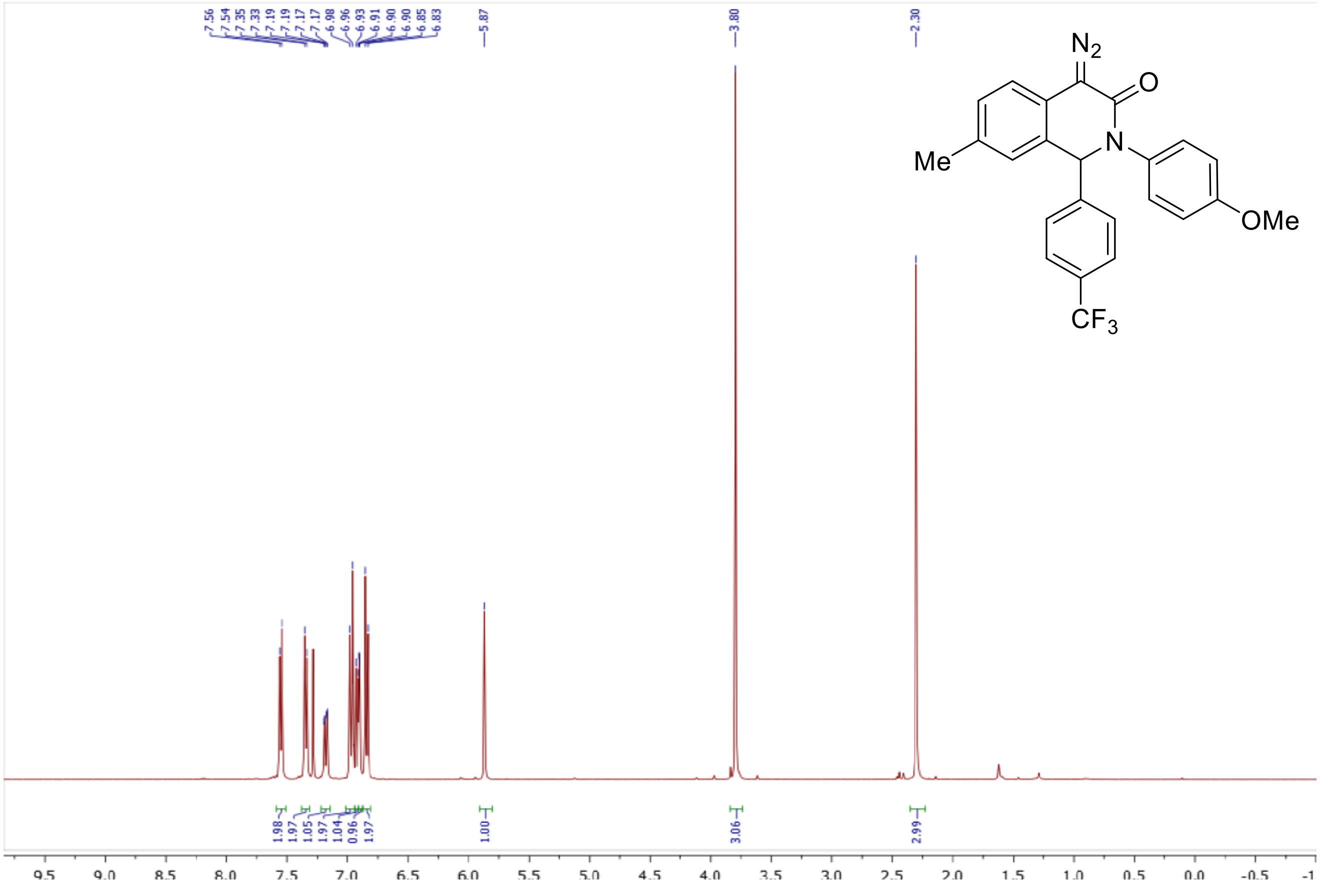

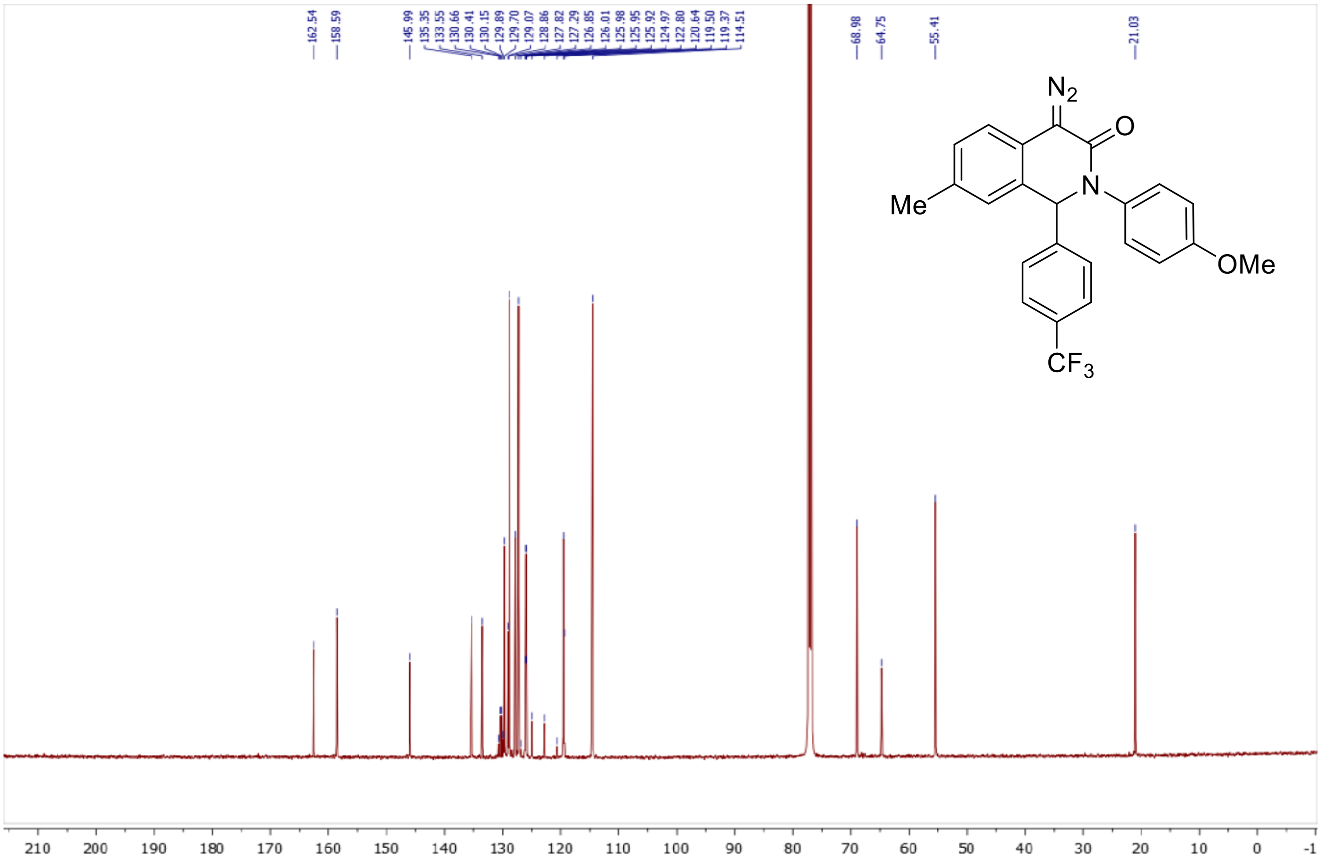

Copy of $^{19}F\{^{1}H\}$ (376.50 MHz, $CDCl_3$) spectrum of **10i**

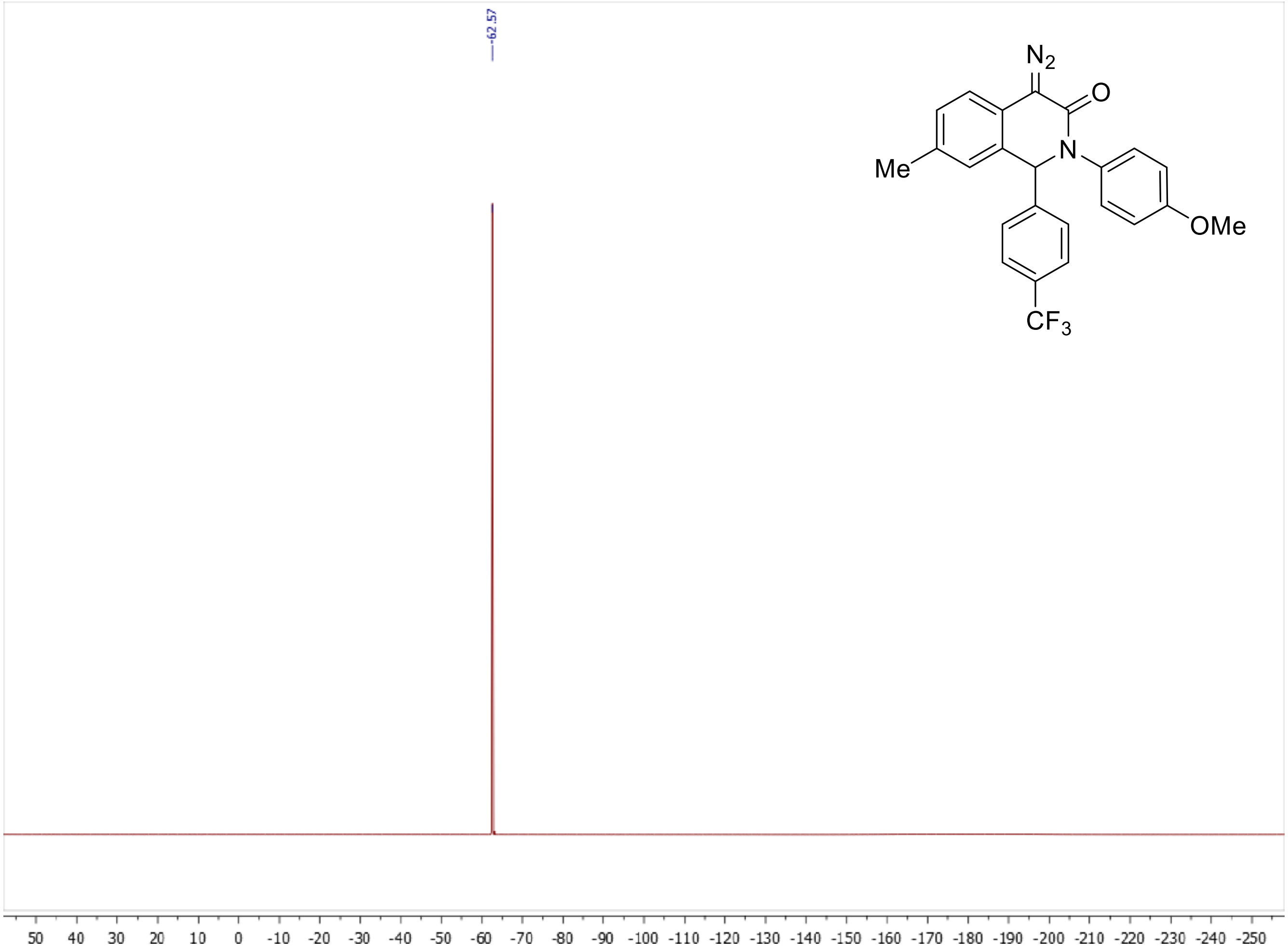

Copies of ^{1}H (400.13 MHz, $CDCl_3$) and ^{13}C{^{1}H} (100.61 MHz, $CDCl_3$) spectra of **10j**

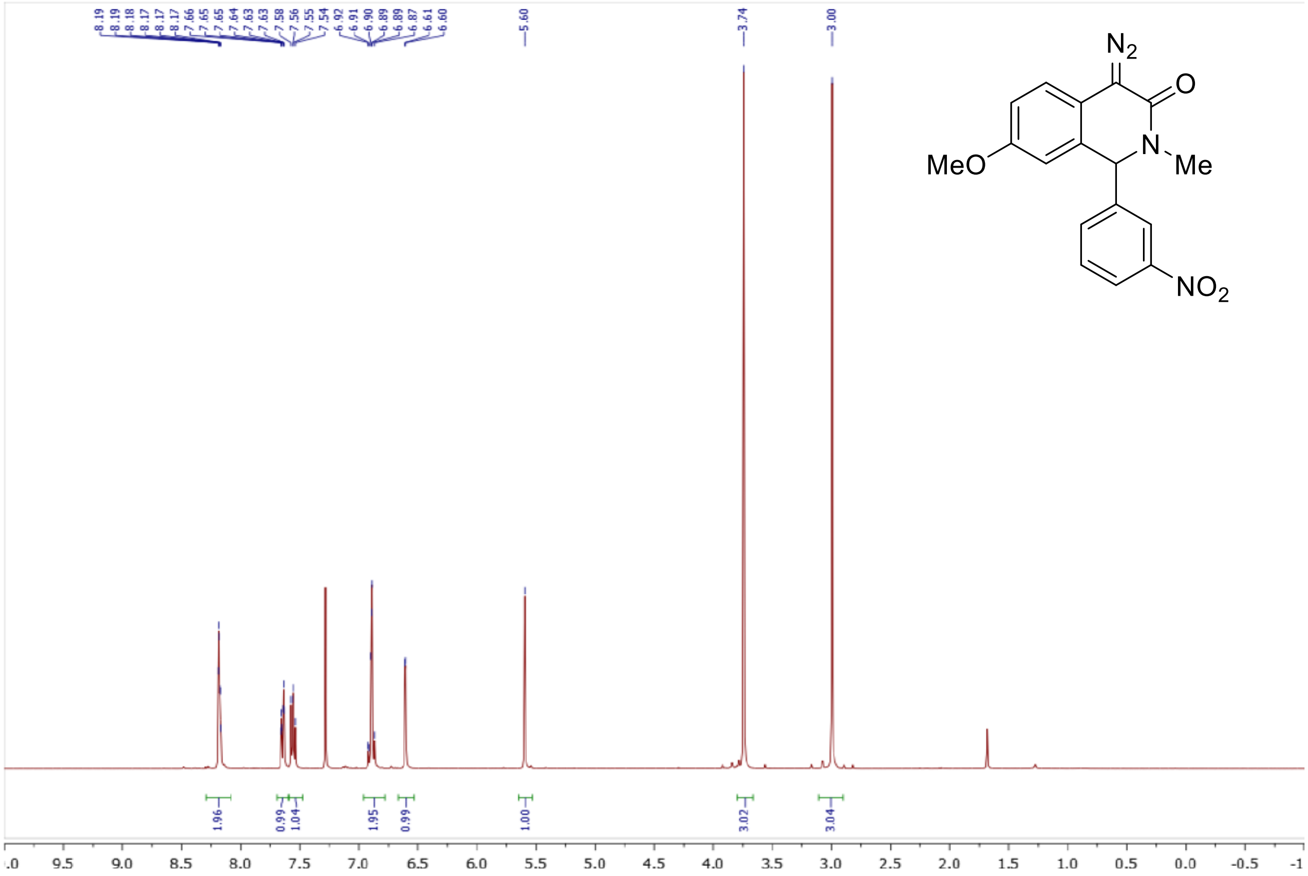

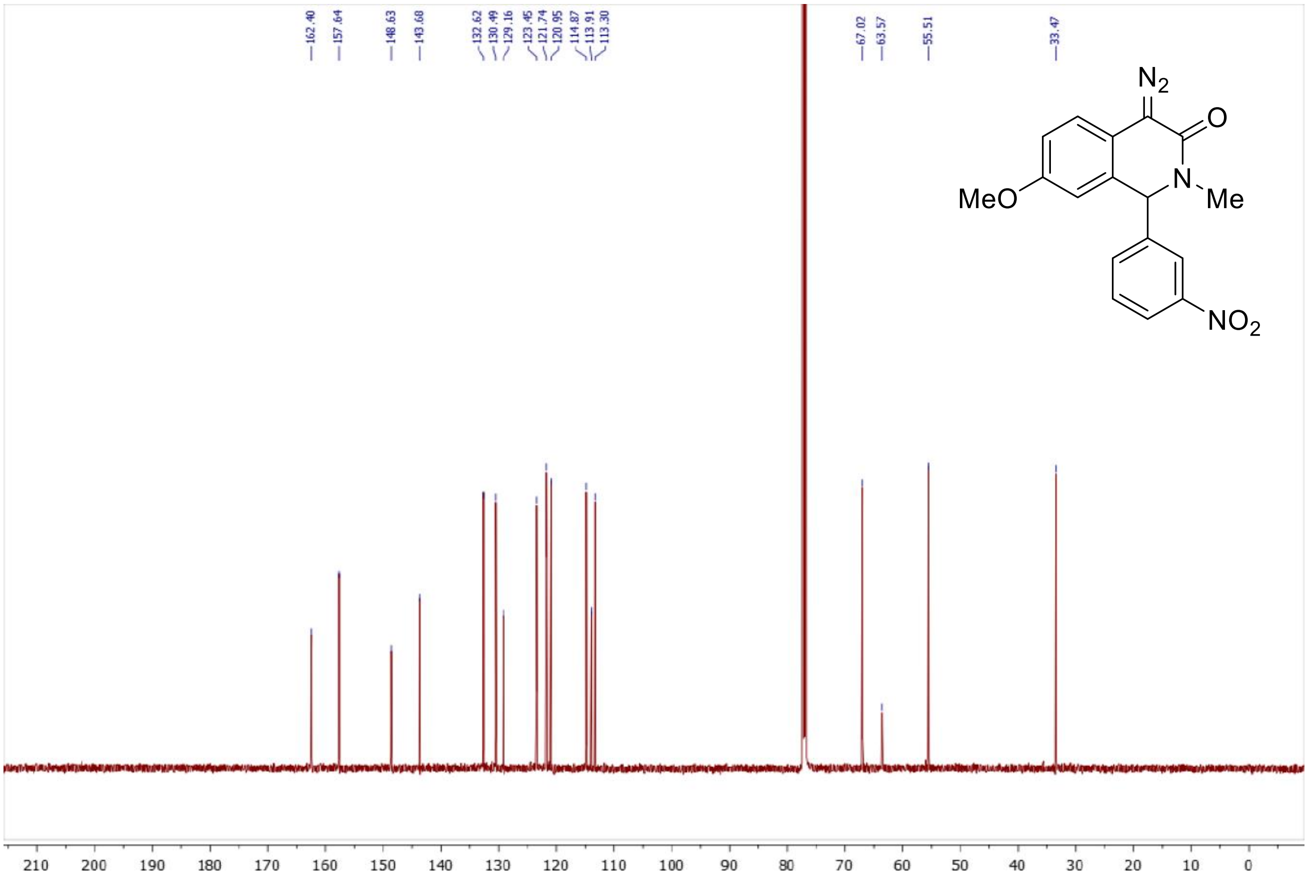

Copies of ^{1}H (400.13 MHz, $CDCl_3$) and $^{13}C\{^{1}H\}$ (100.61 MHz, $CDCl_3$) spectra of **10k**

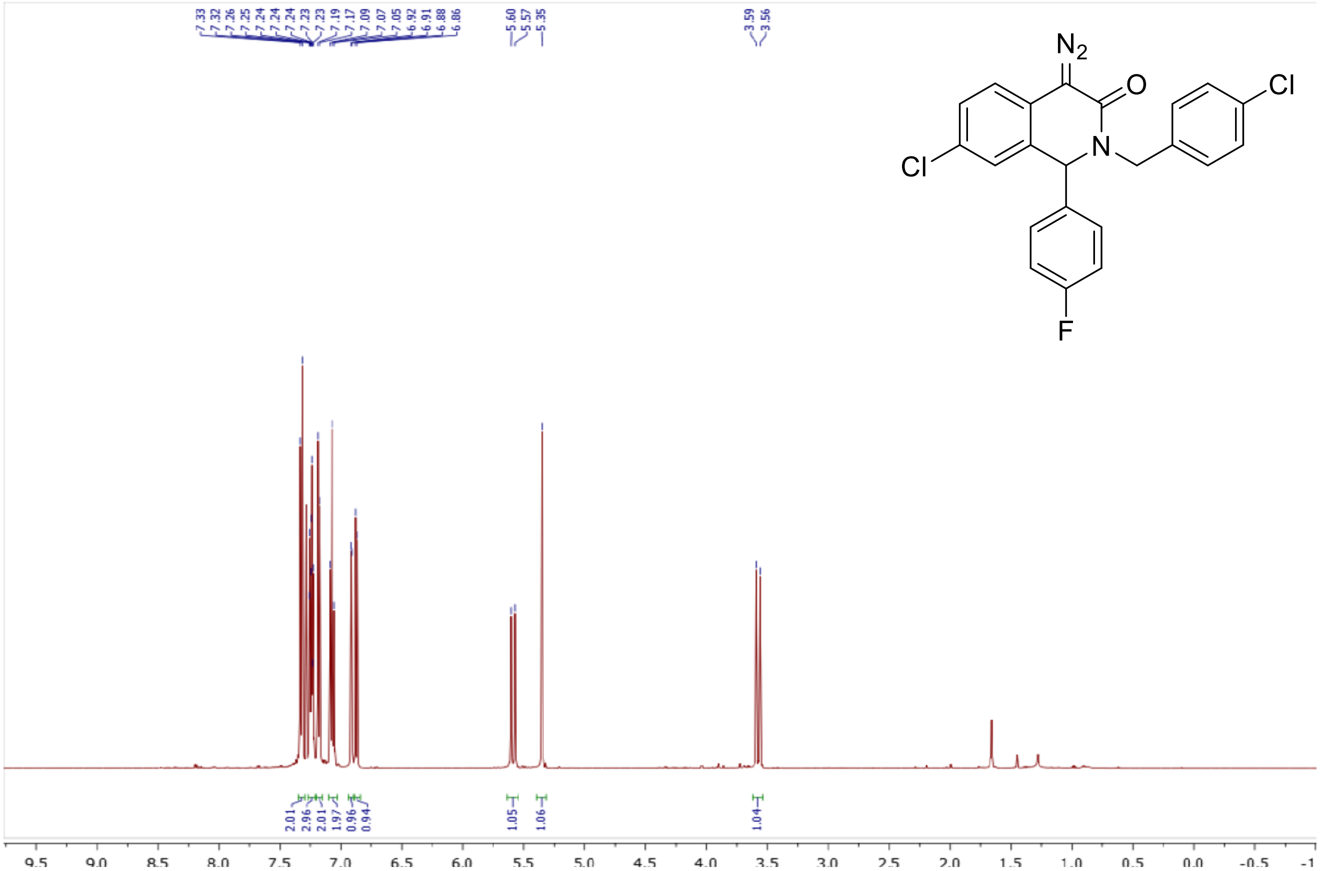

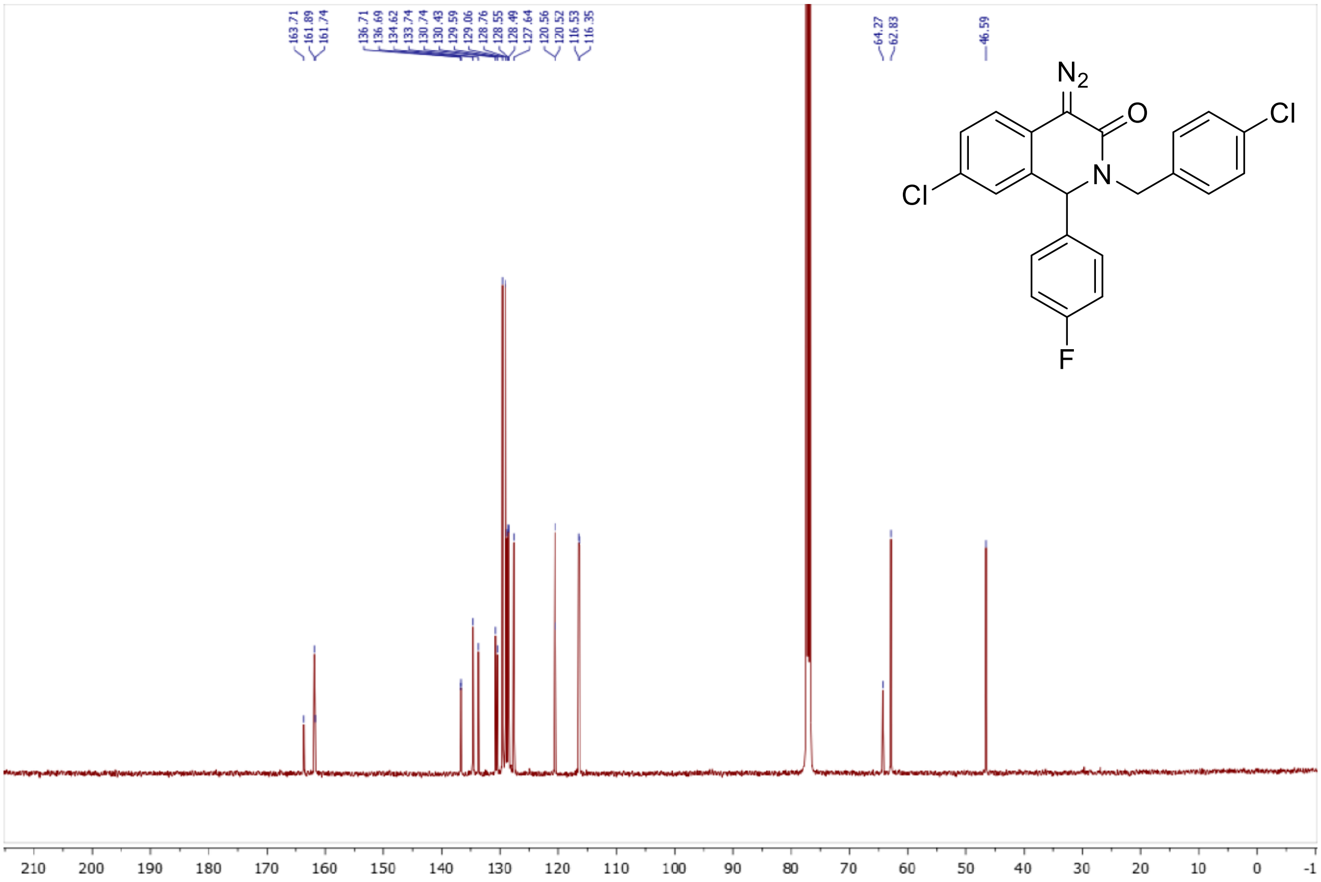

Copy of $^{19}F\{^1H\}$ (376.50 MHz, $CDCl_3$) spectrum of **10k**

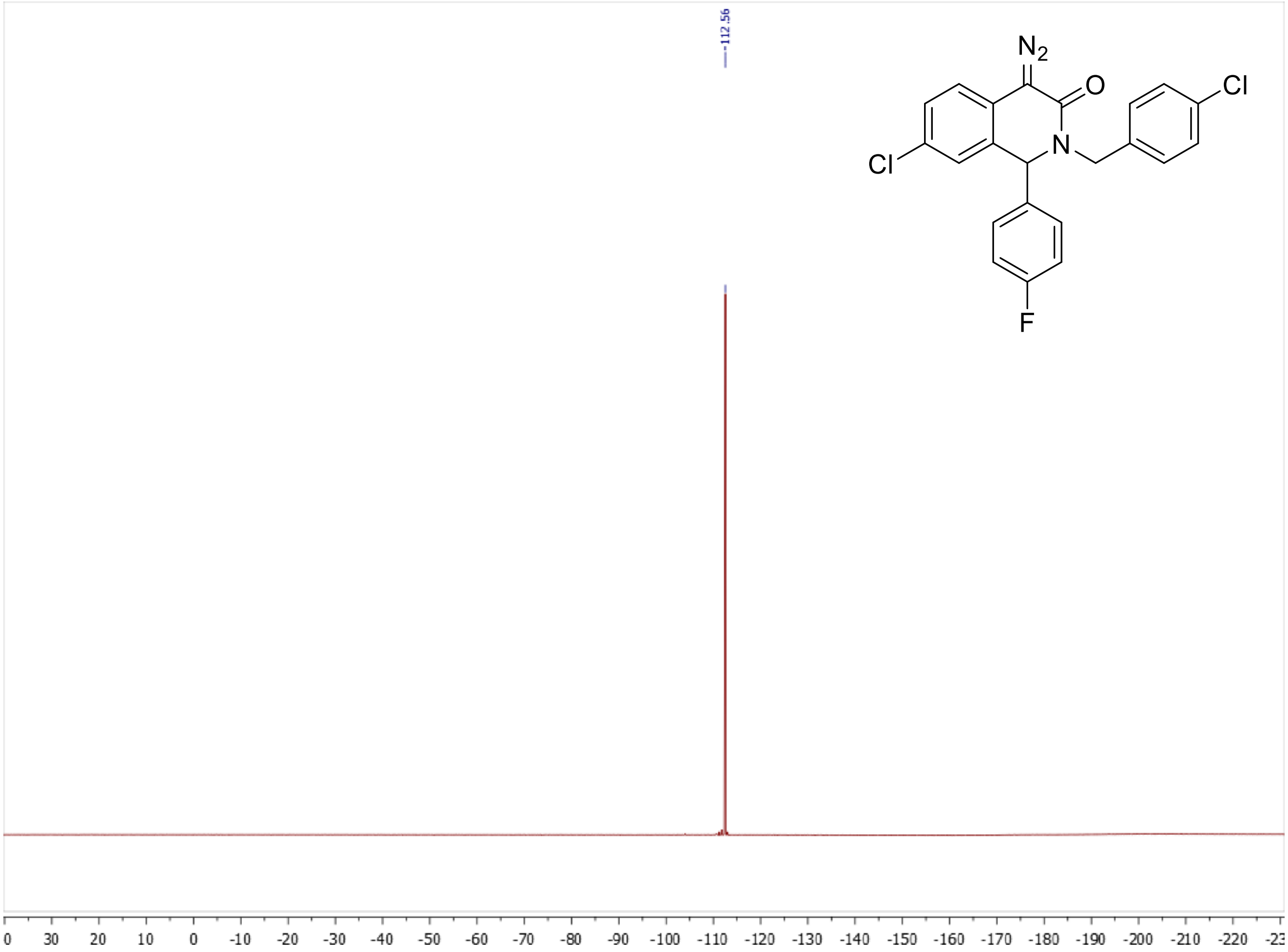

Copies of ^{1}H (400.13 MHz, $CDCl_3$) and ^{13}C{^{1}H} (100.61 MHz, $CDCl_3$) spectra of **10l**

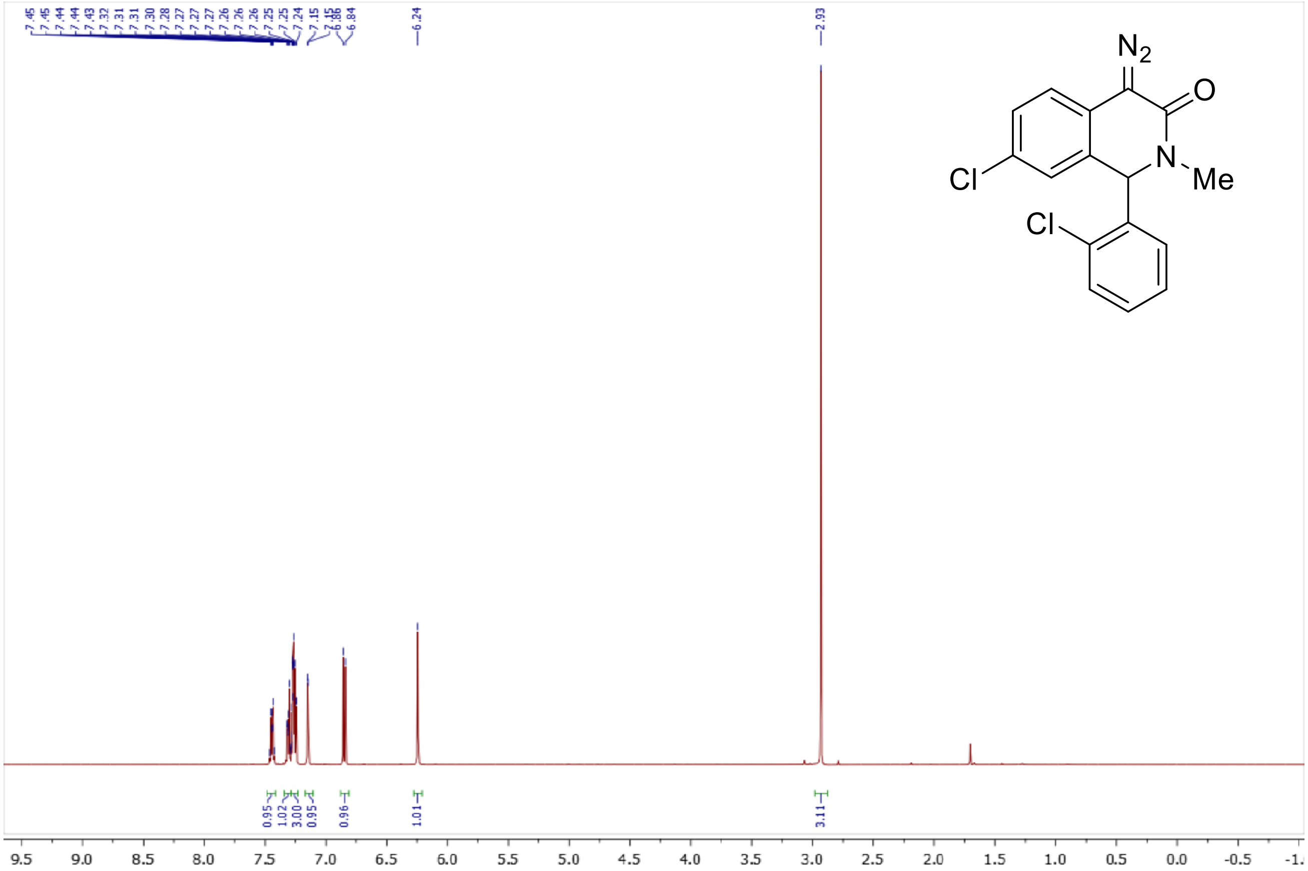

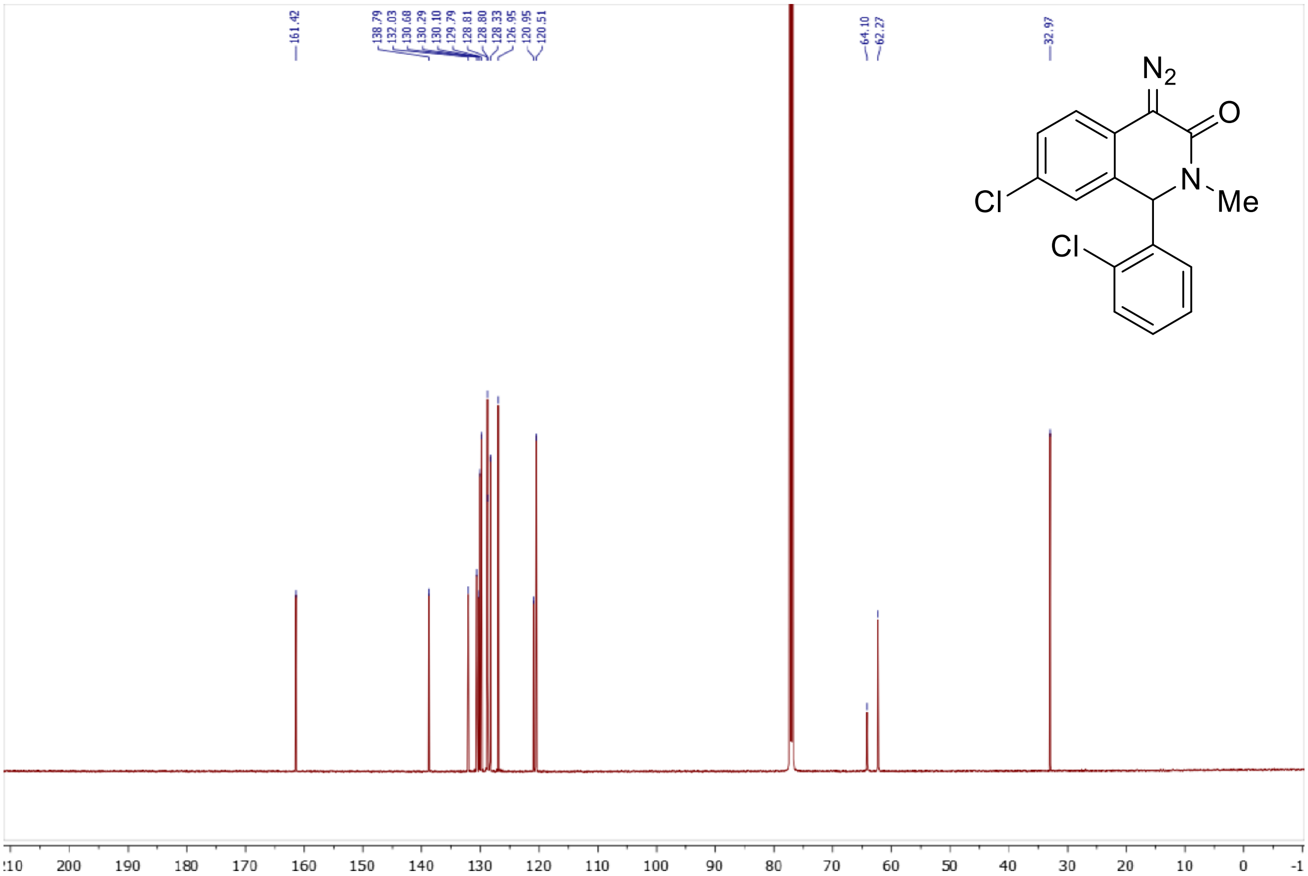

Copies of ^{1}H (400.13 MHz, $CDCl_3$) and $^{13}C\{^{1}H\}$ (100.61 MHz, $CDCl_3$) spectra of **10m**

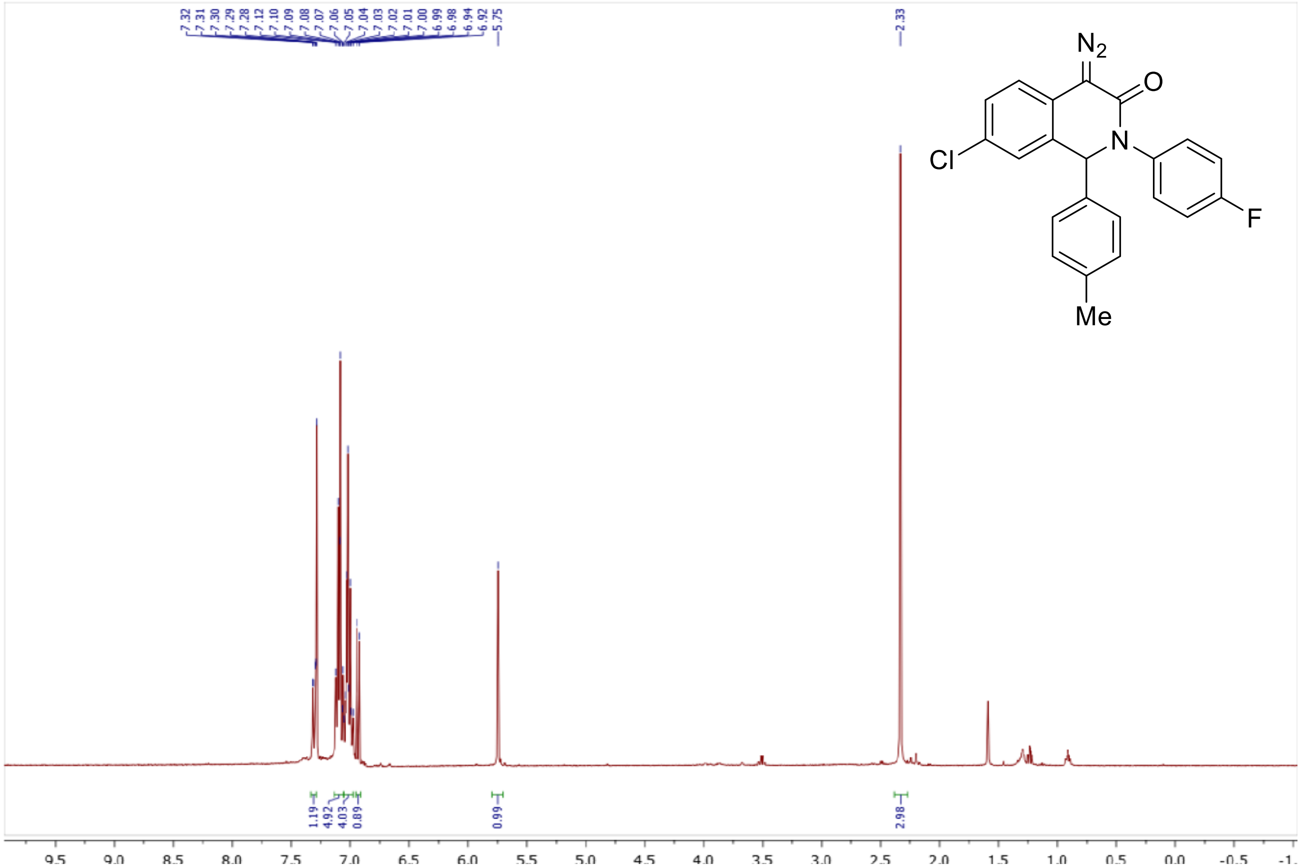

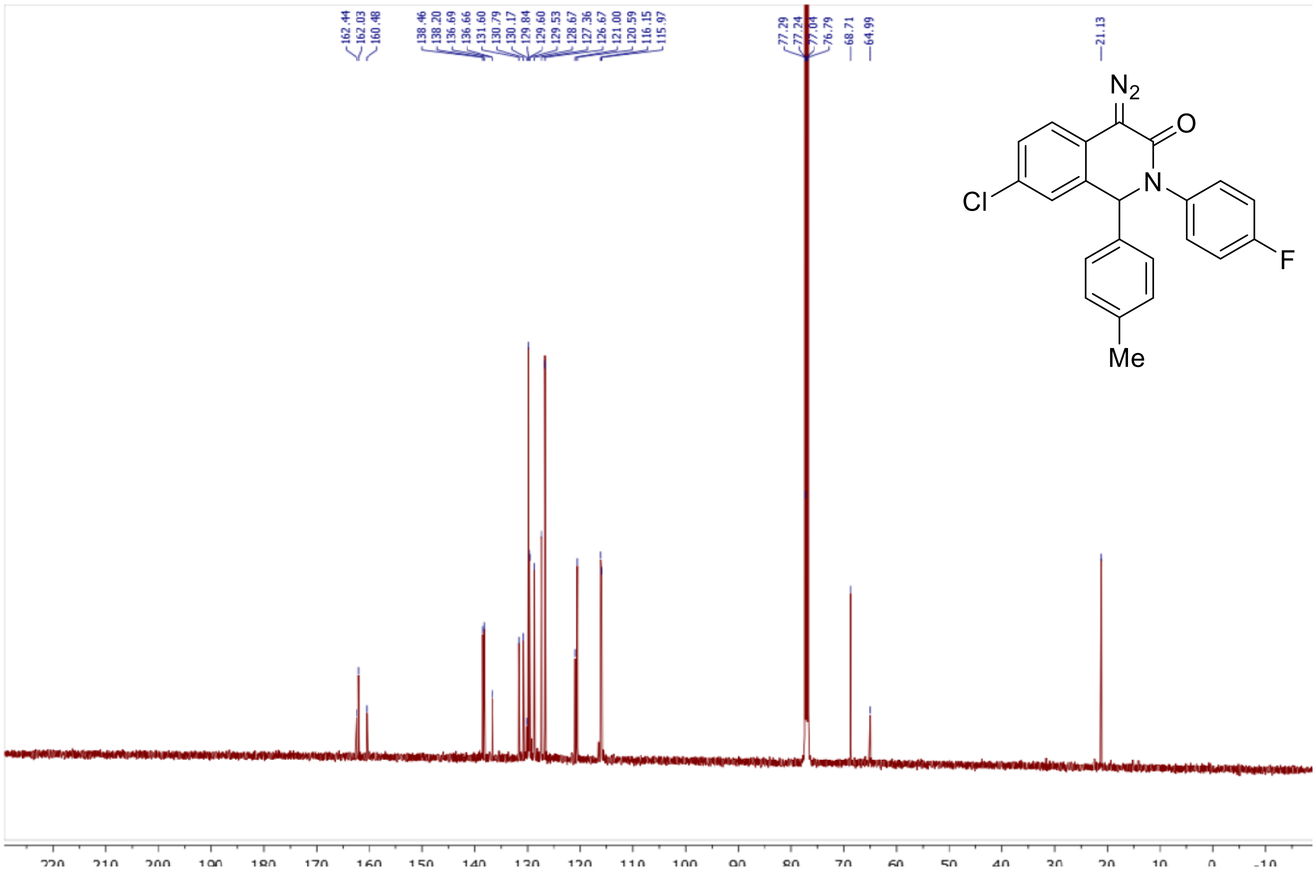

Copy of $^{19}F\{^1H\}$ (376.50 MHz, $CDCl_3$) spectrum of **10m**

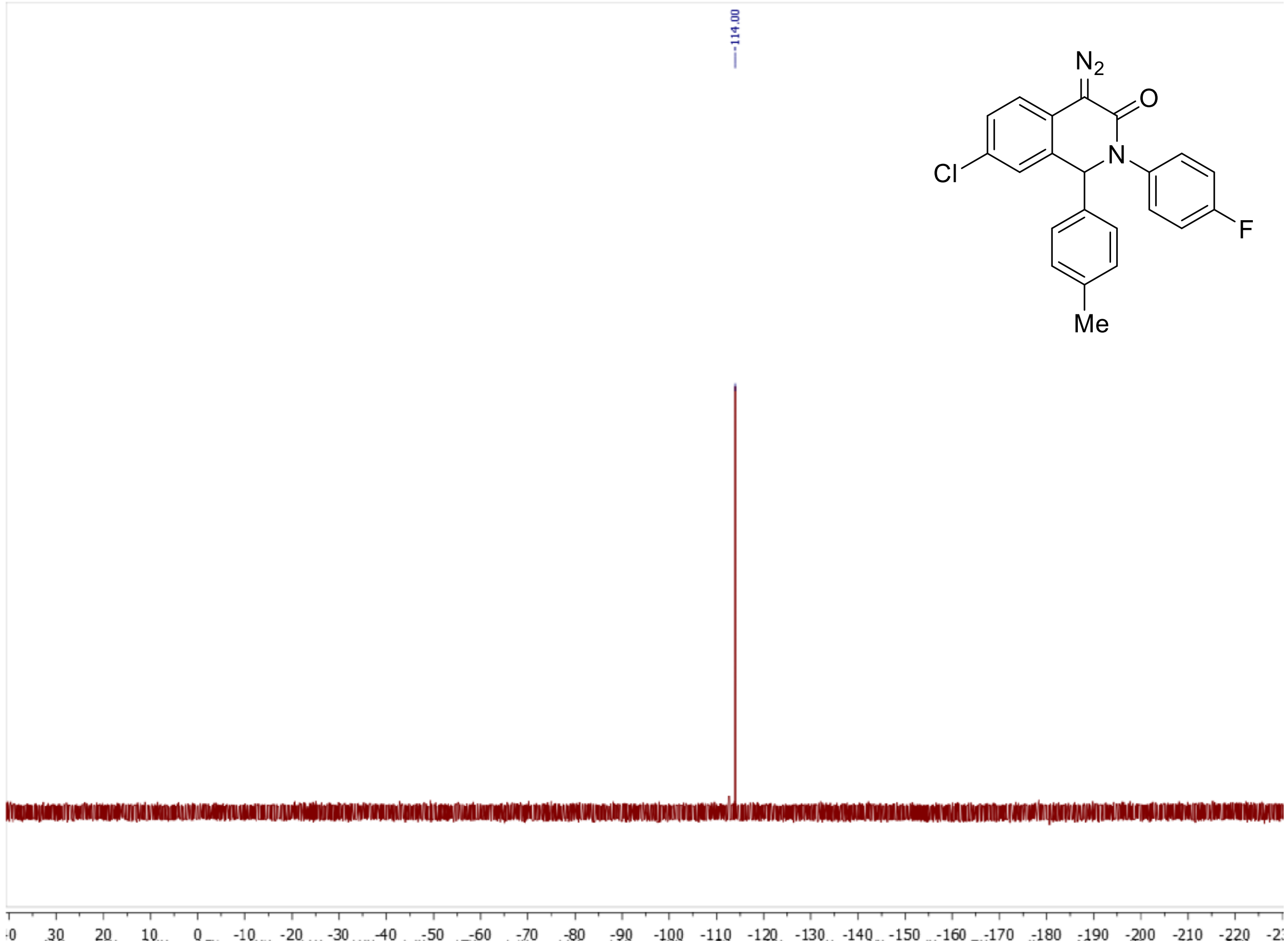

Copies of ^{1}H (400.13 MHz, $CDCl_3$) and $^{13}C\{^{1}H\}$ (100.61 MHz, $CDCl_3$) spectra of **10n**

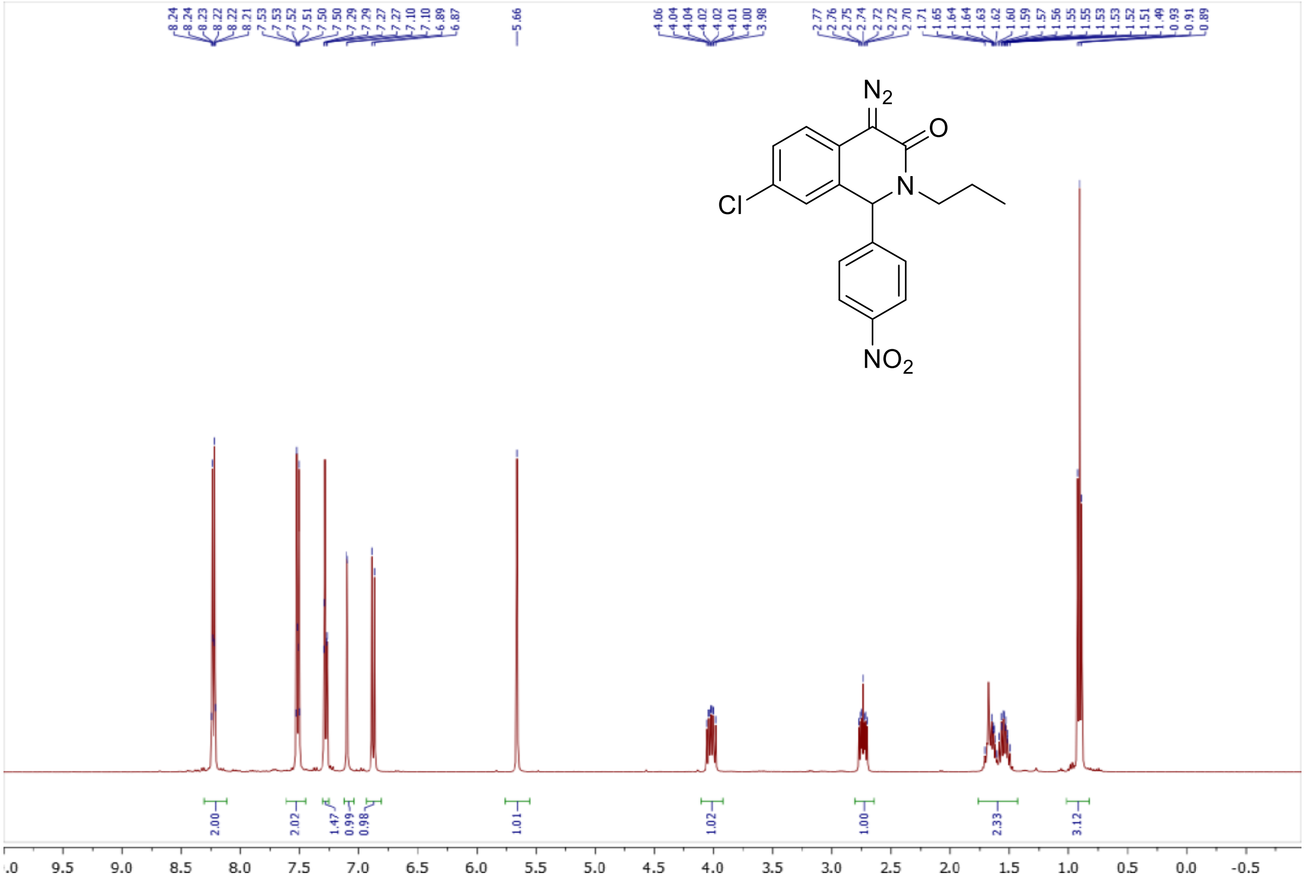

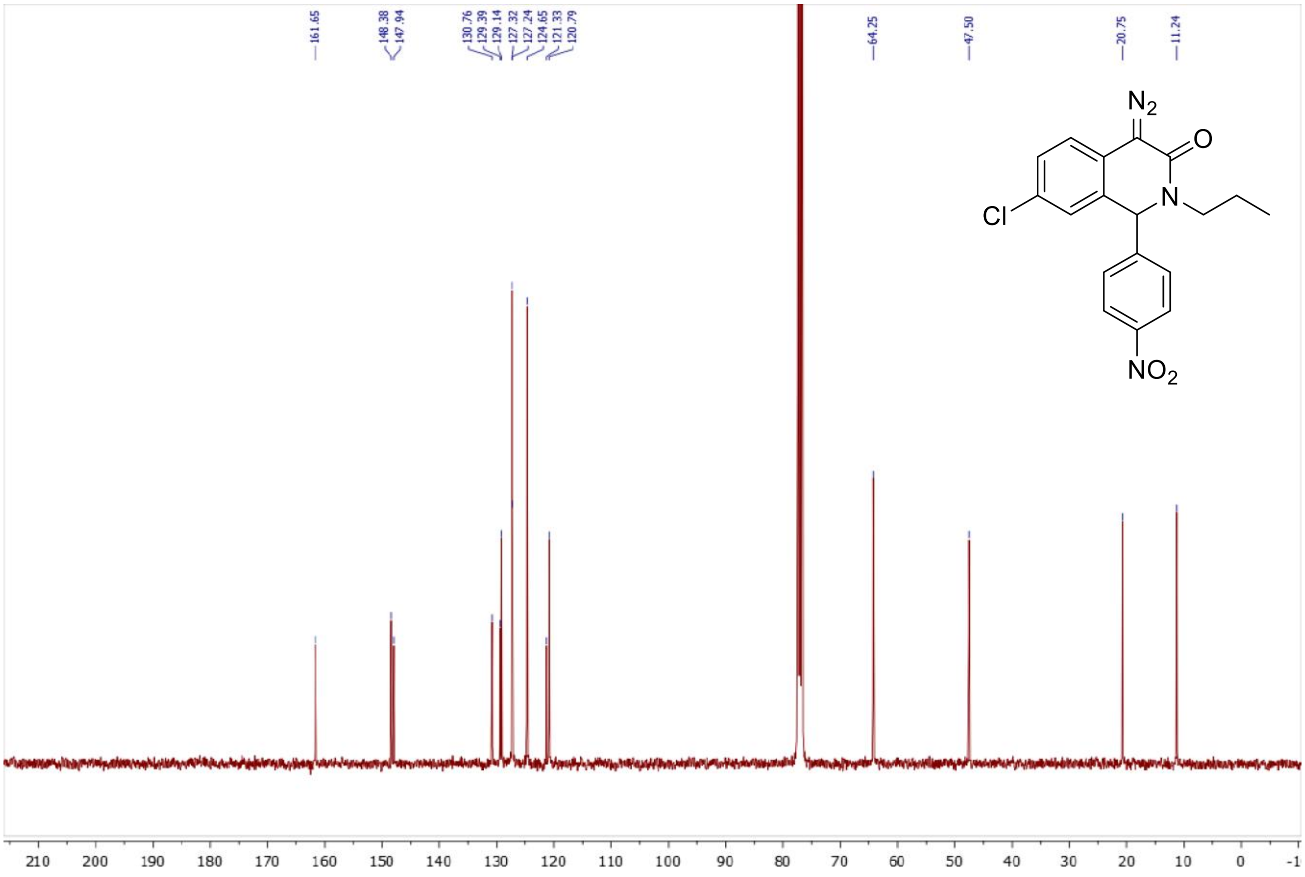

Copies of ^{1}H (400.13 MHz, $CDCl_3$) and $^{13}C\{^{1}H\}$ (100.61 MHz, $CDCl_3$) spectra of **10o**

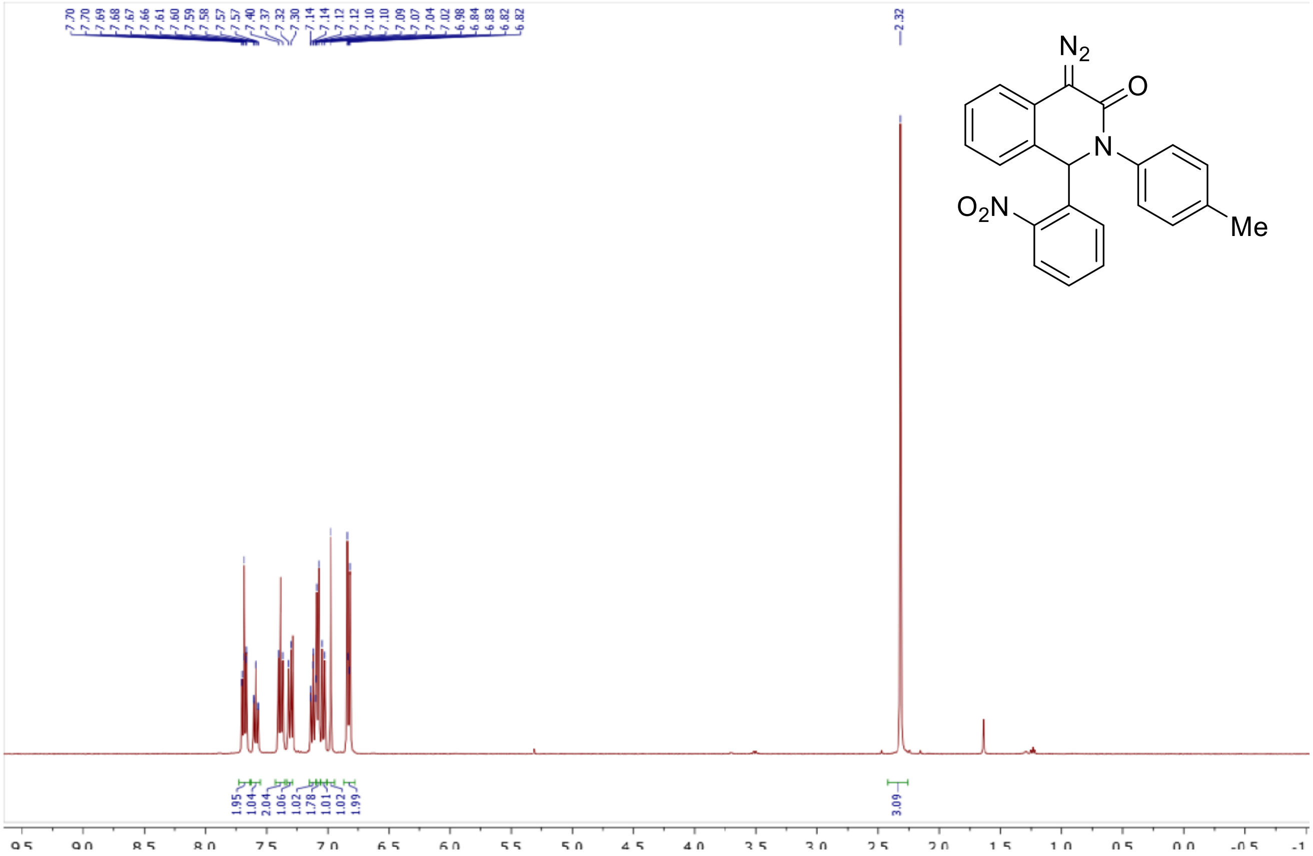

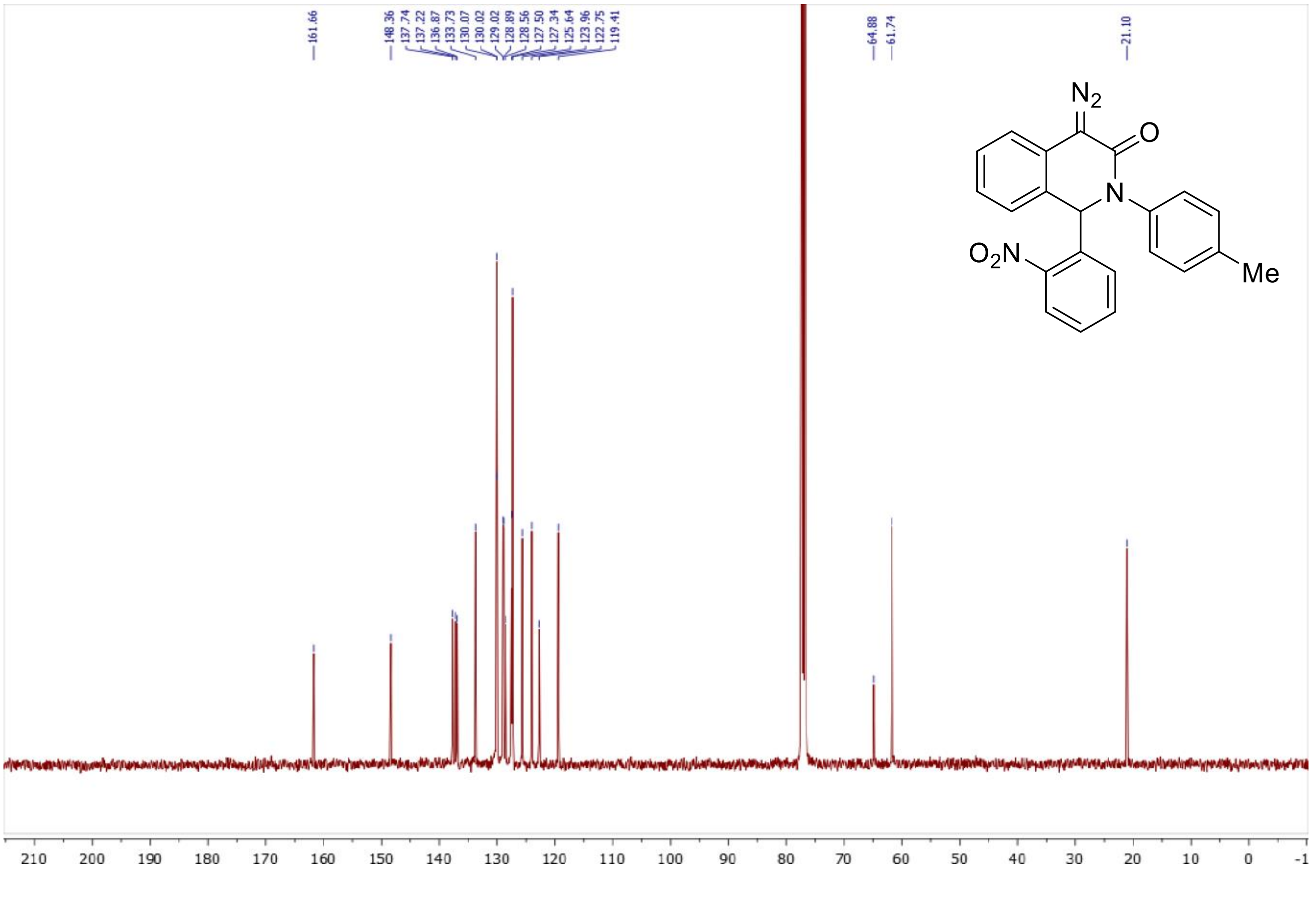

Copies of ^{1}H (400.13 MHz, $CDCl_3$) and ^{13}C{^{1}H} (100.61 MHz, $CDCl_3$) spectra of **10p**

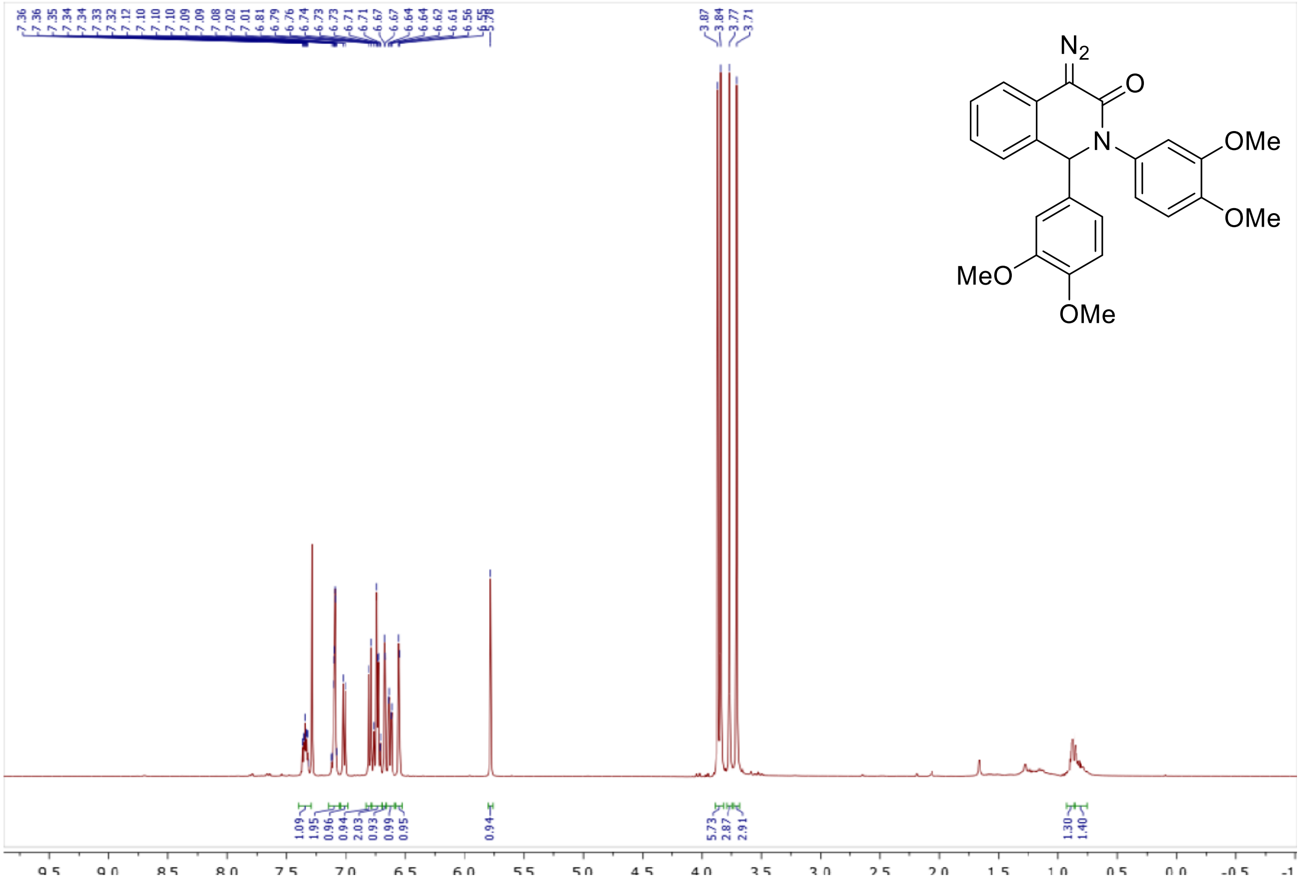

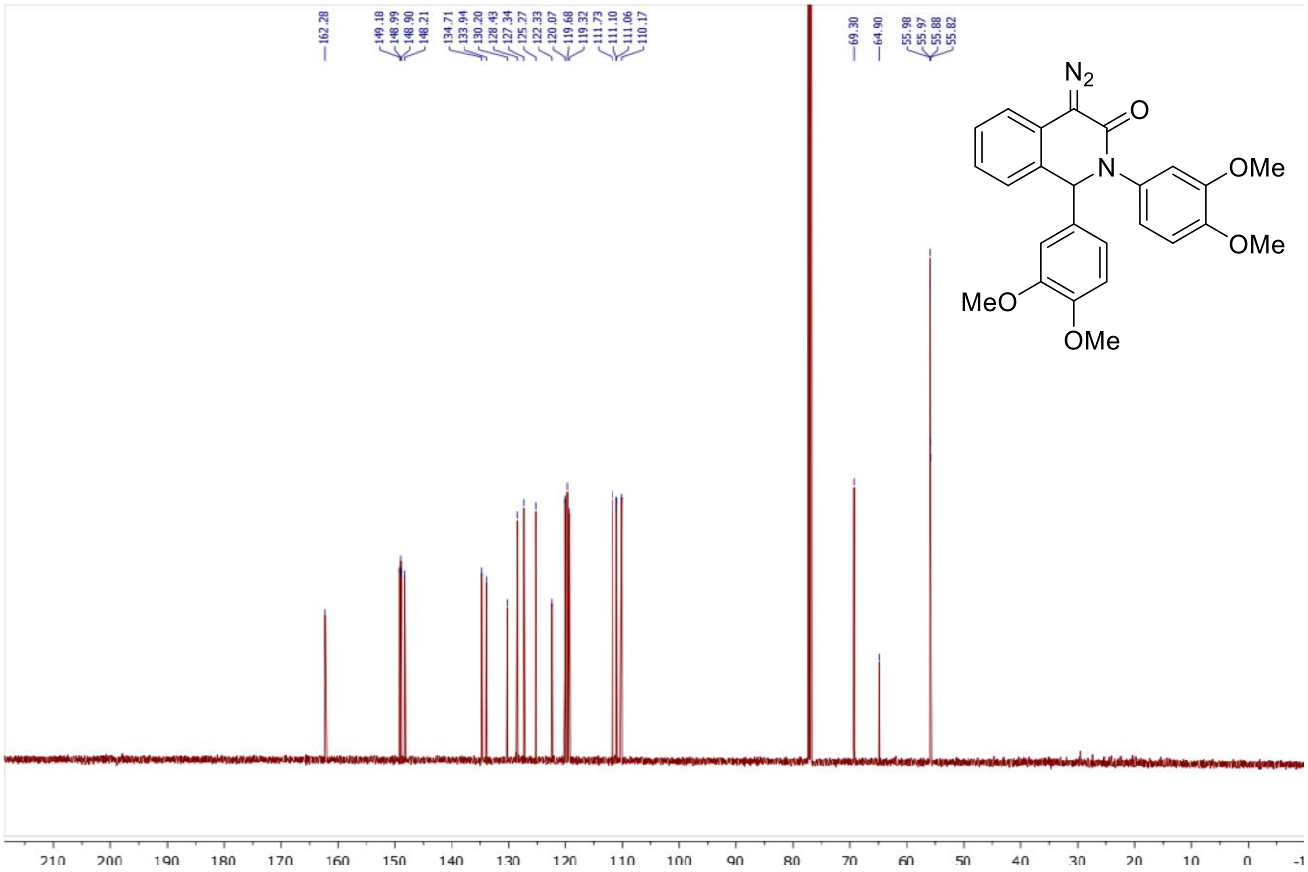

Copies of ^{1}H (400.13 MHz, $CDCl_3$) and $^{13}C\{^{1}H\}$ (100.61 MHz, $CDCl_3$) spectra of **10q**

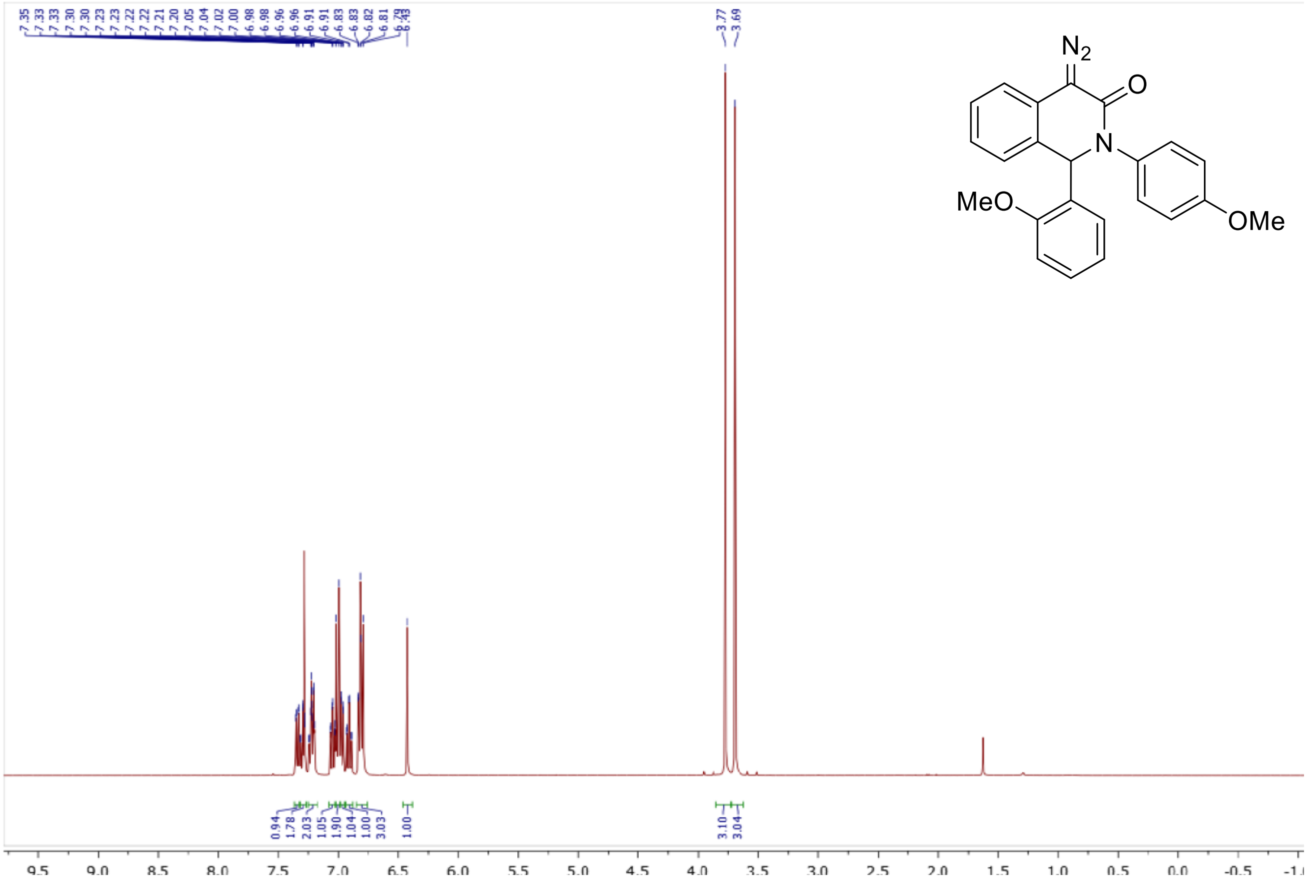

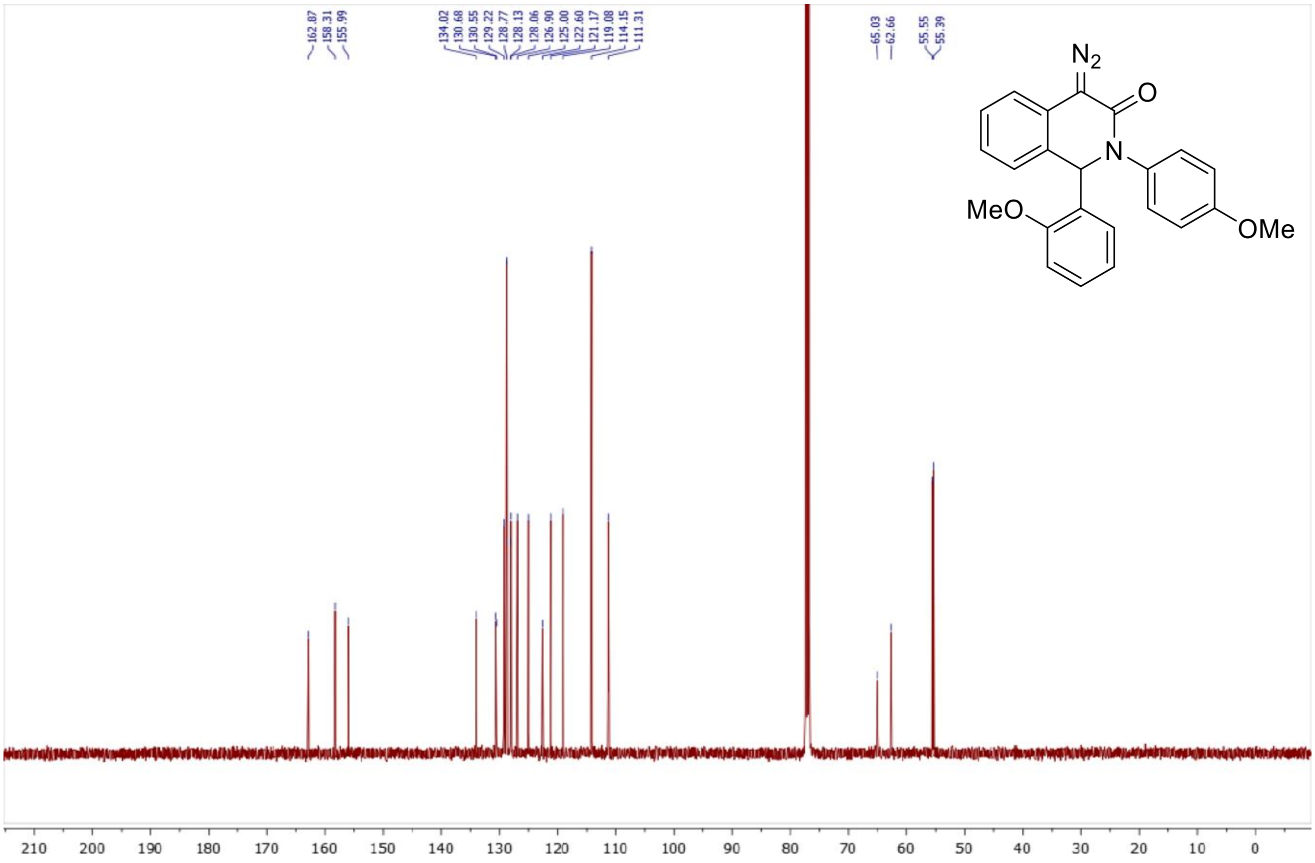

Copies of ^{1}H (400.13 MHz, $CDCl_3$) and $^{13}C\{^{1}H\}$ (100.61 MHz, $CDCl_3$) spectra of **10r**

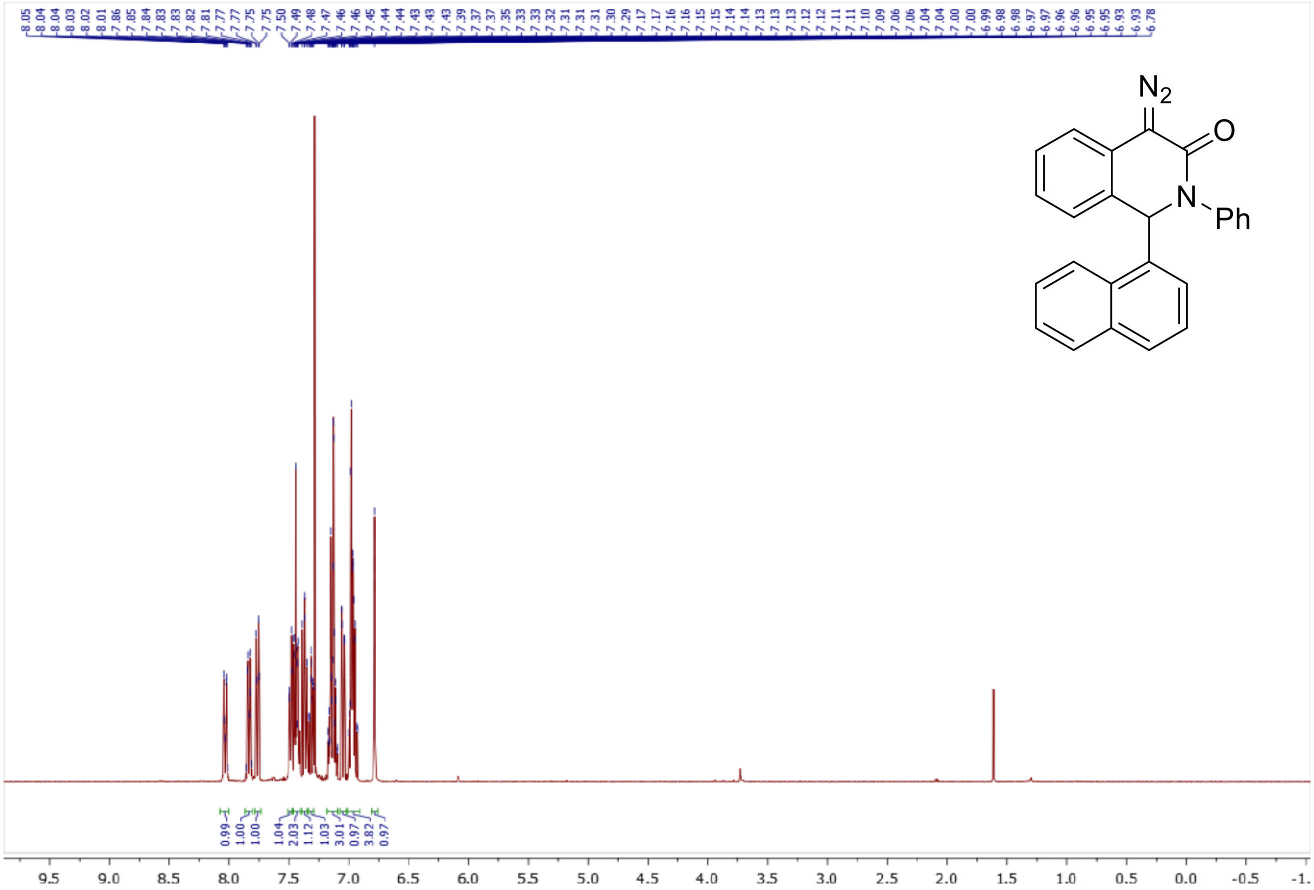

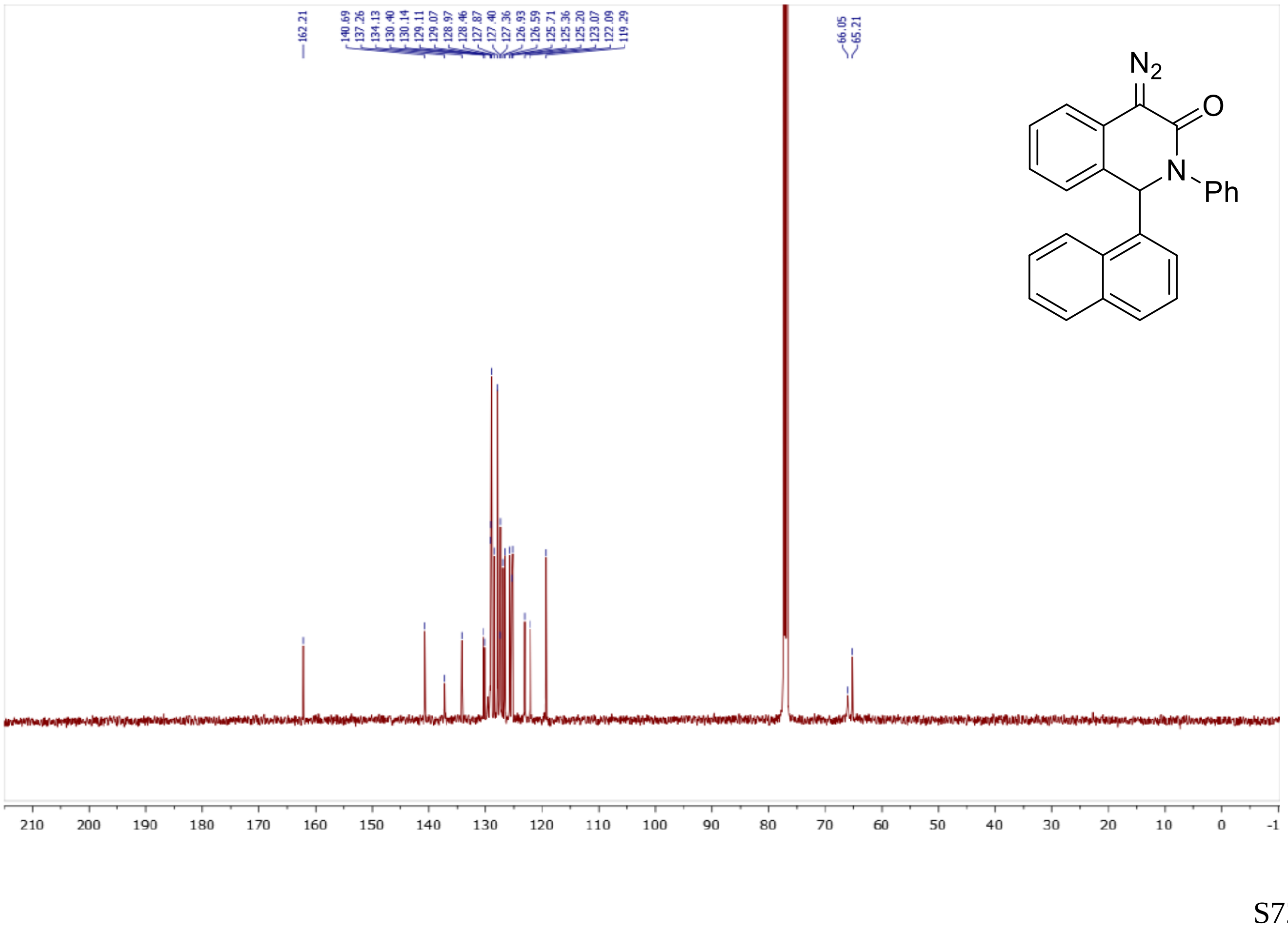

Copies of ^{1}H (400.13 MHz, $CDCl_3$) and $^{13}C\{^{1}H\}$ (100.61 MHz, $CDCl_3$) spectra of **10s**

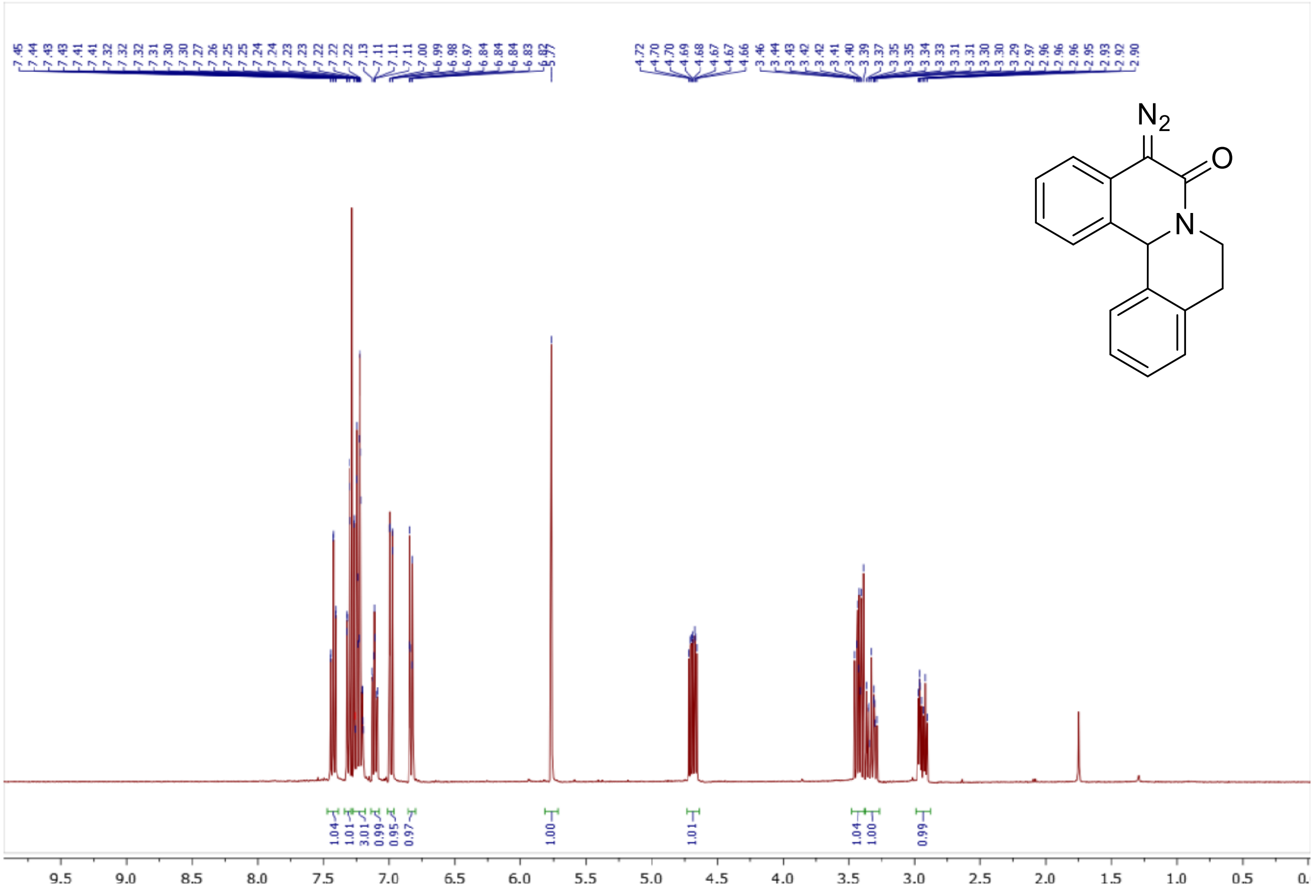

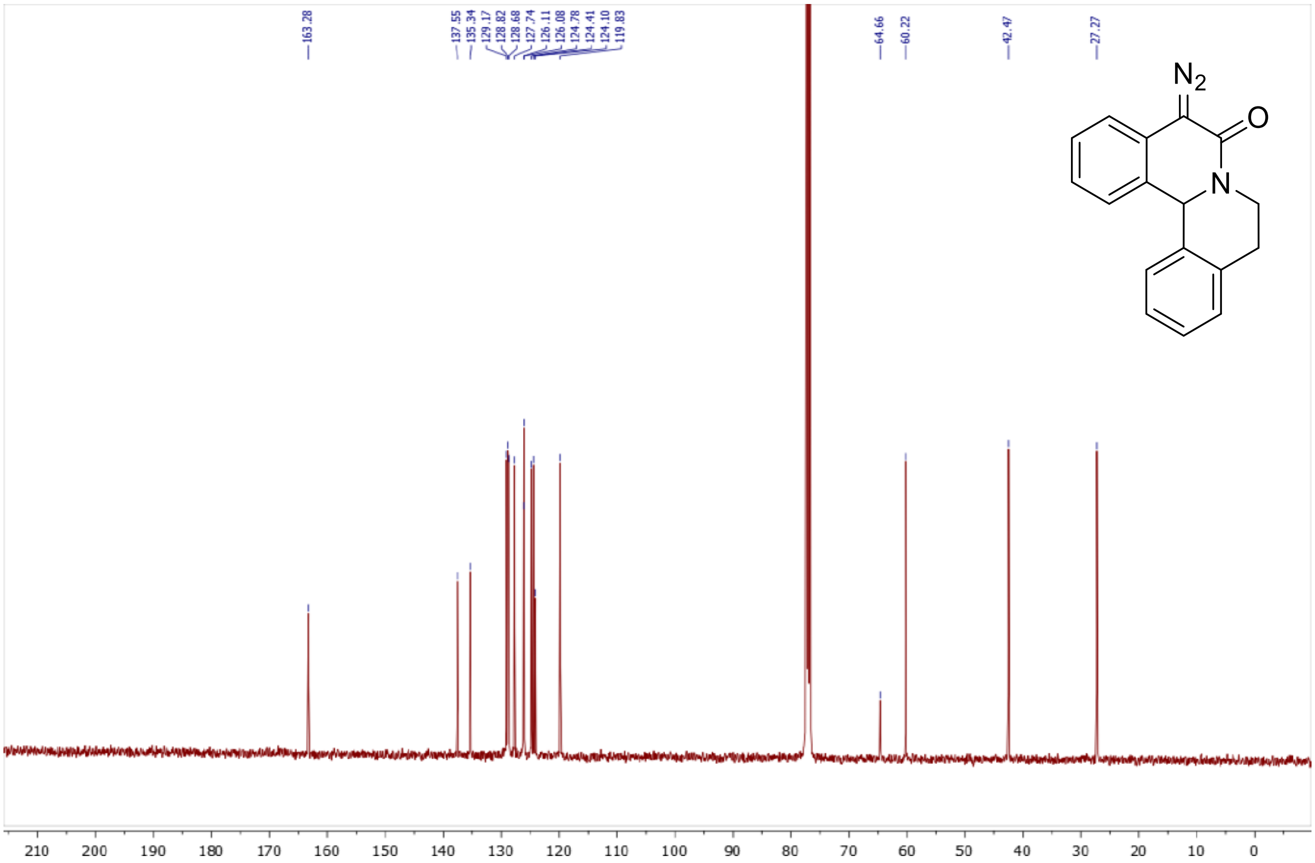

Copies of ^{1}H (400.13 MHz, $CDCl_3$) and $^{13}C\{^1H\}$ (100.61 MHz, $CDCl_3$) spectra of **9a**

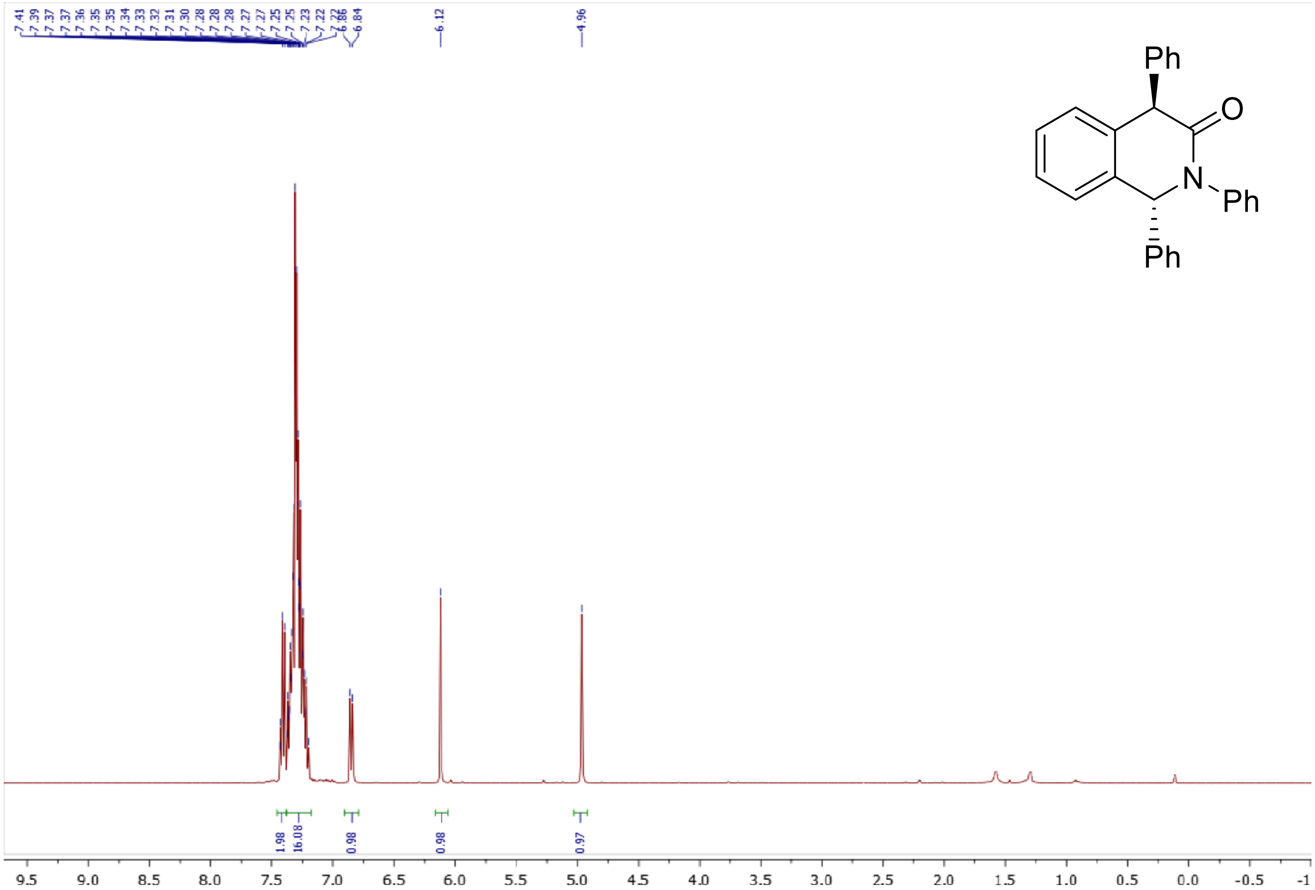

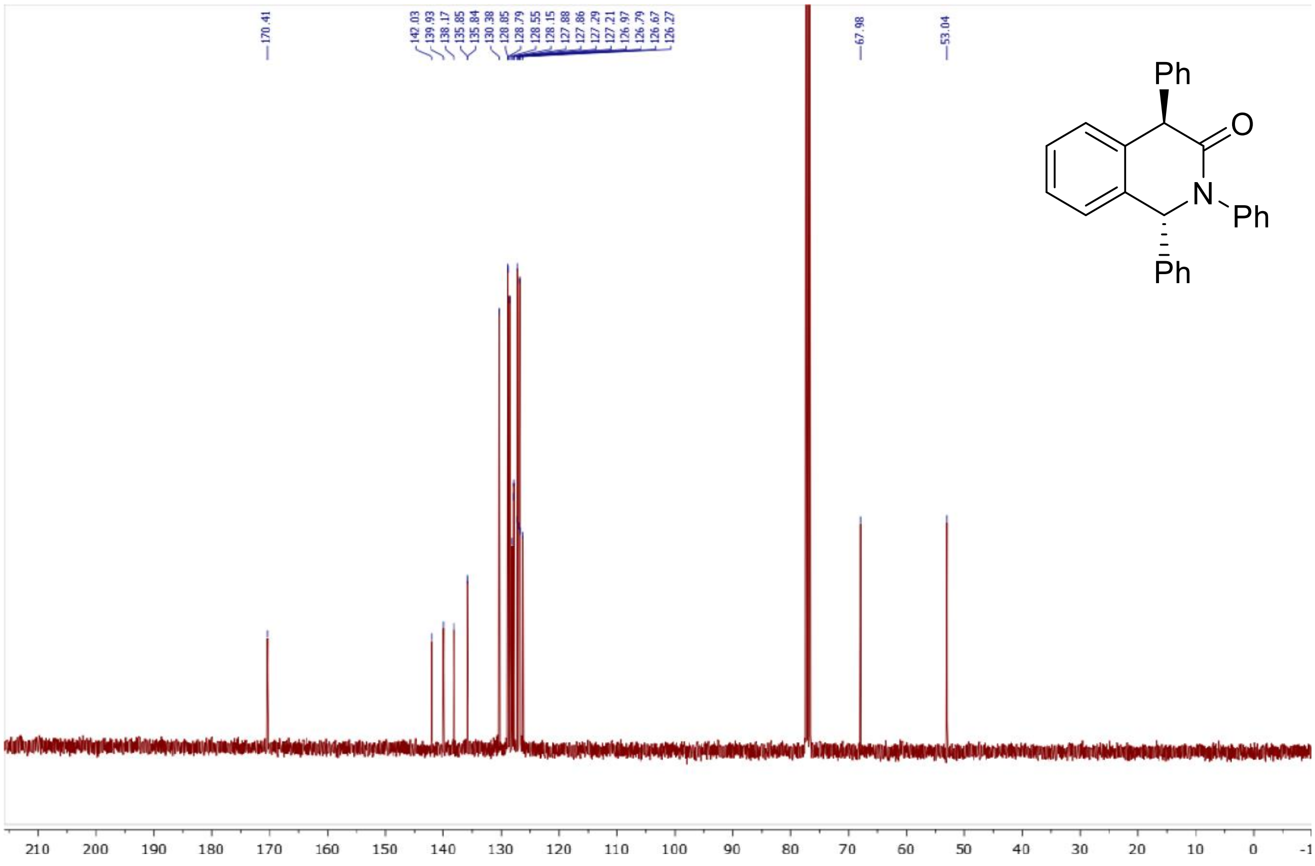

Copies of ^{1}H (400.13 MHz, $CDCl_3$) and $^{13}C\{^{1}H\}$ (100.61 MHz, $CDCl_3$) spectra of **15a**

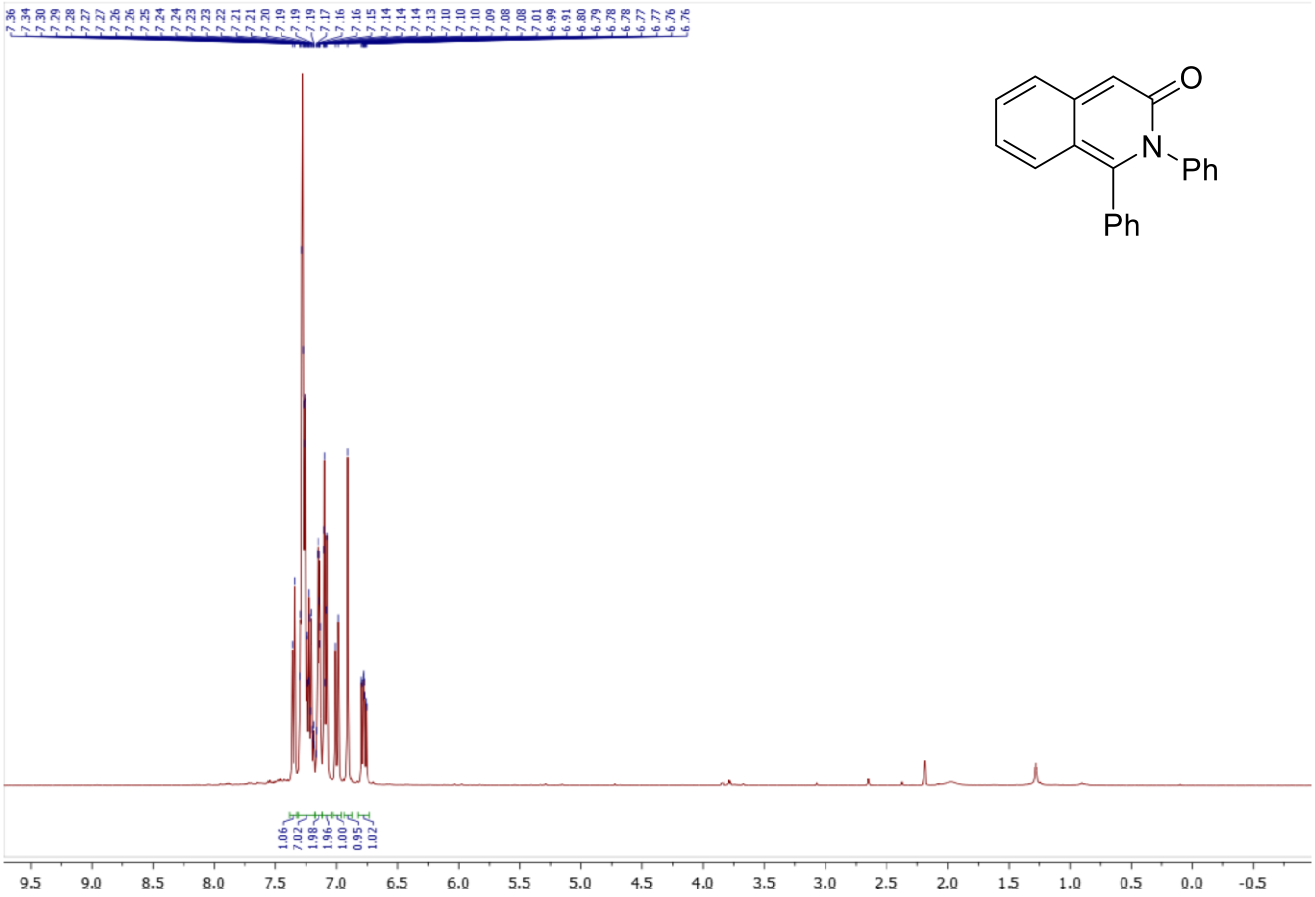

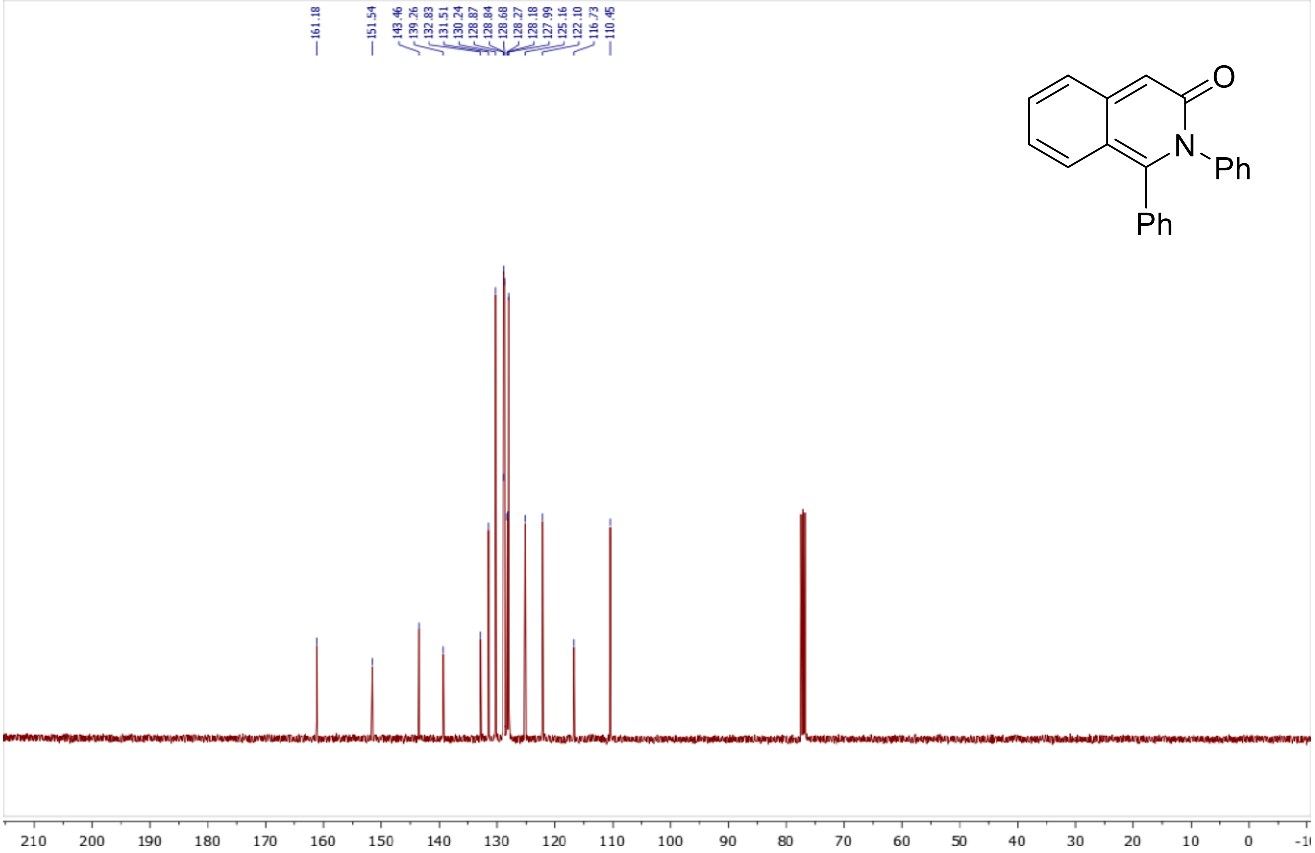

Copies of ^{1}H (400.13 MHz, $CDCl_3$) and $^{13}C\{^{1}H\}$ (100.61 MHz, $CDCl_3$) spectra of **9b**

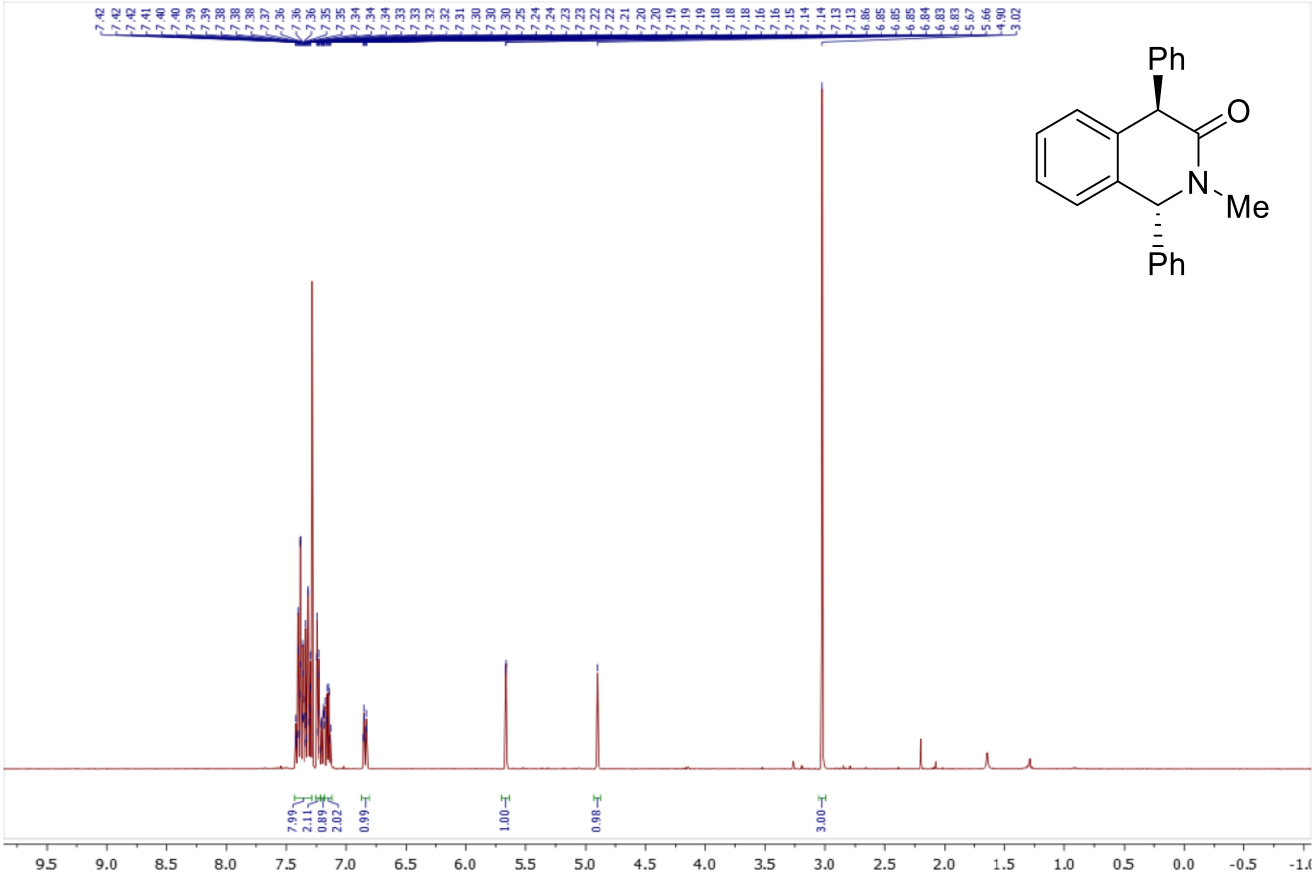

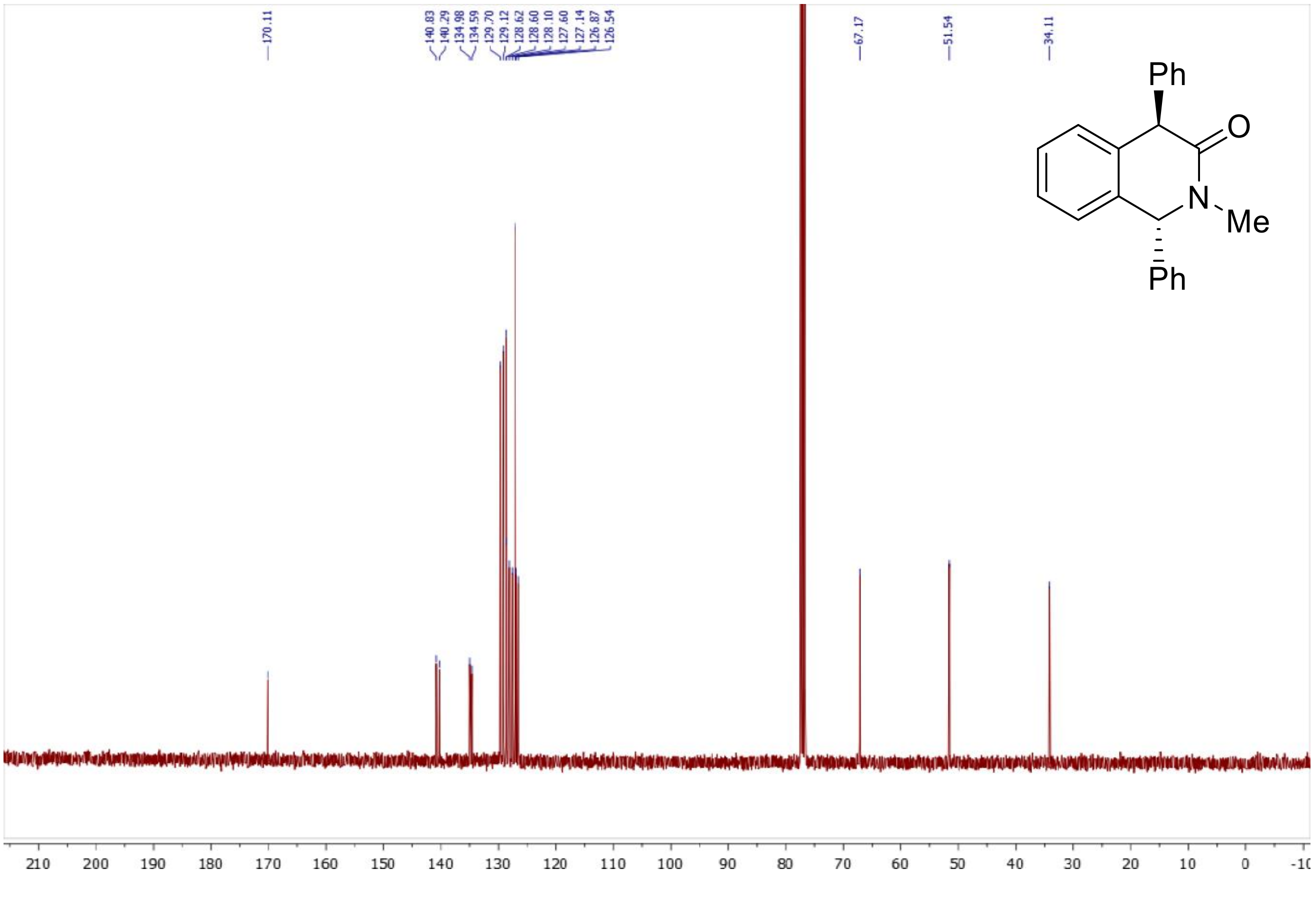

Copies of ^{1}H (400.13 MHz, $CDCl_3$) and $^{13}C\{^{1}H\}$ (100.61 MHz, $CDCl_3$) spectra of **9c**

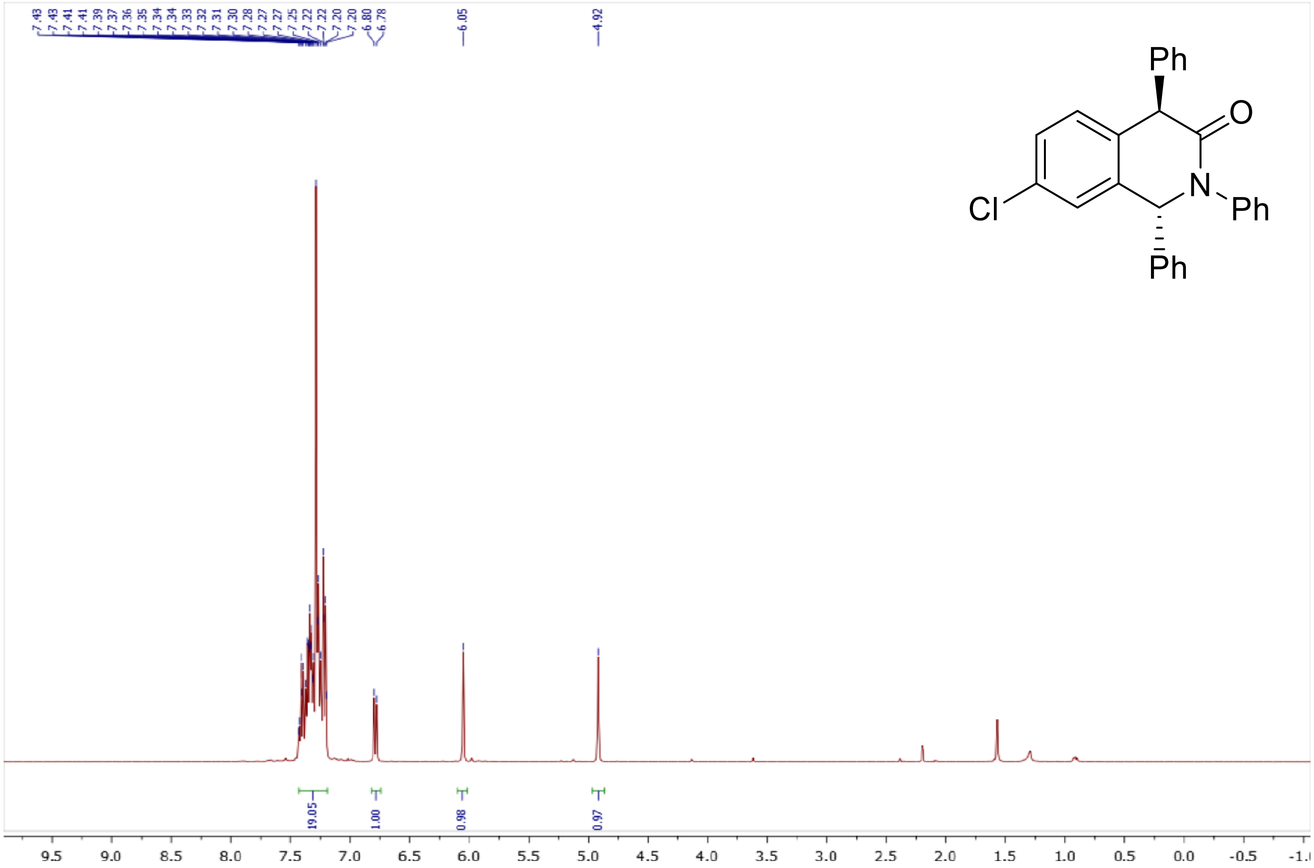

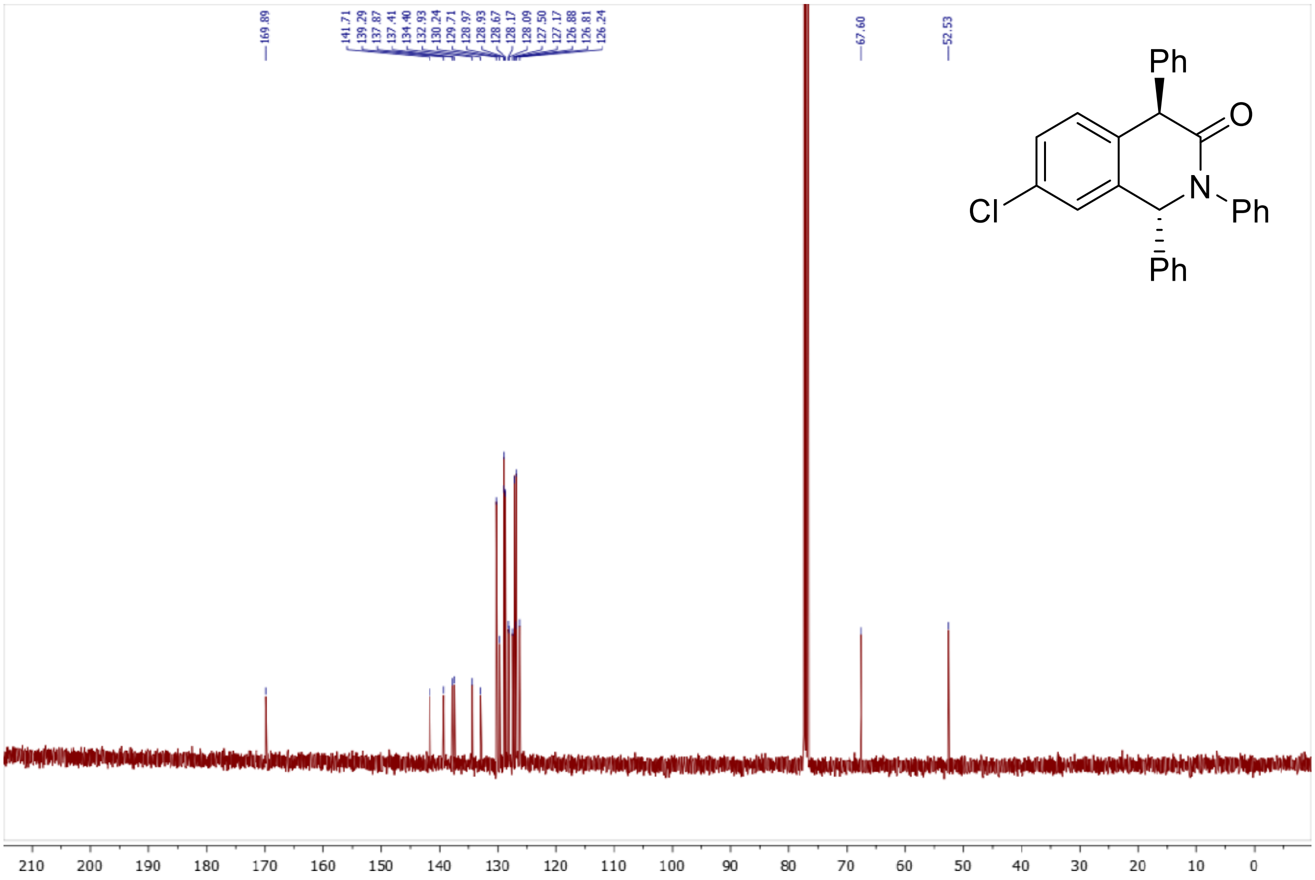

Copies of ^{1}H (400.13 MHz, $CDCl_3$) and $^{13}C\{^{1}H\}$ (100.61 MHz, $CDCl_3$) spectra of **15c**

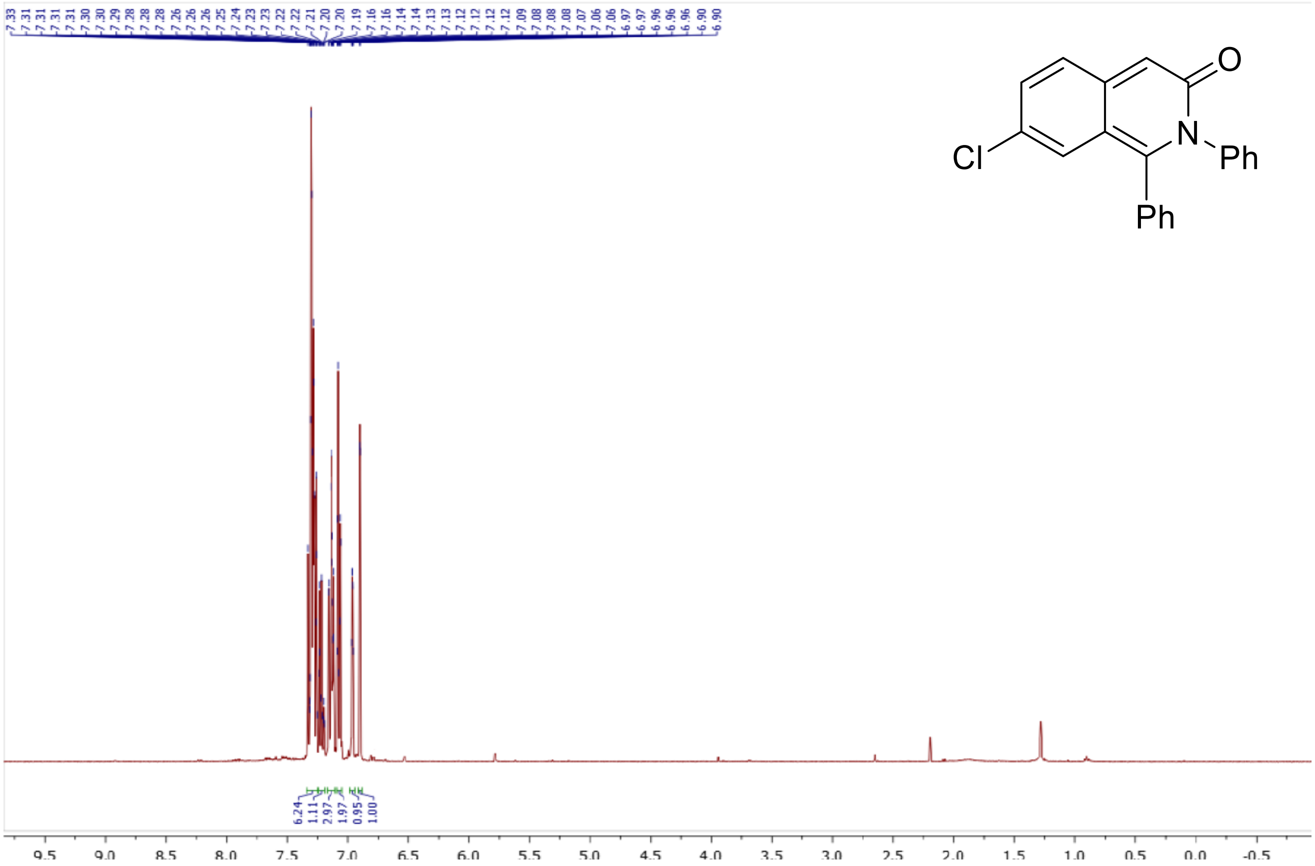

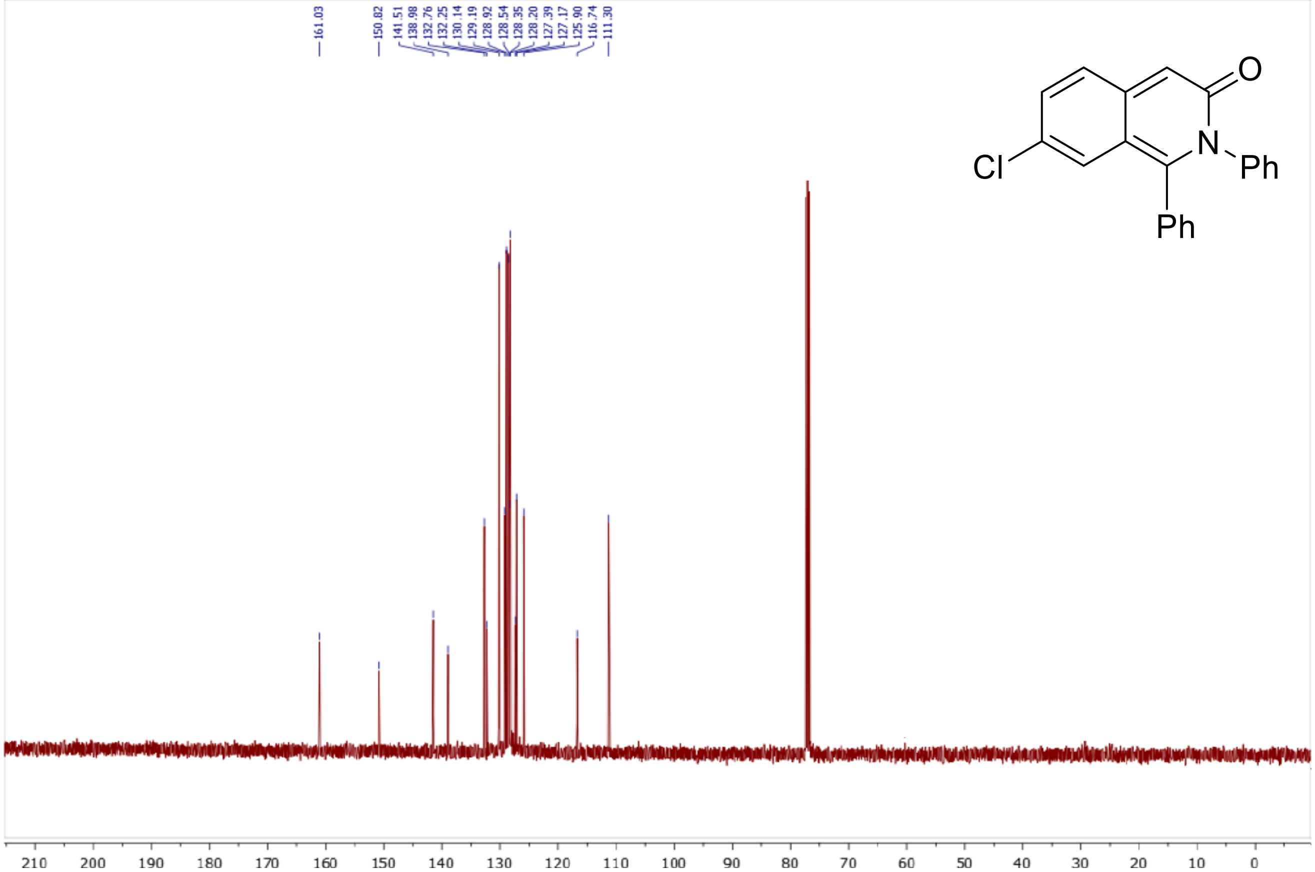

Copies of ^{1}H (400.13 MHz, $CDCl_3$) and $^{13}C\{^{1}H\}$ (100.61 MHz, $CDCl_3$) spectra of **9d**

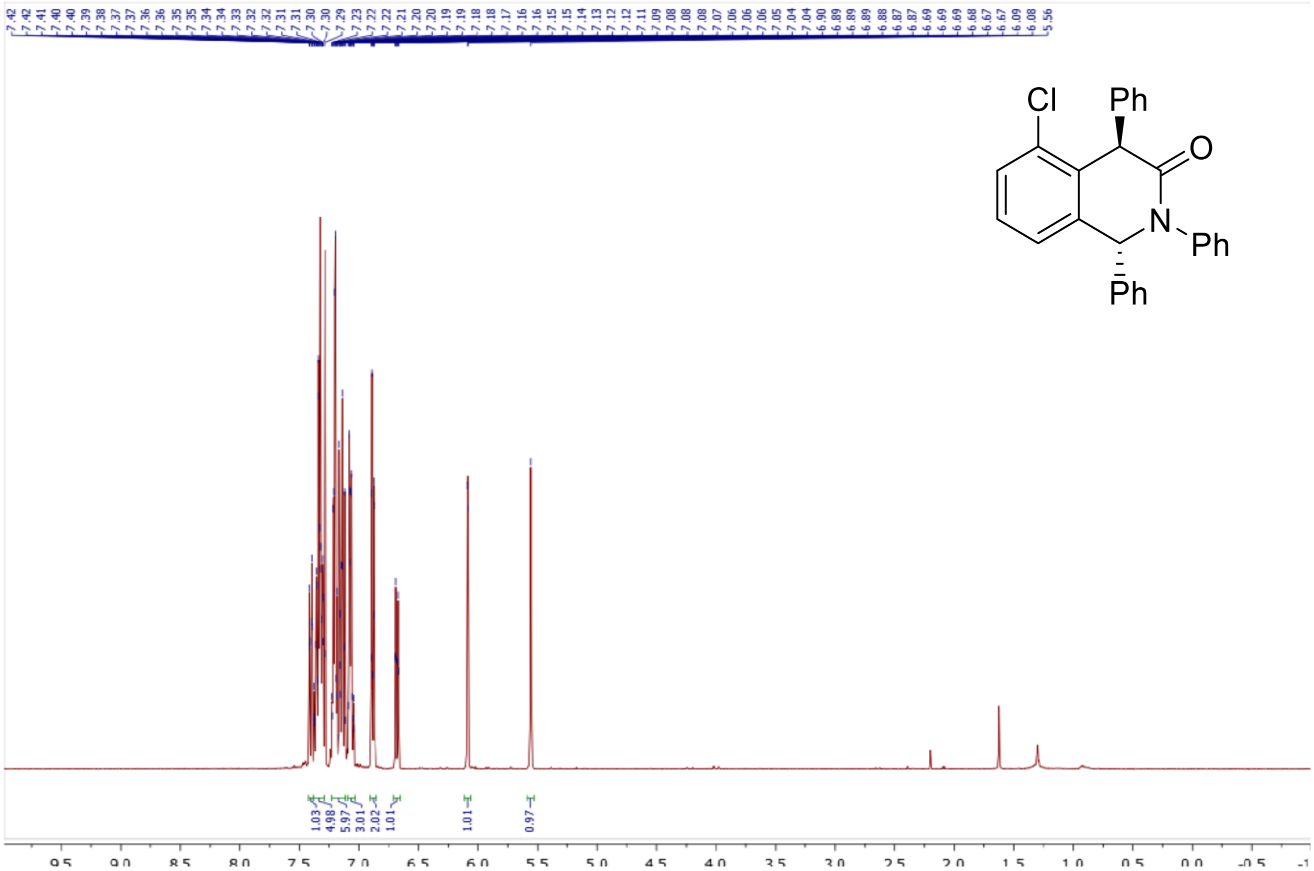

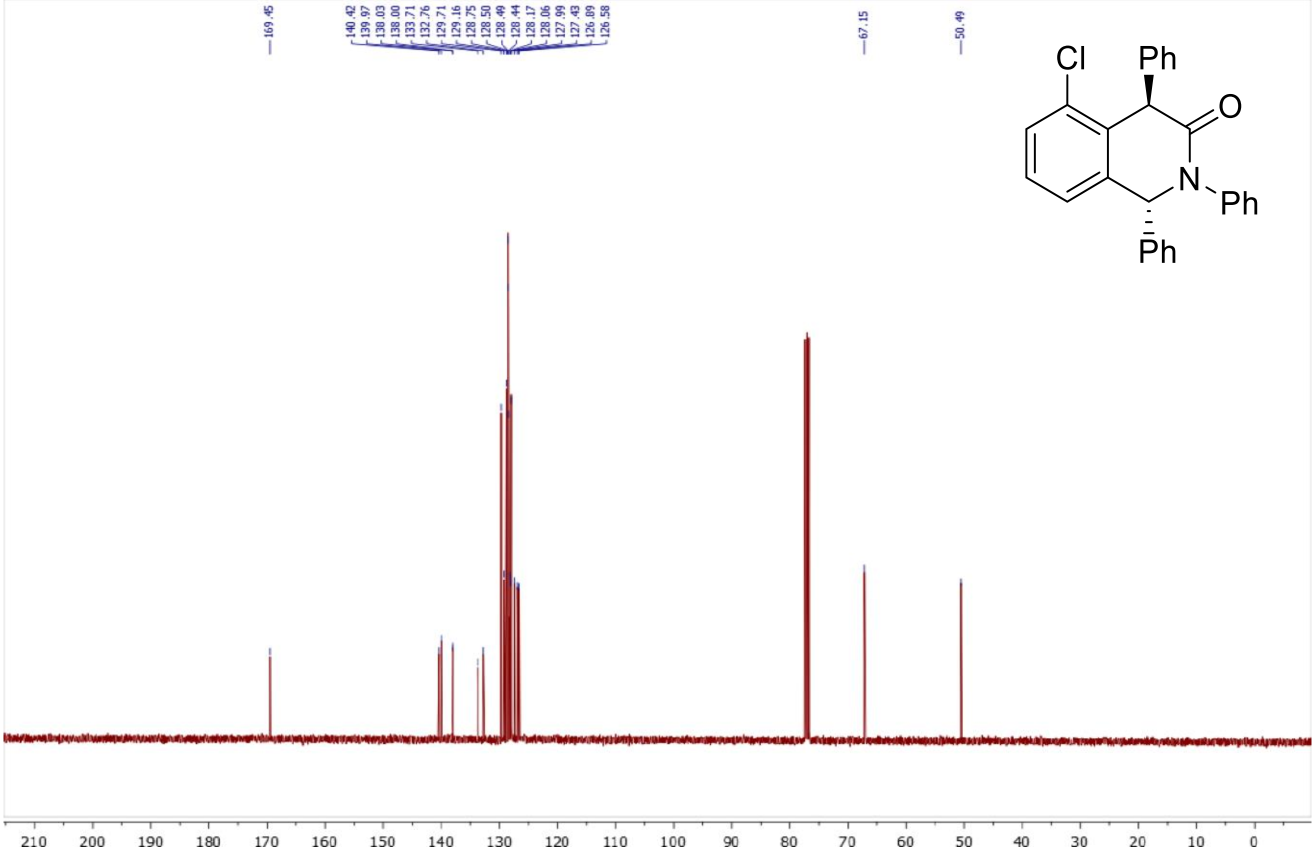

Copies of ^{1}H (400.13 MHz, $CDCl_3$) and $^{13}C\{^{1}H\}$ (100.61 MHz, $CDCl_3$) spectra of **9e**

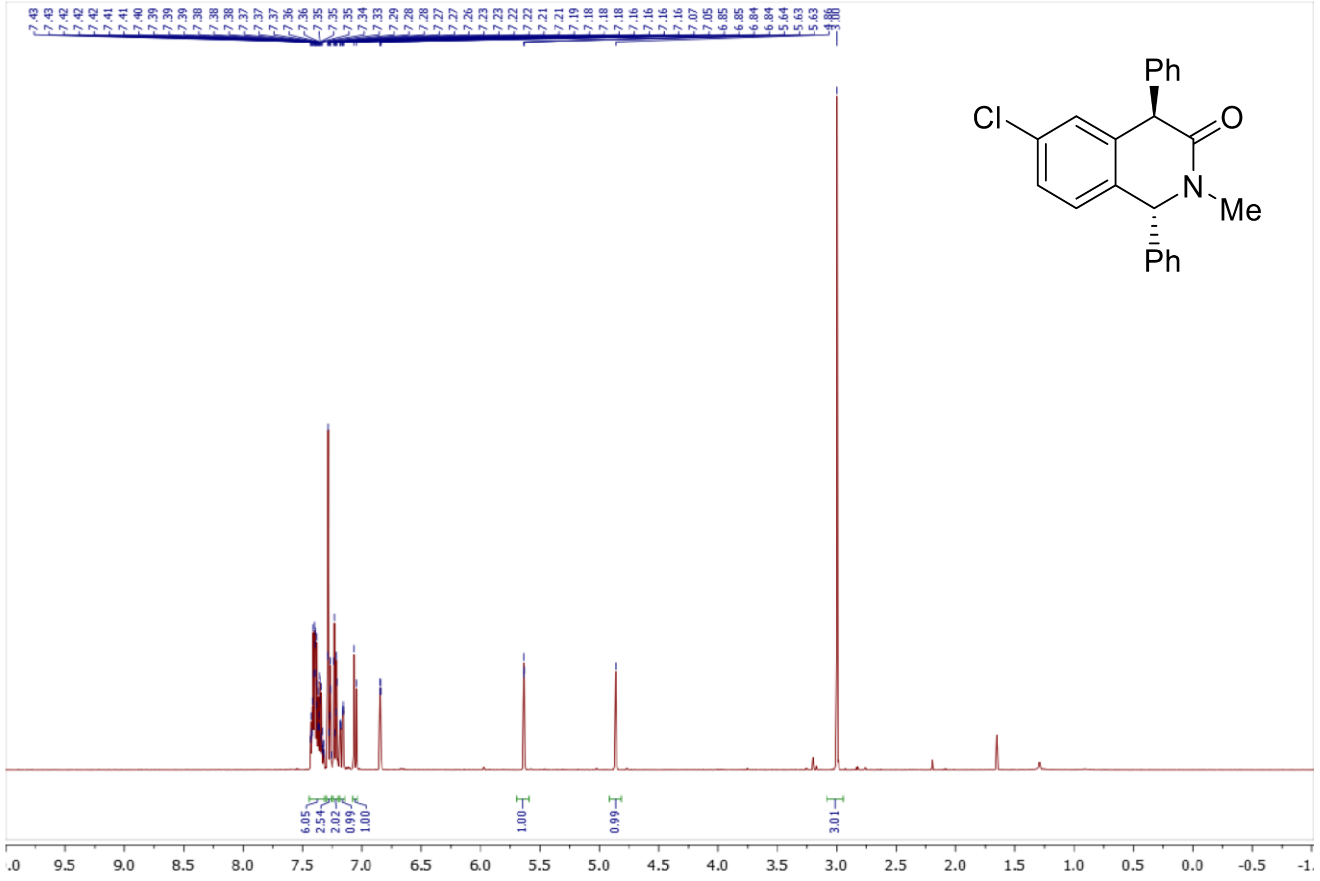

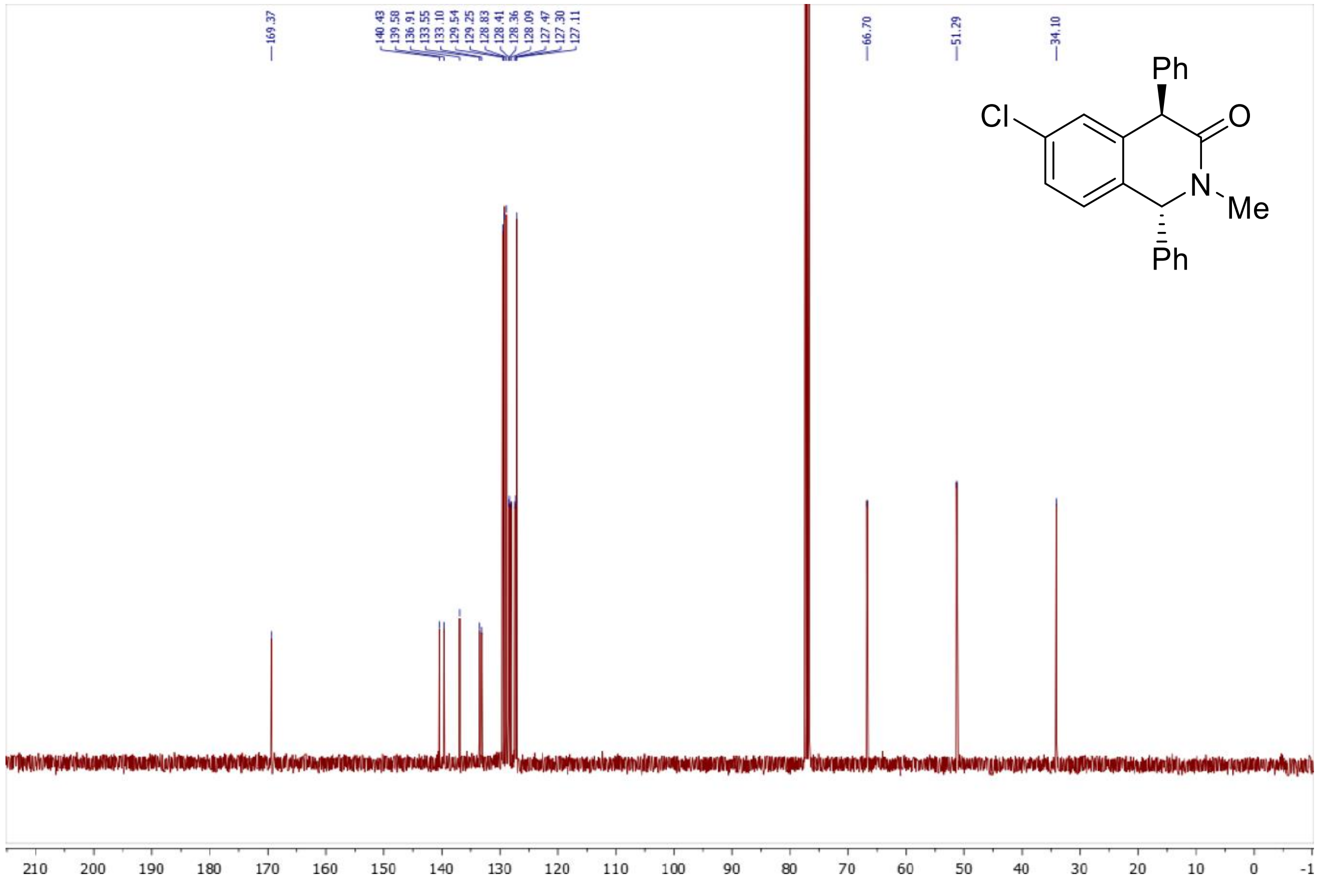

Copies of ^{1}H (400.13 MHz, $CDCl_3$) and $^{13}C\{^{1}H\}$ (100.61 MHz, $CDCl_3$) spectra of **15f**

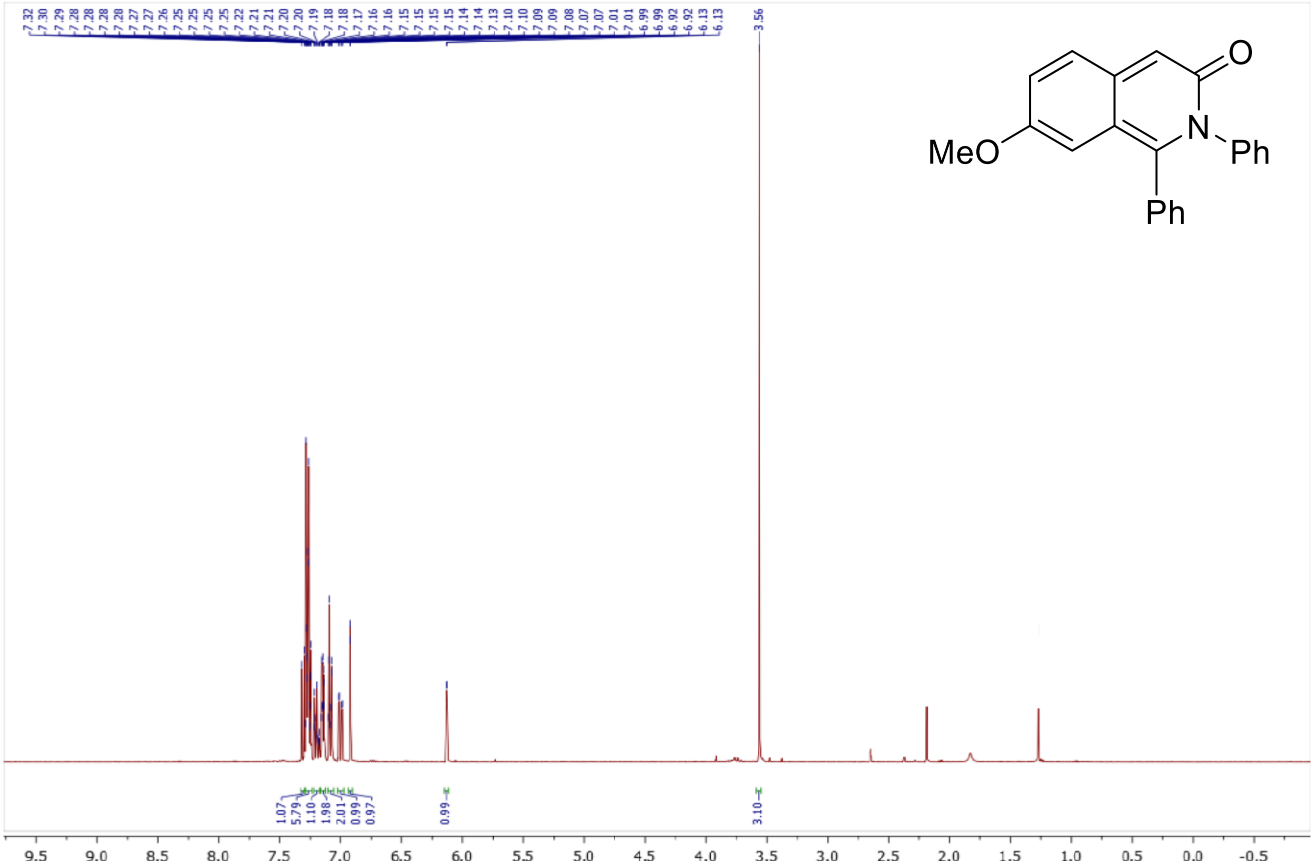

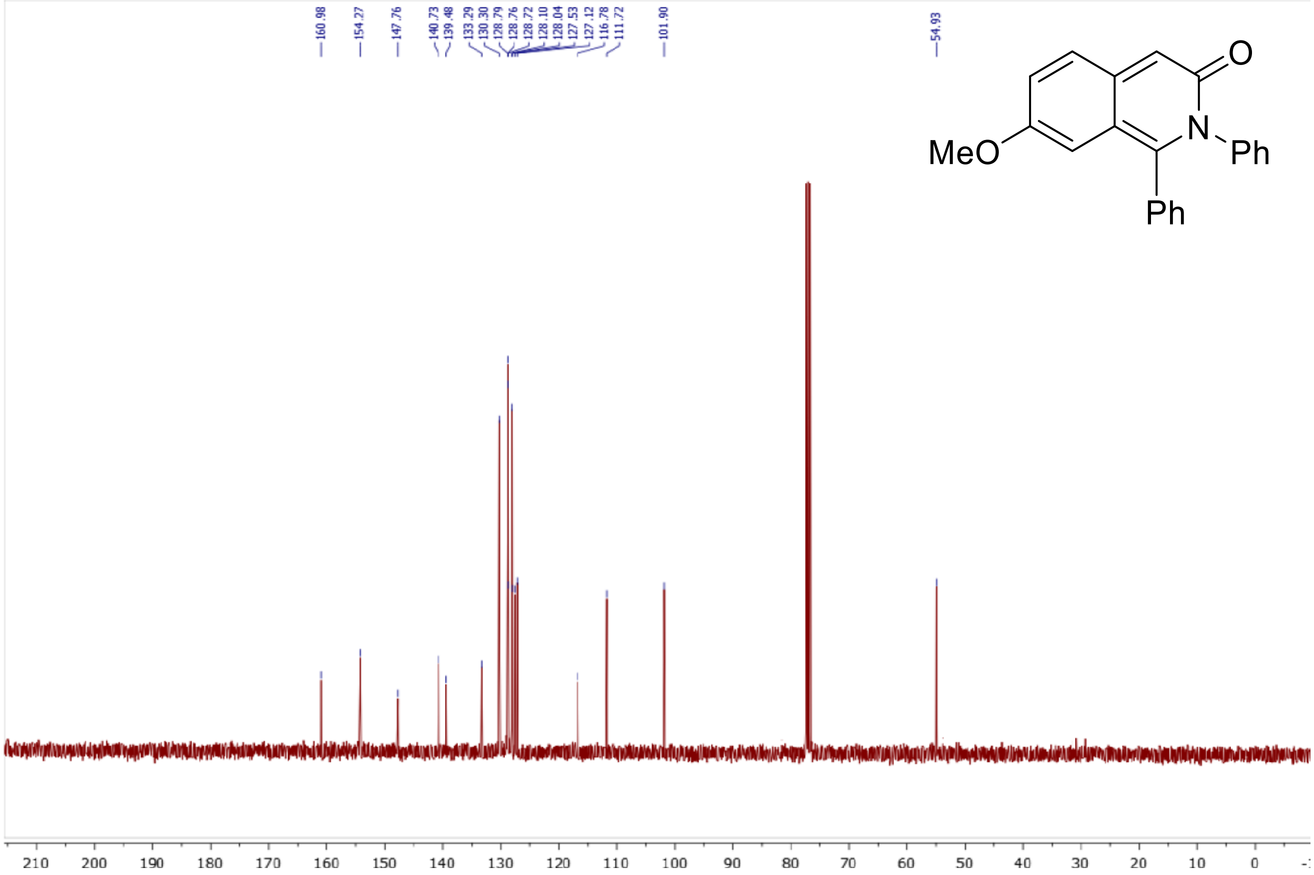

Copies of ^{1}H (400.13 MHz, $CDCl_3$) and $^{13}C\{^{1}H\}$ (100.61 MHz, $CDCl_3$) spectra of **9g**

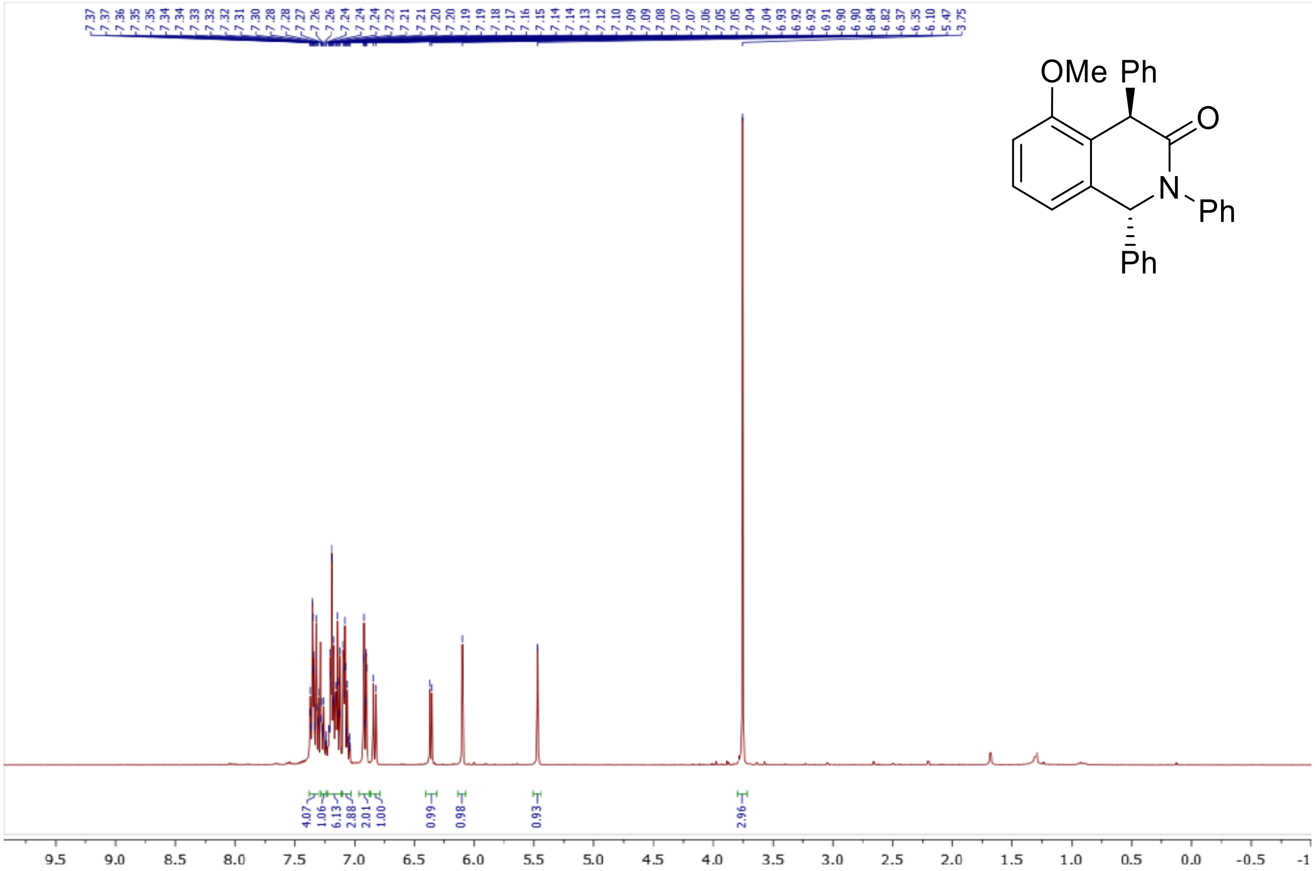

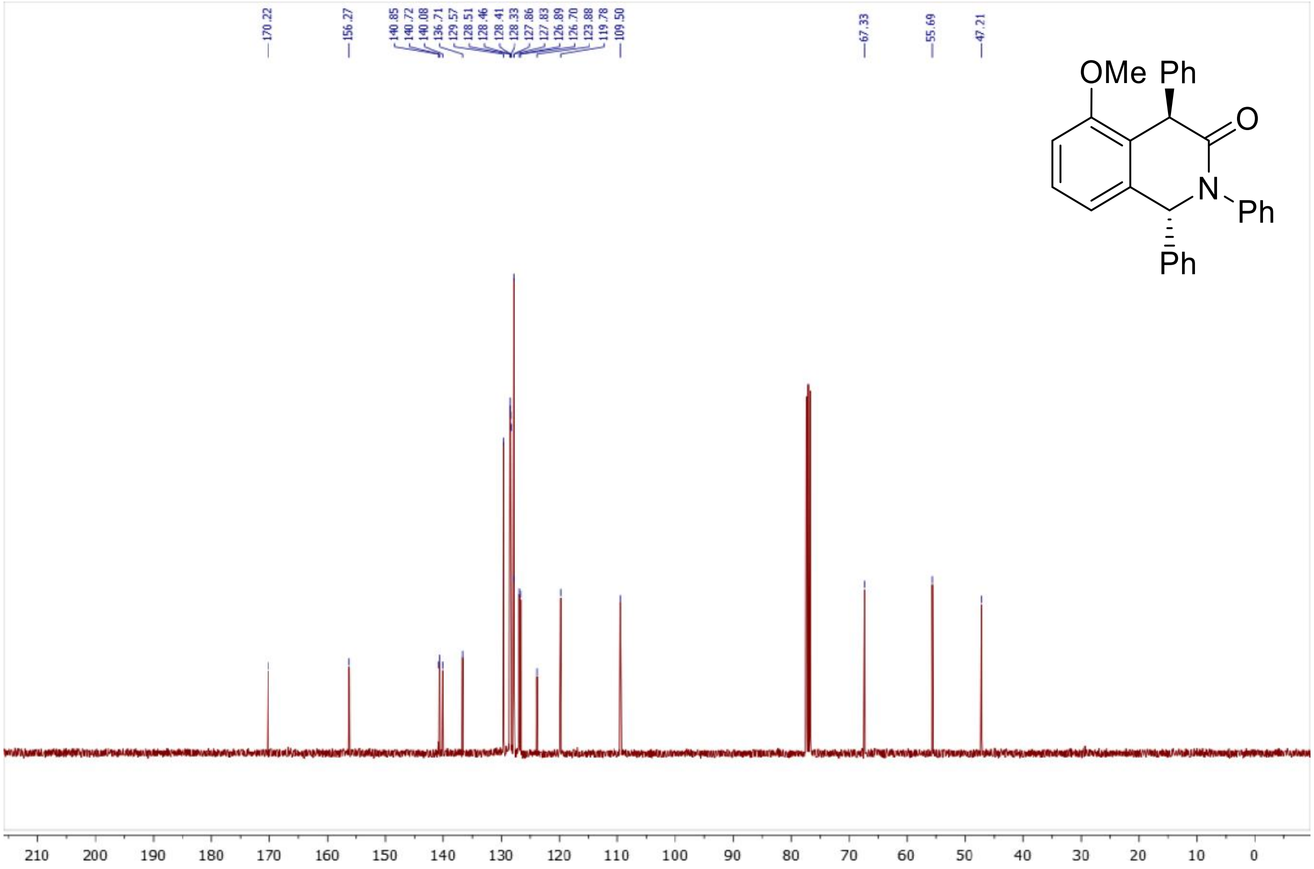

Copies of ^{1}H (400.13 MHz, $CDCl_3$) and $^{13}C\{^{1}H\}$ (100.61 MHz, $CDCl_3$) spectra of **15g**

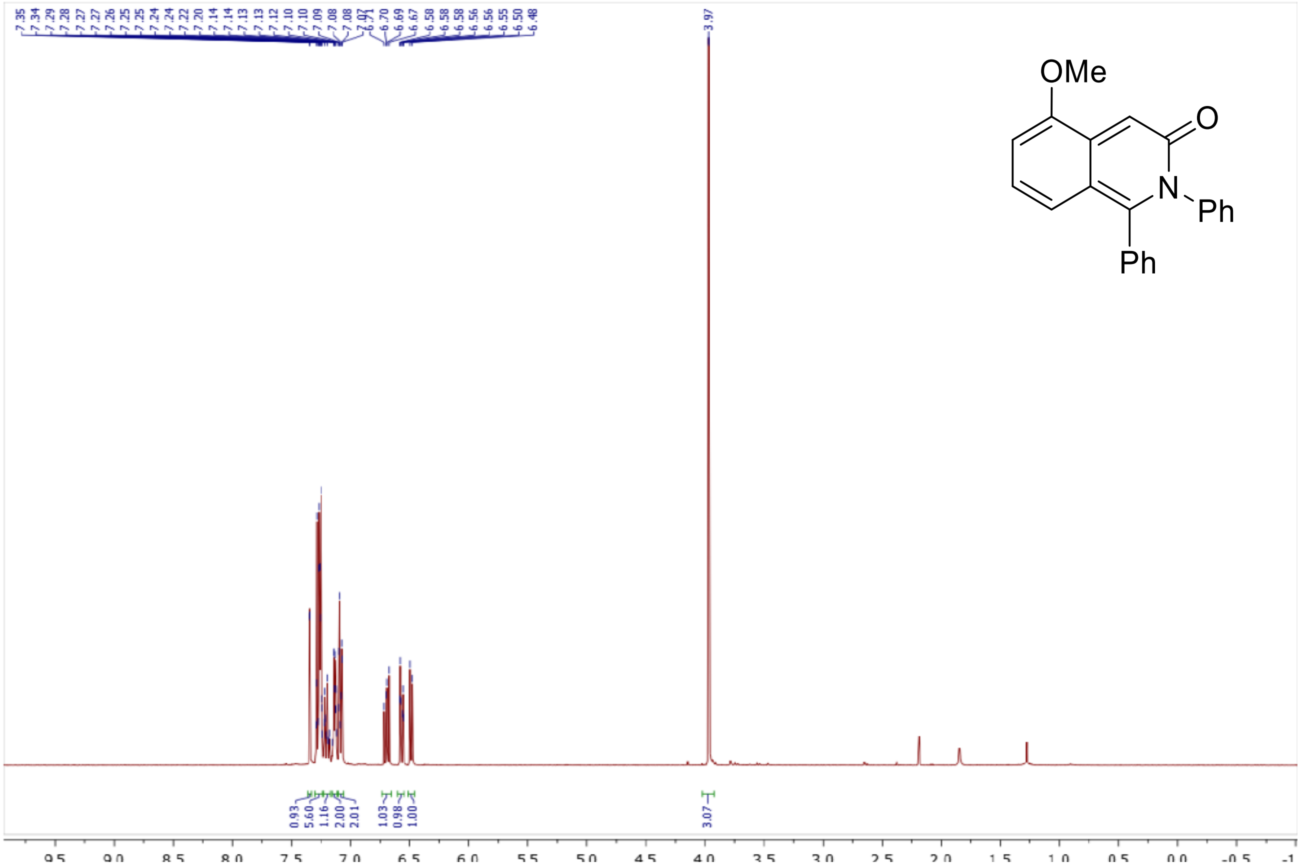

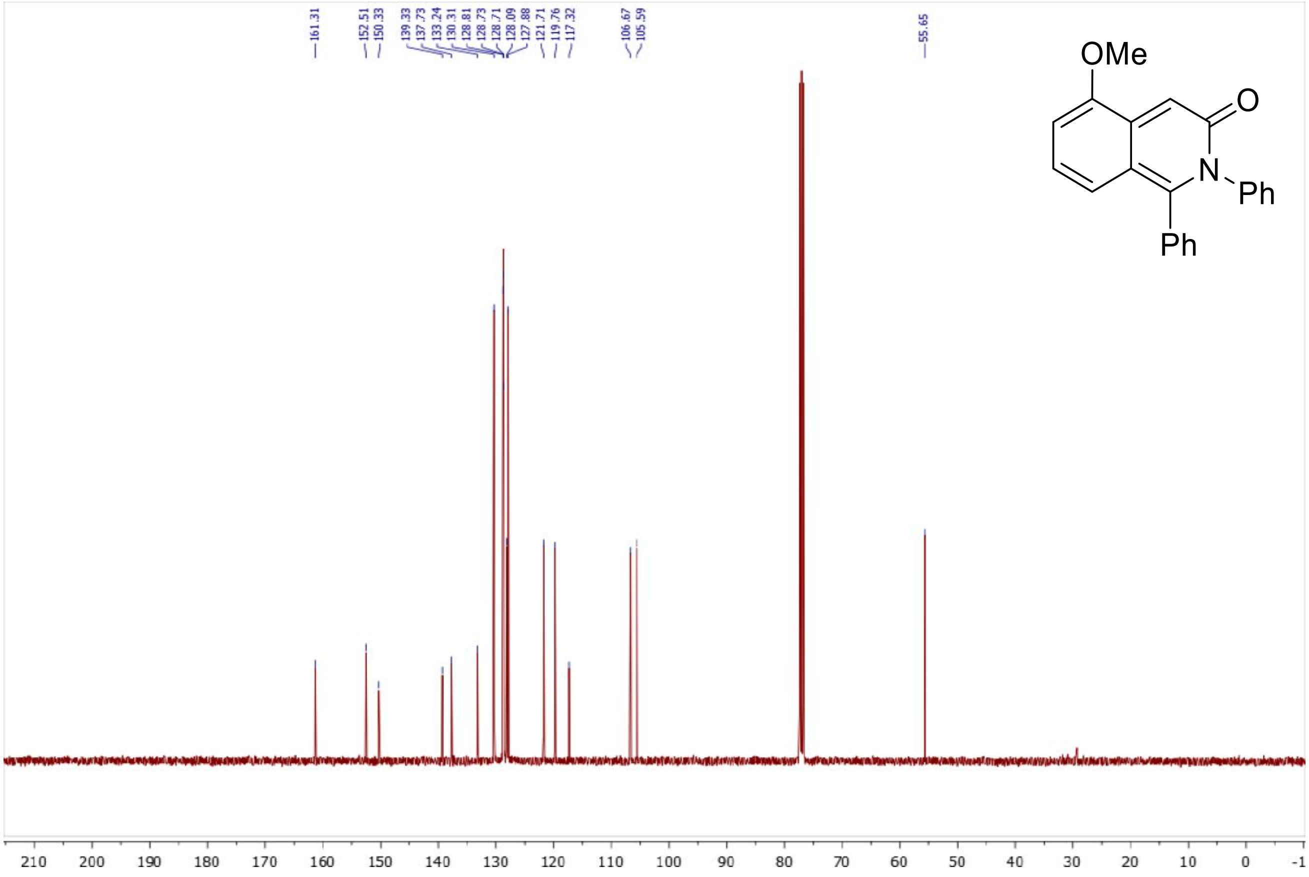

Copies of ^{1}H (400.13 MHz, $CDCl_3$) and $^{13}C\{^{1}H\}$ (100.61 MHz, $CDCl_3$) spectra of **9h**

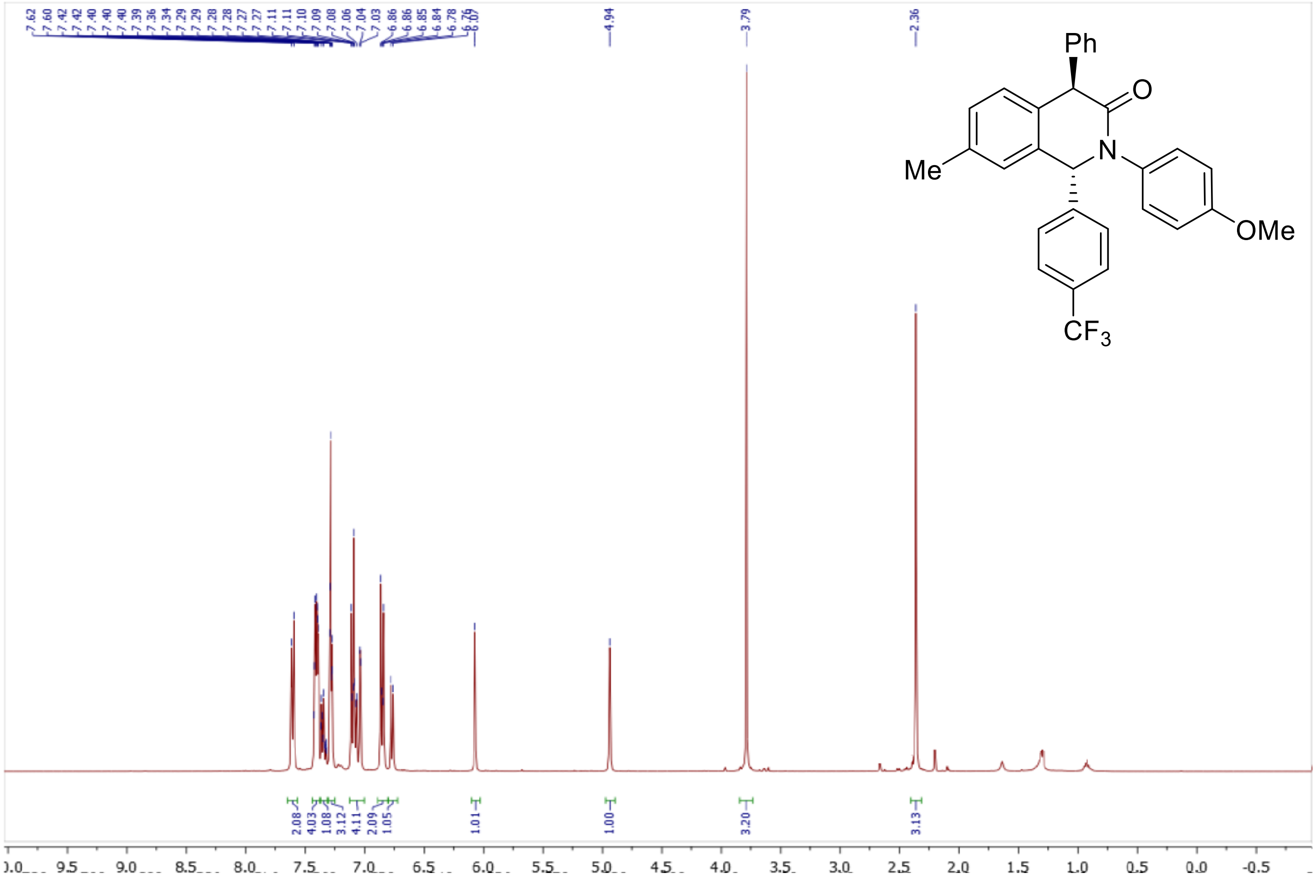

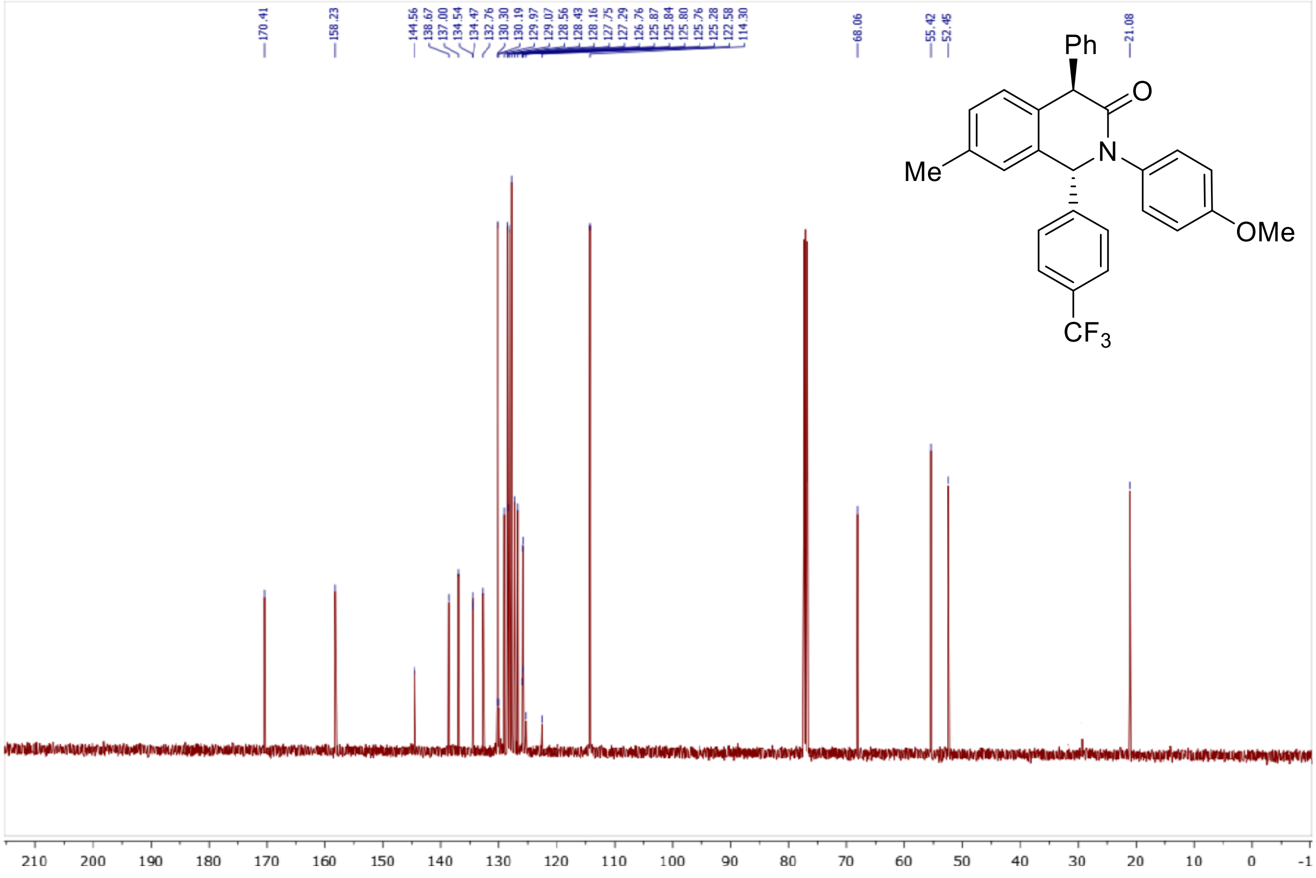

Copy of $^{19}F\{^{1}H\}$ (376.50 MHz, $CDCl_3$) spectrum of **9h**

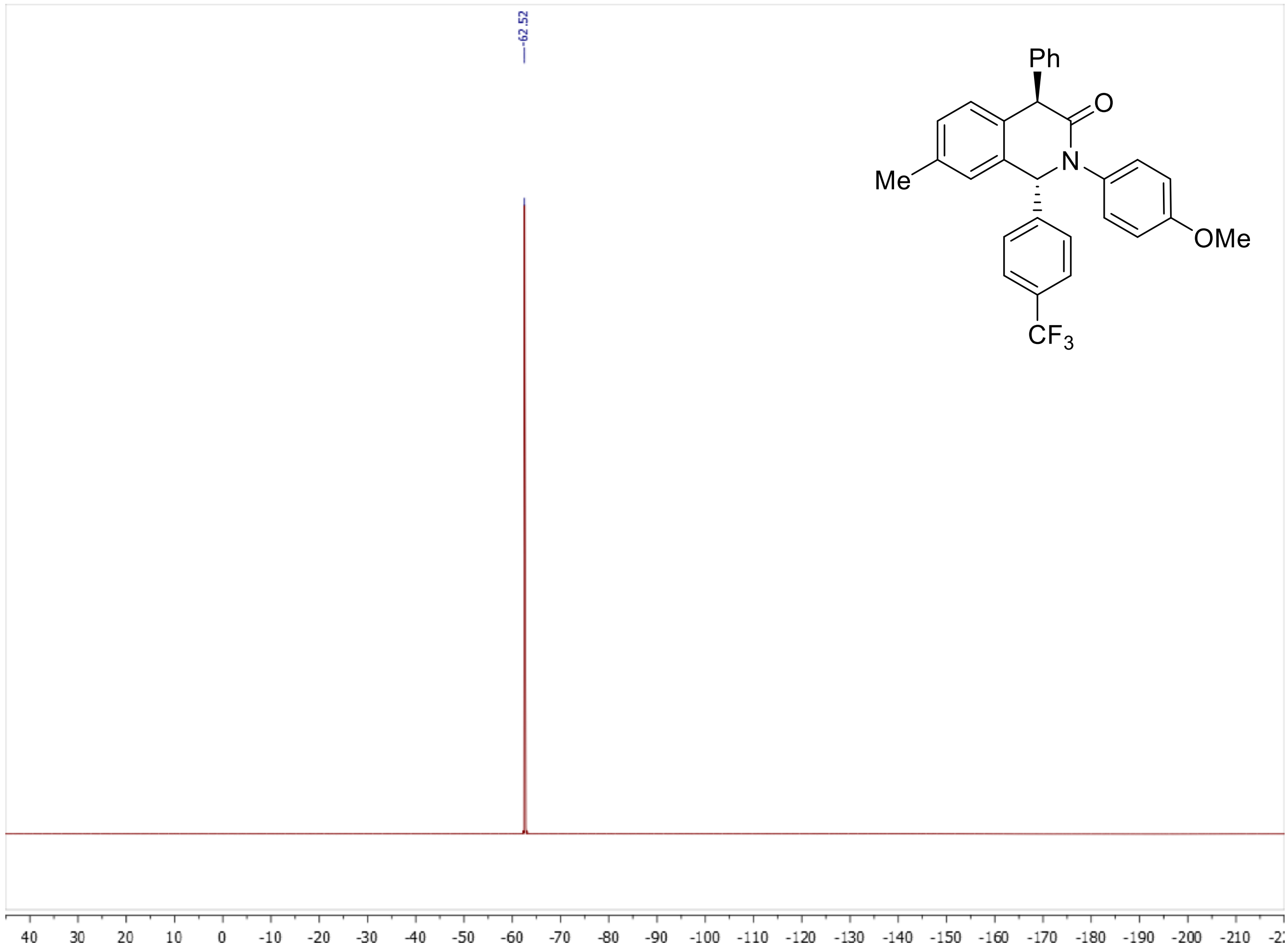

Copies of ^{1}H (400.13 MHz, $CDCl_3$) and $^{13}C\{^{1}H\}$ (100.61 MHz, $CDCl_3$) spectra of **15h**

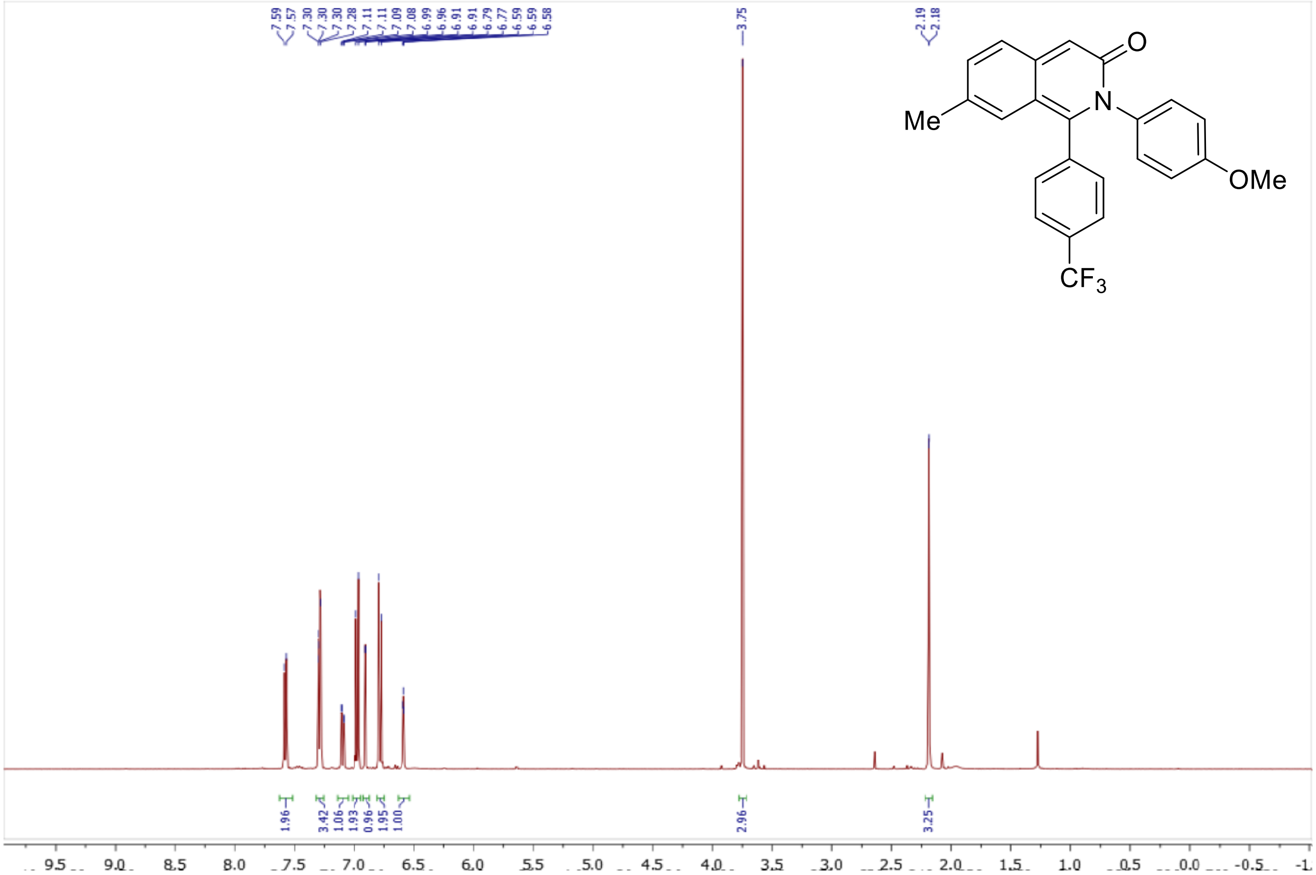

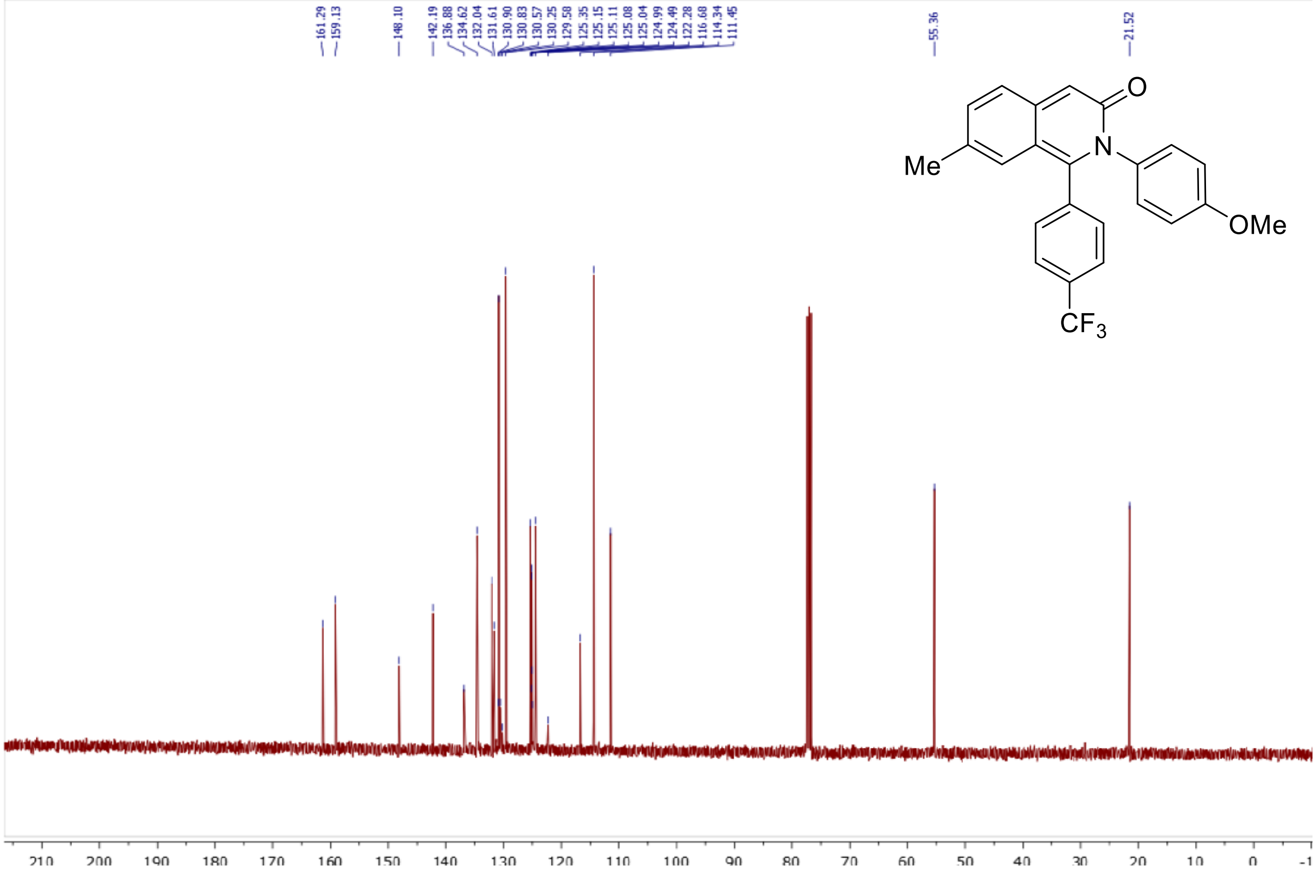

Copy of $^{19}F\{^{1}H\}$ (376.50 MHz, $CDCl_3$) spectrum of **15h**

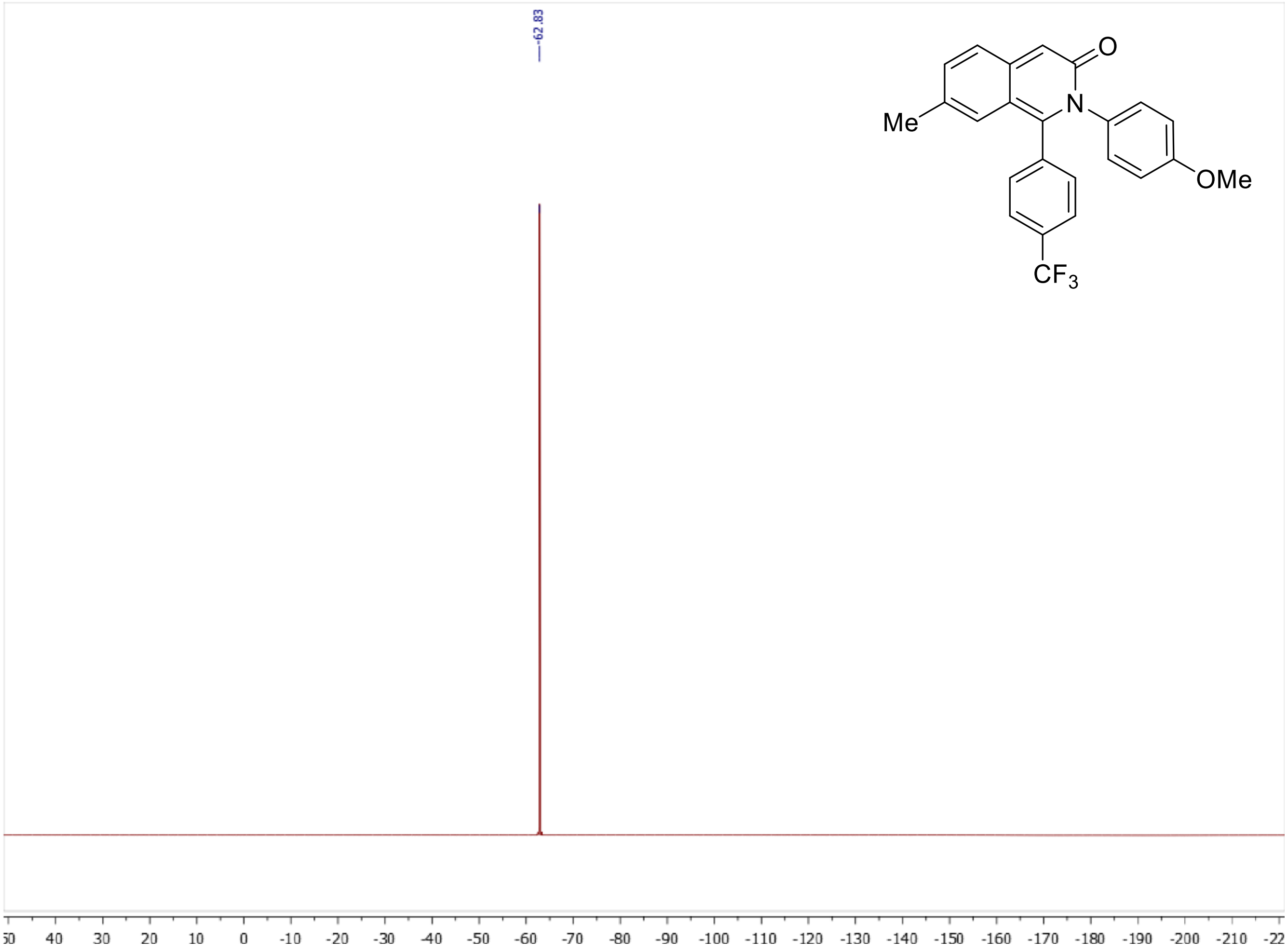

Copies of ^{1}H (400.13 MHz, $CDCl_3$) and $^{13}C\{^{1}H\}$ (100.61 MHz, $CDCl_3$) spectra of **9i**

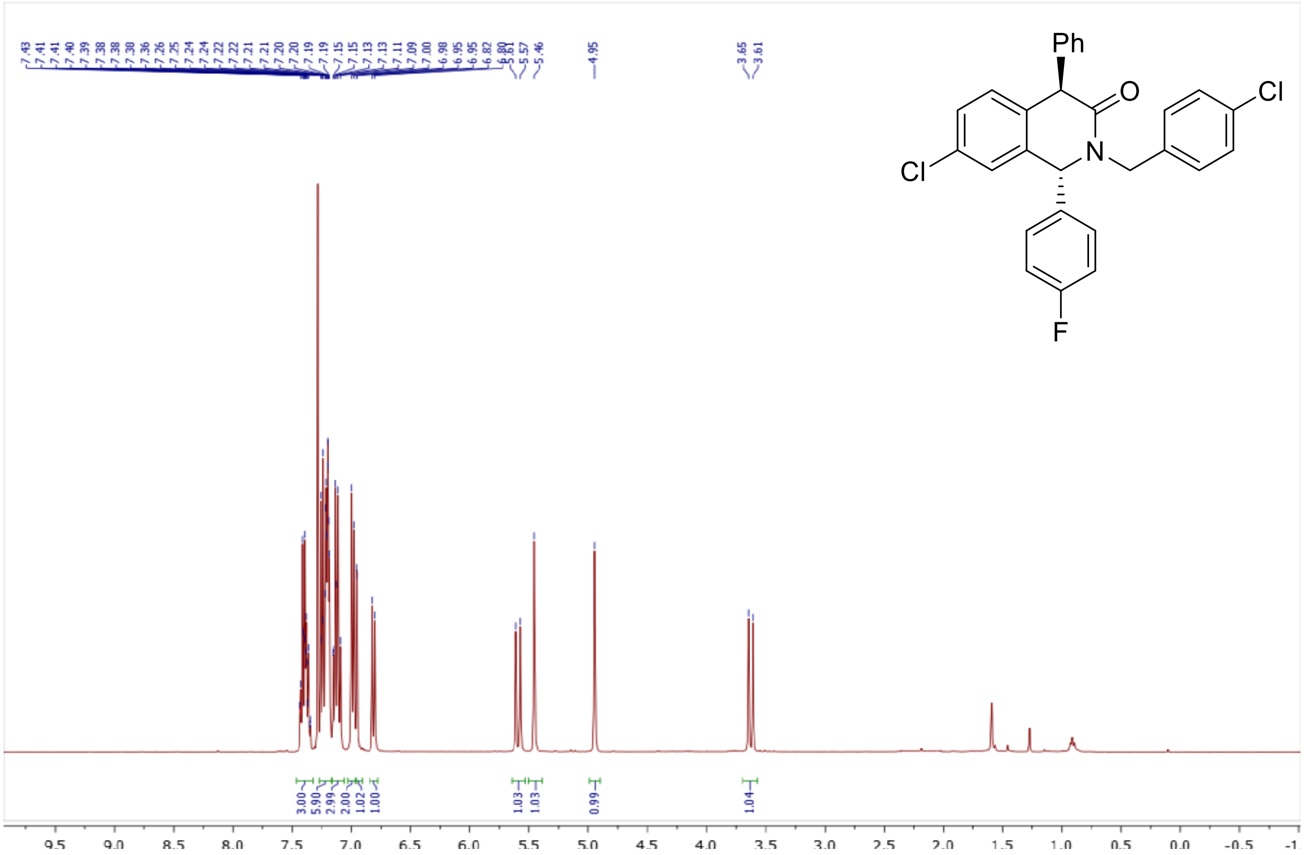

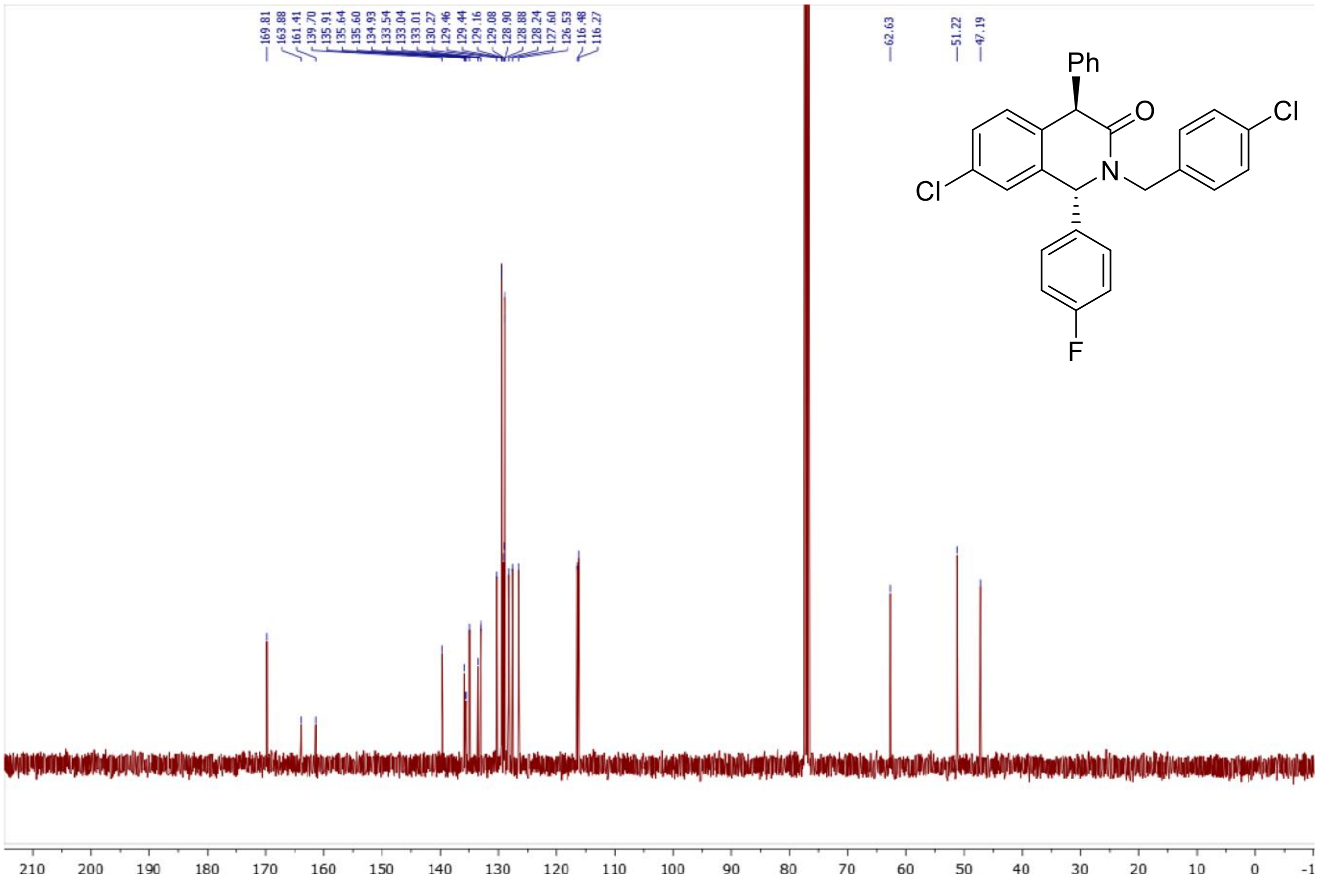

Copy of $^{19}F\{^{1}H\}$ (376.50 MHz, $CDCl_3$) spectrum of **9i**

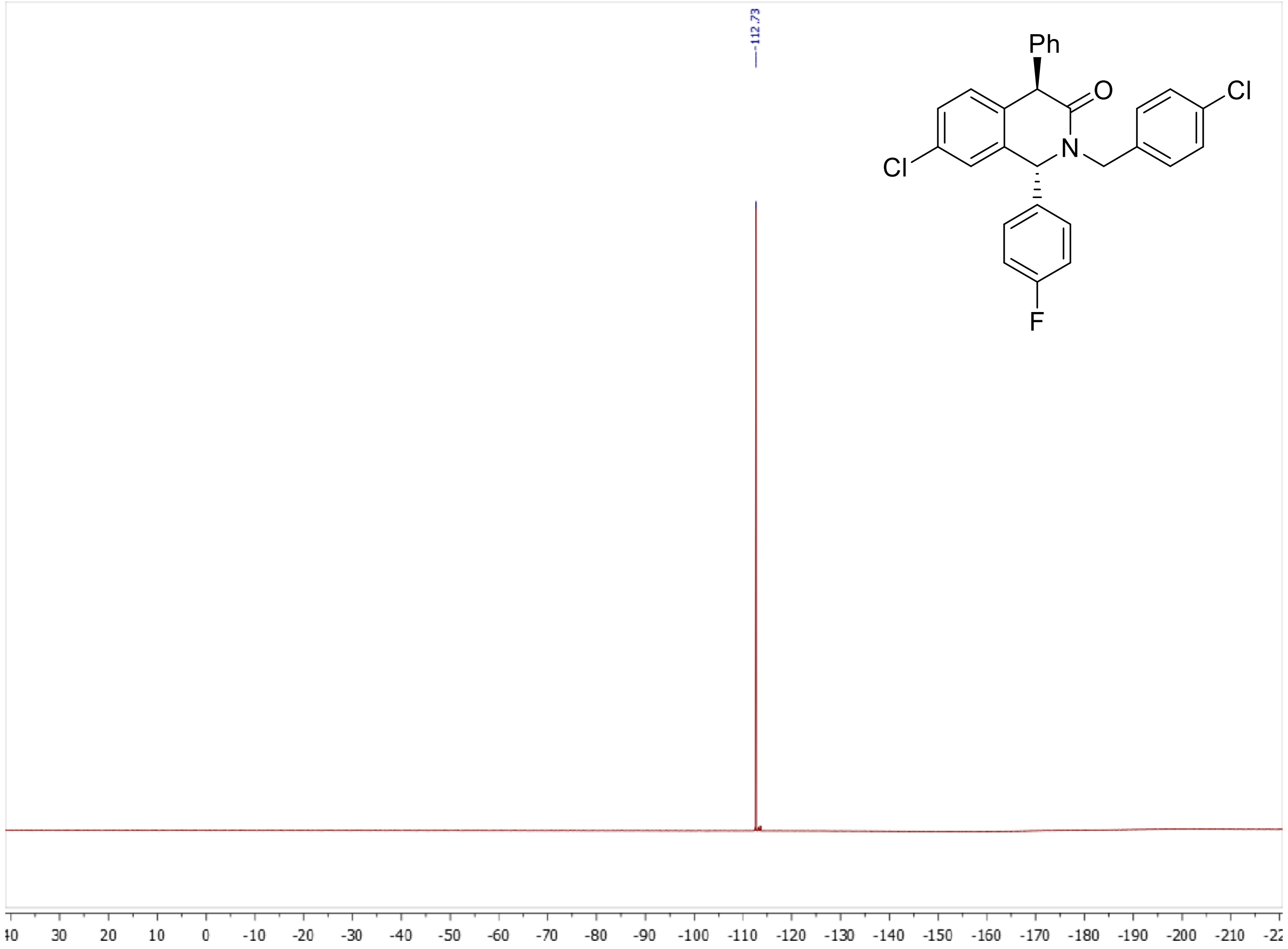

Copies of ^{1}H (400.13 MHz, $CDCl_3$) and $^{13}C\{^{1}H\}$ (100.61 MHz, $CDCl_3$) spectra of **15i**

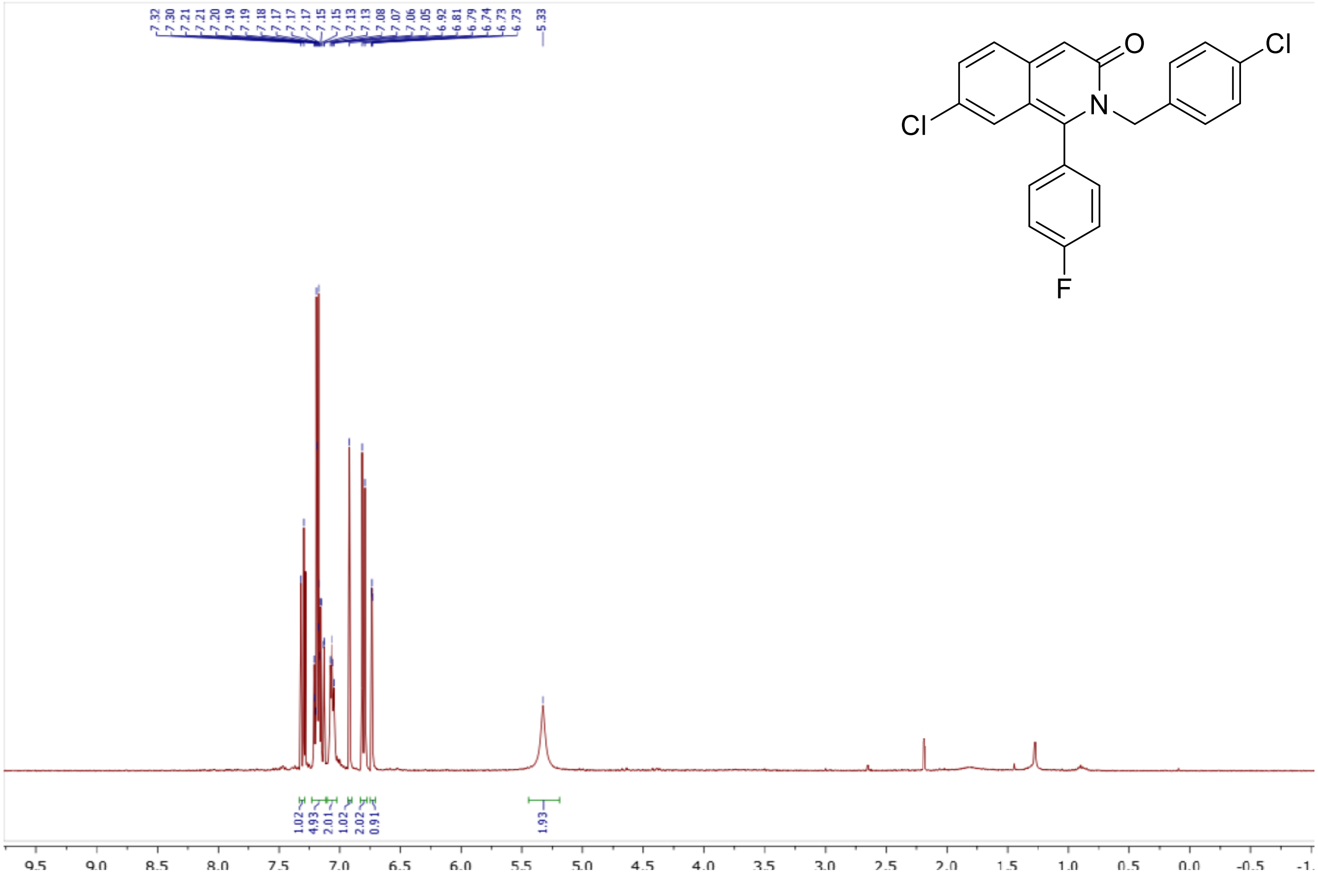

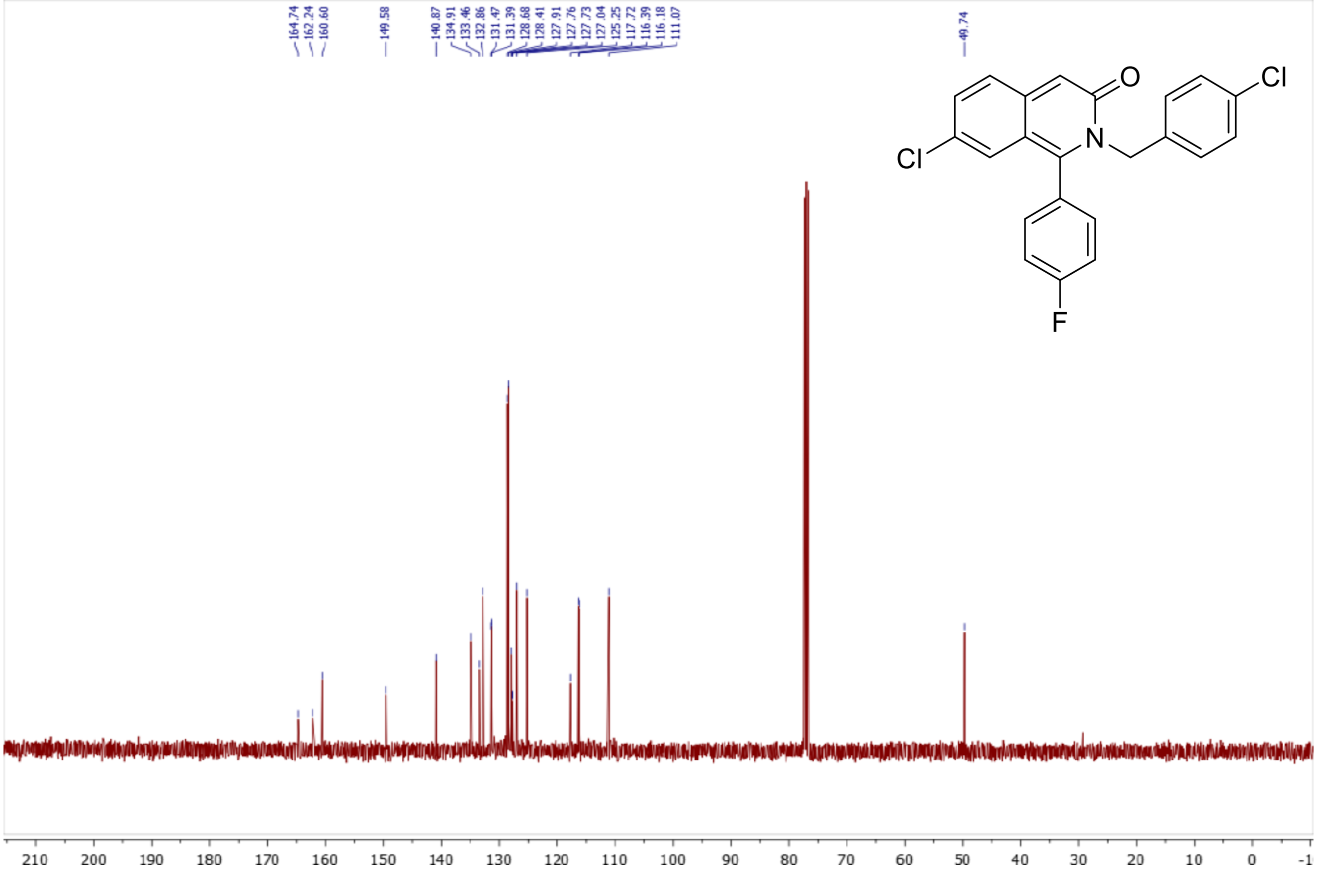

Copy of $^{19}F\{^{1}H\}$ (376.50 MHz, $CDCl_3$) spectrum of **15i**

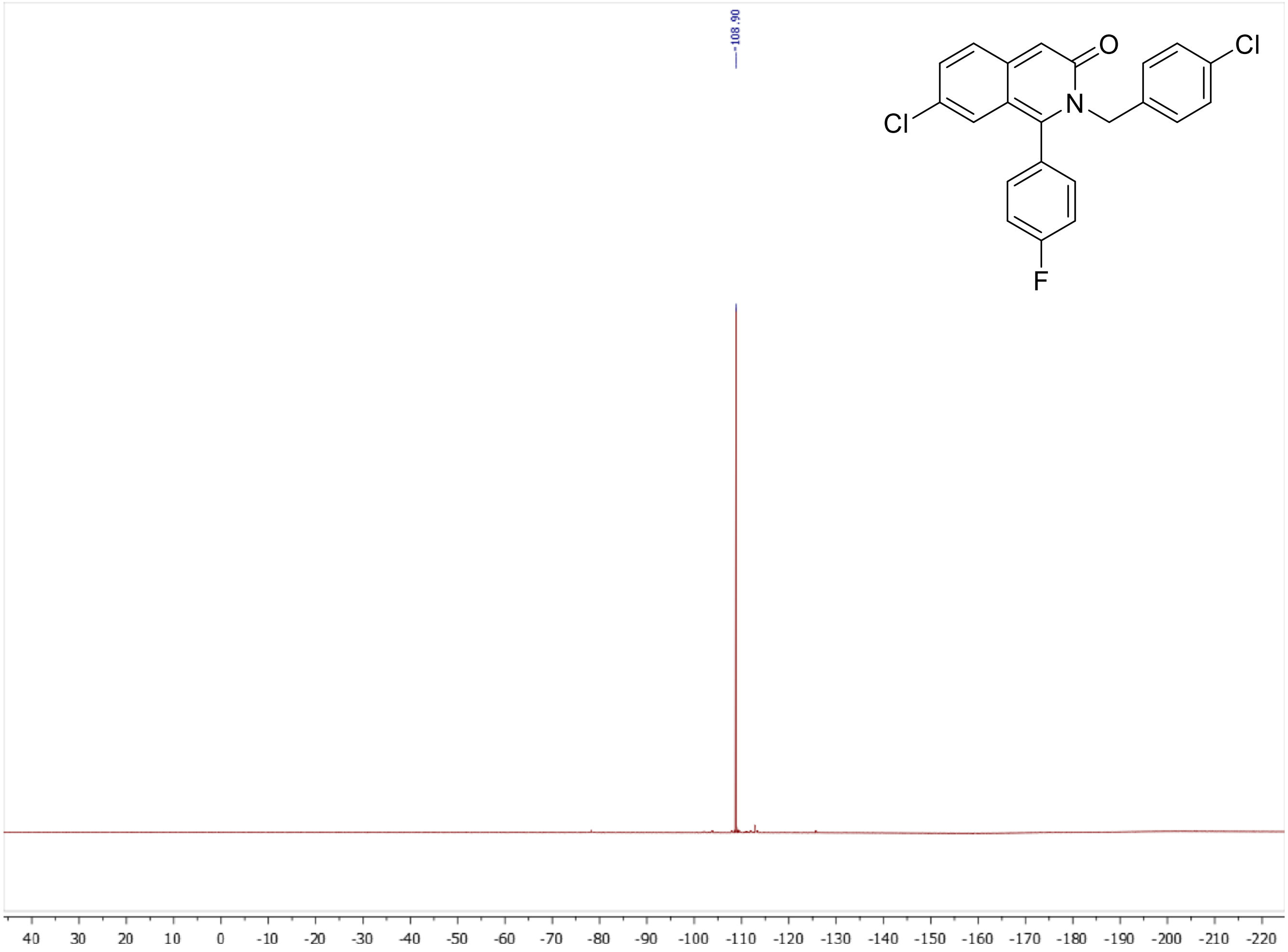

Copies of ^{1}H (400.13 MHz, $CDCl_3$) and $^{13}C\{^{1}H\}$ (100.61 MHz, $CDCl_3$) spectra of **9j**

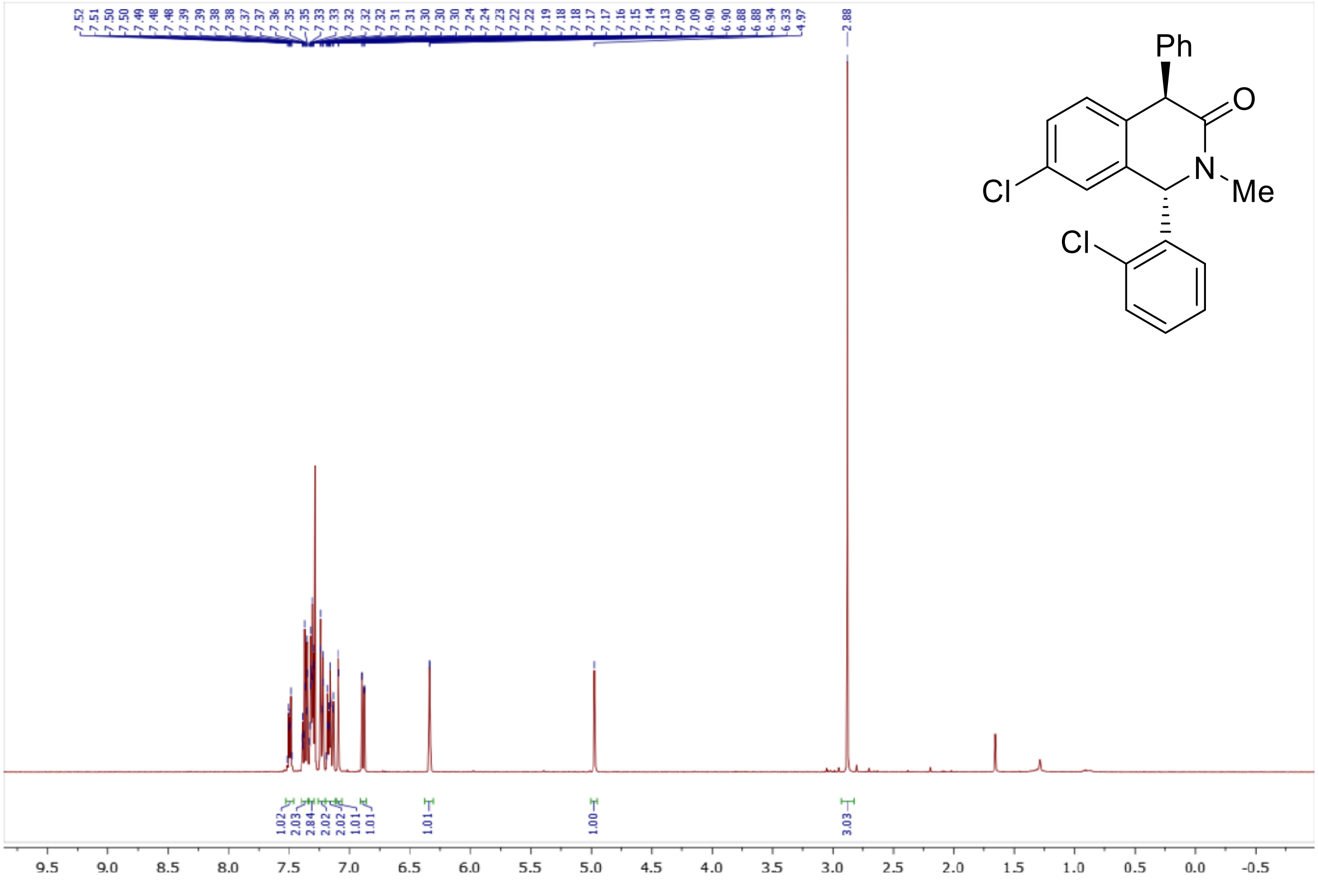

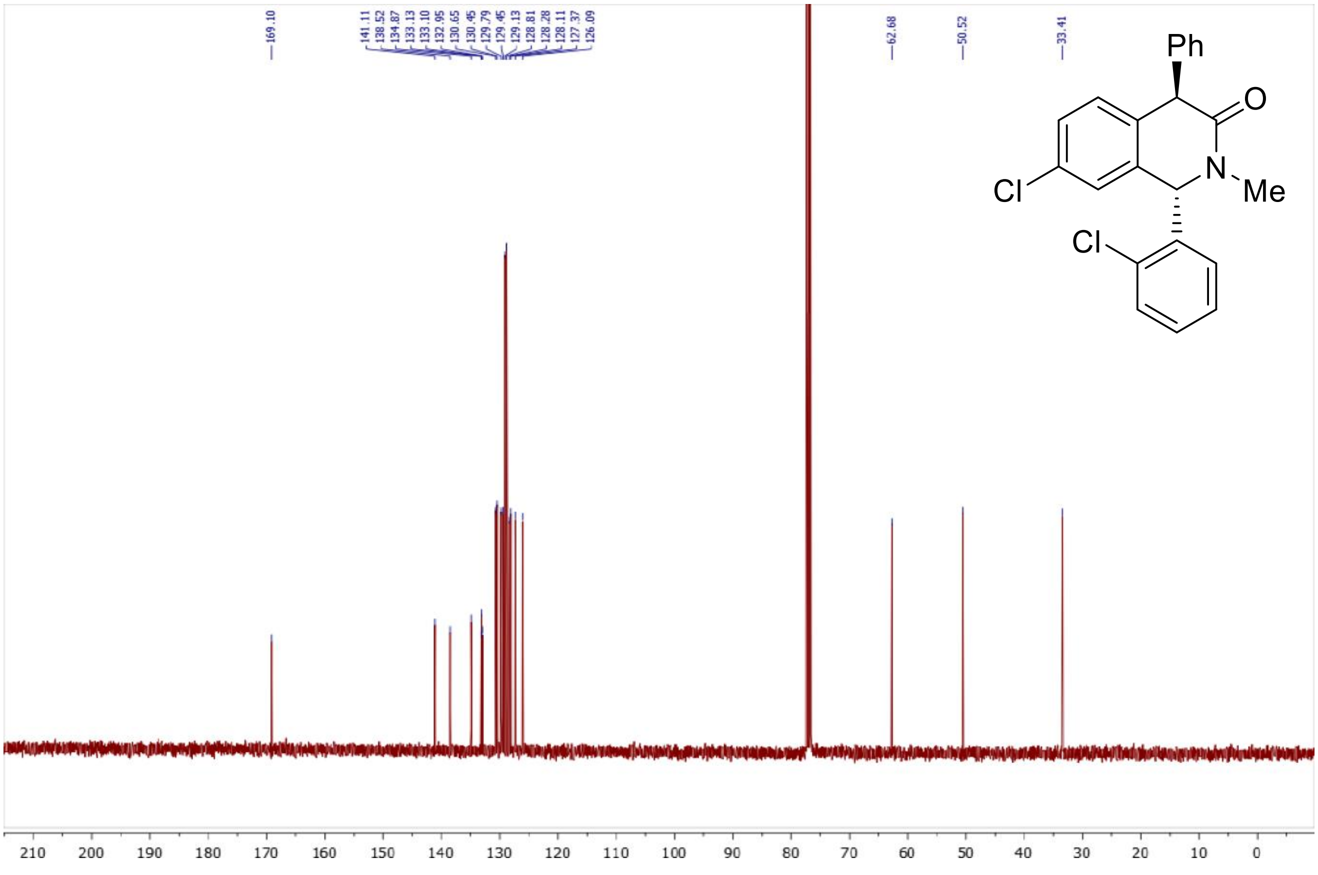

Copies of ^{1}H (400.13 MHz, $CDCl_3$) and $^{13}C\{^1H\}$ (100.61 MHz, $CDCl_3$) spectra of **15j**

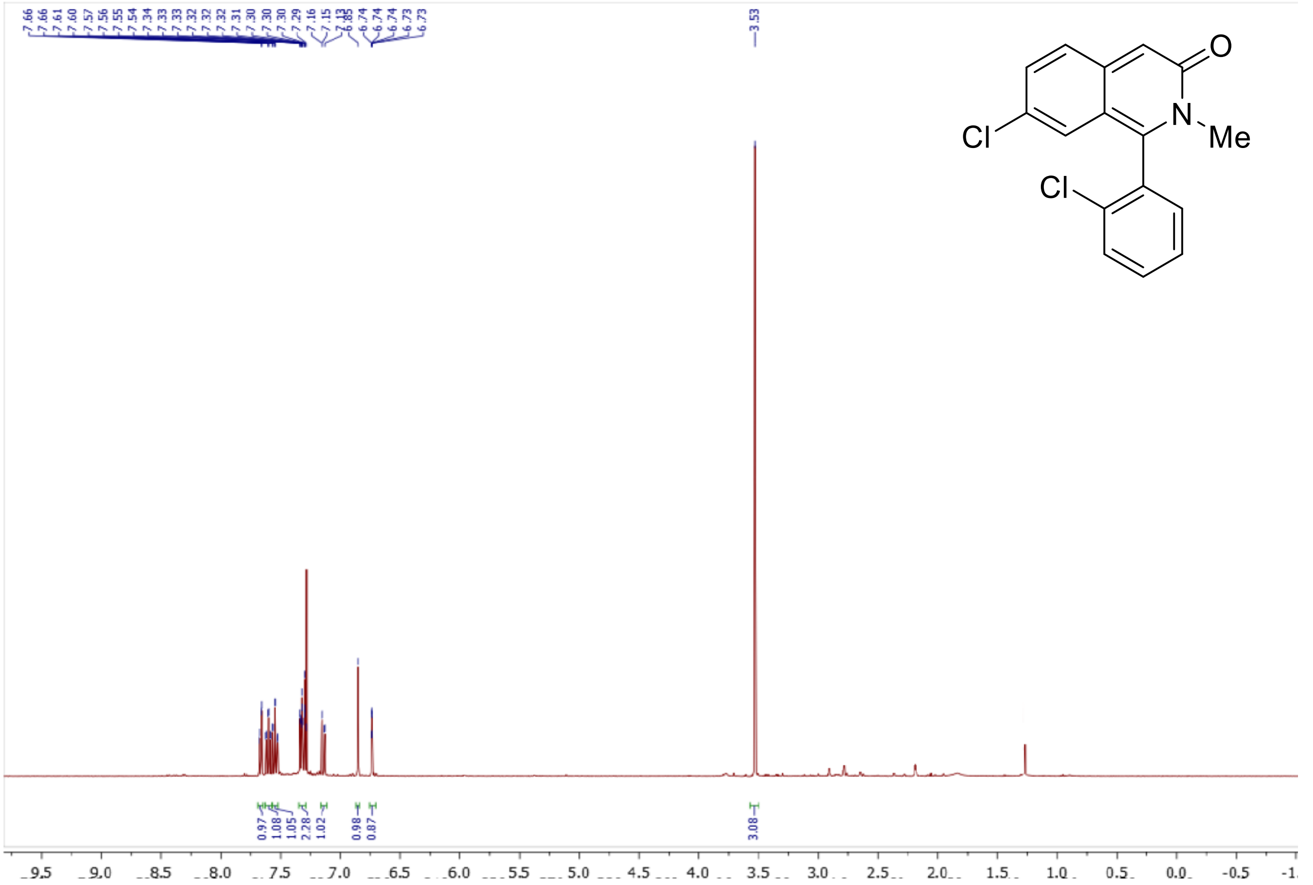

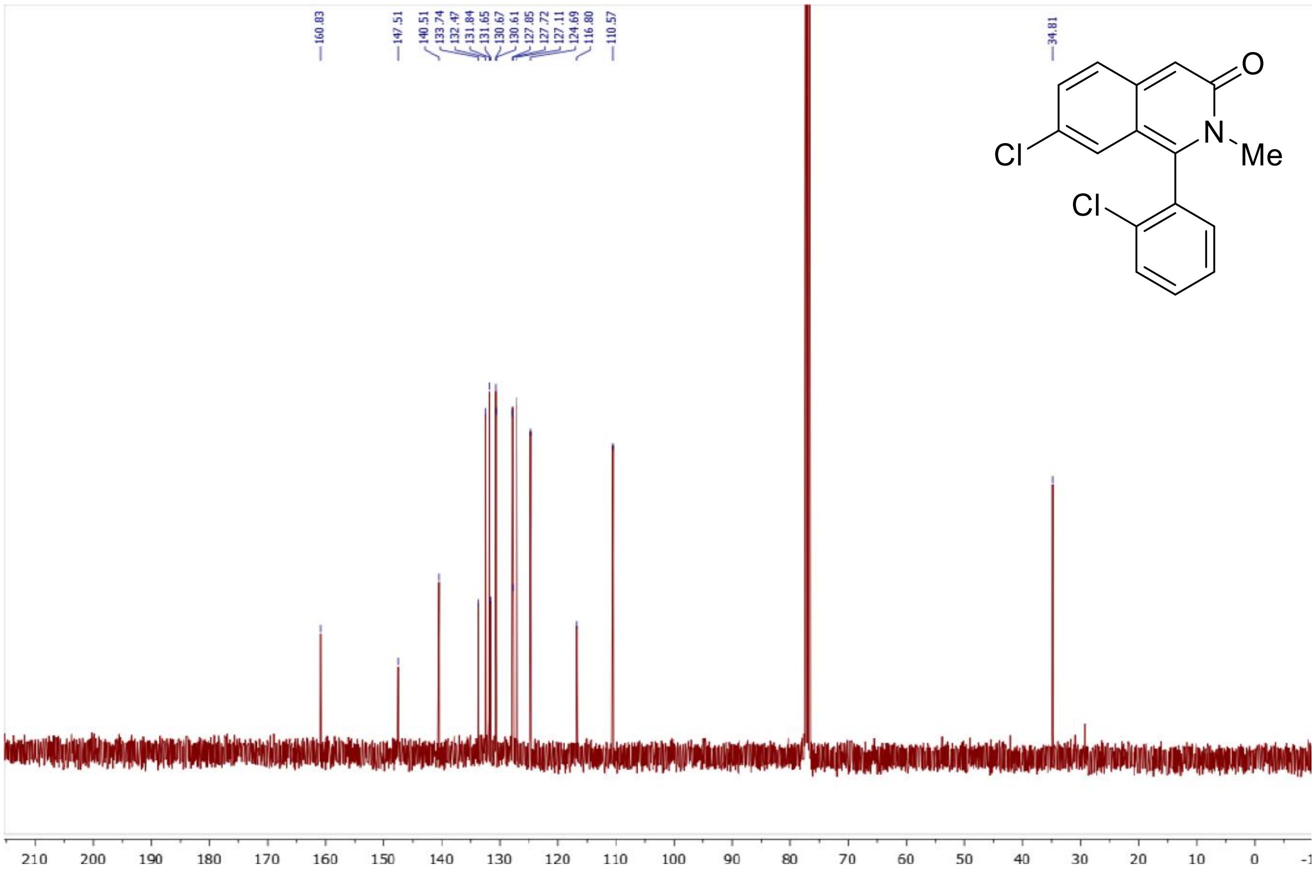

Copies of ^{1}H (400.13 MHz, $CDCl_3$) and $^{13}C\{^{1}H\}$ (100.61 MHz, $CDCl_3$) spectra of **9k**

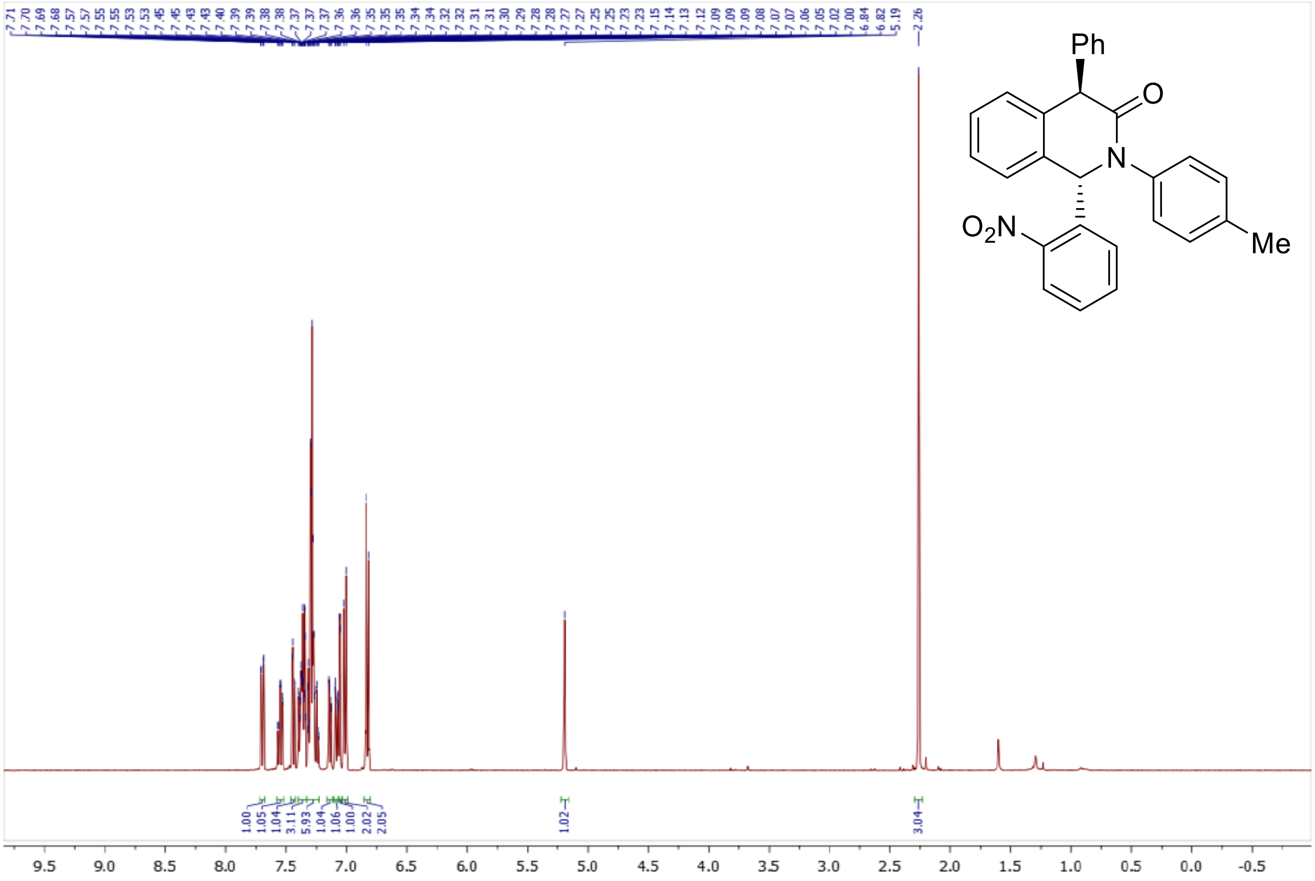

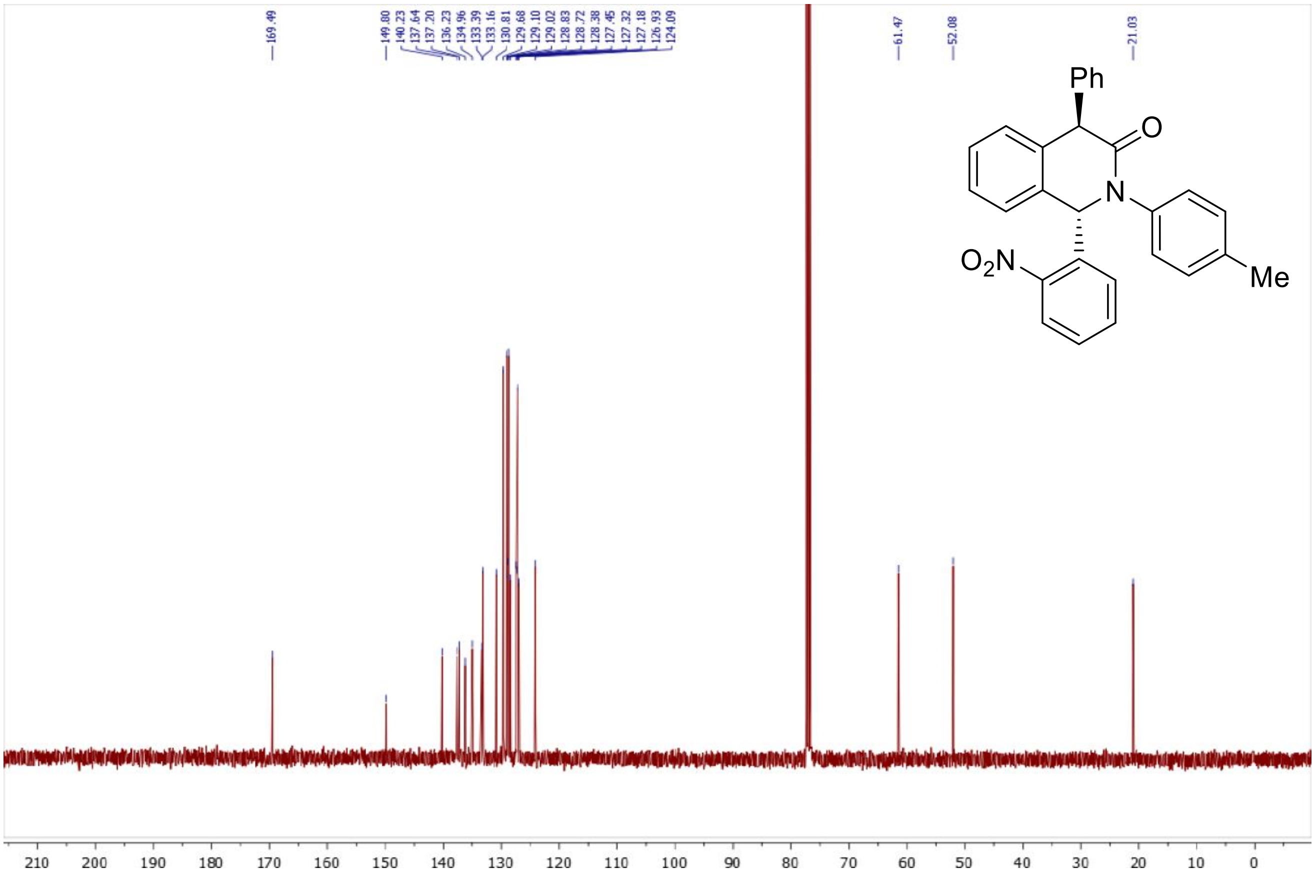

Copies of ^{1}H (400.13 MHz, $CDCl_3$) and $^{13}C\{^{1}H\}$ (100.61 MHz, $CDCl_3$) spectra of **15k**

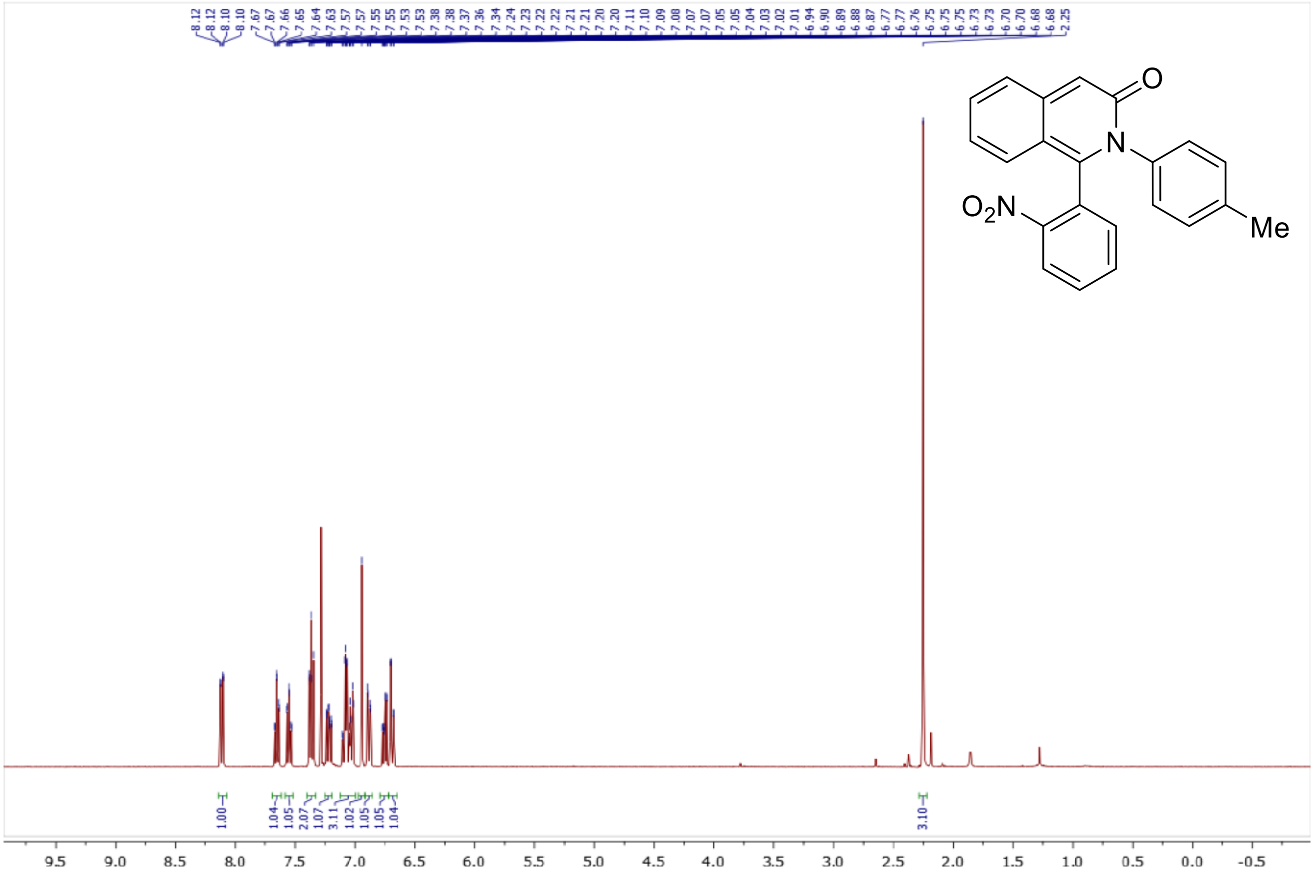

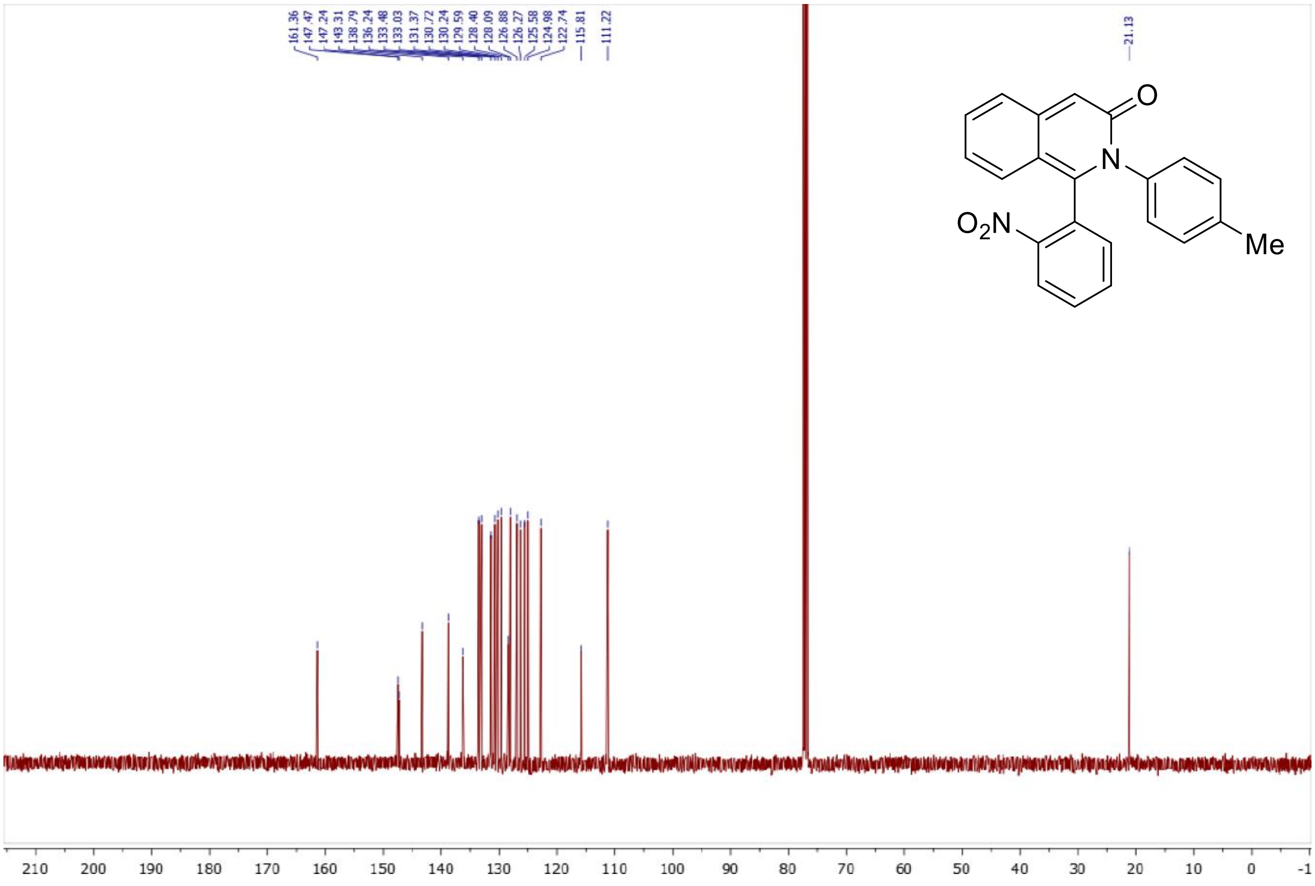

Copies of ^{1}H (400.13 MHz, $CDCl_3$) and $^{13}C\{^{1}H\}$ (100.61 MHz, $CDCl_3$) spectra of **9l**

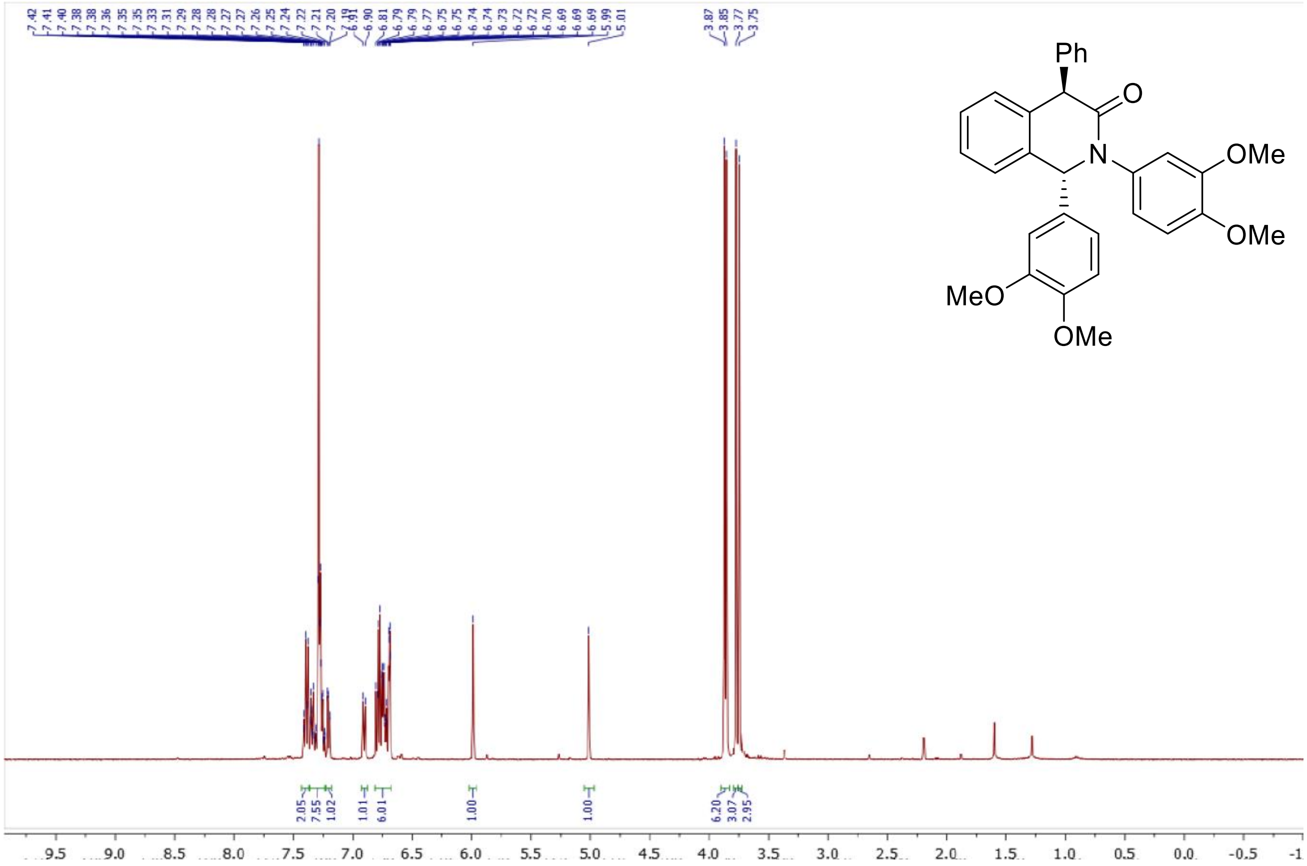

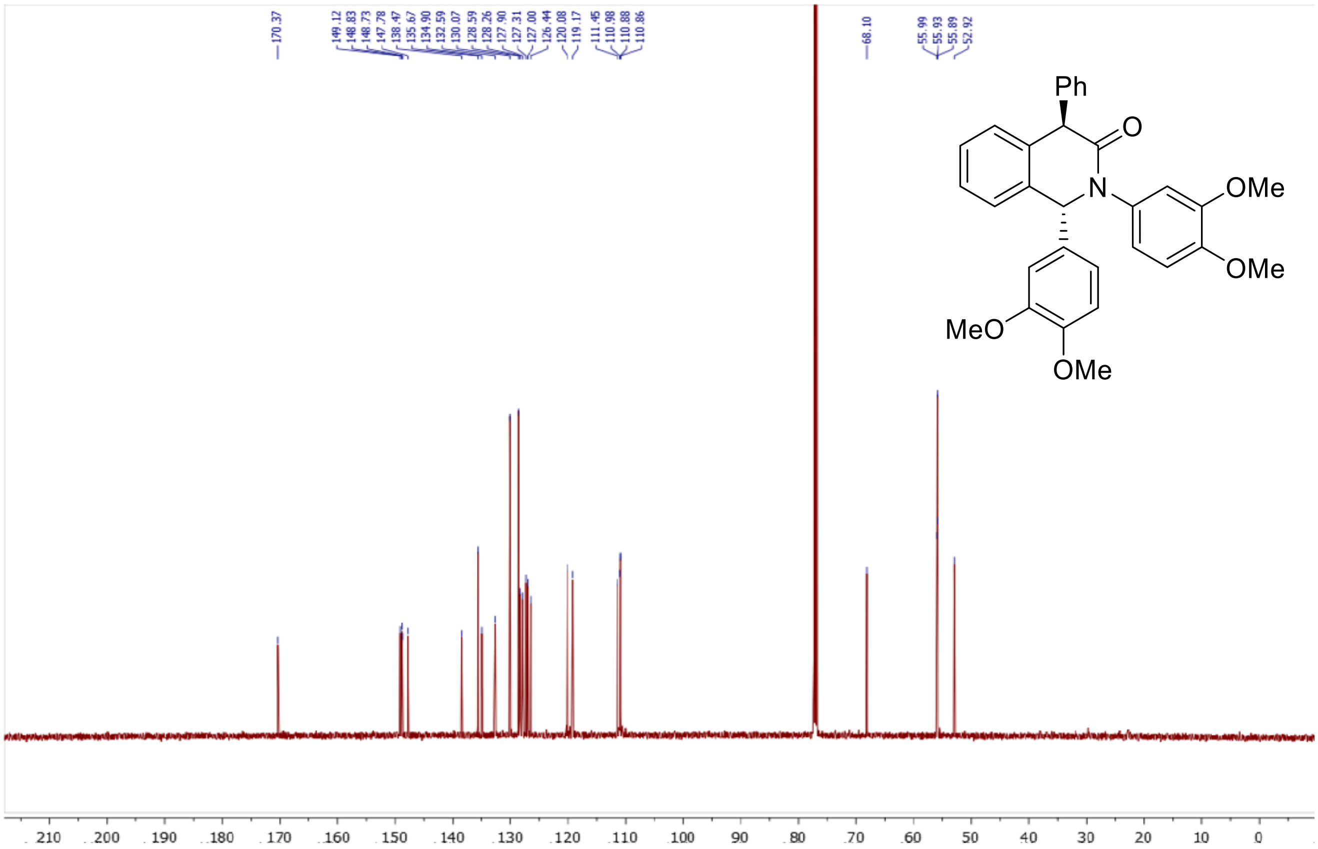

Copies of ^{1}H (400.13 MHz, $CDCl_3$) and $^{13}C\{^{1}H\}$ (100.61 MHz, $CDCl_3$) spectra of **15l**

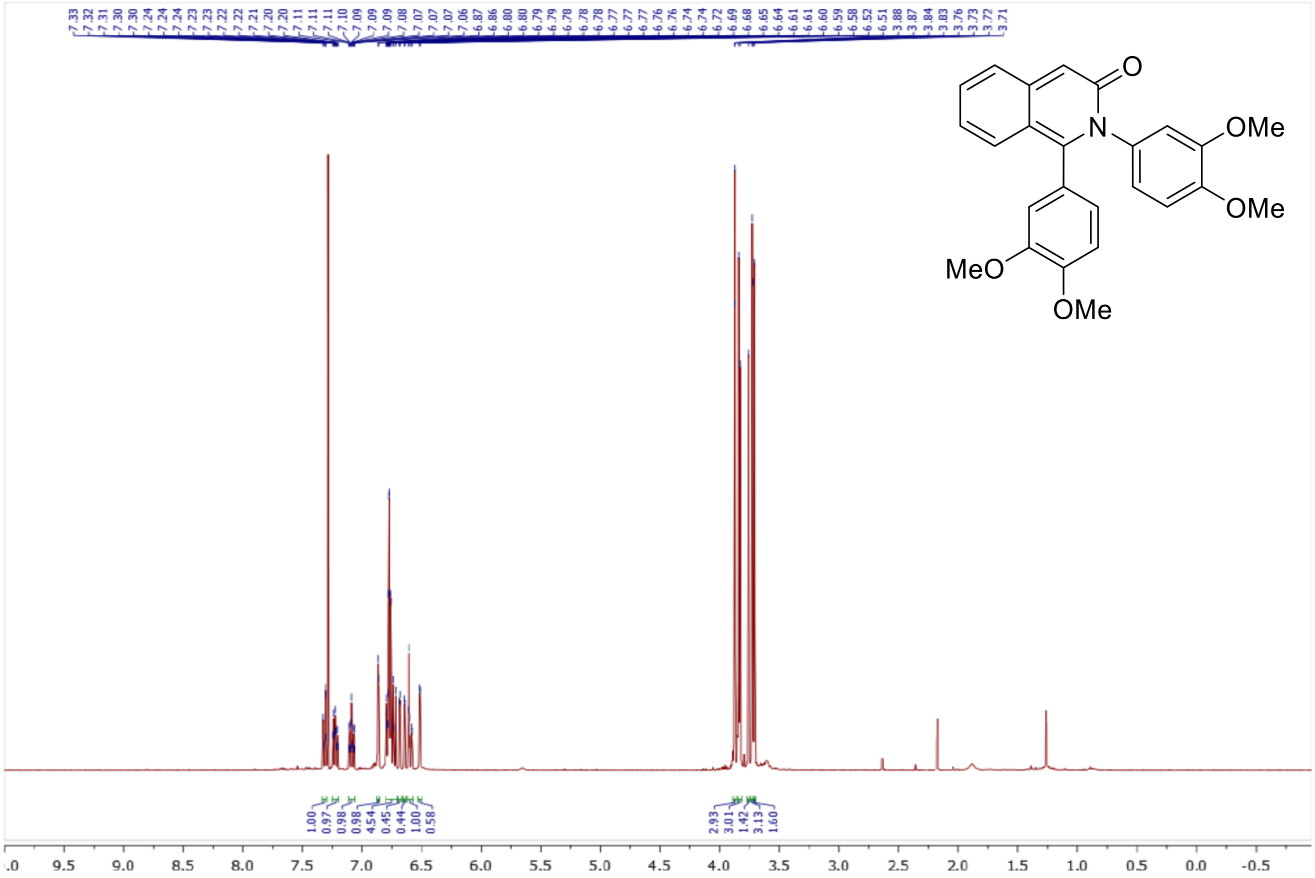

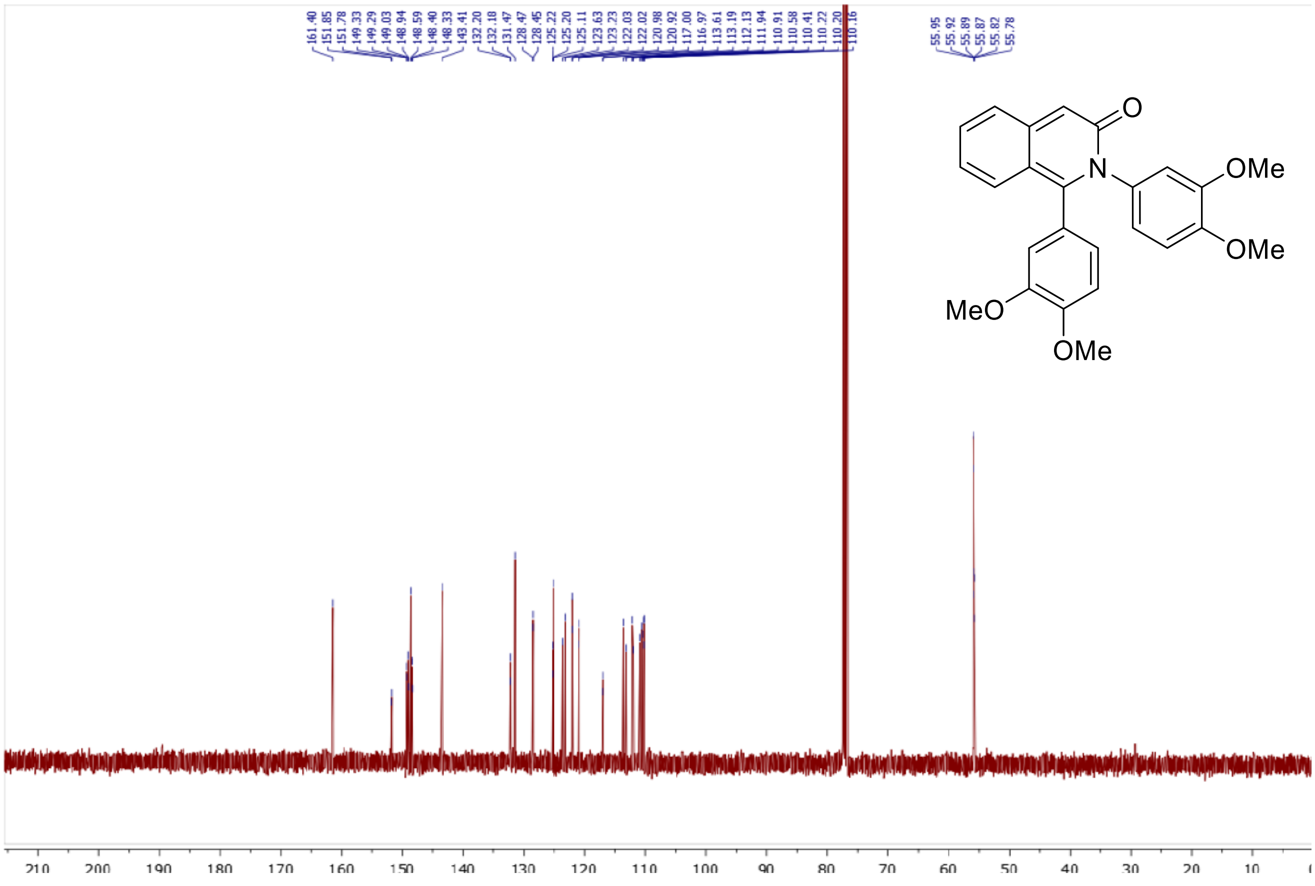

Copies of ^{1}H (400.13 MHz, $CDCl_3$) and $^{13}C\{^{1}H\}$ (100.61 MHz, $CDCl_3$) spectra of **9m**

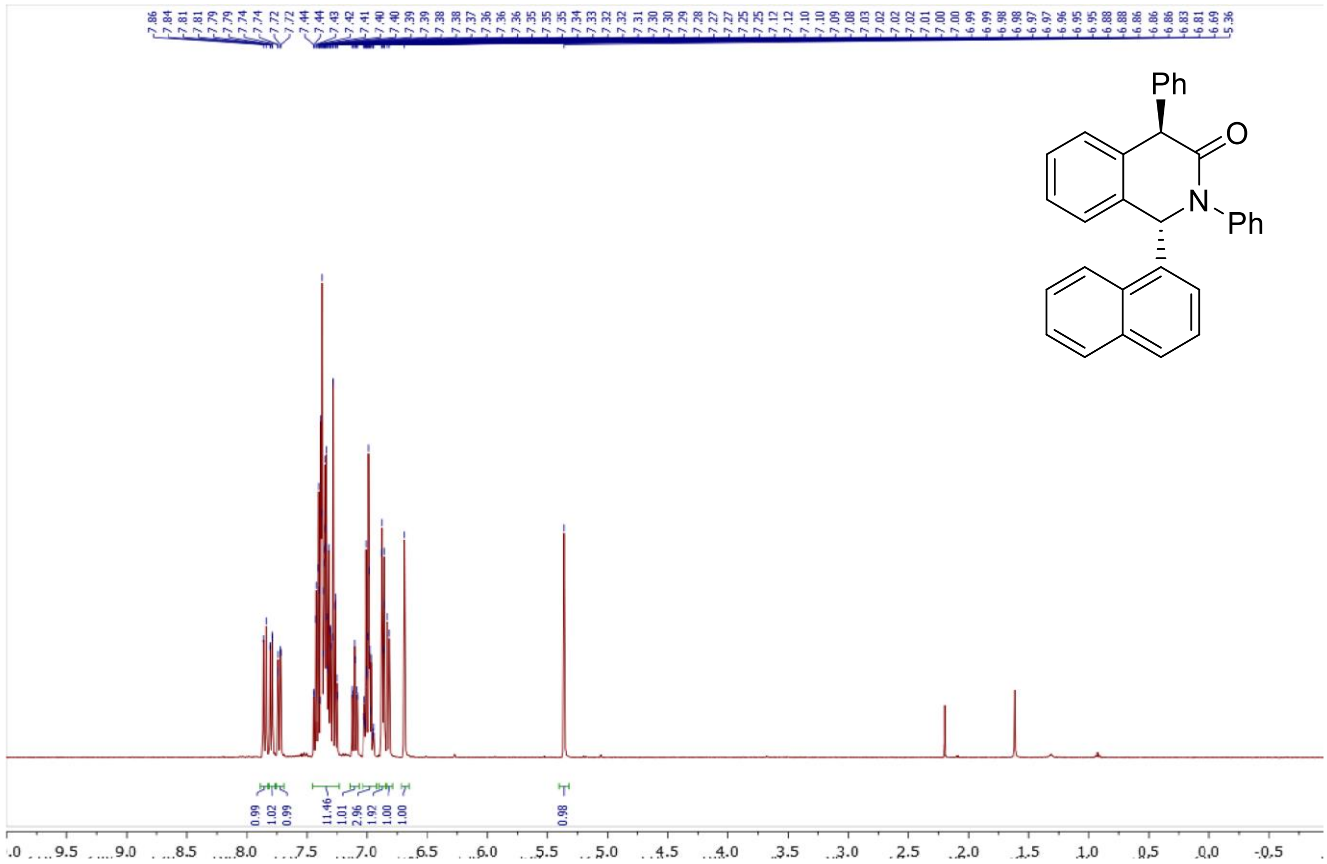

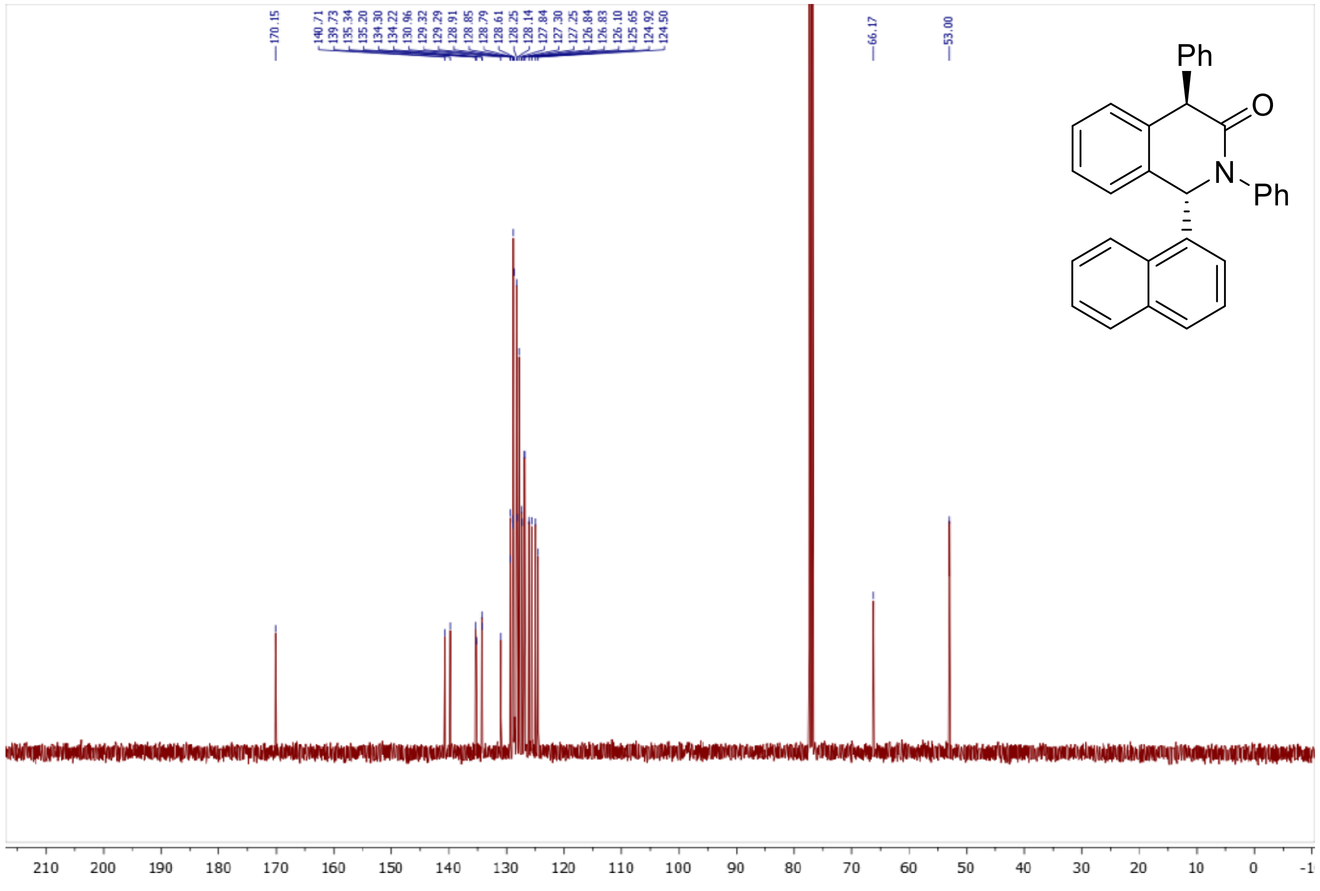

Copies of ^{1}H (400.13 MHz, $CDCl_3$) and $^{13}C\{^{1}H\}$ (100.61 MHz, $CDCl_3$) spectra of **15m**

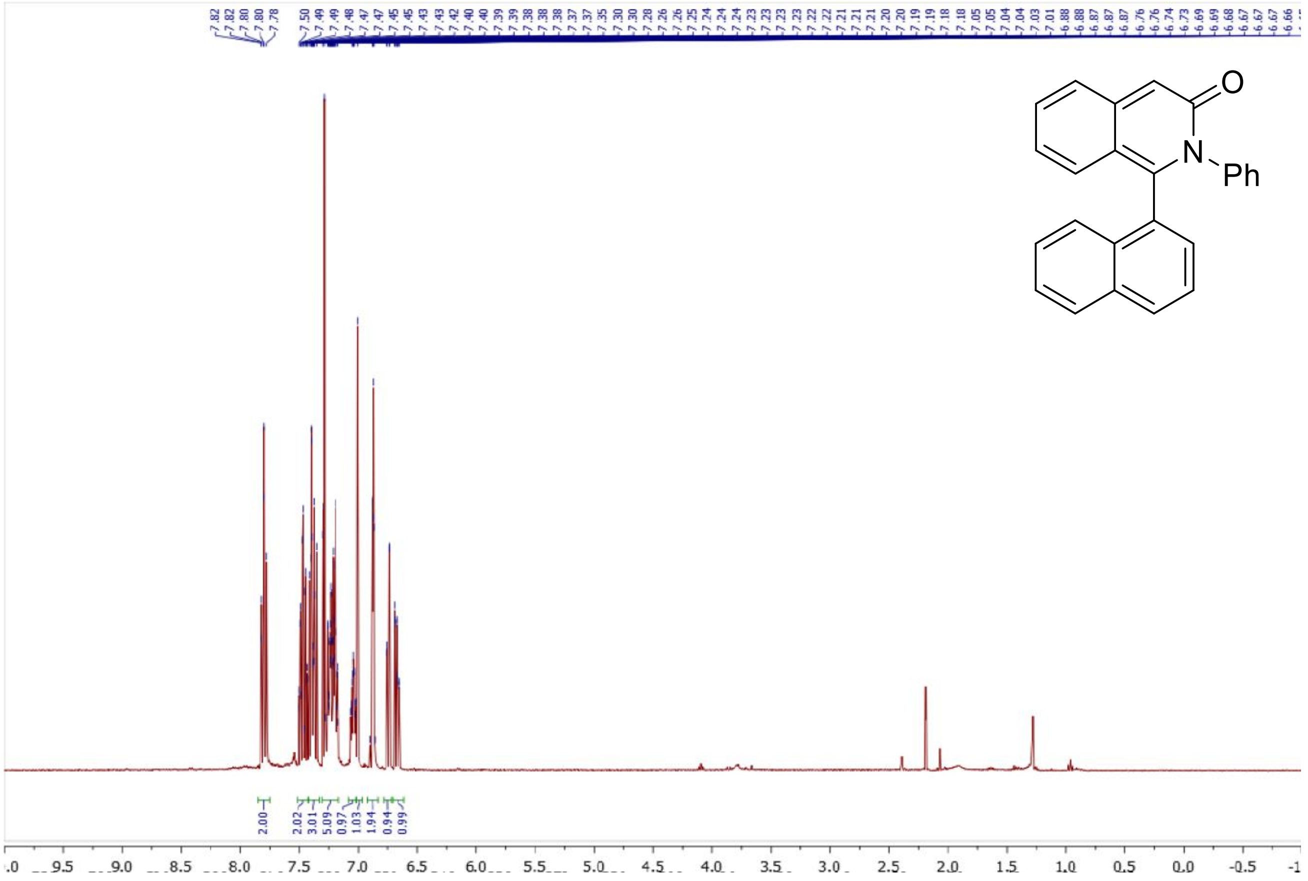

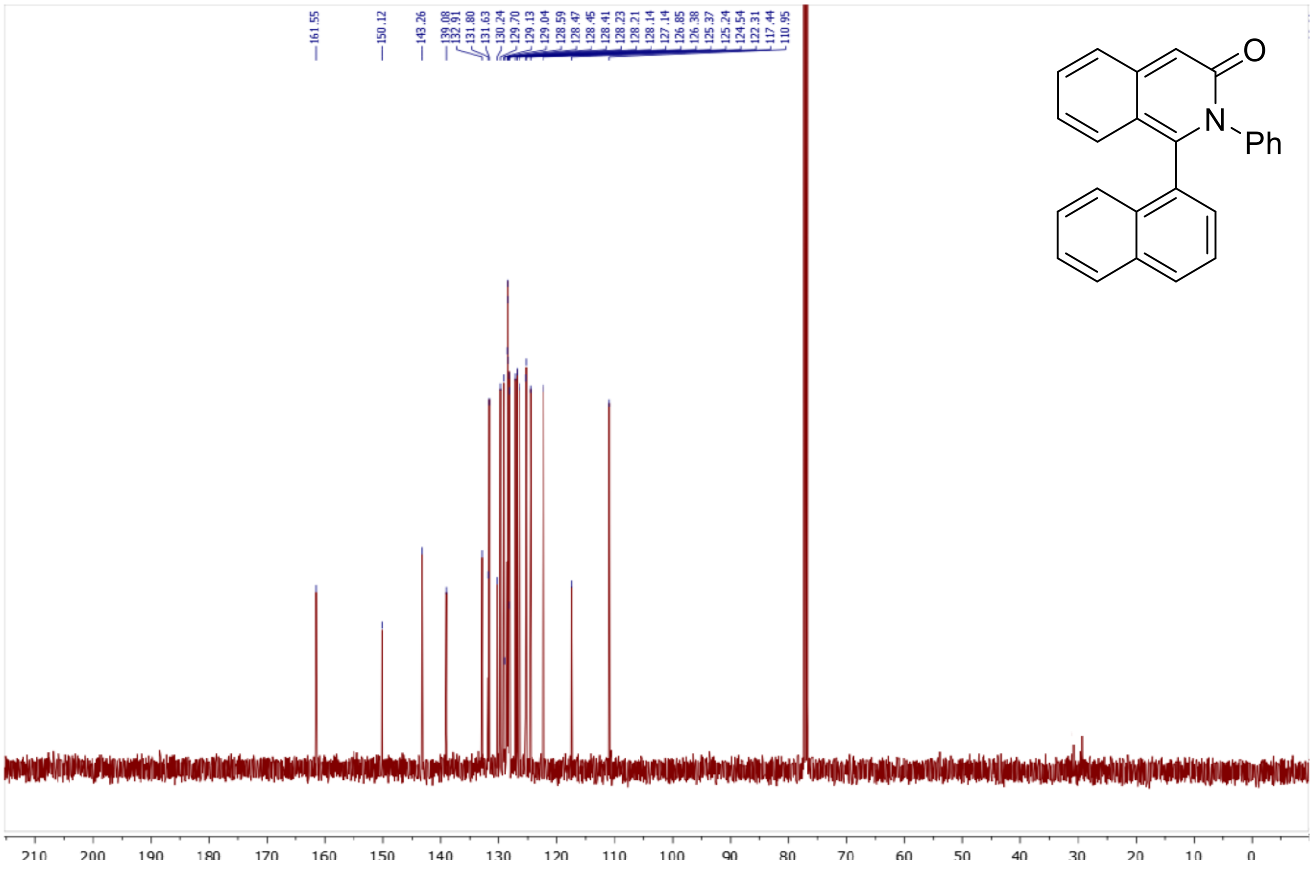

Copies of ^{1}H (400.13 MHz, CDCl$_3$) and ^{13}C{^{1}H} (100.61 MHz, CDCl$_3$) spectra of **9n**

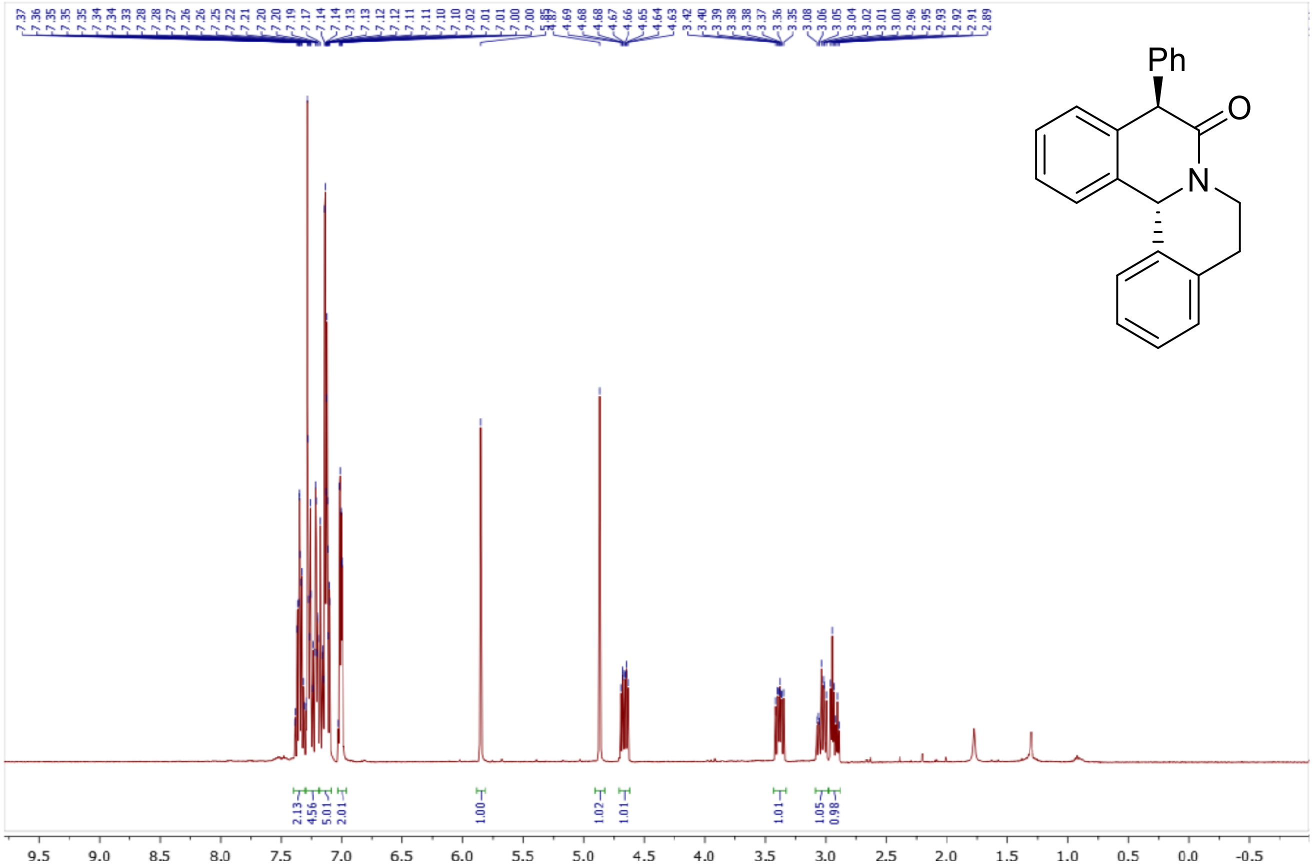

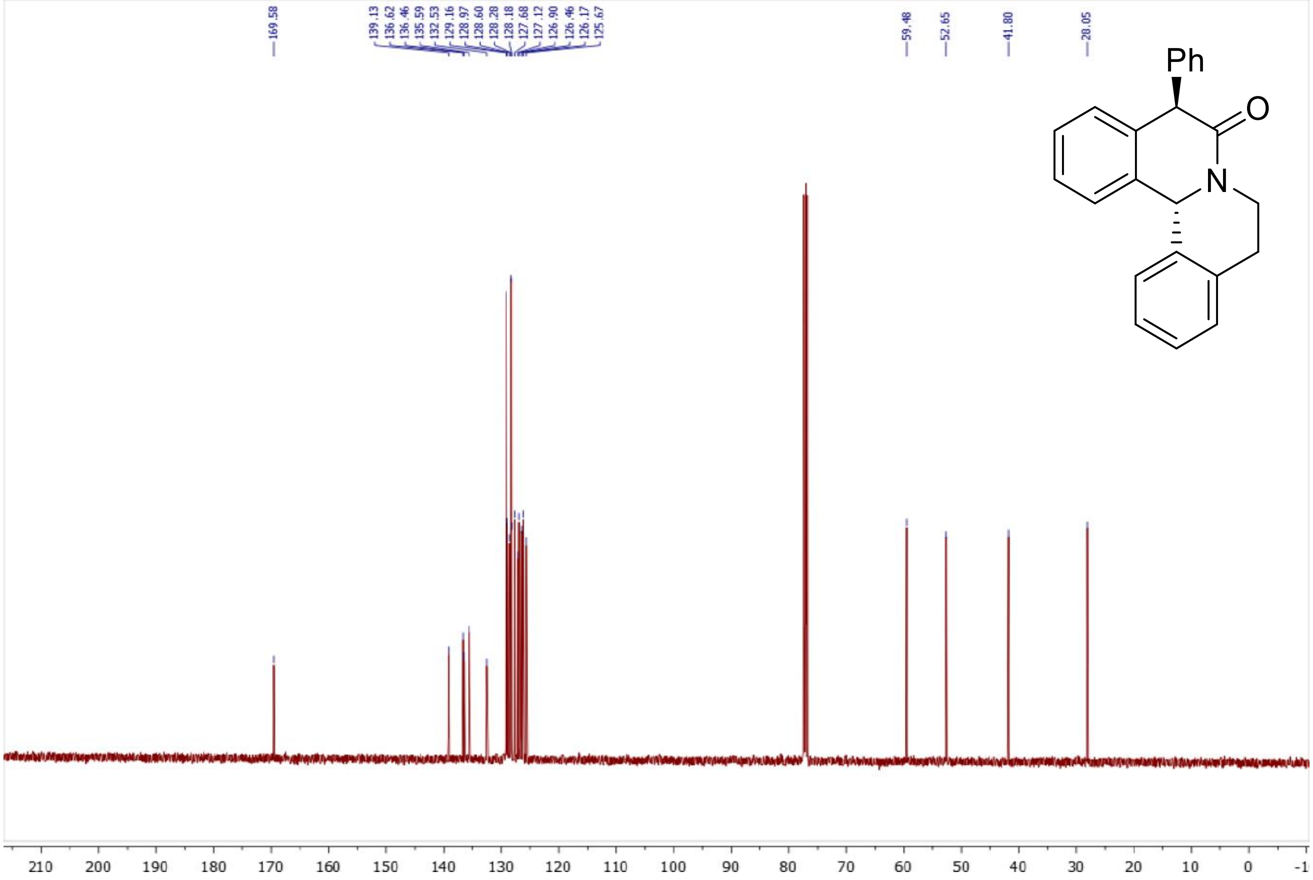

Copies of ^{1}H (400.13 MHz, $CDCl_3$) and $^{13}C\{^{1}H\}$ (100.61 MHz, $CDCl_3$) spectra of **15n**

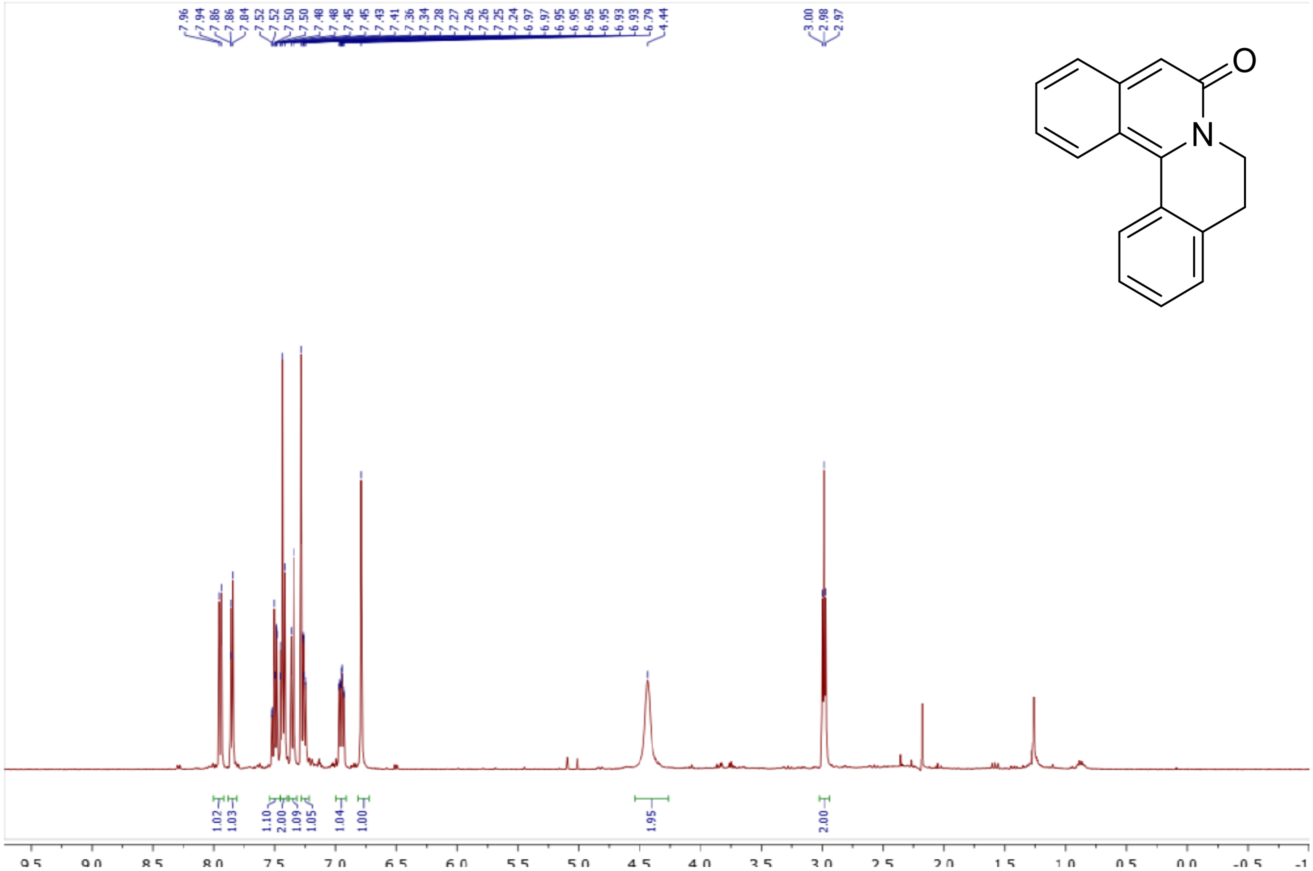

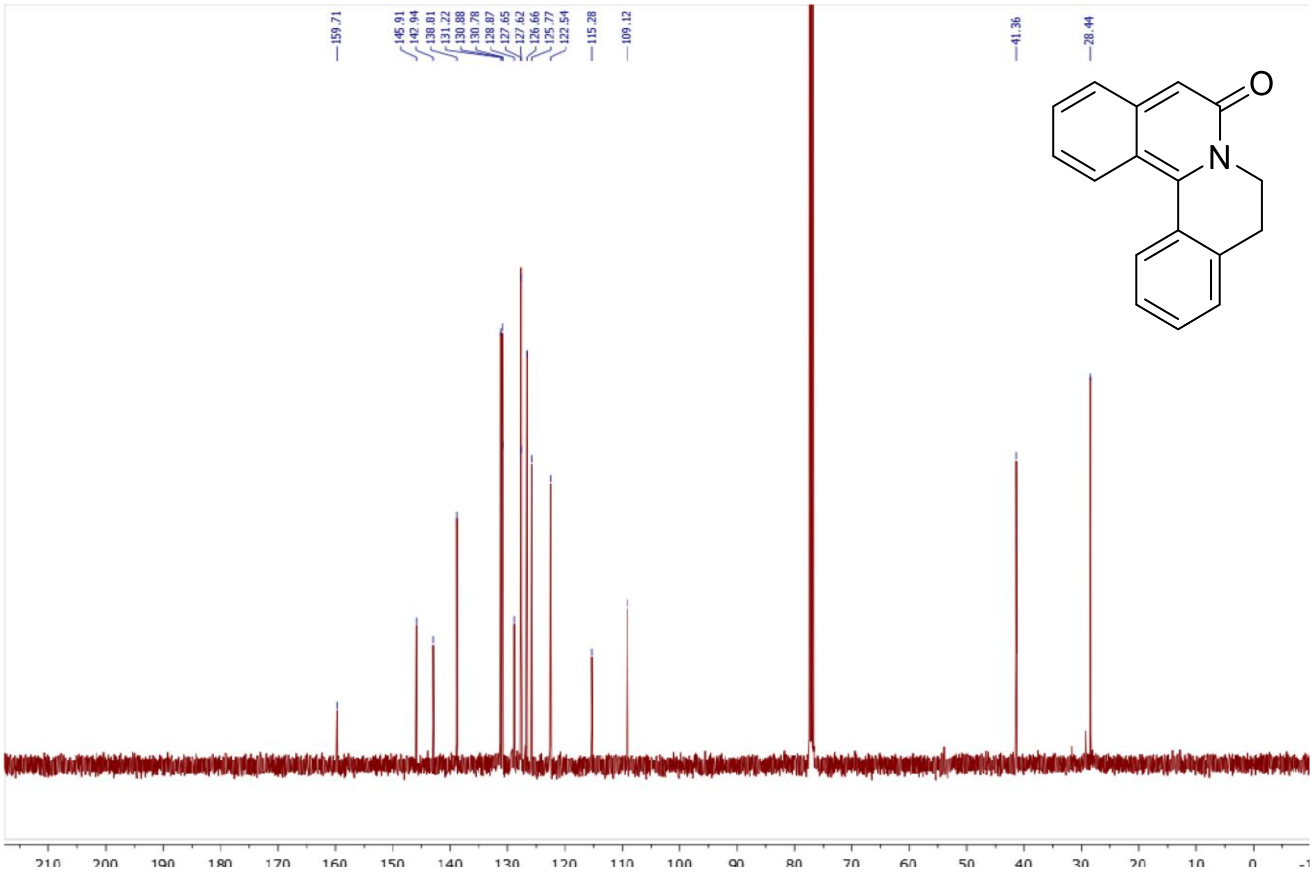

Copies of ^{1}H (400.13 MHz, $CDCl_3$) and $^{13}C\{^{1}H\}$ (100.61 MHz, $CDCl_3$) spectra of **9o**

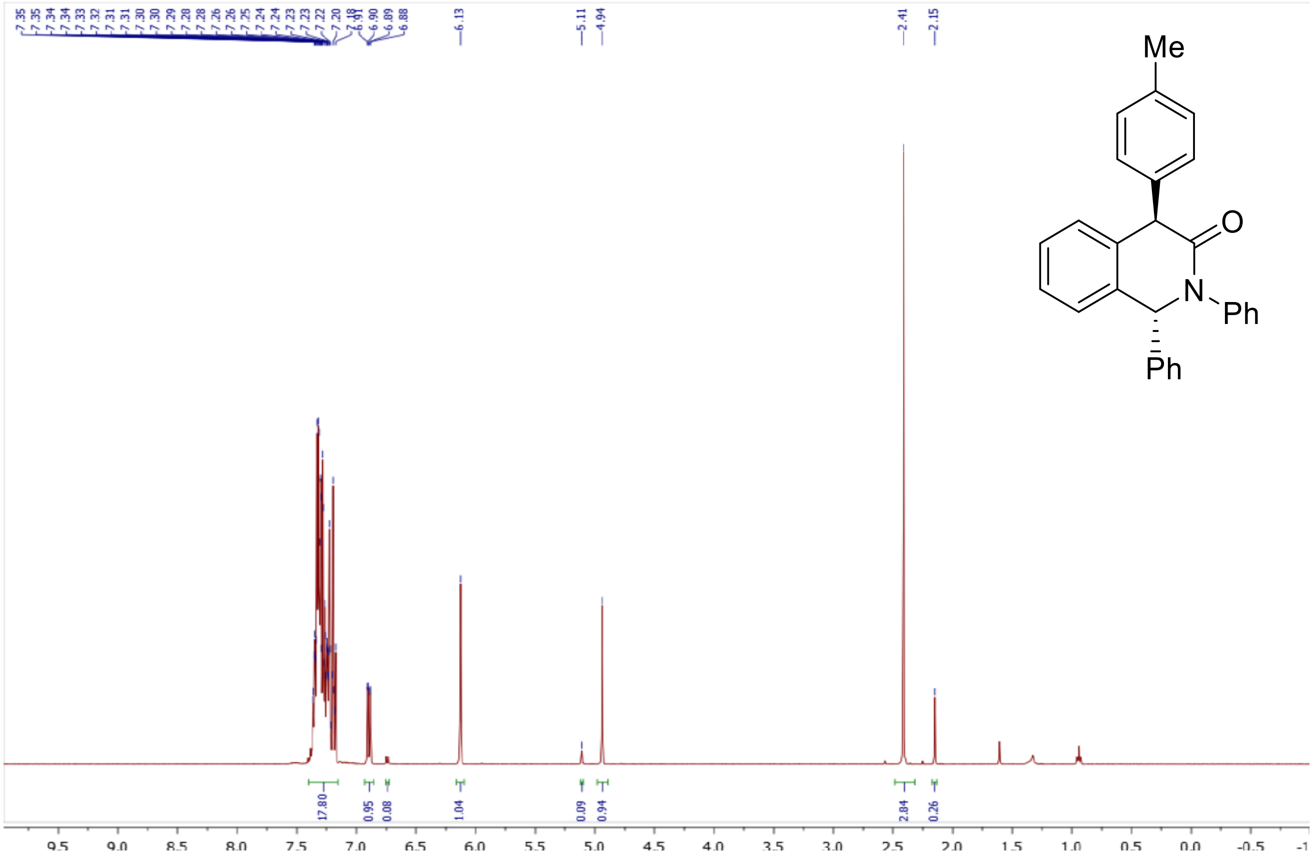

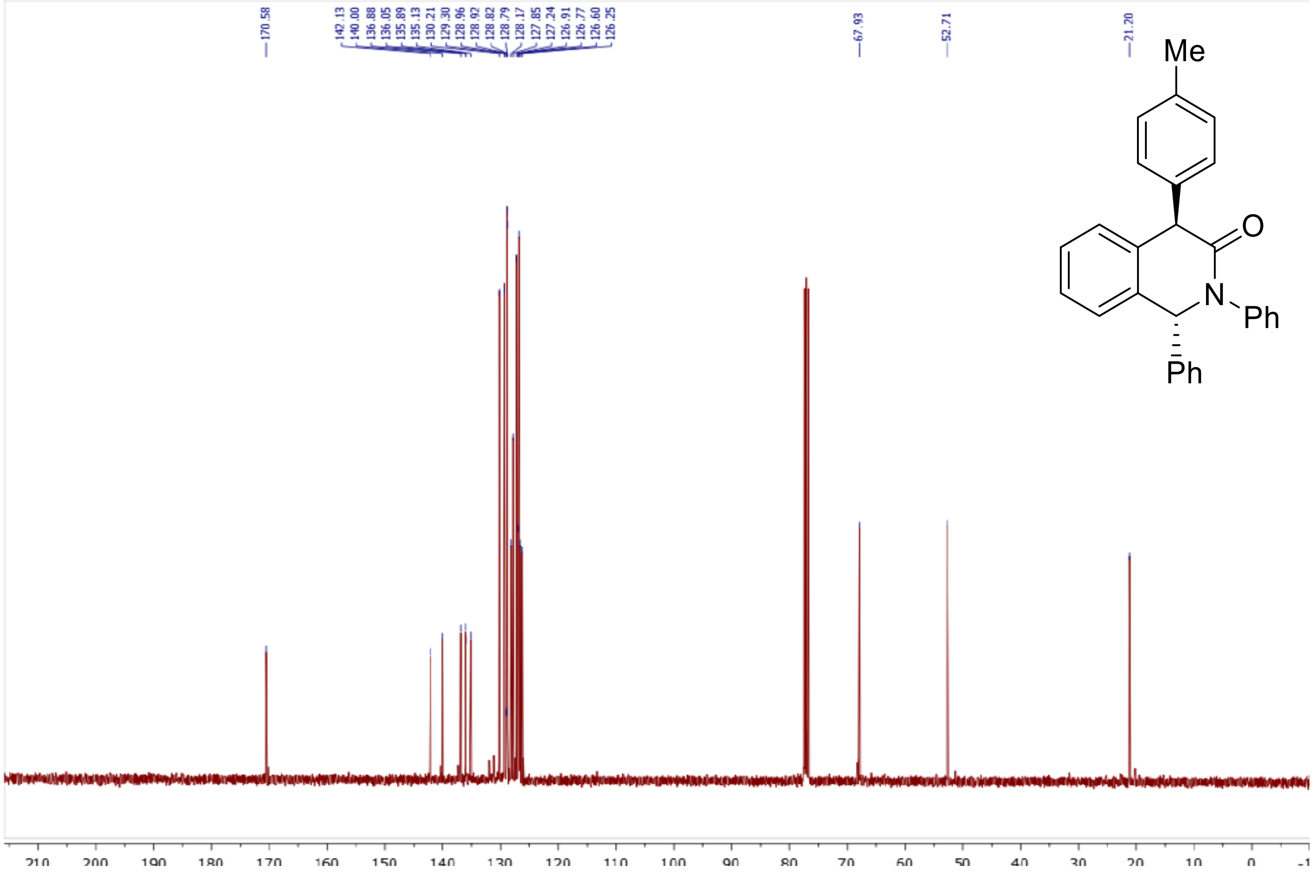

Copies of ^{1}H (400.13 MHz, $CDCl_3$) and $^{13}C\{^{1}H\}$ (100.61 MHz, $CDCl_3$) spectra of **9p**

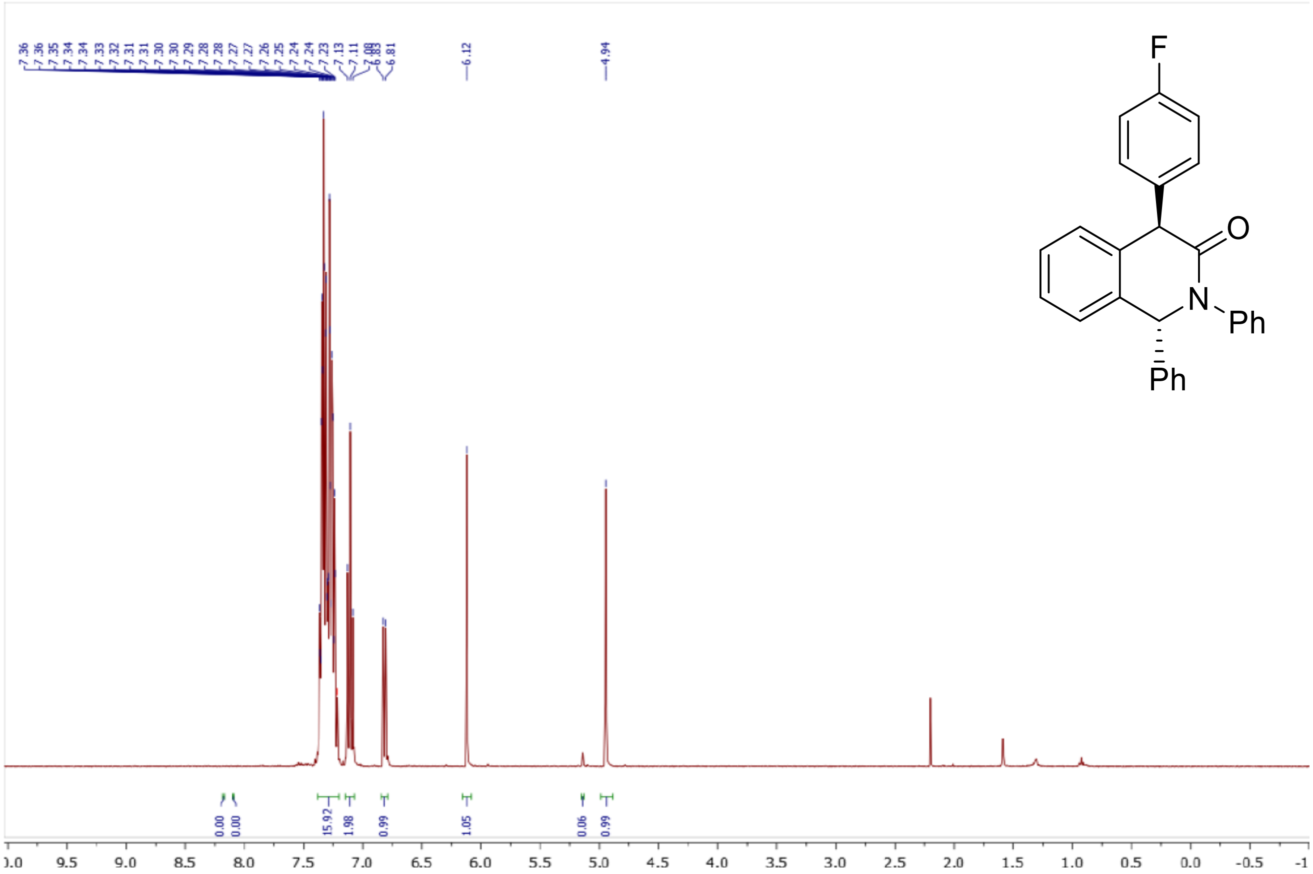

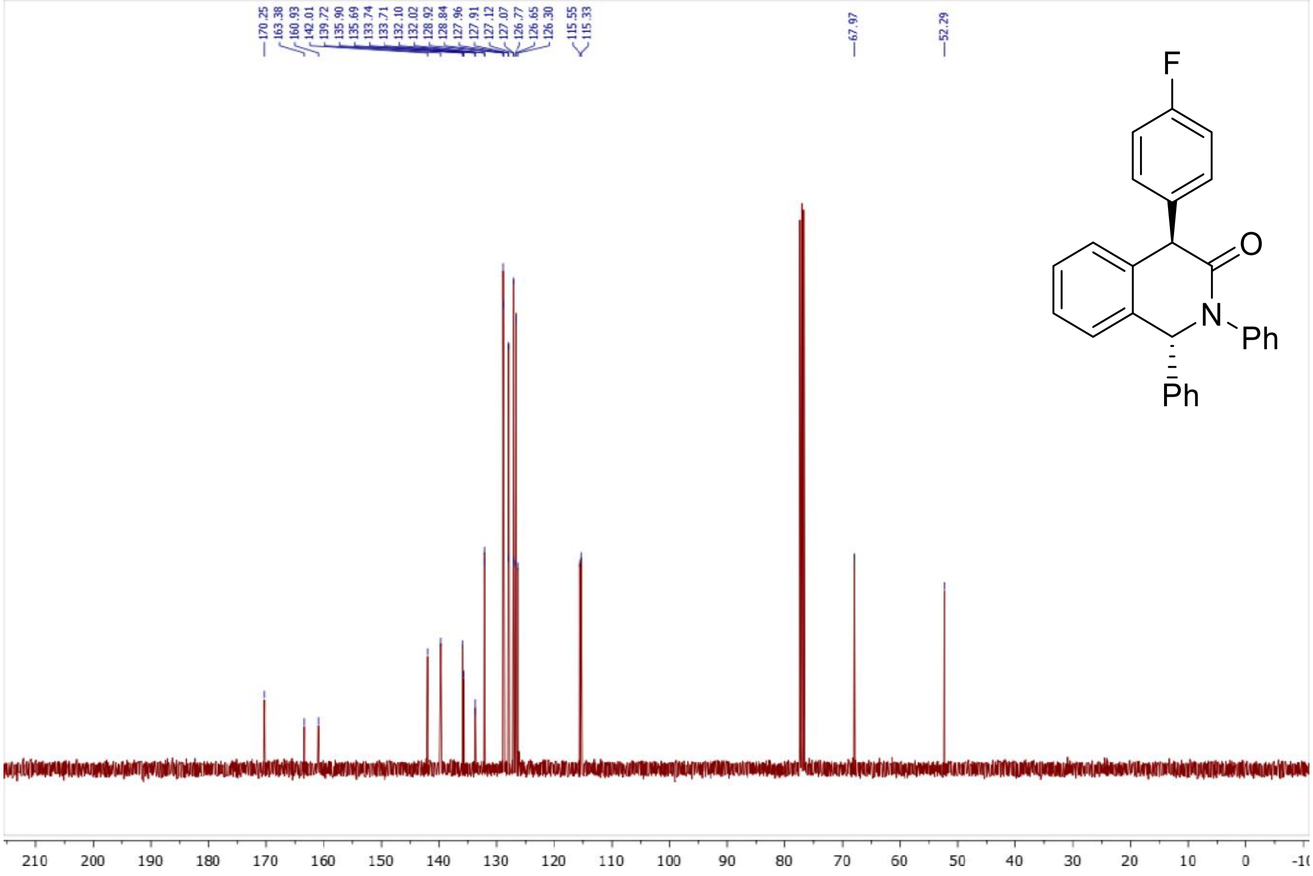

Copy of $^{19}F\{^{1}H\}$ (376.50 MHz, $CDCl_3$) spectrum of **9p**

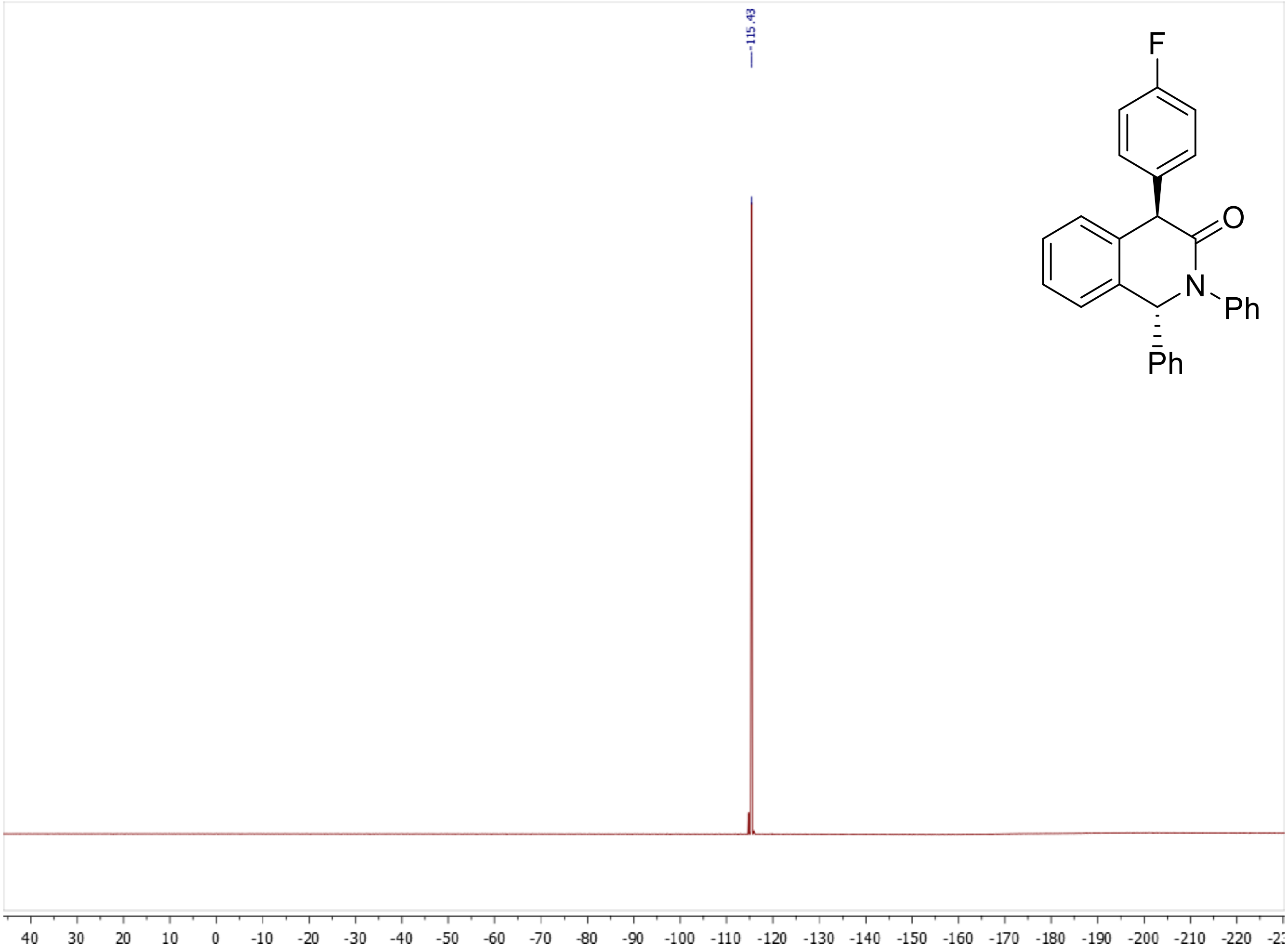

Copies of ^{1}H (400.13 MHz, $CDCl_3$) and $^{13}C\{^{1}H\}$ (100.61 MHz, $CDCl_3$) spectra of **9q**

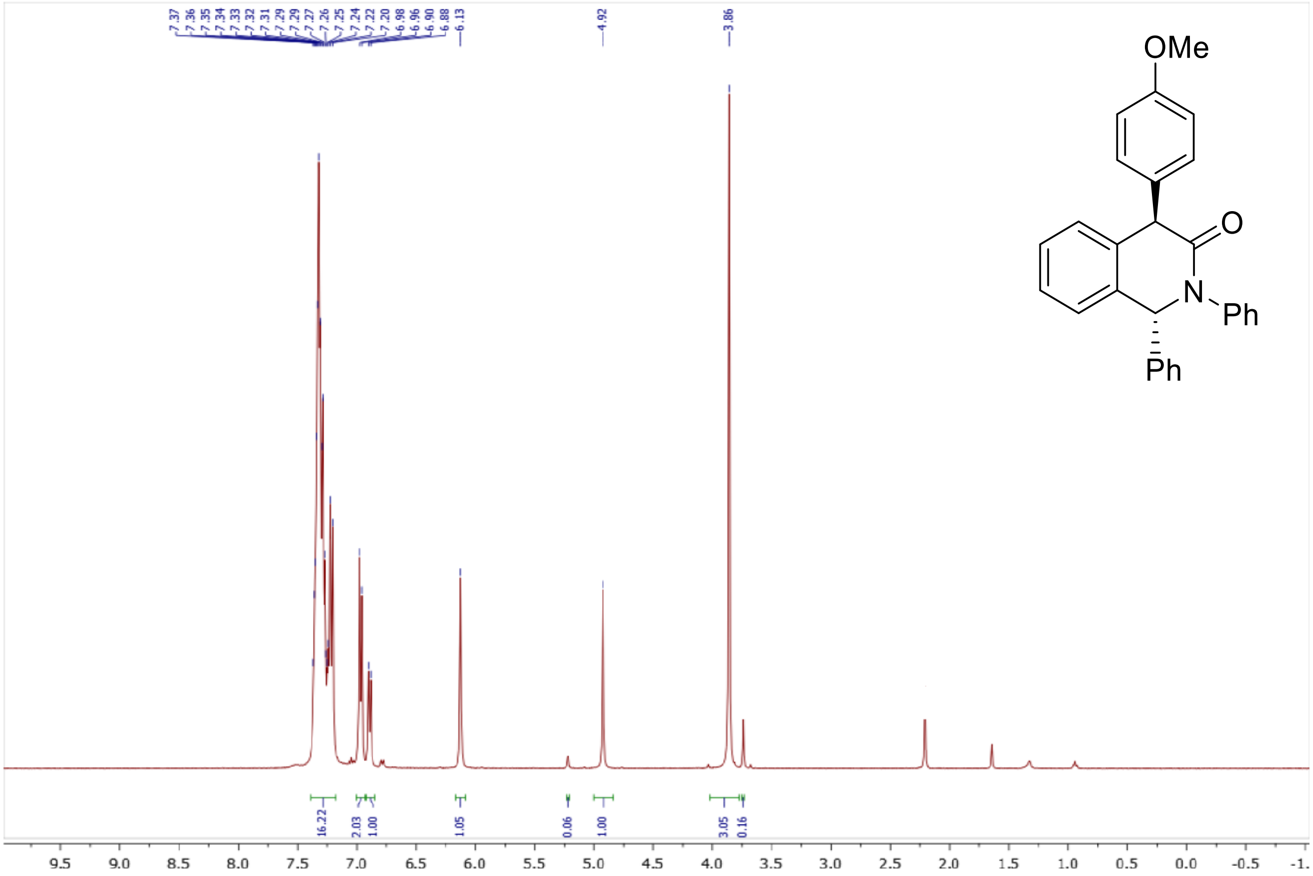

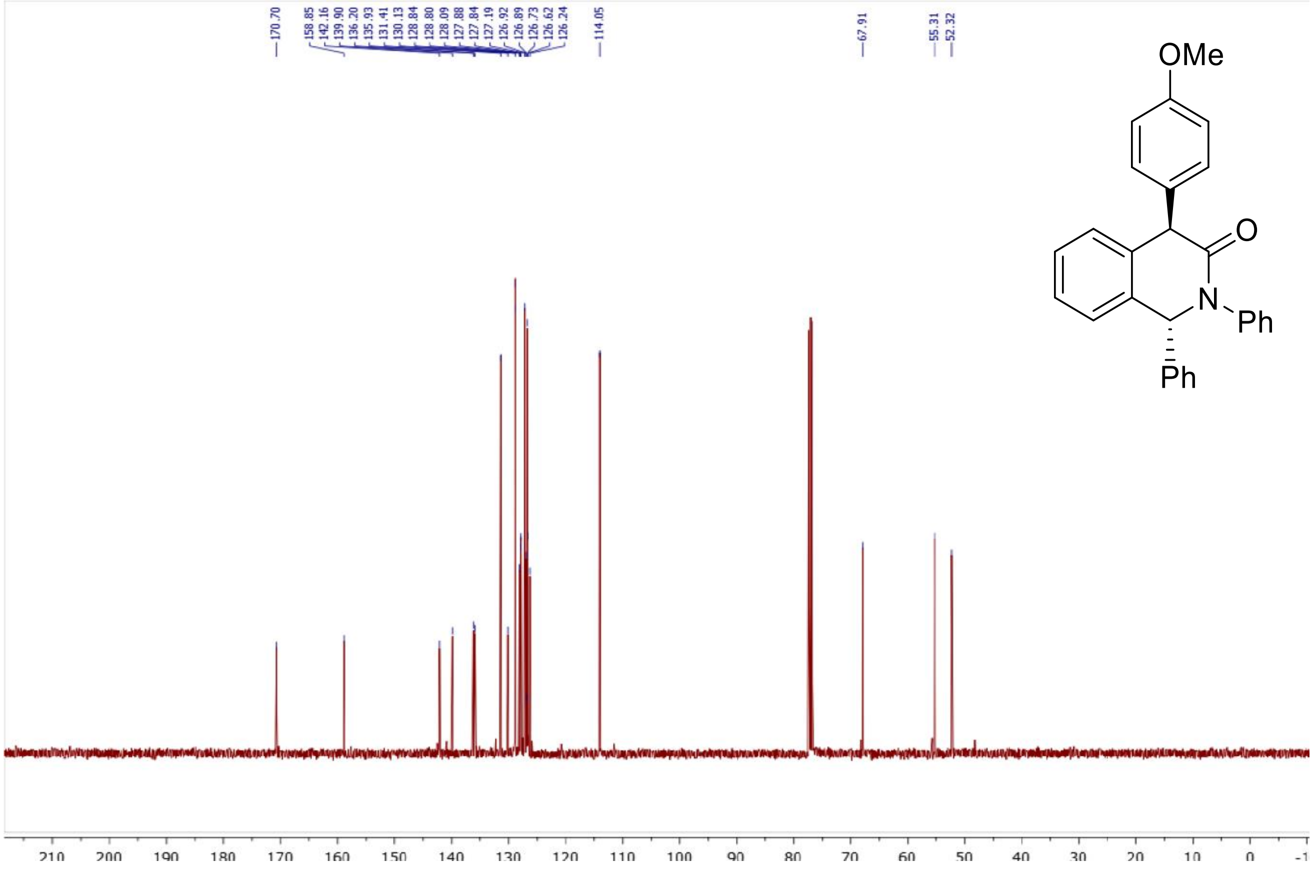

Copies of ^{1}H (400.13 MHz, $CDCl_3$) and ^{13}C{^{1}H} (100.61 MHz, $CDCl_3$) spectra of **9r**

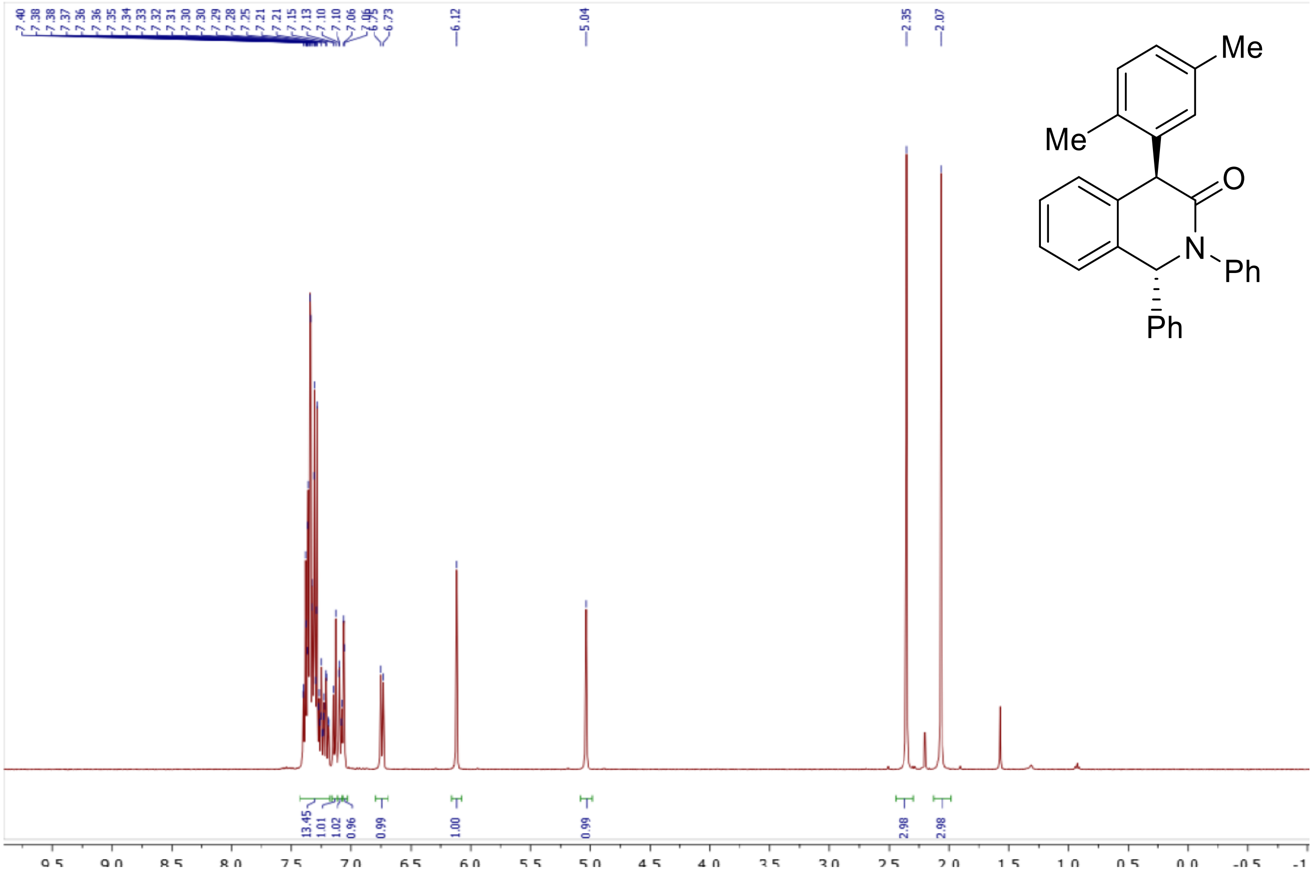

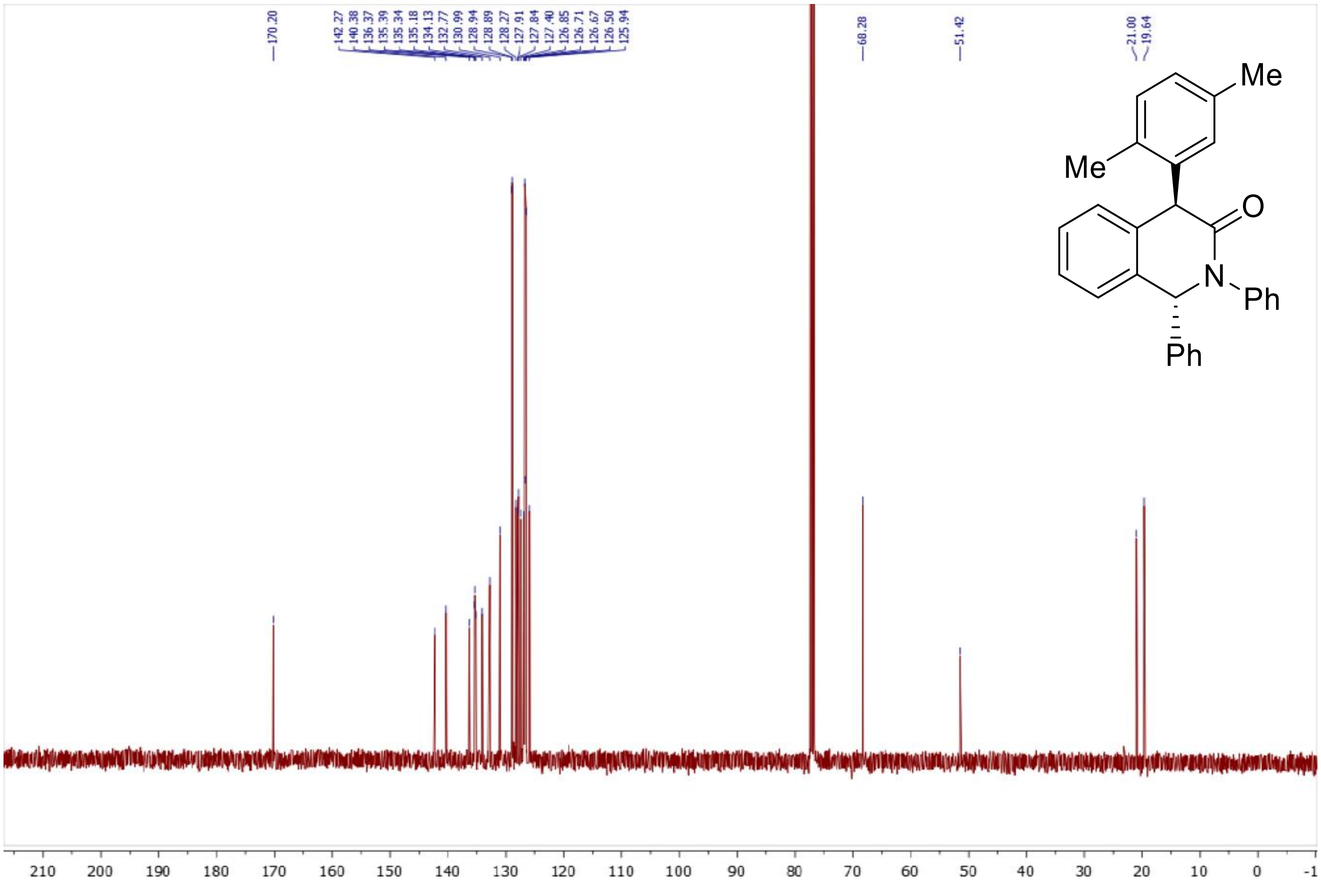

Copies of ^{1}H (400.13 MHz, $CDCl_3$) and $^{13}C\{^{1}H\}$ (100.61 MHz, $CDCl_3$) spectra of **9s**

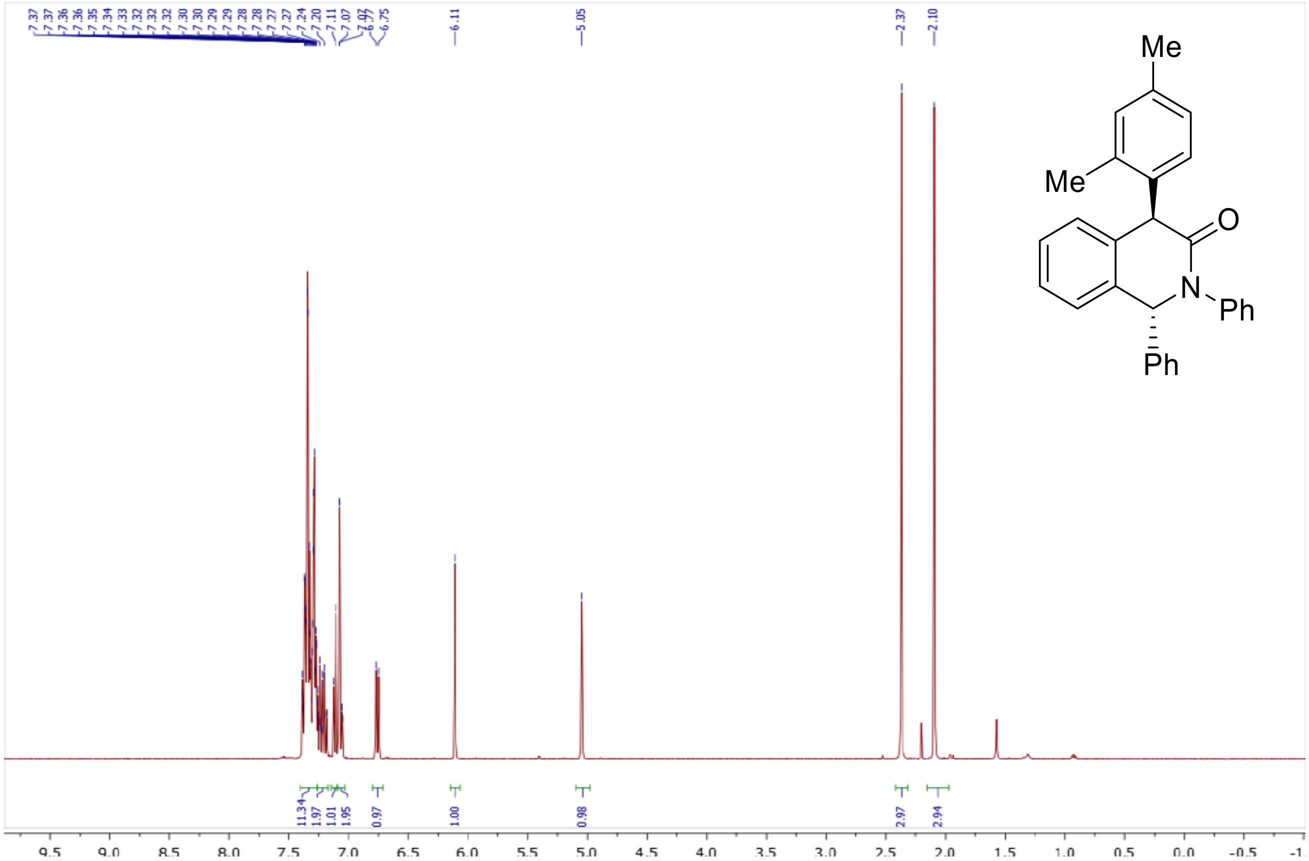

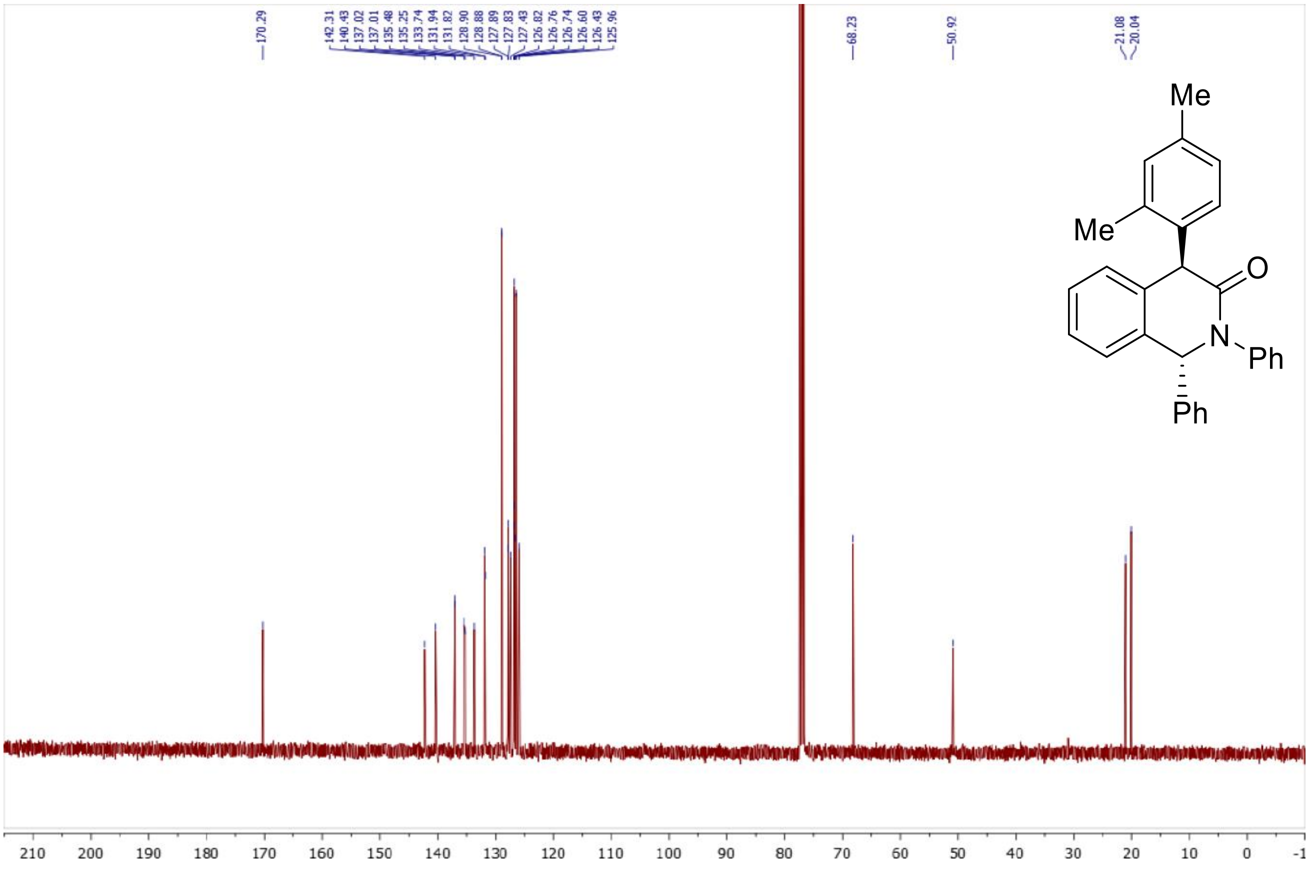

Copies of ^{1}H (400.13 MHz, $CDCl_3$) and $^{13}C\{^{1}H\}$ (100.61 MHz, $CDCl_3$) spectra of **9t**

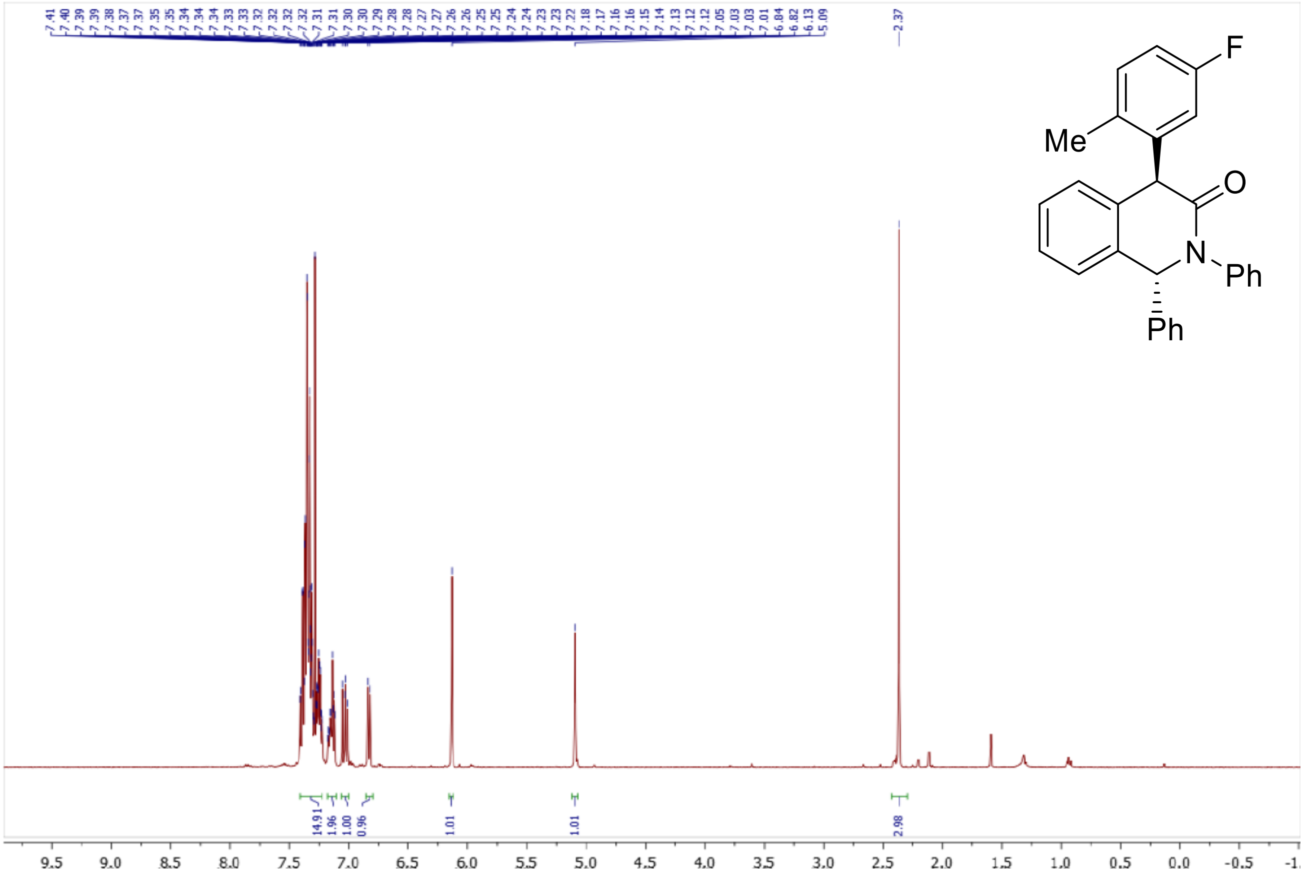

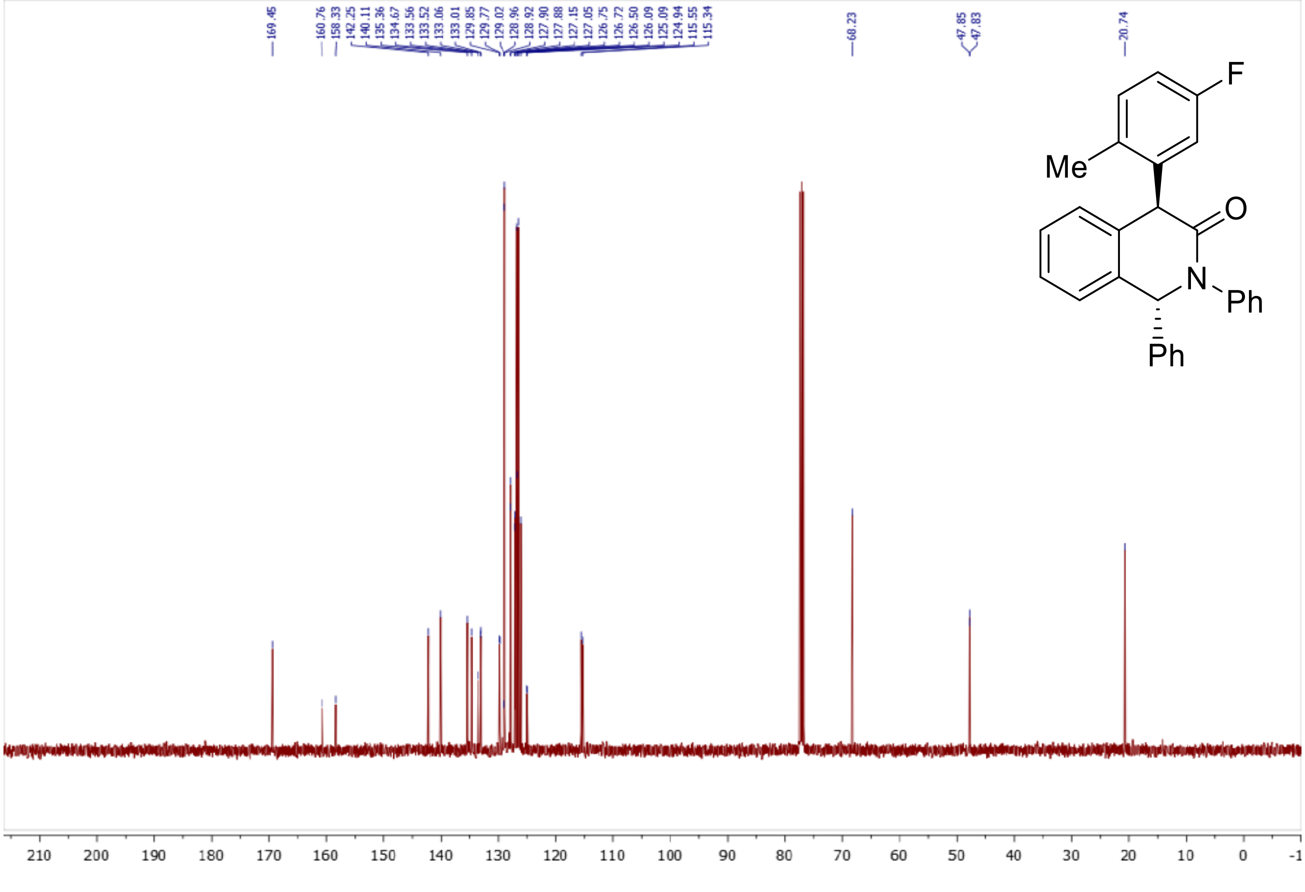

Copy of $^{19}F\{^1H\}$ (376.50 MHz, $CDCl_3$) spectrum of **9t**

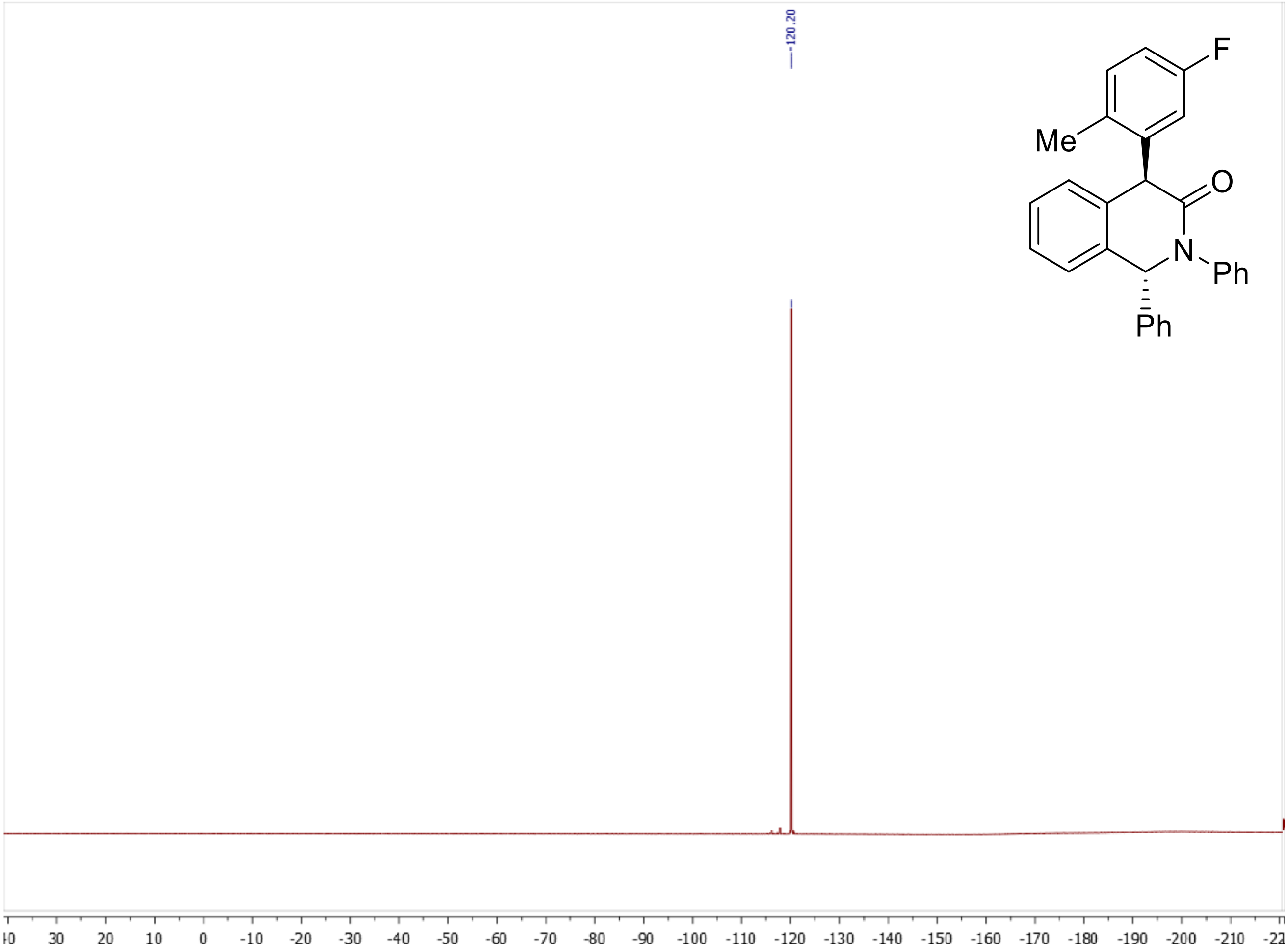

Copies of ^{1}H (400.13 MHz, $CDCl_3$) and ^{13}C{^{1}H} (100.61 MHz, $CDCl_3$) spectra of **9u**

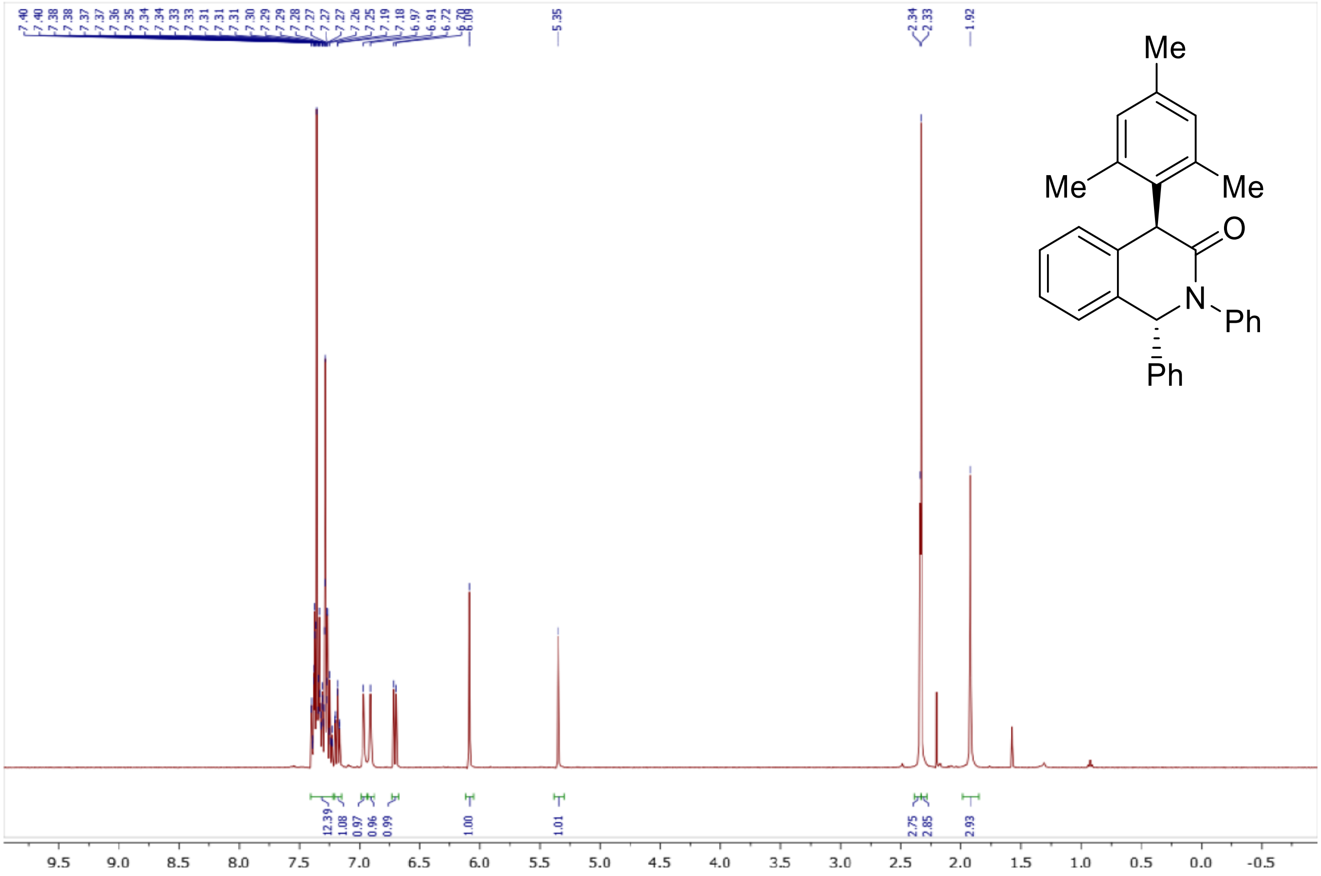

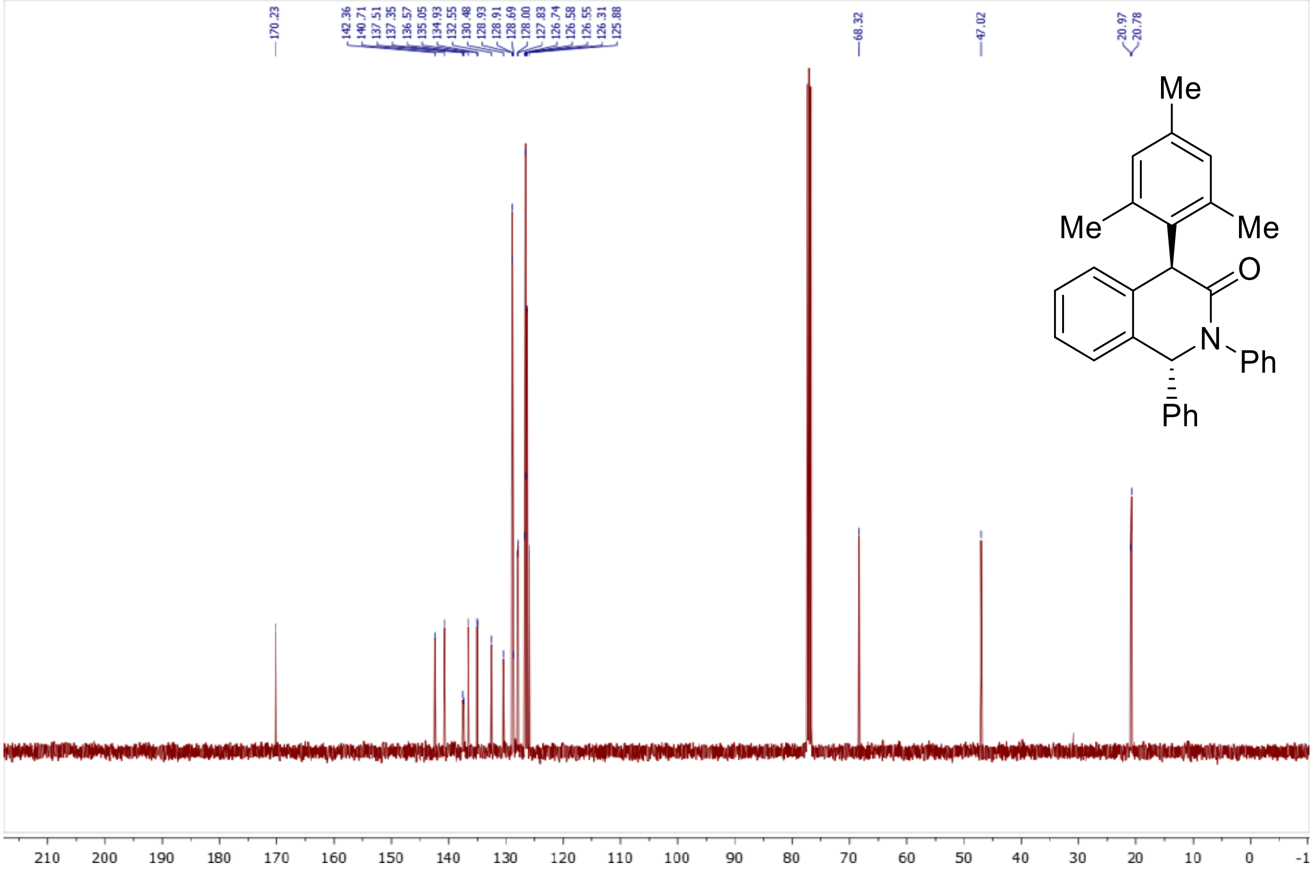

Copies of ^{1}H (400.13 MHz, $CDCl_3$) and ^{13}C{^{1}H} (100.61 MHz, $CDCl_3$) spectra of **9v**

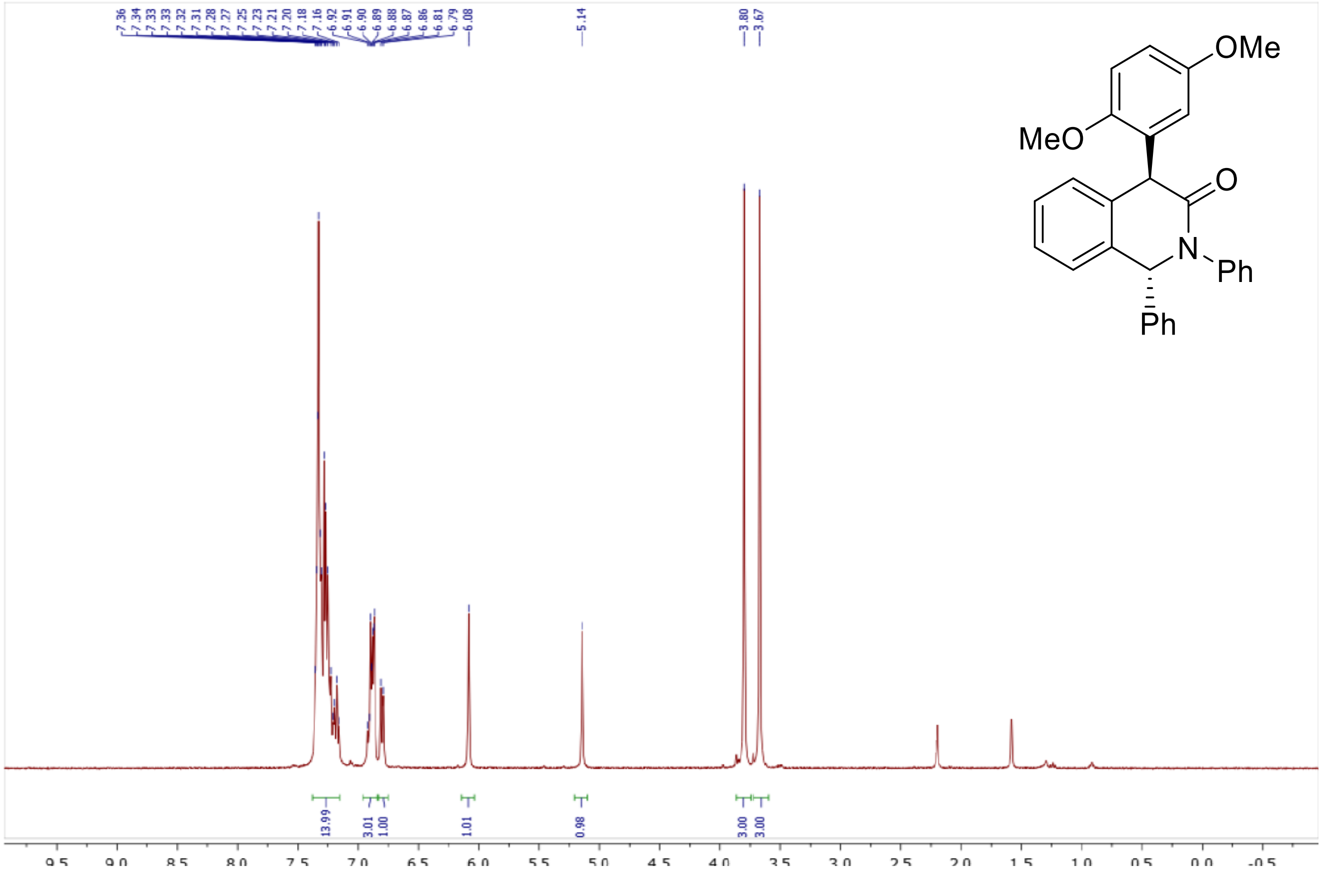

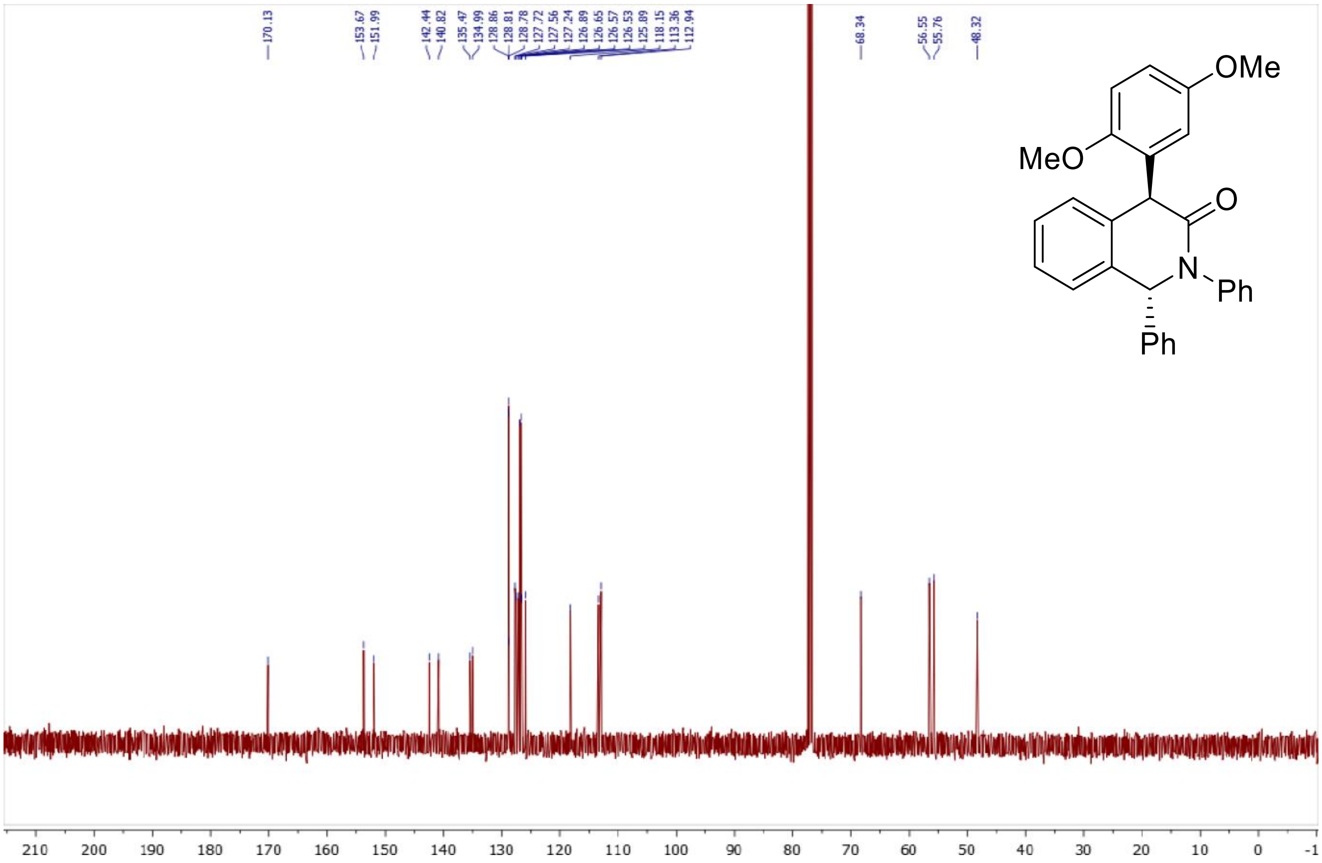

Copies of ^{1}H (400.13 MHz, $CDCl_3$) and $^{13}C\{^{1}H\}$ (100.61 MHz, $CDCl_3$) spectra of **9w**

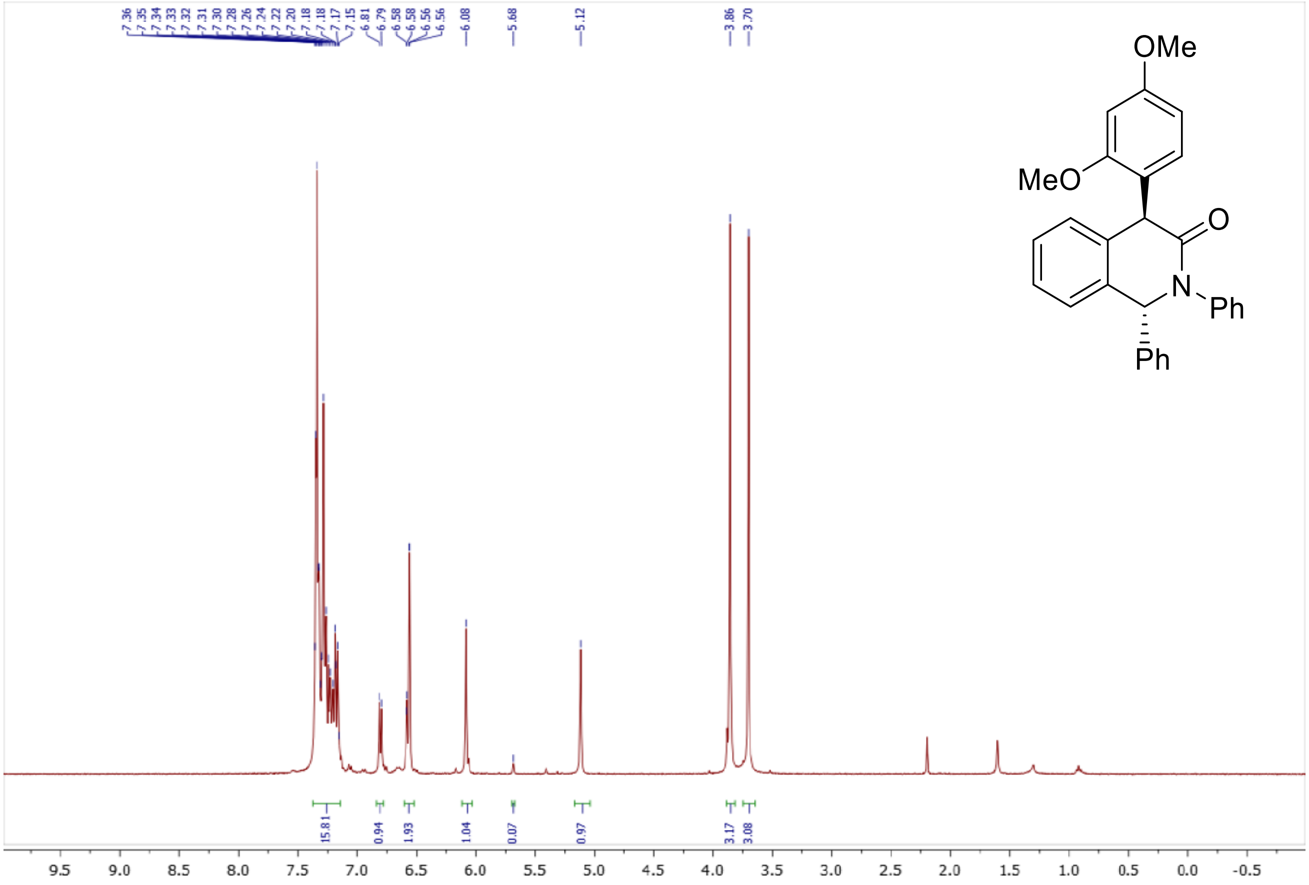

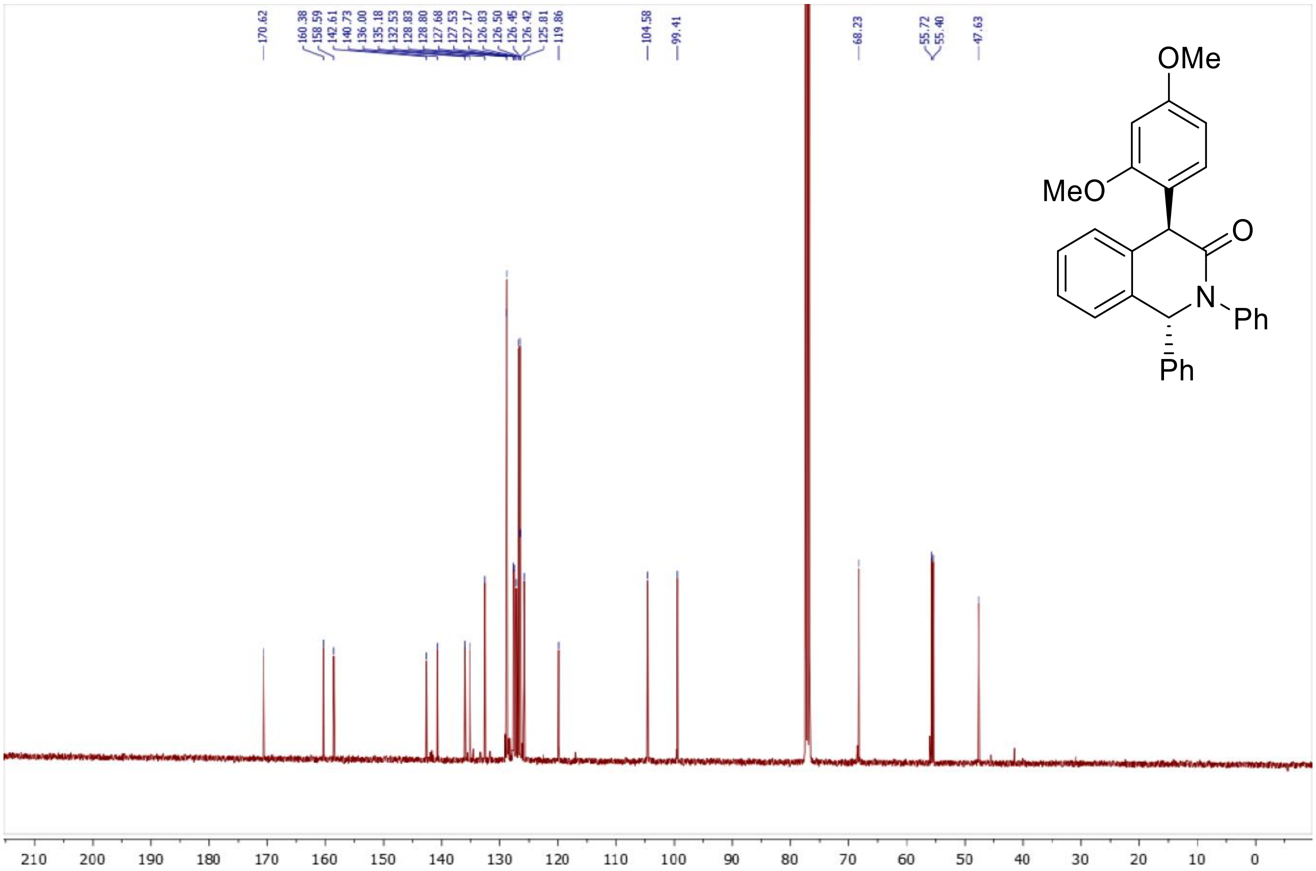

Copies of ^{1}H (400.13 MHz, $CDCl_3$) and $^{13}C\{^{1}H\}$ (100.61 MHz, $CDCl_3$) spectra of **9x**

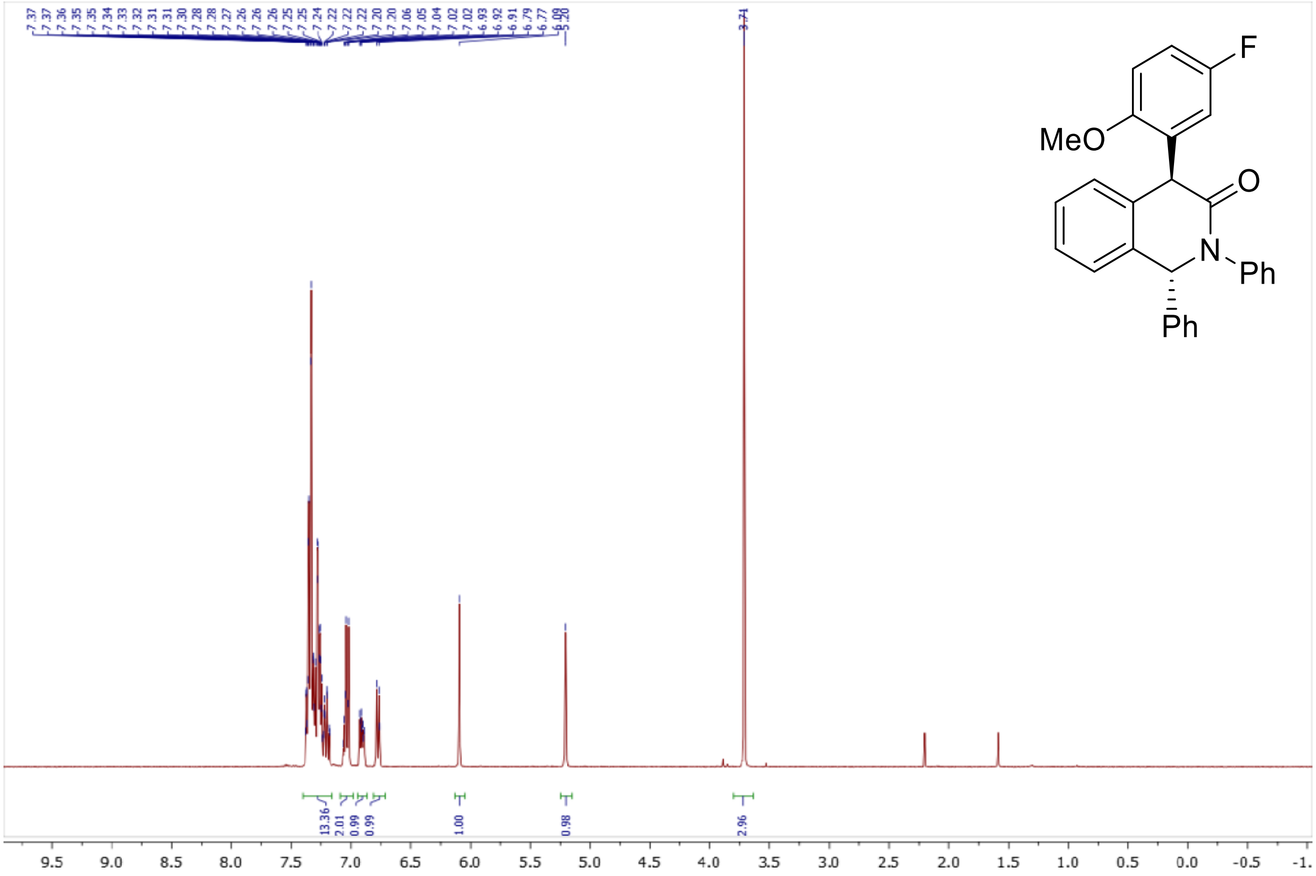

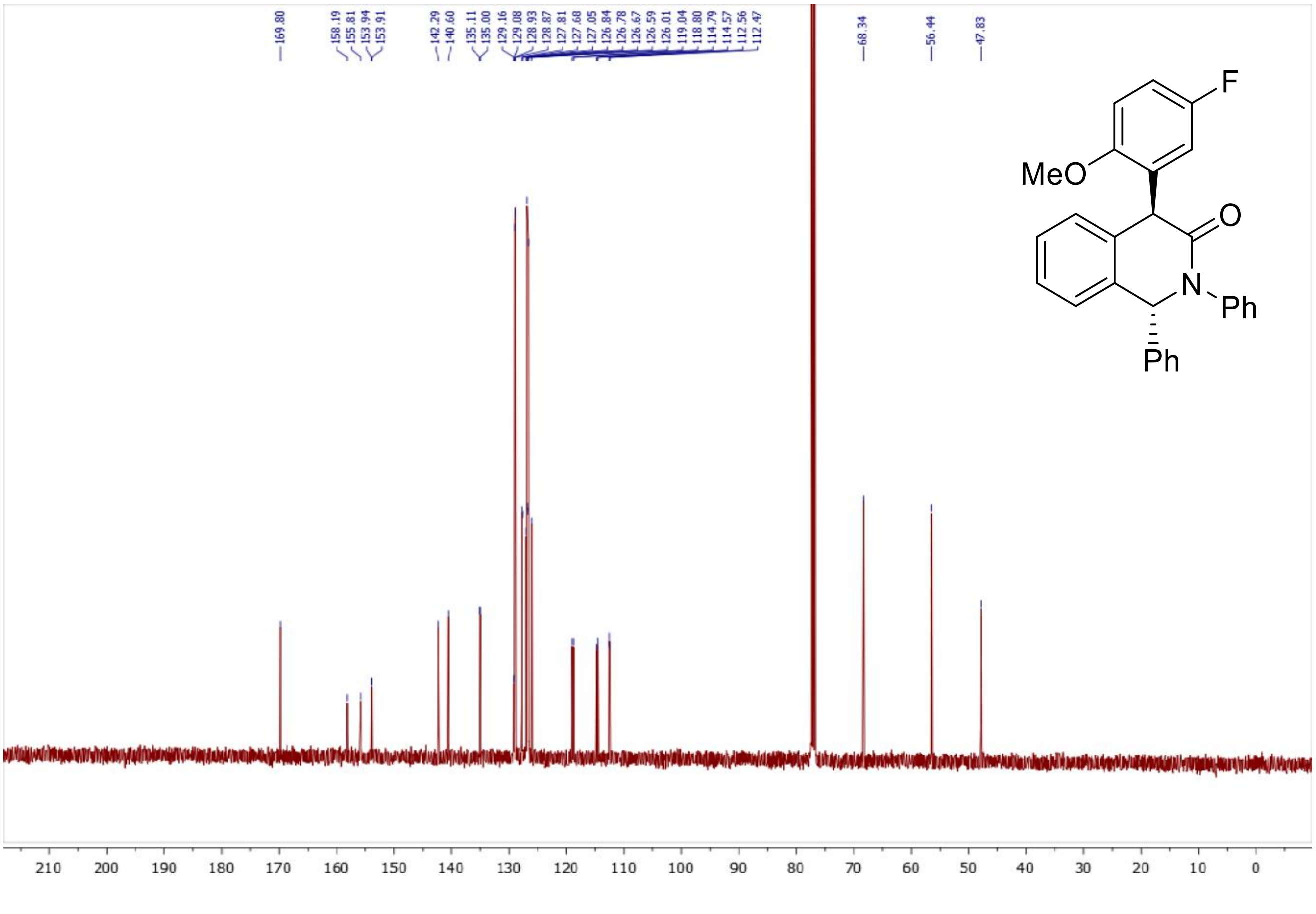

Copy of $^{19}F\{^{1}H\}$ (376.50 MHz, $CDCl_3$) spectrum of **9x**

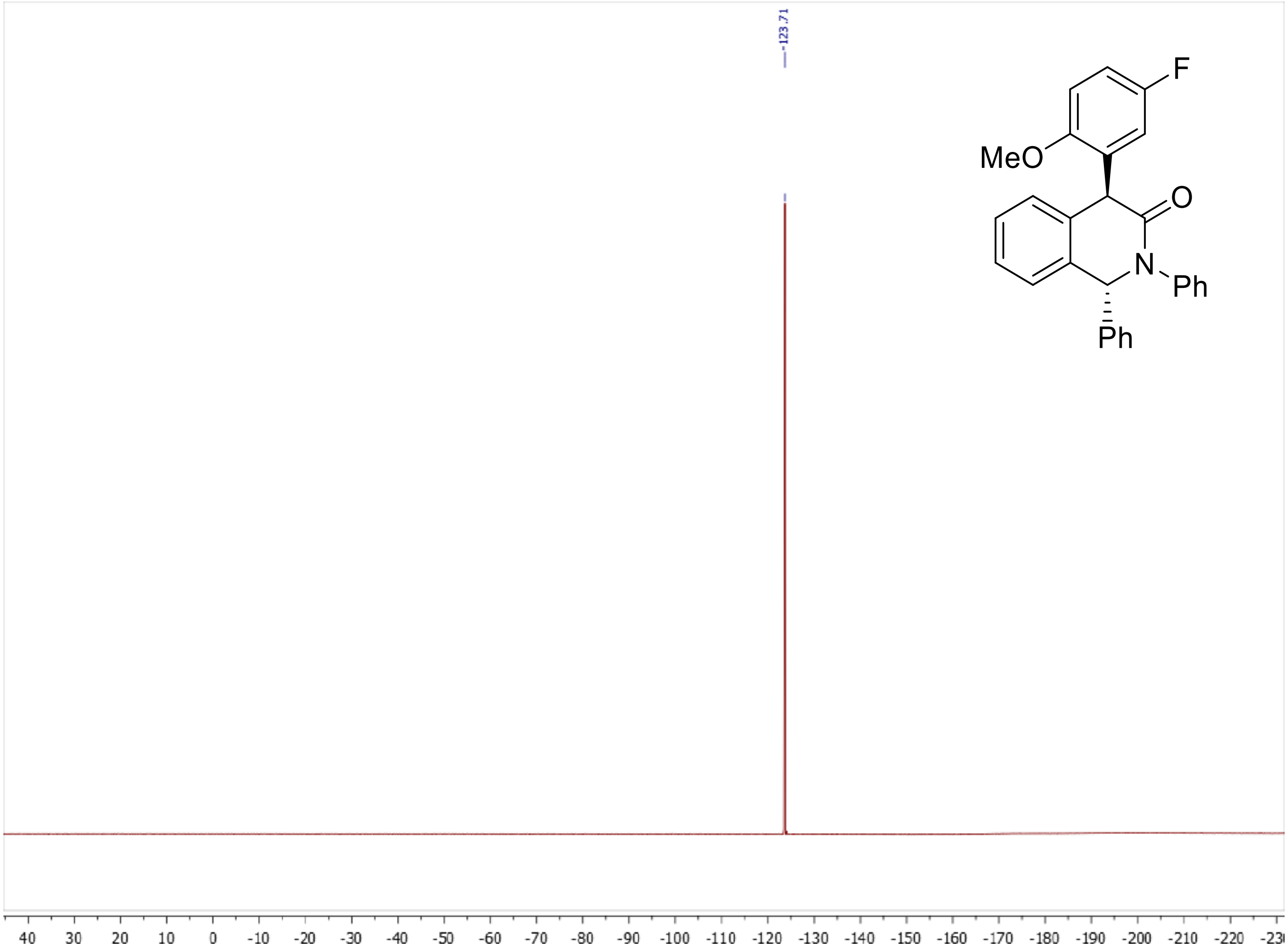

Copies of ^{1}H (400.13 MHz, $CDCl_3$) and $^{13}C\{^{1}H\}$ (100.61 MHz, $CDCl_3$) spectra of **9y**

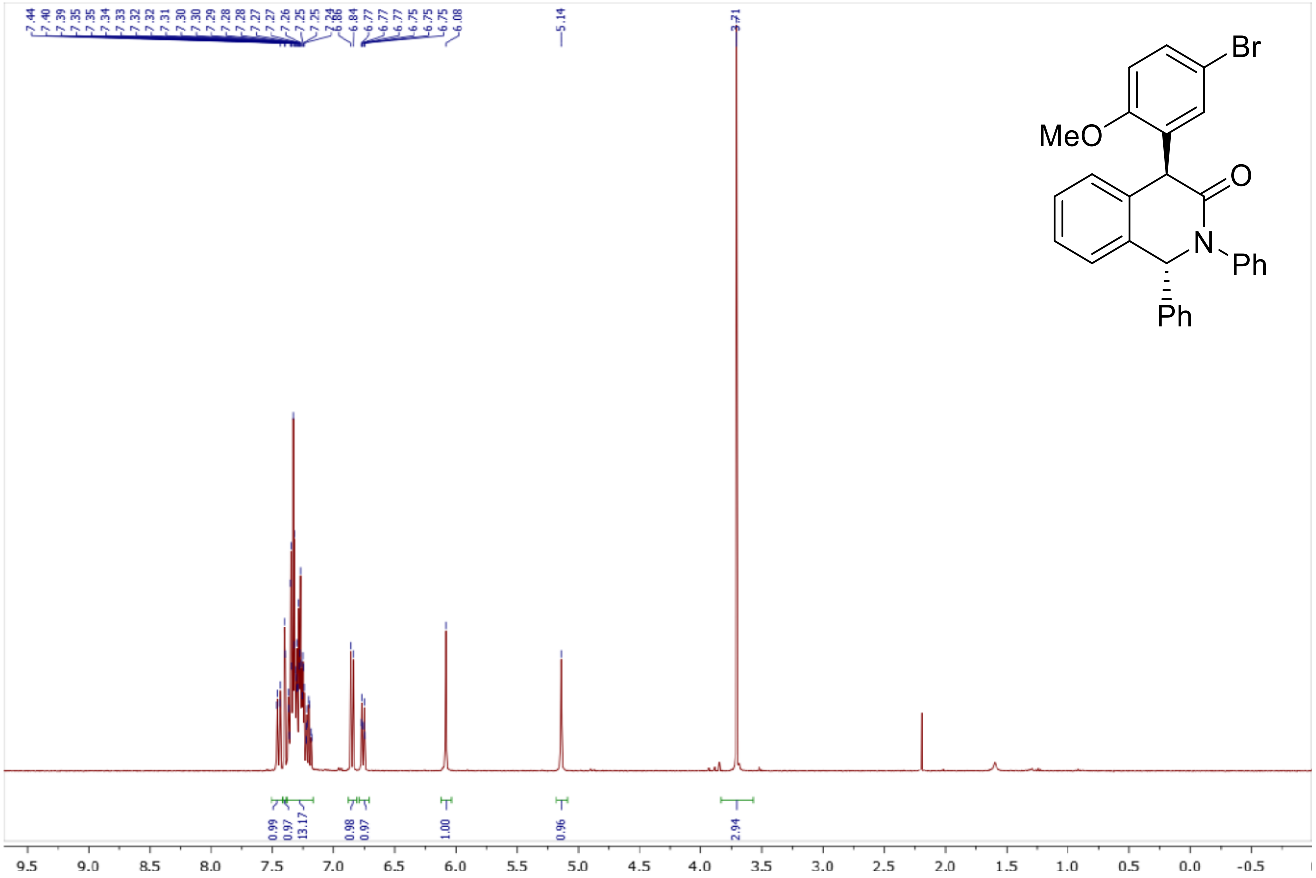

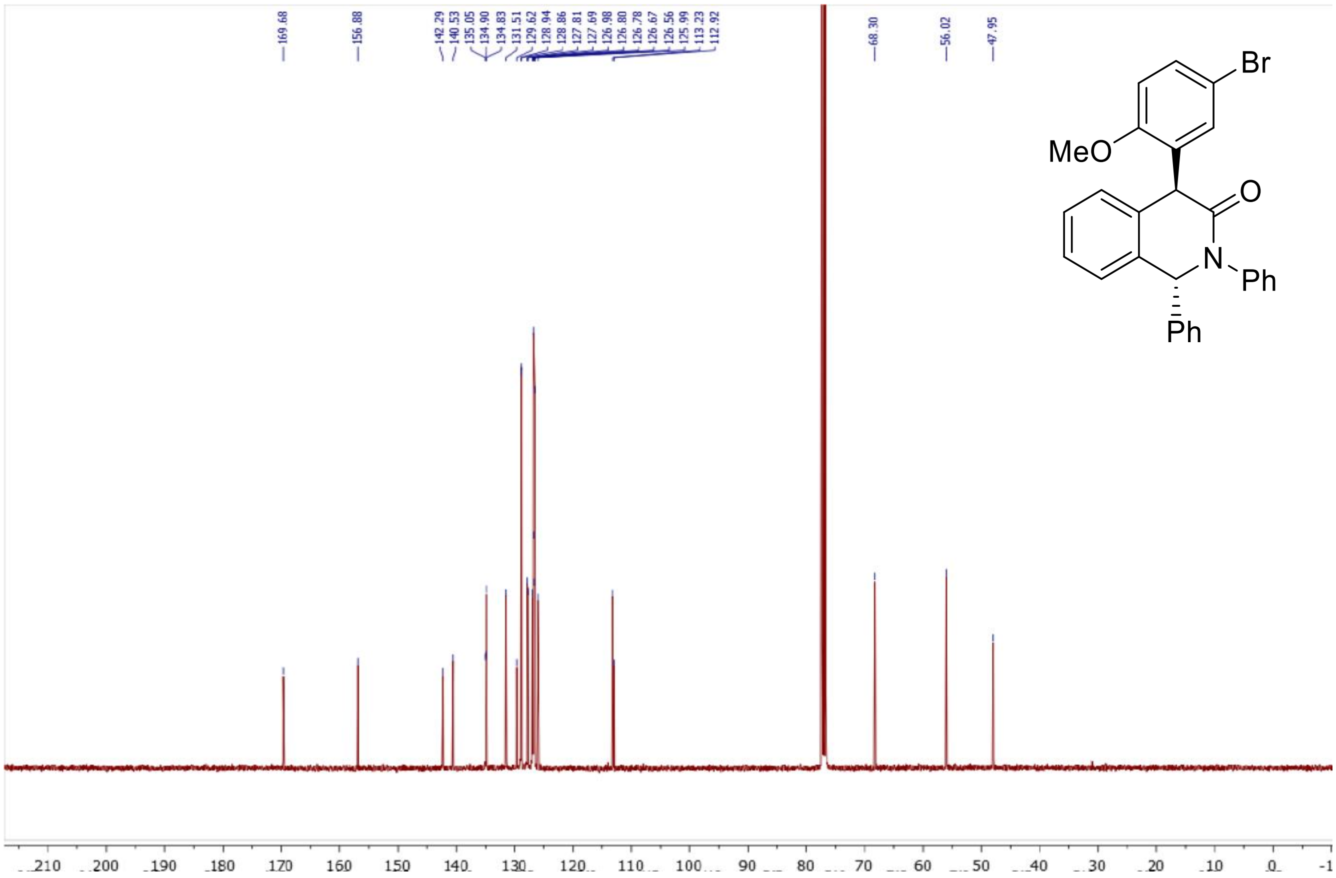

Copies of ^{1}H (400.13 MHz, $CDCl_3$) and $^{13}C\{^{1}H\}$ (100.61 MHz, $CDCl_3$) spectra of **9z**

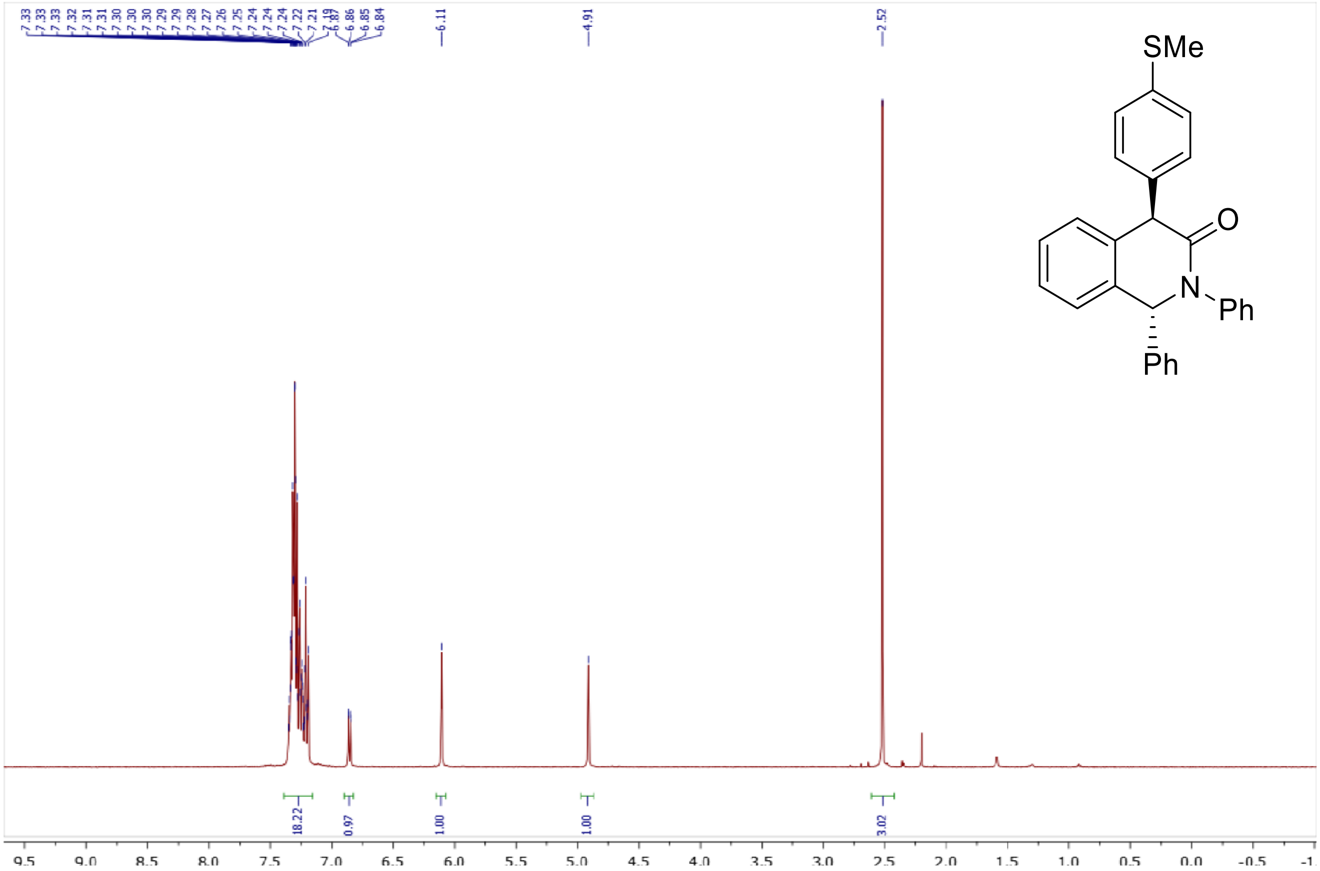

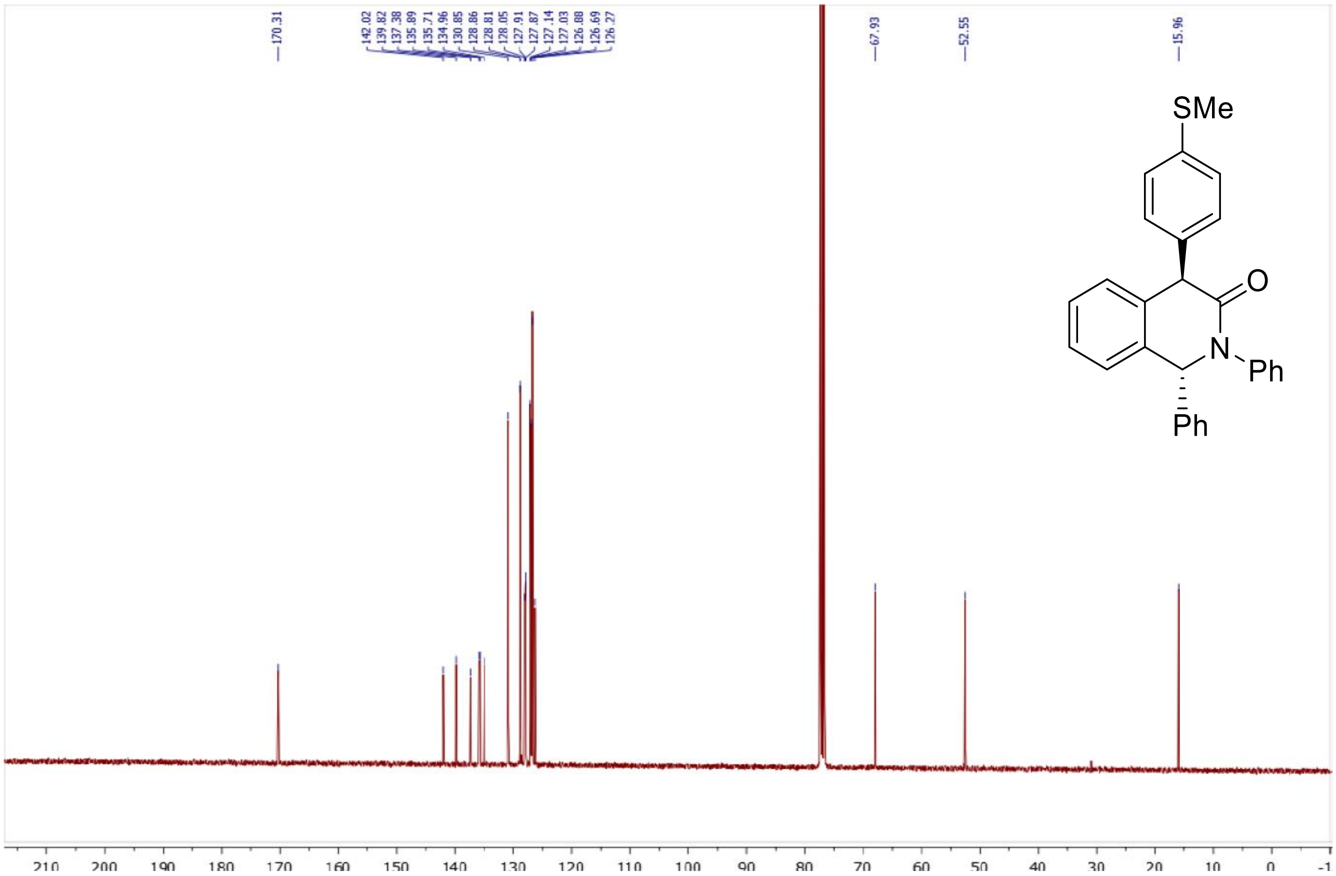

Copies of ^{1}H (400.13 MHz, $CDCl_3$) and $^{13}C\{^{1}H\}$ (100.61 MHz, $CDCl_3$) spectra of **16**

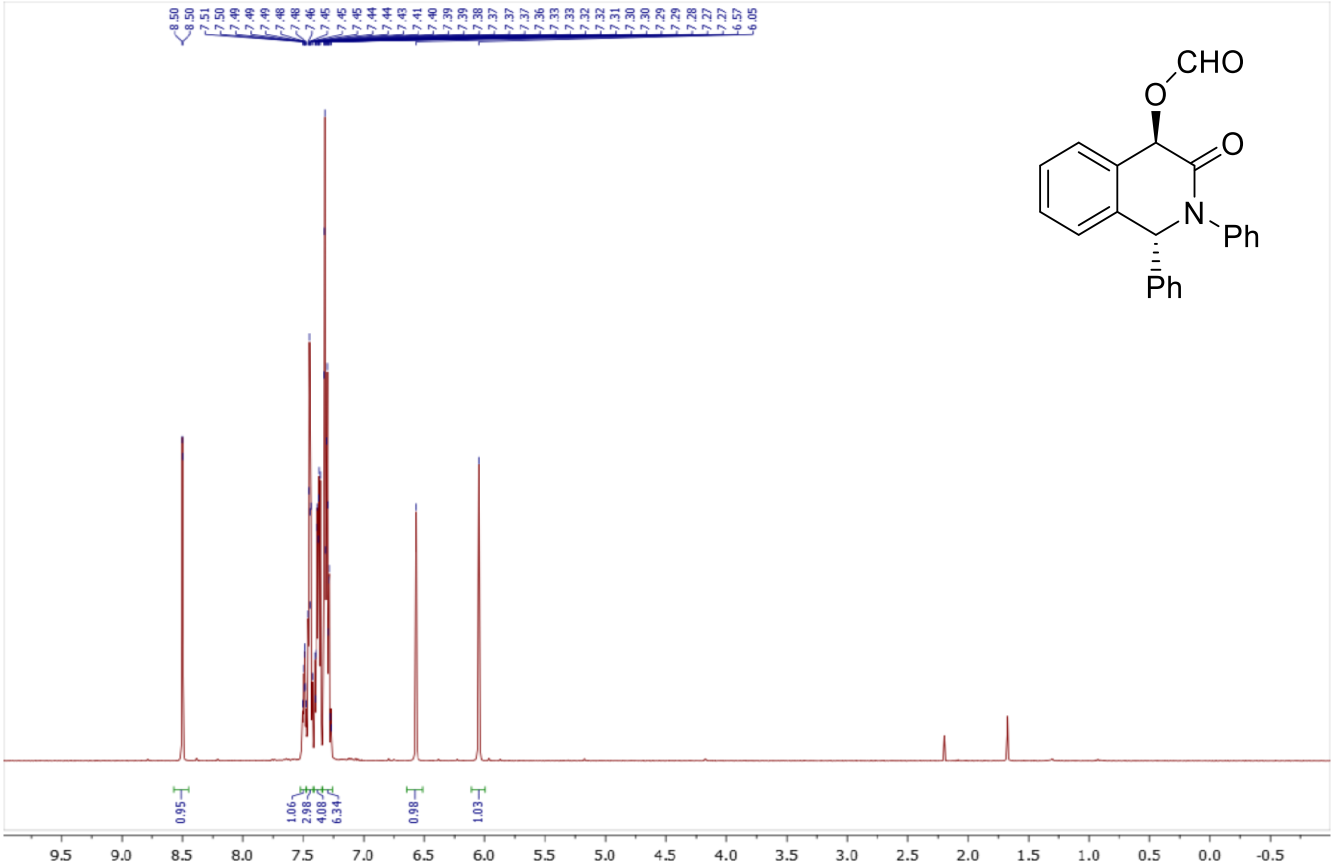

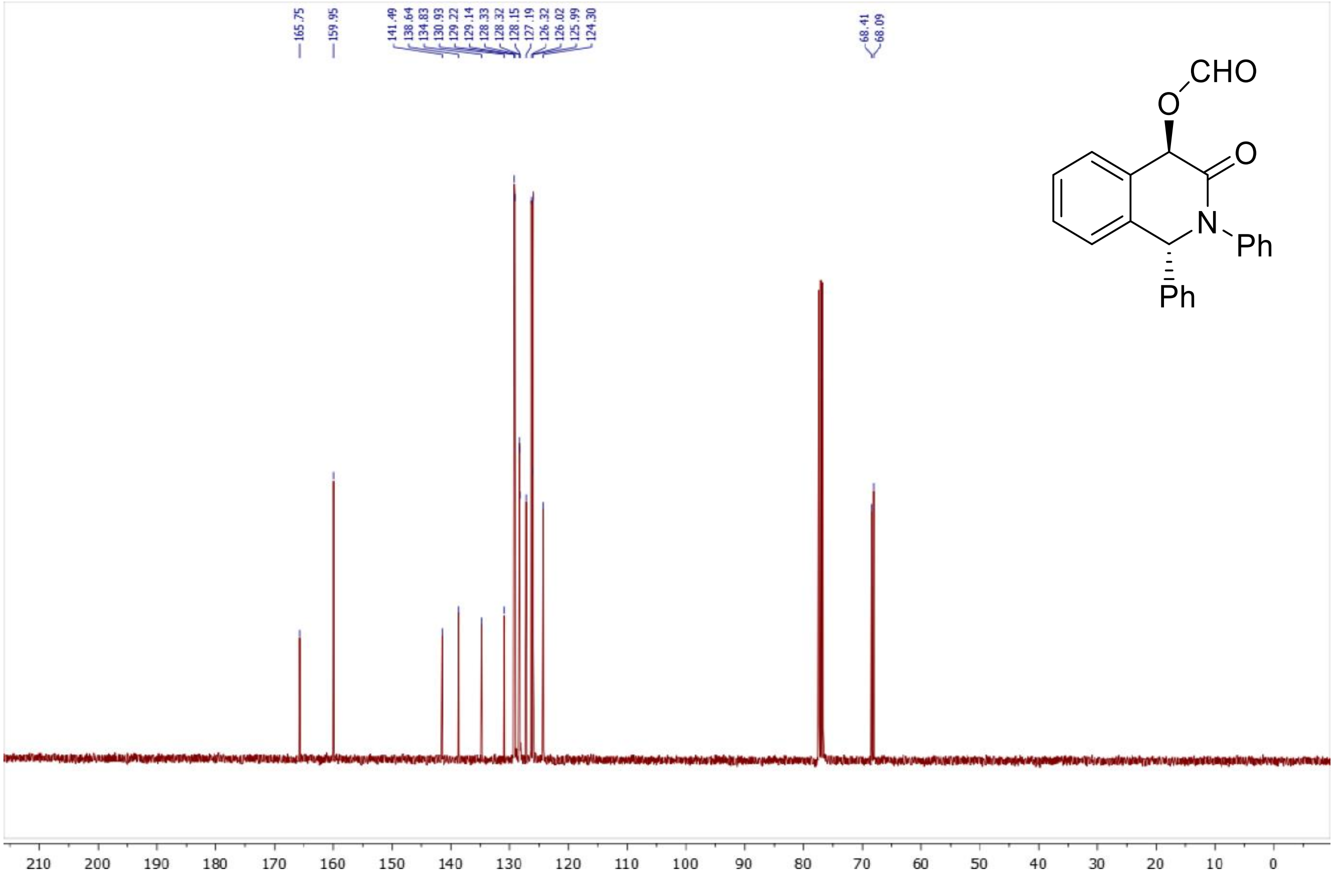

Supplement: File 1 — General experimental information, X-ray crystallographic data, synthetic procedures, analytical data and NMR spectra for the reported compounds. [file Beilstein_J_Org_Chem-18-1070-s001.pdf]
